# Designing a visible light-mediated double photoswitch: a combination of biradical and azobenzene structural motifs that can be switched independently†

**DOI:** 10.1039/d4sc07247b

**Published:** 2024-12-03

**Authors:** Yannic Pilopp, Henrik Beer, Jonas Bresien, Dirk Michalik, Alexander Villinger, Axel Schulz

**Affiliations:** a Institut für Chemie, Universität Rostock Albert-Einstein-Straße 3a Rostock D-18059 Germany axel.schulz@uni-rostock.de; b Leibniz-Institut für Katalyse e.V. an der Universität Rostock Albert-Einstein-Straße 29a Rostock D-18059 Germany

## Abstract

A new molecular switch is presented that combines both biradical and azobenzene motifs to perform visible light-induced constitutional and stereo-isomerisation within the same molecule. The insertion of isonitrile-functionalised azobenzenes into the four-membered biradical [˙P(μ-NTer)_2_P˙] (1), yielding a phosphorus-centred cyclopentane-1,3-diyl (*E*-4B and *E*-5B), represents a straightforward method to generate the desired double switches (*E*-4B and *E*-5B) in excellent yields (>90%). The switching properties are demonstrated for the fluorinated species *E*-5B and, interestingly, can occur either stepwise or simultaneously, depending on the order in which the sample is irradiated with red and/or green light. All possible isomerisation reactions, *i.e.*, housane formation in the phosphorus-centred cyclopentane-1,3-diyl fragment and *E*/*Z* isomerisation at the azobenzene, can be switched by irradiation and the reaction products *E*-5H, *Z*-5H and *Z*-5B (when performing the thermal reverse reaction in the dark) are identified using ^19^F{^1^H} and ^31^P{^1^H} NMR spectroscopy. Results from quantum chemical calculations contribute to the understanding and visualisation of the different isomers of each of the observed compounds (*E*-5B, *E*-5H, *Z*-5H, and *Z*-5B) caused by the unique structure of the double switches.

## Abstract 2

Visible light of different wavelengths can trigger either constitutional (biradical/housane) or stereo isomerisation (*E*/*Z*-azobenzene) in the new double photoswitch.

## Graphical abstract

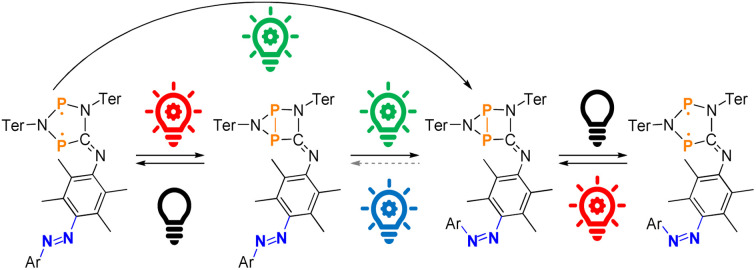

## Introduction

The basic concept of switchable molecules, their reaction behavior towards an external influence as well as their use in various applications has been the subject of intense investigations in the past.^1–6^ Especially the development of new photochromic switches has produced a wide range of compounds containing multiple switchable units.^7–14^ Systems based on hemithioindigo, which can undergo several spatial changes, or molecules based on dithienylethene fulgimide, which undergo several constitutional changes upon irradiation, are just two examples from this field of research.^7–9^ Dithienylethene based photochromic switches have been used further to design novel molecular motors using light from the visible part of the electromagnetic spectrum for operation.^10,15^ Also the use for the invention of photochromic polymeric, chiral, liquid crystal or supramolecular switches has made dithienylethenes a substantial building block when designing new photochromic materials.^16–19^ Moreover, new photochromic materials and polymeric switches can also be designed on the basis of other switchable molecules such as azobenzenes.^16,20^ For example, multiphotochromic diazenes or azobenzenes play an increasing part in the research field of solar energy storage. The concept of capturing energy from photons with the conversion from the *E*- to the *Z*-isomer of azobenzenes and the release of energy *via* thermal radiation for the *Z* to *E* switching promises groundbreaking innovations in the future.^16,21–27^

**Scheme 1:**
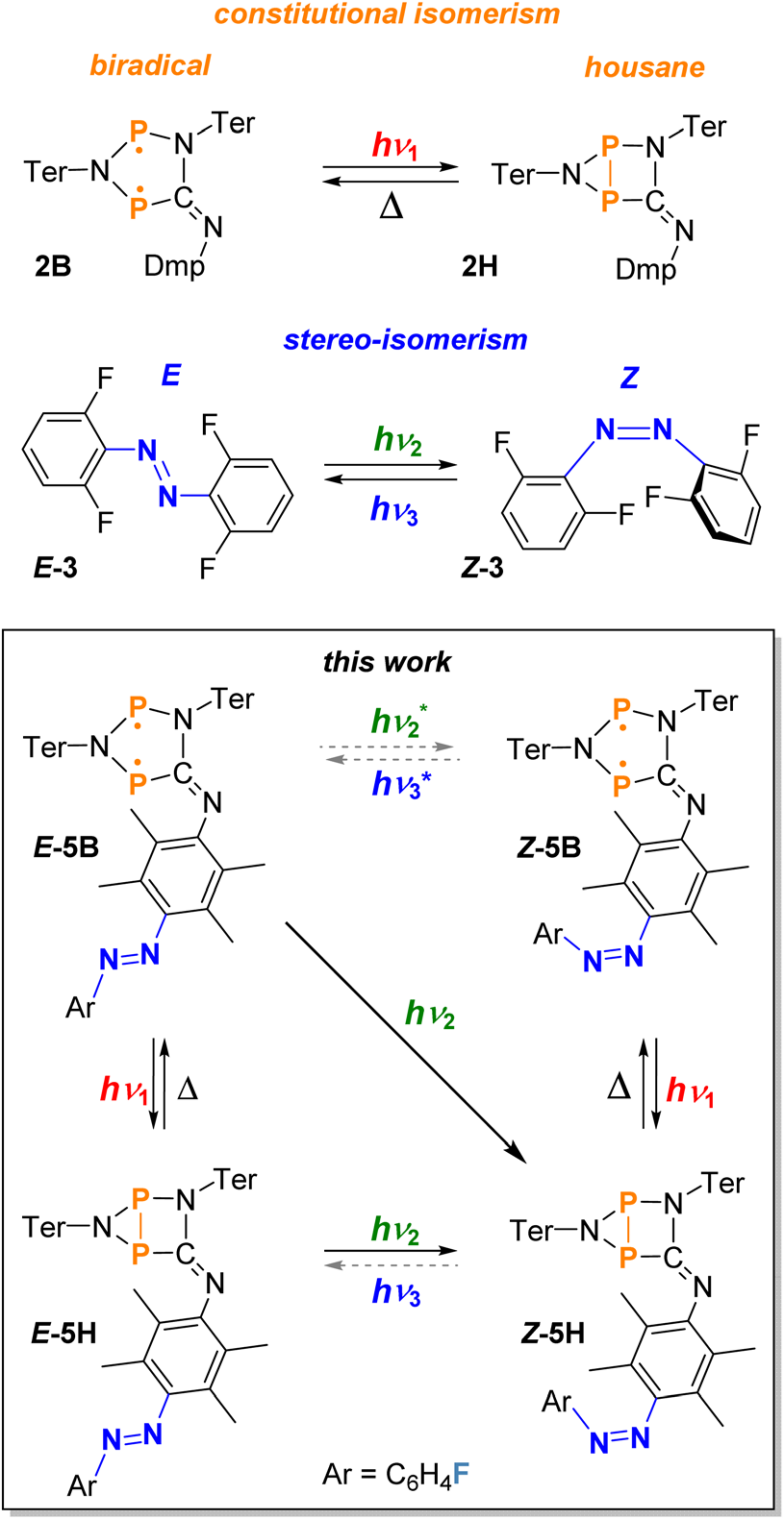
Examples of molecular switches undergoing either constitutional or stereo-isomerisation upon irradiation with visible light (top) and the combination of these types of switches to a novel visible light-mediated double photoswitch as part of this work (bottom). Reactions indicated by asterisks are presumed to take place under irradiation but cannot be traced spectroscopically as always housane formation occurs.^35,49^

Also, the use of functionalised azobenzene based photoswitches in the area of biomedicine has received a lot of attention lately.^28–33^ The convenient synthesis of azobenzene compounds, their stability towards air and moisture and possibilities for further modification of the parent azobenzene to enhance the switching properties has made these types of compounds extremely popular. However, for biomedical applications the use of visible light-mediated azobenzene photoswitches is essential, as UV-light can cause damage to biological tissue and penetrates poorly.^28–33^ In general the selective switching between *E*- and *Z*-isomers of the parent azobenzene with light of the visible part of the electromagnetic spectrum is not possible due to the heavily overlapping absorption bands for the *n* → π* transition of both isomers.^4,34–36^ In recent years, this problem was solved by modifying the azobenzene parent system, *e.g.*, fluorine atoms were introduced at the phenyl ring systems to separate the absorption maxima of the *n* → π* bands further (*E*-3 and *Z*-3, Scheme 1). With this method, differences of about 40 nm between the absorption maxima of the *E*-3 and *Z*-3 species can be achieved. Therefore, the isomerisation of *E* to *Z* can be performed with green light (>500 nm) and the reverse reaction (*Z* to *E*) with blue light (<470 nm, Scheme 1).^34–36^

Another class of molecular switches are photoswitchable biradicals, which are also called biradicaloids, if there are significant interactions between the two radical sites. Some of these systems have been intensively studied in the past and were applied for example in molecular electronics or non-linear optics.^37–46^ We recently introduced and studied a class of photoswitchable phosphorus-centred, heterocyclic biradicals (cyclopentane-1,3-diyl analogues) that can undergo constitutional isomerisation upon irradiation with red light (*ca.* 640 nm, *e.g.*, 2B and 2H, Scheme 1).^47–52^ In this particular case, the stable five-membered cyclic biradicals with radical centres at two phosphorus atoms can form a transannular P–P covalent single bond when irradiated, giving rise to the corresponding bicyclic housane species (bicyclo[2.1.0]pentane analogue). The P–P bond formation can also be thermally reversed in the dark, resulting in the recovery of the five-membered biradical. The Ter- and Dmp-substituted compound 2B (Ter = 2,6-dimesitylphenyl and Dmp = 2,6-dimethylphenyl) is one of the most stable representatives of this type of air and moisture sensitive biradicals, with the sterically demanding terphenyl substituents preventing the dimerisation of the biradical. The reversible switching process to form 2H is depicted in Scheme 1.^47–52^

Here, we now demonstrate the combination of biradical and azobenzene units in the same molecule, resulting in a novel molecular double switch that can perform either a constitutional or stereo-isomerisation process depending on the frequency of the visible light irradiation.

## Results and discussion

### Design of the double switch

**Scheme 2:**
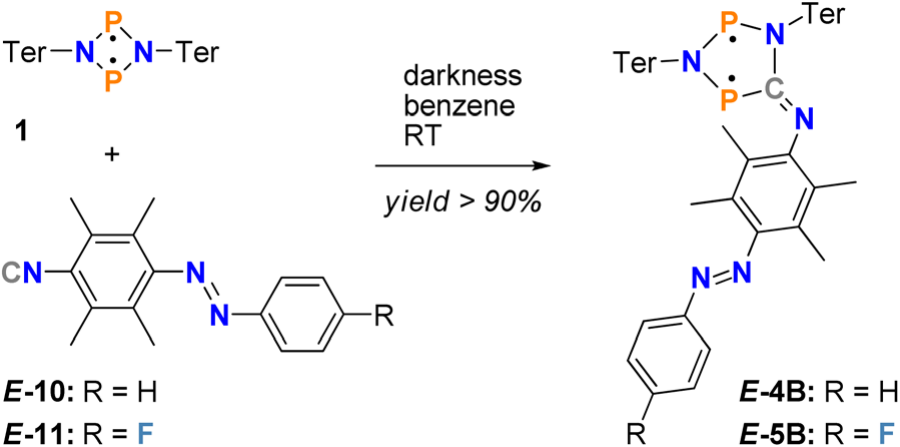
Synthesis of the double switches *E*-4B and *E*-5B with yields >90%.

Since our goal was to combine a biradical with an azobenzene switch, we first had to synthesise the corresponding azobenzenes with an isonitrile function (species *E*-10 and *E*-11, Scheme 2). However, the arylic 2- and 6-positions next to the isonitrile group had to be protected with methyl groups, which is essential to prevent the target compound, the five-membered biradicals *E*-4B and *E*-5B, from undergoing C–H activation at the 2- or 6-position of the phenyl ring system. This is a well-known process, resulting in the formation of benzo-azaphospholes.^53^ Moreover, it quickly became clear that a fluorine atom in para-position to the 2,6-dimethylphenyl substituent (species *E*-11), was advantageous as it provided another analytical tool, in addition to ^31^P{^1^H} NMR and UV/vis spectroscopy, to study the switching processes in detail using ^19^F{^1^H} NMR spectroscopy.

In a second step, starting from the isonitrile-functionalised azobenzene, the biradical double switches should be generated by insertion into a four-membered biradical [˙P(μ-NTer)_2_P˙] (1) (Scheme 2, species *E*-4B and *E*-5B). This is a well-known insertion reaction, which has been intensively investigated for the Dmp derivative 2B (Scheme 1).^49,50^ In addition, to achieve maximum steric shielding in the biradical switch, we chose to use the terphenyl substituent in the four-membered biradical 1.

Note: for better understanding, we labelled all biradical species with the letter B and all housanes with H. The orientation of the azobenzene unit is indicated by the according *E*- or *Z*-prefix. As we also observed different isomers (mostly rotamers) of each possible switching product in the experiments, these are labelled I^A^ and I^B^ (superscript letters for experimentally observed isomers) for better differentiation. The calculated isomers as part of the computational investigations, on the other hand, are labelled I^1^, I^2^, *etc.*, where superscript numbers are used for differentiation.

### Synthesis of the biradical-azobenzene-based double switch

The functionalised azobenzenes were synthesised according to modified literature procedures.^54,55^ At first a reaction of a methylated *p*-phenylenediamine with a (fluorinated) nitroso benzene produces the desired amino-azobenzenes *E*-6 and *E*-7. The following reactions to form the corresponding formamides *E*-8 and *E*-9 and subsequently the isonitriles *E*-10 and *E*-11 (Scheme 2) were carried out successfully. A detailed synthesis protocol for each compound (*E*-6 to *E*-11) can be found in the ESI† alongside the molecular structures of *E*-6, *E*-8 and *E*-10 in the single crystal (Fig. S1–S3†). The most prominent feature of all synthesised azobenzenes is the *E*-orientation in the solid state.

With pure *E*-isomer of azobenzenes *E*-10 and *E*-11 in hand, we now investigated the insertion reaction into 1 to form the five-membered biradicals attached to an azobenzene (Scheme 2). In fact, the reaction of *E*-10 or *E*-11 with biradical 1 in a 1 : 1 ratio in benzene forms the desired five-membered biradicals *E*-4B or *E*-5B within 15 minutes (Scheme 2). The formation of *E*-4B and *E*-5B is accompanied by a colour change from red, the colour of both starting materials, to an intense blue, which appears black in just slightly higher concentrations. In addition, the course of the reaction can be easily followed by ^31^P NMR spectroscopy (1: 276.4 ppm,^49^*E*-4B: 221.0/257.0 ppm, *E*-5B-I^A^: 219.7/257.5 ppm and *E*-5B-I^B^: 222.0/258.8 ppm at 202.5 MHz). Note: for *E*-5B, two sets of signals are observed for two different isomers of the compound (assigned to I^A^ and I^B^).

Interestingly, no *E* → *Z*-isomerisation is observed in the diazene scaffold during the insertion reaction, *i.e.*, when the reaction is carried out in the dark, starting from the *E*-azobenzene, only the *E*-isomer bound to the biradical form of the five-membered heterocycle is obtained. However, as soon as light is present, isomerisation occurs (see below). Therefore, the reaction must be carried out in the dark to avoid photoswitching. Moreover, since all reactants show extreme sensitivity to air and moisture, the reaction should be carried out in an argon-filled drybox. Both synthesised derivatives of the biradical double switch are dark blue, possess high melting points (*E*-4B: 203 °C, *E*-5B: 200 °C) and can be synthesised with excellent yields of over 90% (*E*-4B: 90%, *E*-5B: 92%). These compounds are stored in a sealed glass vial wrapped in aluminum foil in an argon-filled drybox at −20 °C in the dark, ensuring stability for several weeks.

### Structure elucidation

**Fig. 1:**
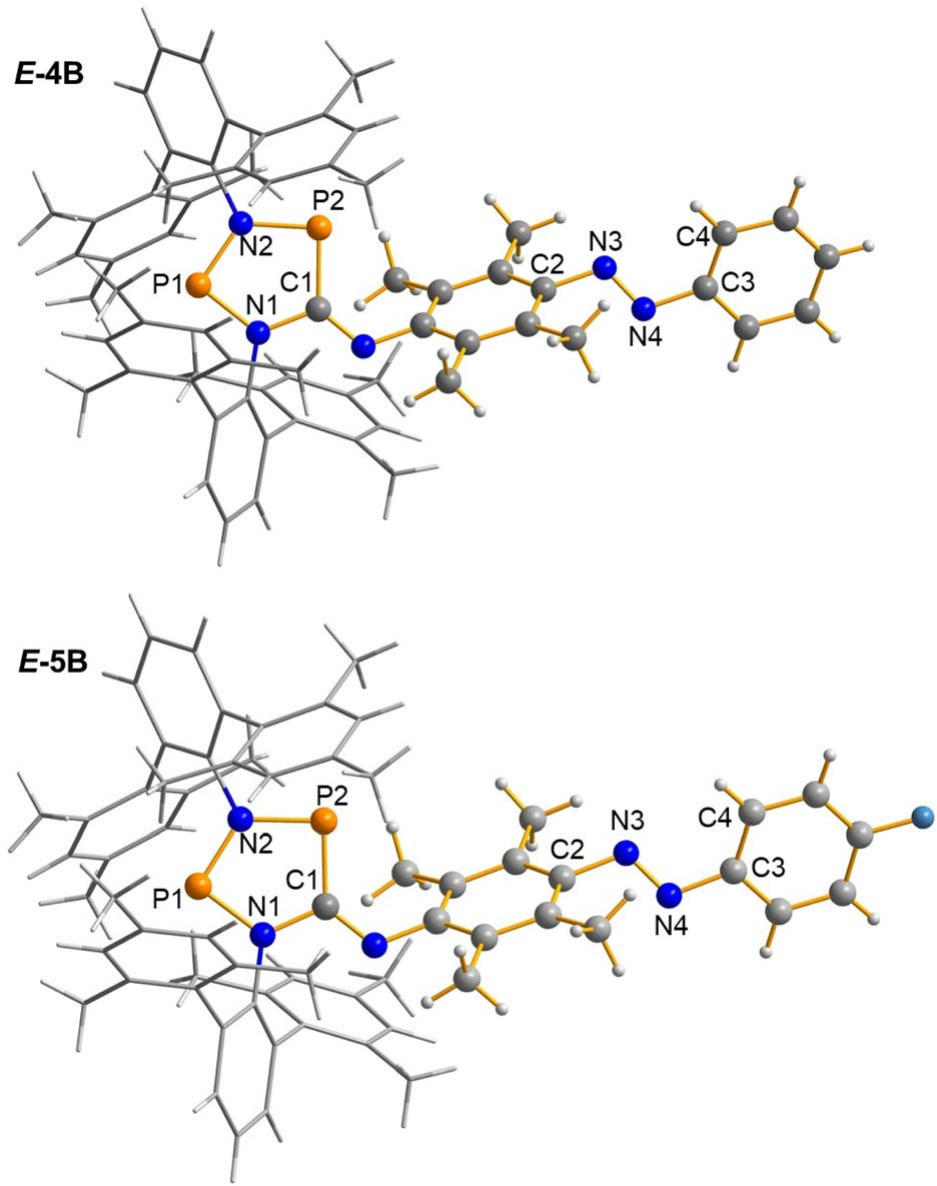
Ball-and-stick representations of the molecular structures of *E*-4B and *E*-5B in the single crystal (123 K). Terphenyls shown as wireframe. Colour code: grey = carbon; white = hydrogen; blue = nitrogen; orange = phosphorus; turquoise = fluorine. Selected bond lengths (Å) and angles (°): *E*-4B: P1⋯P2 2.9418(7); P1–N1 1.675(2); P1–N2 1.653(2); P2–N2 1.724(1); P2–C1 1.790(2); C1–N1 1.428(2); N3–N4 1.251(3); N2–P1–N1–C1 −0.5(1); C2–N3–N4–C3 −175.4(2); N3–N4–C3–C4 36.8(3); *E*-5B: P1⋯P2 2.945(2); P1–N1 1.678(3); P1–N2 1.642(4); P2–N2 1.737(3); P2–C1 1.773(3); C1–N1 1.441(5); N3–N4 1.258(4); N2–P1–N1–C1 0.8(3); C2–N3–N4–C3 174.7(3); N3–N4–C3–C4 −34.4(6). ORTEPs are shown in the ESI.†

Both *E*-4B and *E*-5B can be re-crystallised to obtain dark blue crystals suitable for single-crystal X-ray structural analysis. *E*-4B crystallises from a saturated benzene solution at 25 °C in the monoclinic space group *P*2_1_/*c* with four formula units per unit cell. The fluorinated species *E*-5B can be crystallised from a saturated *n*-hexane solution at 25 °C in the monoclinic space group *P*2_1_/*n* with four formula units per unit cell. The molecular structures of *E*-4B and *E*-5B are depicted in Fig. 1 along with selected structural parameters.

As in the Dmp analogue of the five-membered biradical (2B, Scheme 1), the terphenyl substituents in both *E*-4B and *E*-5B form a pocket containing the well-protected, planar five-membered heterocyclic ring system (*E*-4B: N2–P1–N1–C1 −0.5(1)°, *E*-5B: 0.8(3)°), clearly showing the presence of the biradical structures^49,50^ as indicated by a rather large transannular P⋯P distance with *d*(P1⋯P2) = 2.9418(7) Å for *E*-4B and *d*(P1⋯P2) = 2.945(2) Å for *E*-5B (*cf.* Σ*r*_cov_(P–P) = 2.22 Å).^56^ Housane formation can be ruled out, since in this case a short P–P distance of 2.220 Å (calculated) is expected.^49^ All P–N bonds are rather short (*E*-4B: *d*(P1–N2) = 1.653(2), *d*(P2–N2) = 1.724(1); *E*-5B: *d*(P1–N2) = 1.642(4), *d*(P2–N2) = 1.737(3) Å), indicating the presence of highly polarised P–N bonds with a small amount of double bond character (*cf.* Σ*r*_cov_(P–N) = 1.82 Å and Σ*r*_cov_(P=N) = 1.62 Å).^56^ The second structural motif of interest is the situation around the N=N double bond. In agreement with solution NMR data, only the *E*-isomer is found in the solid state (*E*-4B: C2–N3–N4–C3 −175.4(2)°, *E*-5B: 174.7(3)°). Again, the N=N double bonds are slightly elongated (*E*-4B: *d*(N3–N4) = 1.251(3) Å, *E*-5B: *d*(N3–N4) = 1.258(42) Å, *cf.* Σ*r*_cov_(N=N) = 1.20 Å),^56^ but still in the range of typical values for isonitrile-functionalised diazobenzenes, *e.g.*, 1.255 Å in *E*-10.

### Photoswitching

To investigate the switching capabilities of these new potential dual photoswitches, we performed a series of UV/vis as well as ^31^P{^1^H} and ^19^F{^1^H} NMR spectroscopic experiments in the dark and under irradiation with light of specific wavelengths (for the set-up of the device see ESI†).^49,57^ The experiments were carried out for both the fluorinated and non-fluorinated compounds at different temperatures. For reasons of clarity, only experiments at low temperature −40 (°C) and using the fluorinated compounds are discussed here. The data for the non-fluorinated compounds can be found in the ESI (Sections 5.1, 5.3 and 5.5†). The introduction of fluorine into the azobenzene moiety brings two major advantages that simplify the identification of the switching products by spectroscopic measurements. First, the electron density withdrawing fluorine atom changes the general electronic situation in *E*-11 and *E*-5B in such a way that isomerisation products can be more easily identified (*vide infra*). The second and more obvious advantage is, of course, that all switching processes can be additionally traced by ^19^F{^1^H} NMR spectroscopy.

#### UV/vis spectroscopic investigation of double photoswitch *E*-5B

**Fig. 2:**
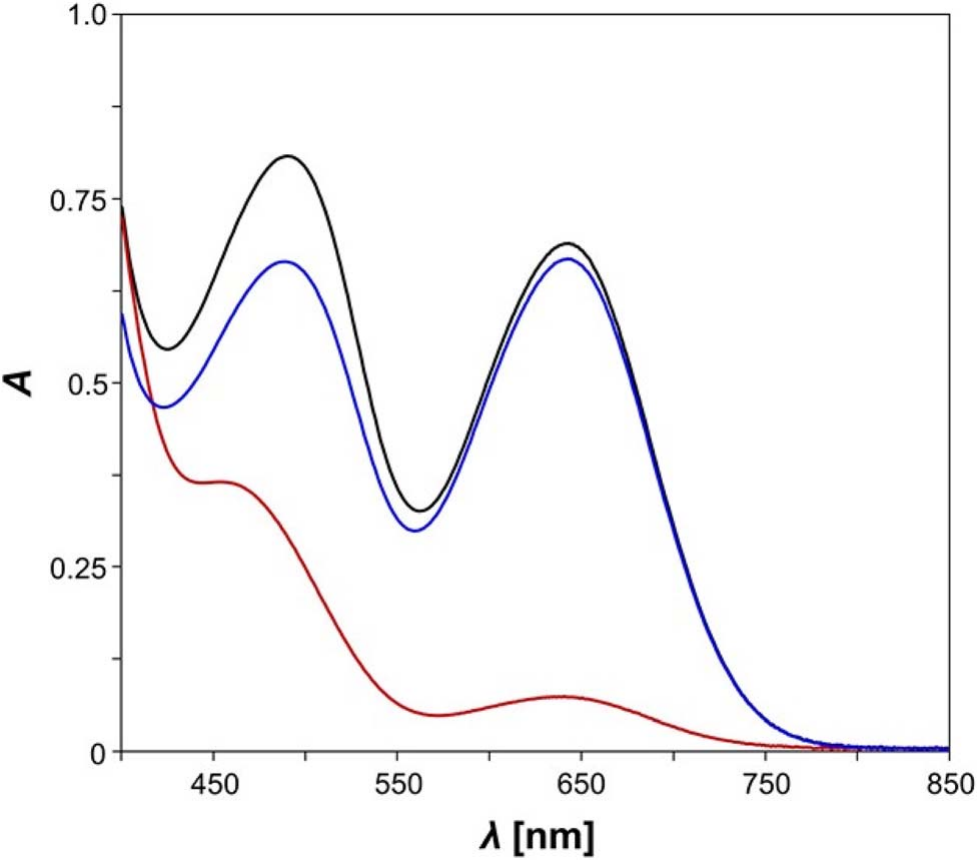
UV/vis spectra of *E*-5B before (black line), directly after irradiation with light (red line) and after 30 minutes in the dark, *i.e.*, after the thermal reverse reaction (blue line), *c* = 0.14 mmol L^−1^, benzene.

We studied double photoswitch *E*-5B by means of UV/vis spectroscopy under irradiation with light (Fig. 2). It should be noted that, when housanes are formed from the blue biradical upon irradiation, the broad band at around 650 nm associated with the presence of the biradical disappears, as a transannular P–P bond is present and housanes are colourless. This means that in such a case only the diazo switch is active. Its decisive UV/vis band is observed at approx. 450–470 nm. UV/vis spectroscopic measurements of the pure isonitrile-functionalised azobenzenes *E*-10 and *E*-11 were conducted as well, the according spectra are depicted in the ESI (Fig. S14 and S15†). It is also worth noting that an isomerisation at the C=N bond of the former isonitrile can be ruled out. According to computations, rotation around the C=N double bond has a high activation barrier due to the large steric hindrance in these compounds. Possible switching products of this isomerisation would feature the azobenzene unit sitting in between the bulky terphenyl substituents. The methyl groups of the azobenzene would overlap with the terphenyl systems, leading to a large Pauli repulsion, which in turn is energetically very unfavourable. Isomerisation at the C=N double bond could also not be observed in earlier experimental studies with the pure biradical switch 2B/2H.^49^

In our experiments, measurements of pure *E*-5B were conducted in the dark at first, revealing that *E*-5B exhibits two broad absorption maxima at wavelengths of 644 nm (biradical switch, calcd 633 nm) and 490 nm (diazo switch, calcd 519 nm, Fig. 2). It becomes evident, that the absorption maximum for the *n* → π* transition of the azobenzene moiety experiences a significant red shift of about 40 nm when compared to species *E*-11 (*cf.* 451 nm). However, irradiation of *E*-5B with red light at 644 nm should undoubtedly lead to housane formation (*E*-5H) as the absorption maximum is in the exact same range as the one for the Dmp monoswitch analogue 2B (*cf.* 643 nm, Scheme 1).^49^ Indeed, irradiation of the solution of *E*-5B with light leads to a significant change of the absorption maxima in the UV/vis spectrum (red line). The absorption band at 644 nm almost completely disappears indicating the formation of housane type species (*E*-5H, *Z*-5H). The absorption maximum for the *n* → π* transition of the azobenzene exhibits a significant blue shift and is now located around 454 nm with less intensity. As with the isonitrile *E*-11, it is assumed that a mixture of *E*- and *Z*-isomers is present after irradiation, however, the different absorption maxima for the *E*- and *Z*-species cannot be distinguished. For further details regarding the different transitions at 644 nm and 490 nm (*n* → π*) and a graphical representation of the corresponding natural transition orbitals (NTOs) for the most important states, please refer to the Computational studies.

When the mixture is left in the dark for a period of 30 minutes, the thermal reverse reaction takes place, as was found for the Dmp-substituted monoswitch analogue 2B/2H,^49^ and the corresponding biradical species *E*-5B and presumably *Z*-5B are formed. The UV/vis spectrum (blue line) now shows the characteristic absorption maximum at 644 nm for the biradical species again; the maximum for the *n* → π* band of the azobenzene experiences a red shift to 488 nm. A notable difference to the spectrum of pure *E*-5B (*cf.* 490 nm) is observed, probably caused by small amounts of *Z*-isomer (*Z*-5B) in the mixture. To summarise, a clear change in the UV/vis spectra of the analysed species can be seen upon irradiation and the biradical-housane switching is observed. However, a detailed discussion of the *E*/*Z* isomerisation process of the azobenzene is not possible because the main problem, the overlapping broad *n* → π* absorption bands of the *E*- and *Z*-isomers of the azobenzene unit, could not be solved. For this reason, variable temperature and time-controlled NMR spectroscopic investigations were carried out.

#### ^19^F{^1^H} NMR spectroscopic investigation of double photo-switch *E*-5B

**Fig. 3:**
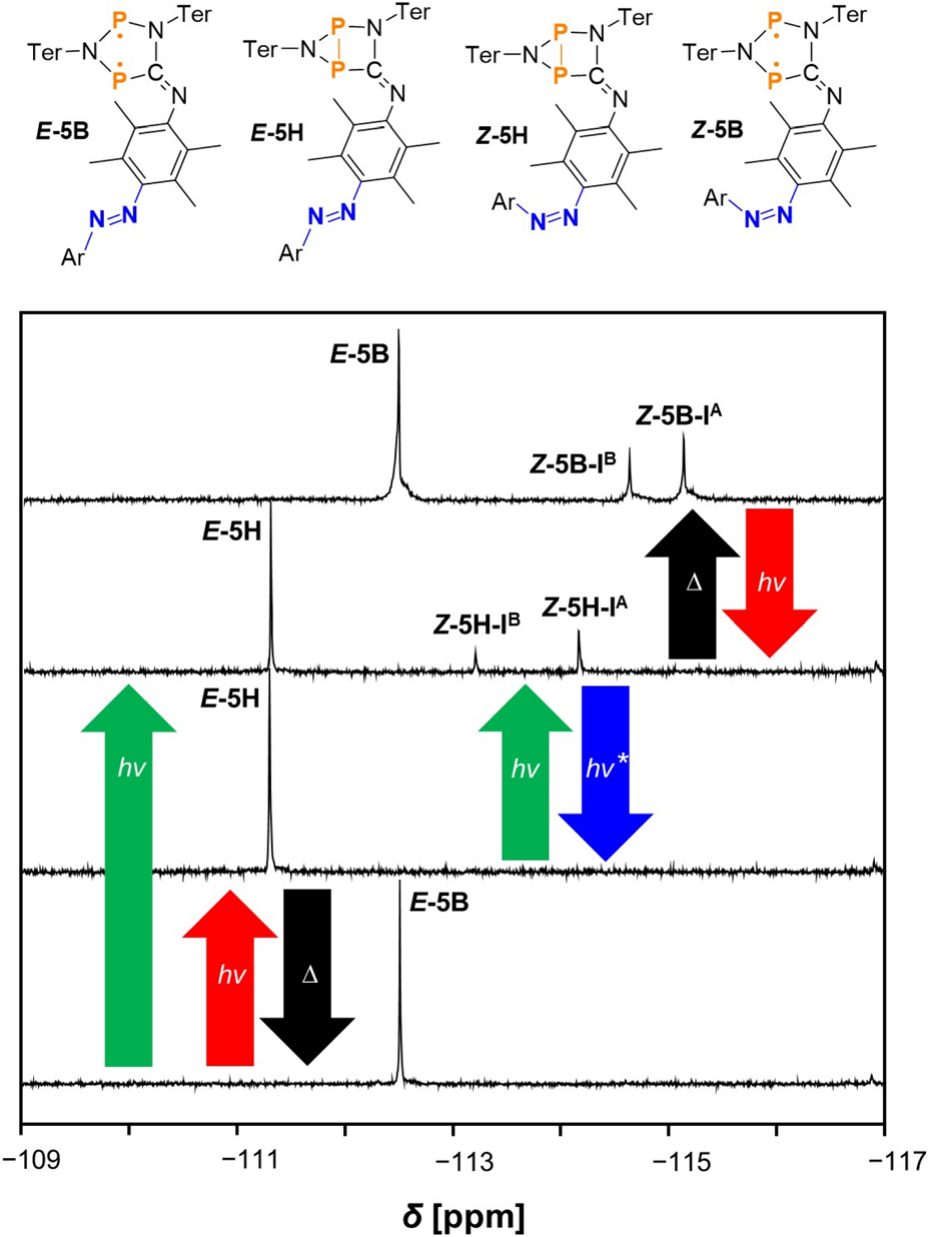
^19^F{^1^H} NMR spectra of *E*-5B at −40 °C (in THF-*d*_8_) before irradiation (bottom), after irradiation with red light (bottom middle), after irradiation with green light (top middle) and after heating the sample to room temperature (in the dark) with re-cooling (top). *The reverse reaction from *Z*-5H to *E*-5H under blue light occurs in a steady state with constant re-formation of *Z*-5H. Therefore, always traces of *Z*-5H are present in solution at this point. The spectrum (bottom middle) shows no signal of *Z*-5H as the experiment was conducted with a freshly prepared, isomer-pure solution of *E*-5H. For *Z*-5H and *Z*-5B signals for two different isomers can be observed assigned with I^A^ and I^B^.

Since UV/vis spectroscopy turned out to be unsuitable for unambiguous detection of the different switching products, we tried to induce the isomerisation process under irradiation in the NMR spectrometer. We started by investigating the pure isonitrile *E*-11 by using an already well-established experimental setup.^49,57^ The data are presented in the ESI (Fig. S17†) and show that switching between *E*- and *Z*-isomers of the azobenzene is possible depending on the irradiation conditions. To also get further spectroscopical evidence for the formation of switching products *E*-5H, *Z*-5H and *Z*-5B, we performed a series of irradiation experiments with double switch *E*-5B in the NMR spectrometer. ^19^F{^1^H} NMR spectra of a sample of *E*-5B in THF-*d*_8_ were recorded in the dark first, followed by irradiation with red light (638 nm), green light (520 nm) and blue light (445 nm). In the end, the thermal reverse reaction was carried out in the dark. As shown in Fig. 3, the switching processes of double switch *E*-5B can be carried out either stepwise or simultaneously.

The first ^19^F{^1^H} NMR spectrum of starting material *E*-5B recorded in the dark shows one singlet at −112.6 ppm at −40 (°C) as expected for pure *E*-5B. If irradiation with red light is turned on in the first part of the stepwise reaction, only the biradical switch responds and performs the formation of the housane type species *E*-5H (one singlet at −111.4 ppm). If the red light is switched off and irradiation is continued with green light, the *E*-to-*Z* isomerisation of the azobenzene is triggered and two new singlets at −114.3 ppm and −113.3 ppm can be observed in the ^19^F{^1^H} NMR spectrum, which can be assigned to two different isomers of compound *Z*-5H labelled as *Z*-5H-I^A^ and *Z*-5H-I^B^. A detailed discussion of the different isomers of each species is included in the Computational studies (*vide infra*). This stepwise switching process (irradiation with red light, then green light) can also be carried out simultaneously by irradiating starting material *E*-5B directly with green light, as both the housane formation as well as the *E*-to-*Z* isomerisation at the azobenzene occur at the same time (forming *Z*-5H) in this case. As already known from the investigations with isonitrile *E*-11, no complete conversion to the housane-*Z* species *Z*-5H can be achieved. With the mixture of *E*-5H and *Z*-5H in hand, the thermal reverse reaction can now take place in the dark after switching off the light. To this end, the sample was heated to room temperature, left in the dark for 15 minutes and subsequently re-cooled to −40 °C for better comparability of the spectra. Indeed, after the thermal reverse reaction took place only biradical species could be observed in the ^19^F{^1^H} NMR spectrum. As expected, *E*-5H reacts back to starting material *E*-5B and the housane *Z*-isomer *Z*-5H reacts to the biradical *Z*-species *Z*-5B in the process as indicated by the appearance of two new singlets at −114.9 ppm and −114.5 ppm (at the expense of the signals caused by compound *Z*-5H). Another irradiation cycle using red light then of course leads to repeated switching of the biradical unit, converting *E*-5B and *Z*-5B back to housane type species *E*-5H and *Z*-5H. This switching process demonstrates the ability of converting the double switch *E*-5B to the other three possible products *E*-5H, *Z*-5H and *Z*-5B, when performing the switching process at the biradical and azobenzene centres. Irradiation of the mixture of housane type species *E*-5H and *Z*-5H with blue light to induce *Z*-to-*E* isomerisation at the azobenzene moiety, yielding *E*-5H, represents a special case. ^19^F{^1^H} NMR spectra before and after irradiation with blue light show only a slight decrease of the amount of *Z*-5H, whereas significant amounts of *Z*-5H remain in solution next to *E*-5H (see Fig. S37†). As the switching process is undoubtedly possible (as demonstrated with *E*-11, see ESI†), it seems that a steady state within the reaction mixture is formed. The influence of blue light induces both the *Z*-to-*E*- as well as the *E*-to-*Z*-reactions at the same time leading to the constant presence of specific amounts of both *E*-5H and *Z*-5H in solution (*i.e.*, leading to no substantial change in the ^19^F{^1^H} NMR spectra). Note that in Fig. 3 the associated spectrum (bottom middle) shows no signal of *Z*-5H as the experiment was conducted with a freshly prepared, isomer-pure solution of *E*-5H.

#### ^31^P{^1^H} NMR spectroscopic investigation of double photo-switch *E*-5B

**Fig. 4:**
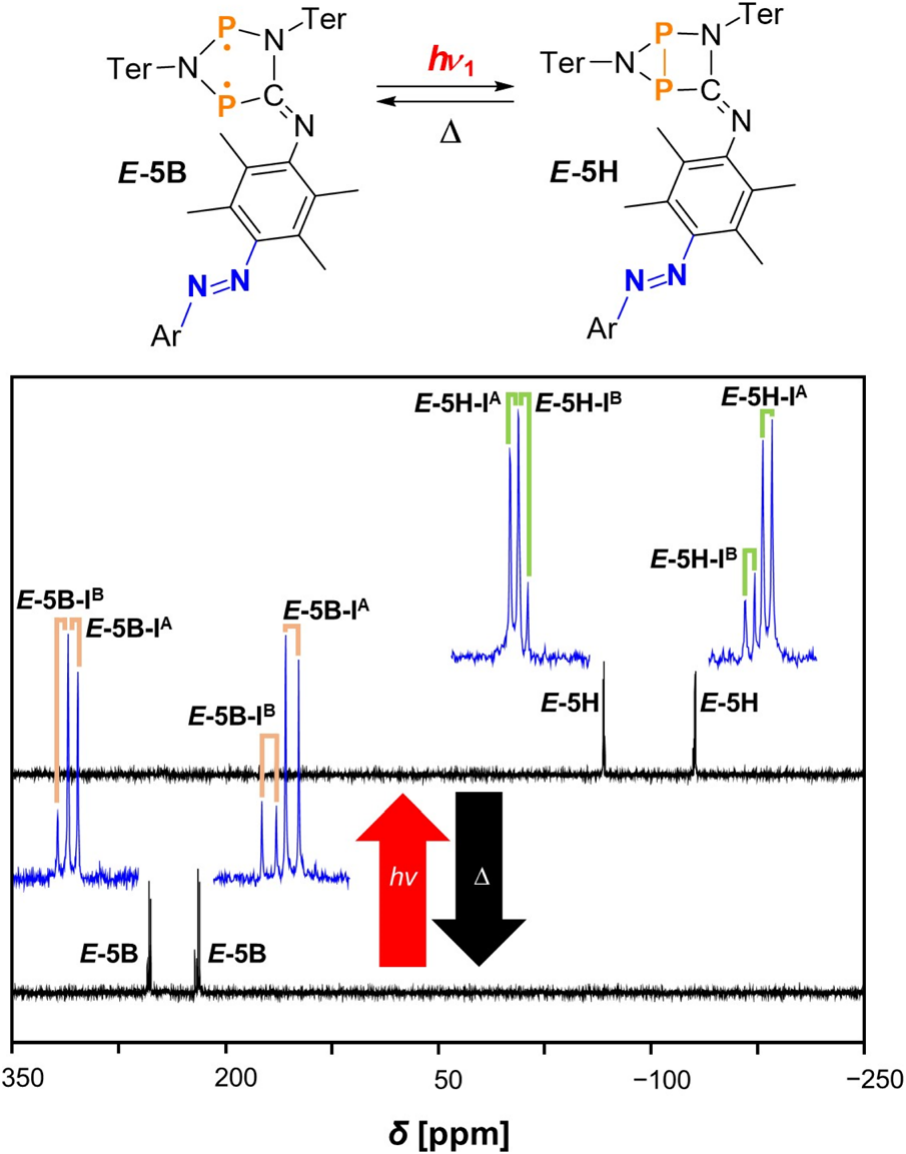
^31^P{^1^H} NMR spectra of the switching process of *E*-5B to *E*-5H at −40 °C (in THF-*d*_8_) under irradiation with red light. Enlarged signals are depicted in blue for better recognisability of the coupling pattern. The doublet resonances for *E*-5B are indicated by orange labels and for *E*-5H by green labels.

With the knowledge obtained from the discussed ^19^F{^1^H} NMR spectra, we now focused on ^31^P{^1^H} NMR spectroscopy as an additional method to verify the observed behaviour of the double switch. We were especially driven by the question, whether the switching between *E*- and *Z*-isomers at the azobenzene sufficiently alters the electronic situation of the switch in order to distinguish signals of the different isomers in the ^31^P{^1^H} NMR spectra. The irradiation process was conducted in the same way as described above for the recorded ^19^F{^1^H} NMR spectra, starting with measurements of pure *E*-5B in the dark at −40 °C followed by irradiation with red, green, and blue light. Finally, the thermal reverse reaction was studied again. As the signals for the different species occasionally overlap, the spectra are depicted in separate figures and the signals are assigned with different colours for better understanding. The spectrum of the double switch *E*-5B (pure starting material) shows a total of four doublets resonances in the dark at −40 °C. However, since only two signals are expected for the two inequivalent phosphorus atoms, this means that two different isomers of double switch *E*-5B are present in solution (assigned with I^A^ and I^B^), as can be seen in the lower spectrum in Fig. 4. Interestingly, these two isomers *E*-5B-I^A^ and *E*-5B-I^B^ are distinguishable in the ^31^P{^1^H} NMR spectrum but not in the ^19^F{^1^H} NMR spectrum (one broad singlet at −112.6 ppm, *vide supra*). A detailed discussion on the nature of these isomers I^A^ and I^B^ may be found in the section on Computational studies, a clear identification of the doublet signals can be made based on the *J*(^31^P,^31^P) coupling constants. In detail, *E*-5B-I^A^ shows doublet resonances at 219.2 ppm with a coupling constant of ^2^*J*(^31^P,^31^P) = 120 Hz for the N*P*C phosphorus atom and at 253.8 ppm with a coupling constant of ^2^*J*(^31^P,^31^P) = 120 Hz for the N*P*N phosphorus atom. In addition, doublet resonances are also observed for isomer *E*-5B-I^B^, however, at 221.3 ppm with ^2^*J*(^31^P,^31^P) = 130 Hz for the N*P*C and 255.1 ppm with ^2^*J*(^31^P,^31^P) = 130 Hz for the N*P*N phosphorus atom (Table 1).

**Table 1:** Experimental ^31^P{^1^H} NMR shifts [ppm] and coupling constants *J* [Hz] for all observed isomers of *E*-5B, *E*-5H, *Z*-5H and *Z*-5B at −40 °C. The sign of *J* was determined by computations

	*δ*_exp_ N*P*N	*δ*_exp_ N*P*C	*J*_exp_
*E*-5B-I^A^	253.8	219.2	120
*E*-5B-I^B^	255.1	221.3	130
*E*-5H-I^A^	−67.1	−131.9	−62
*E*-5H-I^B^	−67.7	−130.8	−62
*Z*-5H-I^A^	−67.2	−132.5	−62
*Z*-5H-I^B^	−55.9	−129.2	−62
*Z*-5B-I^A^	253.3	219.5	120
*Z*-5B-I^B^	255.2	221.5	130

In the following stepwise irradiation process, compound *E*-5H can be generated again by irradiating the sample solution with red light, leading to the appearance of four new doublet resonances which can be assigned to two different isomers (I^A^ and I^B^) of housane *E*-5H. These isomers show significantly different chemical shifts compared to *E*-5B as can be seen in Table 1. In general, it can be said that the biradical species (here *E*-5B) show doublet resonances at around 210 to 260 ppm and the housane type species (here *E*-5H) at around −50 to −150 ppm. The spectra of this switching process are depicted in Fig. 4.

**Fig. 5:**
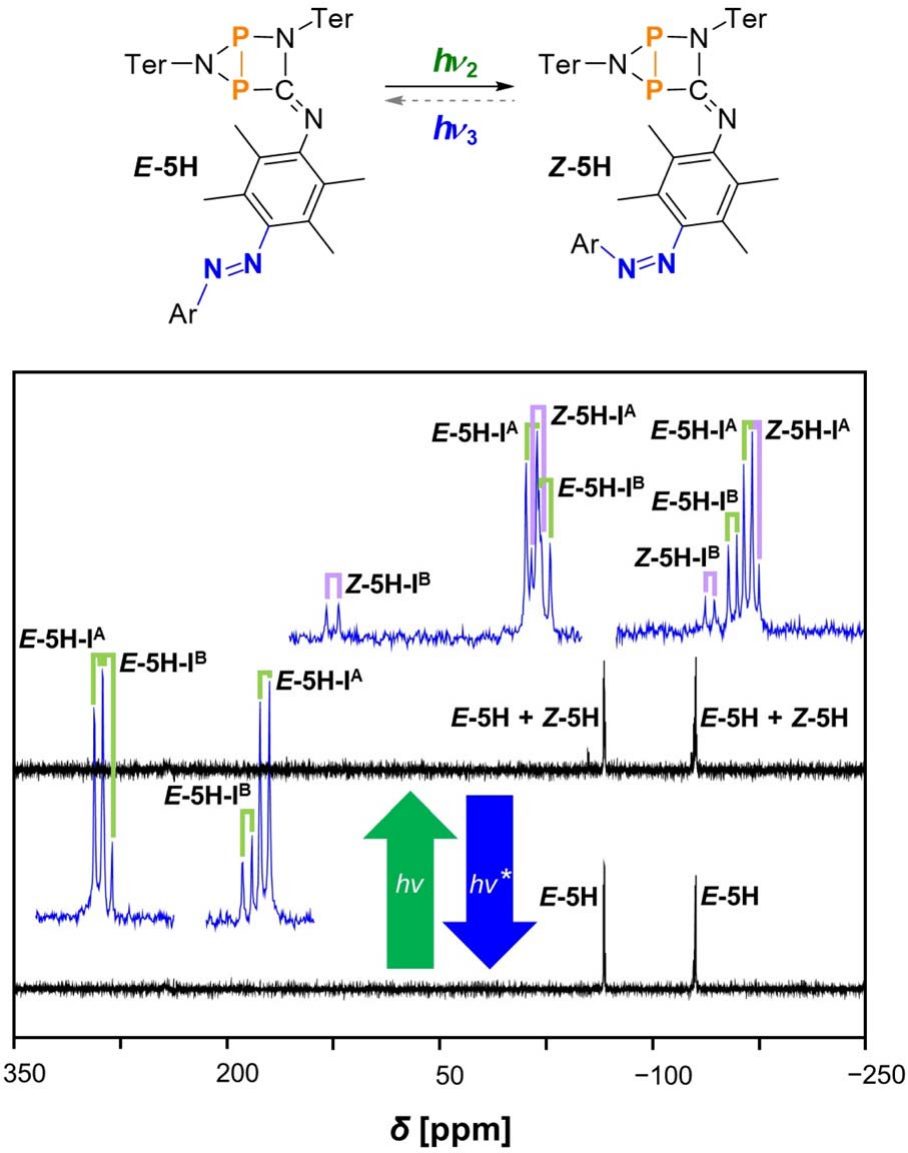
^31^P{^1^H} NMR spectra of the switching process from *E*-5H to *Z*-5H at −40 °C (in THF-*d*_8_) under irradiation with green light. *The reverse reaction from *Z*-5H to *E*-5H under blue light leads to a steady state with constant re-formation of *Z*-5H. Therefore, always traces of *Z*-5H are present in solution at this point. The spectrum (bottom) shows no signal of *Z*-5H as the experiment was conducted with a freshly prepared, isomer-pure solution of *E*-5H. Enlarged signals are depicted in blue for better recognisability of the coupling pattern. The doublet resonances for *E*-5H are indicated by green labels and for *Z*-5H by purple labels.

If the sample is now irradiated with green light in the second part of the stepwise reaction, the *E*-to-*Z* isomerisation at the azobenzene is partially triggered again, resulting in the appearance of four new doublets that can be assigned to switching product *Z*-5H (again formation of two different isomers I^A^ and I^B^), while compound *E*-5H is still present in solution. Again, the signals of *Z*-5H can be assigned using the ^1^*J*(^31^P,^31^P) coupling constants and show slightly different chemical shifts compared to *E*-5H (Table 1). The spectra recorded during this switching process can be seen in Fig. 5. Of course, the stepwise irradiation with red light at first, followed by green light can be carried out simultaneously again by irradiating starting material *E*-5B directly with green light, affording the mixture of *E*-5H and *Z*-5H.

Irradiation of a mixture of *E*-5H and *Z*-5H with blue light to induce the *Z*-to-*E* reverse reaction at the azobenzene again just leads to a slight decrease of the amount of *Z*-5H in the reaction mixture; significant amounts of *Z*-5H remain next to *E*-5H. The influence of blue light again induces both the *Z*-to-*E* as well as the *E*-to-*Z* isomerisations at the same time, leading to the formation of a steady state with a constant presence of specific amounts of both *E*-5H and *Z*-5H in solution. Note that in Fig. 5 the associated spectrum (bottom) shows no signal of *Z*-5H as the experiment was conducted with a freshly prepared, isomer-pure solution of *E*-5H (*cf.*^19^F{^1^H} NMR spectra).

**Fig. 6:**
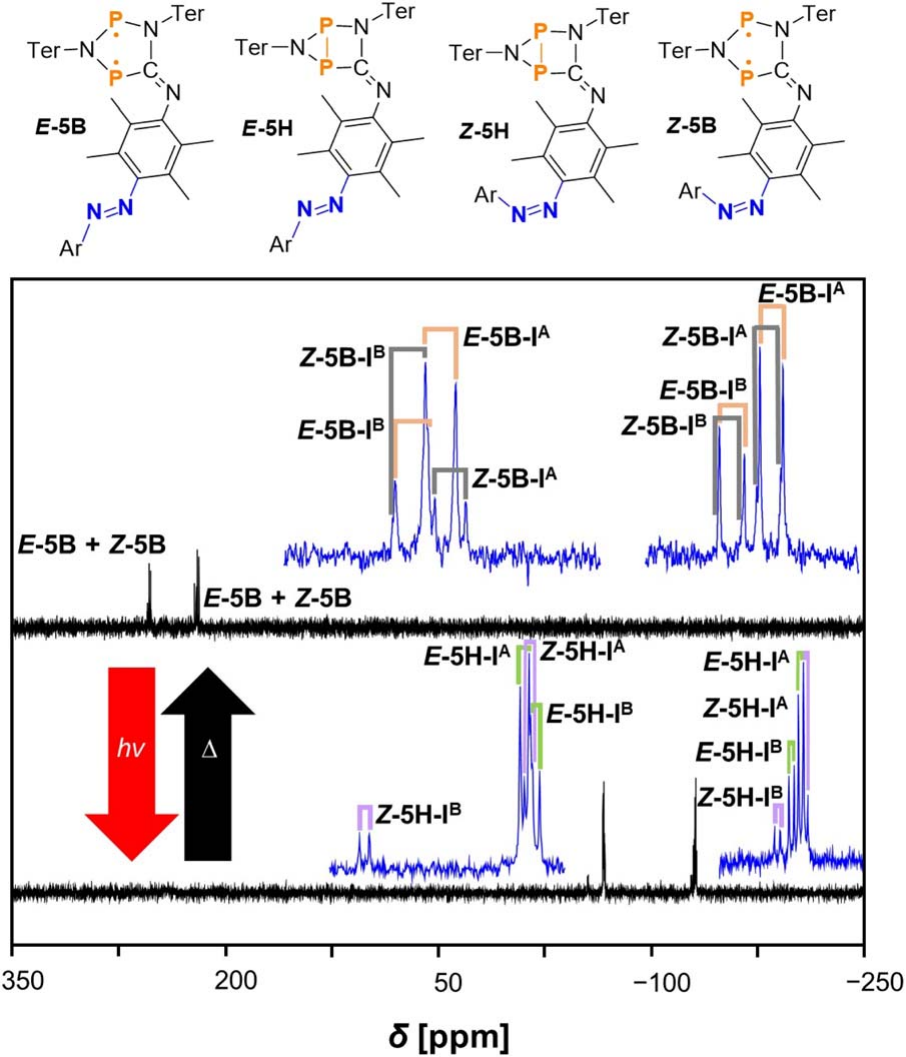
^31^P{^1^H} NMR spectra of the switching process from *Z*-5H to *Z*-5B and *E*-5H to *E*-5B at −40 °C (in THF-*d*_8_) after performing the thermal reverse reaction in the dark at room temperature. Enlarged signals are depicted in blue for better recognisability of the coupling pattern. The doublet resonances for *E*-5B are indicated by orange labels, for *E*-5H by green labels, for *Z*-5H by purple labels and for *Z*-5B by grey labels.

The biradical species *Z*-5B can be generated in the same manner as described above (*vide supra*). The thermal reverse reaction in the dark leads to formation of the starting material *E*-5B from *E*-5H and the newly formed *Z*-5B from *Z*-5H. The corresponding ^31^P{^1^H} NMR spectrum displays eight doublet resonances in total: four resonances attributed to I^A^ and I^B^ of starting material *E*-5B and four doublet resonances for two isomers (I^A^ and I^B^) of newly formed compound *Z*-5B. The chemical shifts and coupling constants of compound *Z*-5B are listed in Table 1 and differ slightly compared to those of *E*-5B. The spectra recorded before and after the thermal reverse reaction are depicted in Fig. 6. As already discussed earlier, subsequent irradiation of the mixture of *E*-5B and *Z*-5B with red light leads to re-formation of the housane type species *E*-5H and *Z*-5H.

**Fig. 7:**
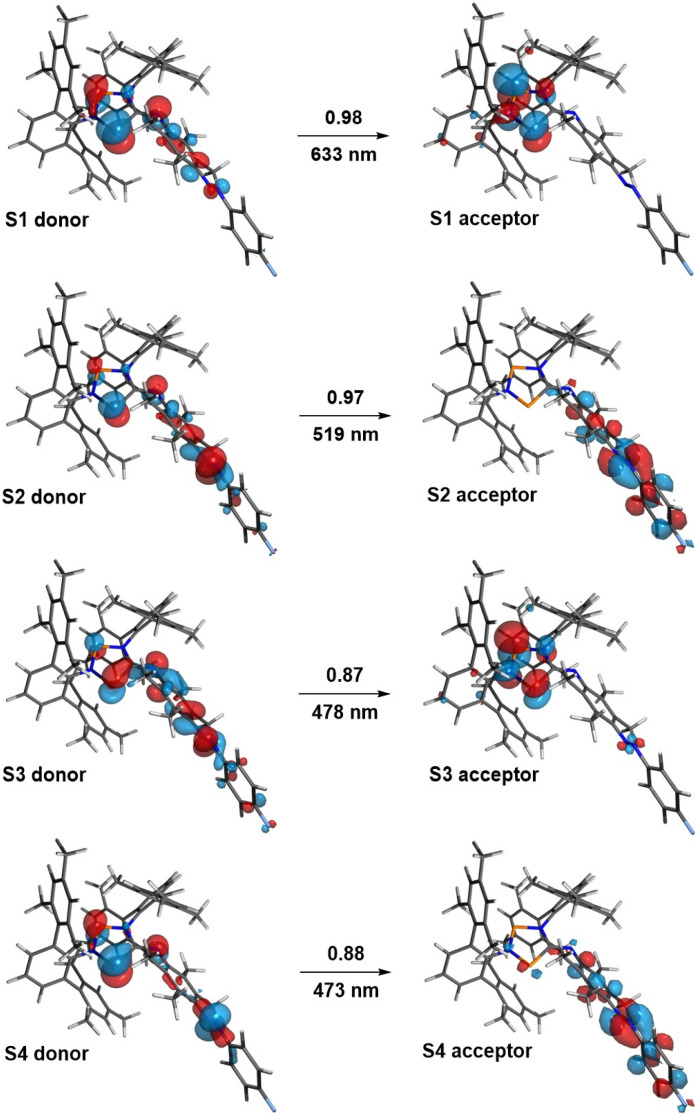
Natural transition orbitals (NTOs) of *E*-5B. States S1 to S4 (with contributing coefficients) are depicted alongside the corresponding wavelengths.

In summary, these experiments have provided a detailed insight into the complex character of the newly designed visible light-mediated double photoswitch *E*-5B. Furthermore, it was shown that there are interesting possibilities to influence the specific isomerisation processes at the biradical and azobenzene moieties depending on the irradiation conditions. The biradical switch performs well with a full conversion to the housane-type species upon irradiation, while the quantum yield is estimated to be similar to that of the Dmp-analogue 2B (24.6(8)%).^49^ The *Z*-isomers of the azobenzene switch (*Z*-5B and *Z*-5H) can be generated with a yield of 32% in the PSS, which is somewhat less efficient than already established azobenzene monoswitches (∼80%).^35,36^ One reason for this is the strong influence of the attached biradical unit in our conjugated double photoswitch (see Computational details, Fig. 7) since the pure azobenzene *E*-11 can be switched to *Z*-11 with a yield of about 56% (ESI, Fig. S17†). Parent compound *E*-5B shows a very high thermal stability (up to 200 °C), the housane type species *E*-5H and *Z*-5H, however, need to be handled either at low temperatures (−40 °C) or under constant irradiation with light (*e.g.*, 638 nm) as otherwise the backward reaction to the biradical species takes place. Species *Z*-5B is thermally stable for several hours since a slow reaction to *E*-5B seems to progress at room temperature (also see Computational details). Finally, a short comment on the fatigue resistance: The switching processes of the biradical switch (as shown in Scheme 1) can be carried out numerous times without decomposition of the switch and the switching capabilities are still present after several months if *E*-5B is stored properly. Switching of the azobenzene isomers is also possible with a large number of irradiation cycles as observed with the azobenzene monoswitch *E*-11 (Fig. S17†). However, the azobenzene switch needs to be improved further in the future to perform *Z*-5H to *E*-5H isomerisation with satisfying yields under irradiation with blue light for continuously carrying out numerous irradiation cycles.

**Table 2:** Number of computed isomers for *E*-5 and *Z*-5

	*E*-5B	*E*-5H	*Z*-5H	*Z*-5B
#(CREST)^a^	75	54	96	73
#(CENSO)^b^	63	20	22	49
#(discussed)^c^	6	4	3	7

a Number of isomers identified by CREST.

b Number of isomers remaining after optimization using CENSO.

c Number of discussed isomers which represent the energetically lowest lying isomers.

### Computational studies

At first, we performed calculations to compare our experimentally observed UV/vis absorption maxima to calculated values. The corresponding natural transition orbitals (NTOs) of biradical *E*-5B are shown in Fig. 7 (see also ESI Section 6.3† for further details). In general, the computed transitions are in good agreement with experimentally observed UV/vis absorption bands.

For example, the transition for the S1 state, experimentally observed as broad absorption maximum at 644 nm for the housane formation (Fig. 2), corresponds well with a calculated wavelength of 633 nm. The S2 state can be regarded as an *n* → π* transition with the NTOs located mostly at the azobenzene fragment. This transition comes along with a calculated wavelength of 519 nm (*cf.* Experimental value: 490 nm). It also becomes evident, that the shown NTOs of the donor states are delocalized across the biradical and azobenzene fragments indicating electronic conjugation along these molecular entities. This also explains the red shift of the absorption maximum for the *n* → π* transition of *E*-5B (490 nm) compared to *E*-11 (451 nm).

**Fig. 8:**
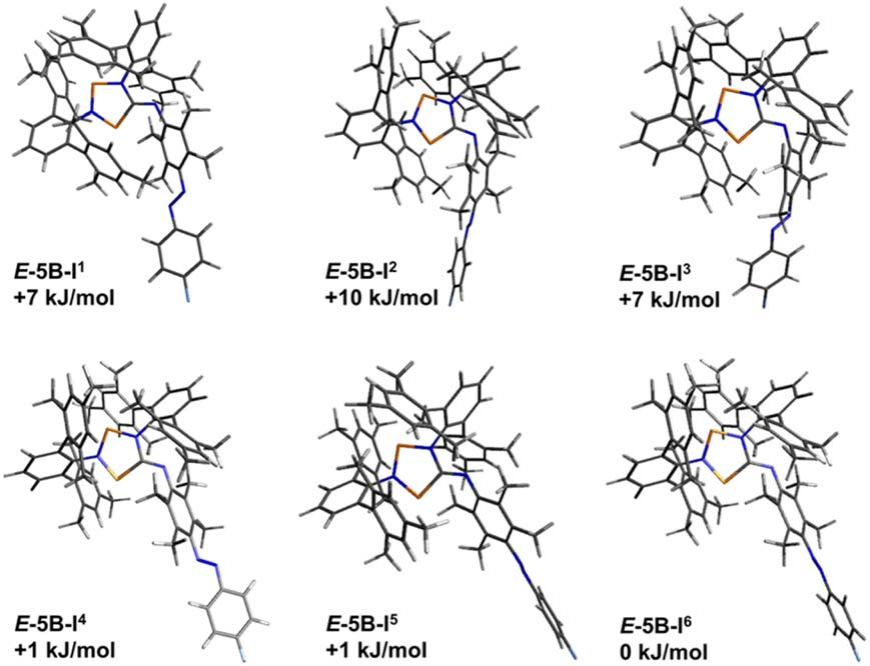
Graphical representation of the significant calculated isomers of *E*-5B, which were identified as the six lowest lying isomers (standard Gibbs free energies in relation to the lowest energy isomer).

**Fig. 9:**
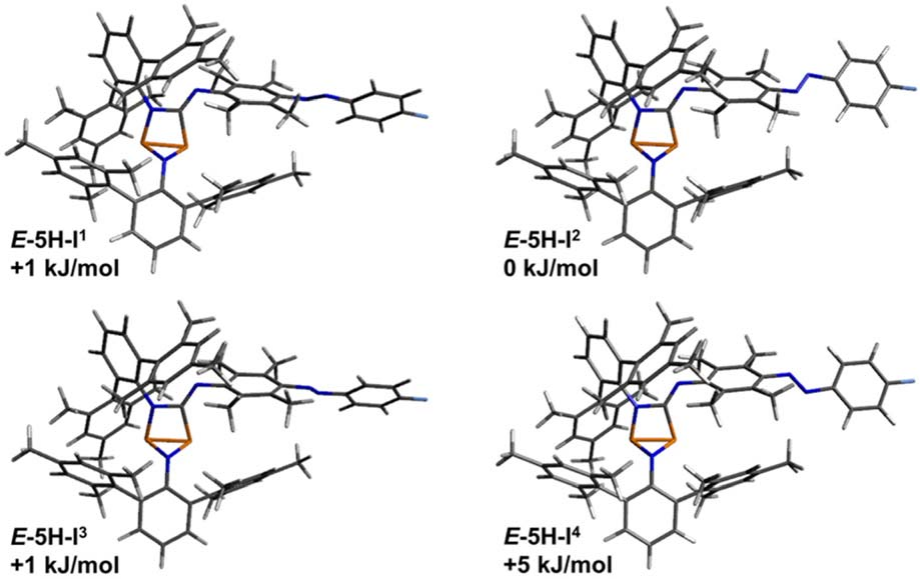
Graphical representation of the significant calculated isomers of *E*-5H, which were identified as the four lowest lying isomers (standard Gibbs free energies in relation to the lowest energy isomer).

**Fig. 10:**
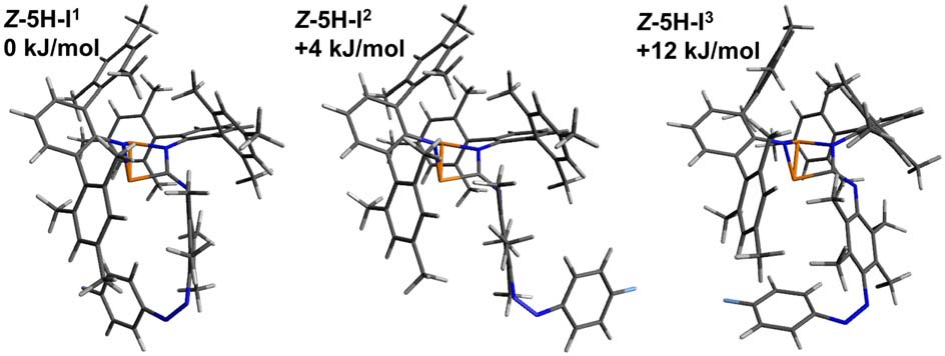
Graphical representation of the significant calculated isomers of *Z*-5H, which were identified as the three lowest lying isomers (standard Gibbs free energies in relation to the lowest energy isomer).

**Fig. 11:**
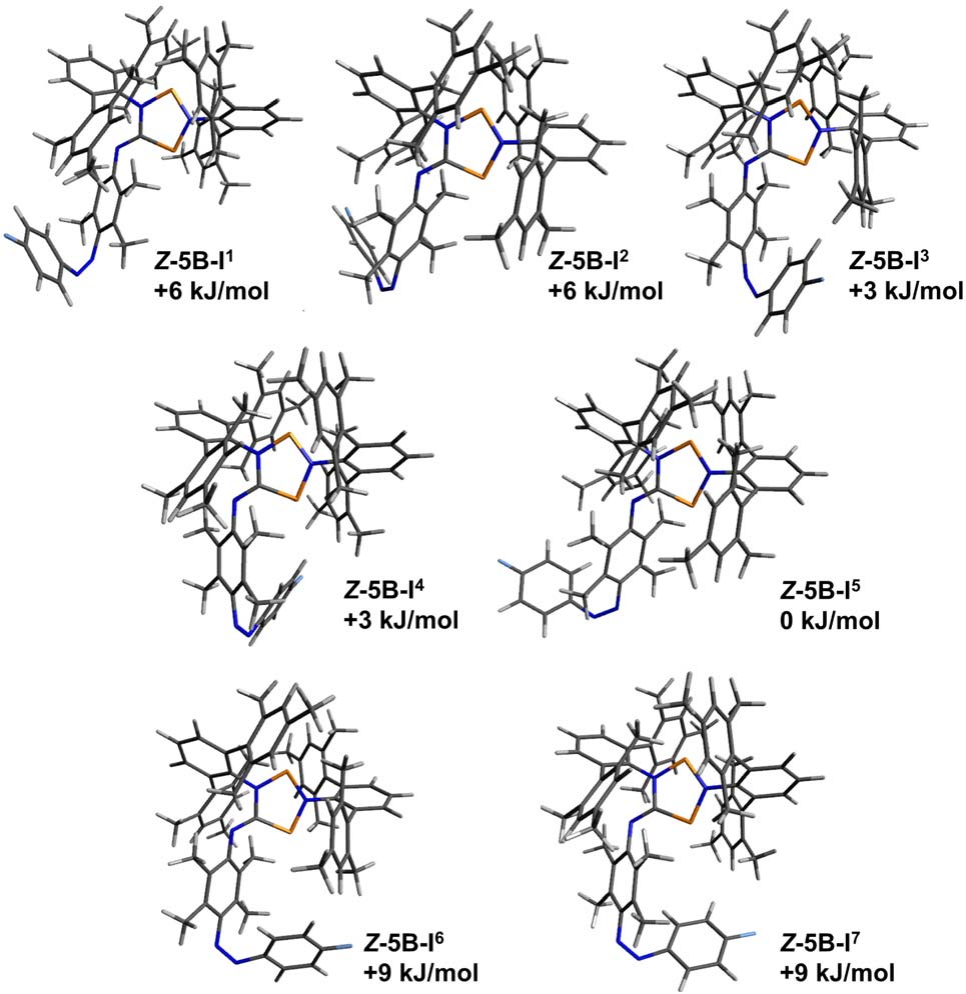
Graphical representation of the significant calculated isomers of *Z*-5B, which were identified as the seven lowest lying isomers (standard Gibbs free energies in relation to the lowest energy isomers).

As we encountered a variety of different isomers for the different switching products *E*-5B, *E*-5H, *Z*-5H and *Z*-5B as described above, we conducted an isomer search employing the xTB software (GFN2-xTB level of theory)^58,59^ as well as the CREST computer code^60,61^ and CENSO algorithm^62^ to better understand the complex three-dimensional structure of the double switch. All switching products *E*-5B, *E*-5H, *Z*-5H and *Z*-5B were investigated and the identified isomers were further optimized followed by calculation of ^19^F and ^31^P NMR shifts according to the methods describes in the ESI.† A full set of data of the calculated isomers can also be found in the ESI.† Since many different isomers for compound 5 were found in the various theoretical calculations (Table 2 and Fig. 8–11), we have limited our discussion to a few, namely the most thermodynamically stable isomers.

As shown in Fig. 8–11, the structural differences between the various isomers are very small, mostly due to rotations in the backbone. The energy differences are also very small (only isomers within 12 kJ mol^−1^ are shown). In the following, we would like to briefly discuss the structural differences of the three isomers of *Z*-5H as examples (Fig. 10). Computationally, for housane *Z*-5H a variety of different isomers was found of which the isomers I^1^, I^2^ and I^3^ represent the lowest energy isomers as depicted in Fig. 10. These three isomers differ by less than 12 kJ mol^−1^, which again underlines that these isomers might be observed at ambient temperature, which is consistent with the NMR experiments where we observed signals for two different isomers of *Z*-5H.

Although the energetic differences between these three isomers of switching product *Z*-5H are relatively small, the structural parameters show clear differences. For example, while in Z-5H-I^1^ the terminal phenyl ring of the azobenzene moiety takes an orientation pointing to the left side of the molecule in Z-5H-I^2^ the same phenyl ring is orientated into the opposite direction pointing to the right side of the molecule. And while in Z-5H-I^3^ the orientation of the terminal phenyl ring matches the one in Z-5H-I^1^ the phenyl ring itself is twisted by roughly 90° as opposed to the situation in Z-5H-I^1^.

Similar structural differences, which mainly indicate rotamers, are found in the species *E*-5B, *E*-5H, *Z*-5B (Fig. 8–11). These structural differences in the isomers of *Z*-5H also come along with differences in the calculated ^19^F NMR shifts of up to 10 ppm between the isomers, explaining why multiple signals appear in the corresponding ^19^F{^1^H} NMR spectrum for compound *Z*-5H. A summary of all calculated ^19^F NMR shifts (298 K) for the different isomers are listed in Table 3.

**Table 3:** Calculated ^19^F NMR shifts [ppm] for selected isomers of *E*-5B, *E*-5H, *Z*-5H and *Z*-5B (PBE0-D3/def2SVP level of theory,^63–69^ see ESI for further details on calculations)

	*E*-5B	*E*-5H	*Z*-5H	*Z*-5B
I^1^	−98.4	−96.2	−99.2	−104.8
I^2^	−98.3	−96.1	−96.1	−104.8
I^3^	−98.4	−95.9	−105.9	−109.1
I^4^	−98.9	−95.7		−109.1
I^5^	−98.8			−100.7
I^6^	−98.8			−106.4
I^7^				−106.4

For the biradical-*Z*-isomer *Z*-5B, the calculated ^19^F NMR shifts of the possible isomers also show differences of up to 10 ppm. The calculated ^19^F NMR shifts for the *E*-azobenzene species *E*-5B and *E*-5H almost show no difference at all and are in the same range of −98 ppm for starting material *E*-5B and −96 ppm for switching product *E*-5H. This also explains why in the ^19^F{^1^H} NMR spectrum only one singlet can be observed for compounds containing the *E*-azobenzene isomer, although different isomers of the species are present in solution as proven by the recorded ^31^P{^1^H} NMR spectra. The ^31^P NMR shifts of all discussed isomers were calculated as well and are in good agreement with our experimental findings (see ESI† for further information). In contrast to the calculated ^19^F shifts for isomers of *E*-5B and *E*-5H, where the different isomers are indistinguishable, the calculated ^31^P NMR shifts of the same isomers show significant differences of up to 7 ppm, in agreement with the experimental observation that the different isomers are distinguishable in the corresponding ^31^P{^1^H} NMR spectra. The differences observed also apply for the *Z*-isomers *Z*-5H and *Z*-5B, underlining the fact that these species can be distinguished in both ^31^P{^1^H} and ^19^F{^1^H} NMR spectra. For further details on the calculated isomers and a summary of calculated ^31^P NMR shifts for every discussed isomer please refer to the ESI, Tables S5 and S6.†

To better understand the *E*/*Z* isomerisation process, we also optimized the transition states (TS) between *E*- and *Z*-isomers of the investigated species to estimate the Gibbs free activation energies in relation to the *E*- and *Z*-isomers (Table 4). The isomerisation process is mainly a rotation around the N=N axis of the diazo unit. Starting with pure isonitrile *E*-11, the *E*-isomer is energetically favoured with about 33 kJ mol^−1^ in comparison to the *Z*-isomer *Z*-11. For the *E*-to-*Z* isomerisation an approximate energy barrier of 107 kJ mol^−1^ must be overcome, for the reverse reaction (*Z*-to-*E*) the energy barrier is considerably smaller with about 74 kJ mol^−1^ (note that these values are only estimates; GGAs such as PBE are known to underestimate the activation barriers).^70^ These values underline that especially the *Z*-to-*E* isomerisation might progress slowly at ambient temperature and explain why after freshly synthesizing *E*-11, only the *E*-isomer is obtained.

**Table 4:** Calculated Gibbs free energies [kJ mol^−1^] of the *E*-to-*Z* isomerisation process along with the activation barriers ((U)PBE-D3/def2-TZVP level of theory)^63–68^

	Azobenzene 11	Biradical 5B	Housane 5H
Δ*G*° (*E* → *Z*)	33	34	34
Δ*G*^‡^ (*E* → *Z*)	107	104	109
Δ*G*^‡^ (*Z* → *E*)	74	70	75

The transition states for the *E*-to-*Z* isomerisation process of biradical *E*-5B and housane type species *E*-5H were also calculated utilising only the lowest-lying isomers of each species (Table 4). For the biradical species, the *Z*-isomer *Z*-5B-I^5^ (Fig. 11) lies 34 kJ mol^−1^ higher in energy than the *E*-isomer (*E*-5B-I^6^, Fig. 8). The *E*-to-*Z* isomerisation is associated with an estimated energy barrier of 104 kJ mol^−1^ while the reverse reaction (*Z* to *E*) has an energy barrier of 70 kJ mol^−1^. For the housane type species, the *E*-isomer *E*-5H-I^2^ (Fig. 9) is also favoured by about 34 kJ mol^−1^ in comparison the *Z*-isomer (compound *Z*-5H-I^1^, Fig. 10) and the energy barriers for the isomerisation reactions have approximate values of 109 kJ mol^−1^ (*E* to *Z*) and 75 kJ mol^−1^ (*Z* to *E*), respectively. This means that the activation barriers and isomerisation energies for both compounds of the double switch *E*-5H and *E*-5B differ very little from those of pure diazo-isonitrile (*E*-11, Table 4). This in turn means that a slow *Z*-to-*E* isomerisation is likely to be observed for all three classes of compounds, which is consistent with our experimental observations. This also explains why only the *E*-isomers are obtained after synthesis, as they are the thermodynamically most favourable isomers and are protected from rapid isomerisation to the *Z*-isomer by a relatively high barrier (Table 3). In the irradiation experiments carried out at −40 °C, *Z*-to-*E* isomerisation could not be observed because the (calculated) energy barrier (70 to 75 kJ mol^−1^) is too high to be overcome at such low temperatures.

## Conclusions

In summary, we developed a new class of molecular double switches by combining biradical and azobenzene moieties in the same molecule. These double switches mediated by visible light can undergo either constitutional isomerisation, *i.e.*, housane formation, or stereo isomerisation (*E*/*Z* isomerisation on azobenzene) upon irradiation. Two such molecular double switches were synthesised in good yields and fully characterised (*E*-4B and *E*-5B).

While irradiation of the double switch with red light only triggers the housane formation, irradiation with green light also leads to *E*-to-*Z* isomerisation at the azobenzene moiety. Both molecular switches can be switched independently or simultaneously, depending on the wavelength of the irradiated light. Therefore, depending on the chronological order of irradiation with light of different wavelengths, all four different states of the molecular double switch are accessible (*E*-5B, *E*-5H, *Z*-5H and *Z*-5B; *cf.*Scheme 1) and can be detected by ^19^F{^1^H} and ^31^P{^1^H} NMR spectroscopy. Different rotational isomers of the investigated species *E*-5B, *E*-5H, *Z*-5H and *Z*-5B were also discussed and quantum chemical calculations provided additional information to understand the unique three-dimensional structure of the double switches. These results will further broaden the scope for the understanding and development of new visible light-mediated multicomponent molecular switches.

## Experimental

All manipulations were carried out under oxygen- and moisture free conditions under argon atmosphere using standard Schlenk or drybox techniques. The reported reaction products are extremely sensitive toward oxygen and moisture in most cases and need to be handled carefully to prevent decomposition reactions. All starting materials were produced using literature procedures as stated in the ESI.† The reactants and solvents from commercial sources were dried and purified. If not stated otherwise, experiments and crystallisation attempts were carried out at room temperature (approximately 25 °C). The removal of solvents *in vacuo* was carried out at 1 × 10^−3^ mbar and at room temperature if not stated otherwise. Further information on experimental procedures, data acquisition and processing, purification of starting materials and solvents, and on computational investigations as well as a full set of analytical data for each compound and crystallographic information can be found in the ESI.†

### NMR spectroscopy under irradiation

NMR spectra under irradiation were recorded using our previously published setup, which was adopted from a setup published by the Gschwind group using a fibre-coupled (10 m multimode fibre, 0.39 NA, high OH, 1000 μm core diameter, ThorLabs FT1000UMT) laser diode (red: Oclaro HL63193MG, 638 nm, bandwidth 632–643 nm, 700 mW; green: Osram PL520, 520 nm, bandwidth 510–530 nm, 50 mW; blue: Nichia NDB7875, 445 nm, bandwidth 435–455 nm, 1600 mW).^49,57^

### Synthesis of *E*-4B

Note, in order to prevent housane formation the reaction must be carried out in the dark, thoroughly covering the reaction vessels with aluminium foil! Biradical 1 [˙P(μ-NTer)_2_P˙] (0.18 g, 0.25 mmol) and *E*-10 (0.066 g, 0.25 mmol) were added together in a glass vessel inside an argon filled drybox and dissolved in 10 mL of benzene. The colour of the solution turned from red to an intense blue/black immediately and the mixture was stirred for 30 min at room temperature with a glass stir bar. Subsequently, the solvent of the solution was removed *in vacuo* (1 × 10^−3^ mbar, 50 °C, water bath) yielding product *E*-4B as a blue/black solid. Yield: 0.221 g (0.225 mmol, 90%). Mp. 203 °C (decomp.). CHN calc. (found) in % (deviations due to extreme sensitivity of compound towards moisture): C 79.65 (76.04), H 6.89 (6.45), N 7.14 (6.52). ^31^P{^1^H} NMR (25 °C, C_6_D_6_, 101.3 MHz): *δ* = 221.0 (d, 1 P, N*P*C, ^2^*J*(^31^P,^31^P) = 127 Hz), 257.0 (d, 1 P, N*P*N, ^2^*J*(^31^P,^31^P) = 127 Hz). ^1^H NMR (25 °C, C_6_D_6_, 500.1 MHz): *δ* = 1.64 (s, 6H, azo-C*H*_3_), 1.74 (s, 6H, azo-C*H*_3_), 1.96 (s, 12H, Mes-*o*-C*H*_3_), 2.28 (s, 6H, *p*-C*H*_3_), 2.31 (s, 12H, Mes-*o*-C*H*_3_), 2.35 (s, 6H, *p*-C*H*_3_), 6.64–7.03 (m, 19H, TerC*H*).

### Synthesis of *E*-5B

Note, in order to prevent housane formation the reaction must be carried out in the dark, thoroughly covering the reaction vessels with aluminium foil! Biradical 1 [˙P(μ-NTer)_2_P˙] (0.18 g, 0.25 mmol) and *E*-11 (0.071 g, 0.25 mmol) were added together in a glass vessel inside an argon filled drybox and dissolved in 10 mL of benzene. The colour of the solution turned from red to an intense blue/black immediately and the mixture was stirred for 30 min at room temperature with a glass stir bar. Subsequently, the solvent of the solution was removed *in vacuo* (1 × 10^−3^ mbar, 50 °C, water bath) yielding product *E*-5B as a blue/black solid. Yield: 0.23 g (0.23 mmol, 92%). Compound *E*-5B is present in two sets of isomers (I^A^ and I^B^). Mp. 200.1 °C. CHN calc. (found) in %: C 78.21 (78.14), H 6.66 (6.59), N 7.02 (5.73). ^31^P{^1^H} NMR (25 °C, THF-*d*_8_, 202.5 MHz): *δ* = 219.7 (br. d, 1P, ^2^*J*(^31^P,^31^P) = 127 Hz, I^A^-N*P*C), 222.0 (br. d, 1P, ^2^*J*(^31^P,^31^P) = 136 Hz, I^B^-N*P*C), 257.5 (br. d, 1P, ^2^*J*(^31^P,^31^P) = 127 Hz, I^A^-N*P*N), 258.8 (br. d, 1P, ^2^*J*(^31^P,^31^P) = 136 Hz, I^B^-N*P*N). ^1^H NMR (25 °C, THF-*d*_8_, 500.1 MHz): *δ* = 1.16–1.32 (m, 6H, azo-C*H*_3_), 1.57 (m, 6H, Mes-*p*-C*H*_3_), 1.82 (m, 12H, Mes-*o*-C*H*_3_), 1.98 (s, 6H, Mes-*p*-C*H*_3_), 2.13 (s, 6H, azo-C*H*_3_), 2.34 (m, 12H, Mes-*o*-C*H*_3_), 6.67–6.81 (m, 8H, TerC*H*), 6.90–6.98 (m, 2H, TerC*H*), 7.01–7.07 (m, 2H, TerC*H*), 7.22 (t, 2H, FC(C*H*)_2_), 7.33 (m, 1H, TerC*H*), 7.39–7.47 (m, 1H, TerC*H*), 7.83 (m, 2H, N_2_C(C*H*)_2_). ^19^F{^1^H} NMR (25 °C, THF-*d*_8_, 470.6 MHz): *δ* = −112.3 (s, *F*C).

### Generation of *E*-5H

50 mg of *E*-5B (50 mmol) were dissolved in 0.4 mL of THF-*d*_8_ in an NMR tube equipped with a coaxial insert. The sample was prepared inside an argon filled drybox in the dark, covering the NMR tube thoroughly with aluminium foil. Afterwards the sample was transferred to the spectrometer, cooled to −40 °C and irradiated with light from a red laser diode (638 nm) yielding *E*-5H in solution. ^31^P{^1^H} NMR (−40 °C, THF-*d*_8_, 101.25 MHz): *δ* = −131.9 (br. d, ^1^*J*(^31^P,^31^P) = 62 Hz, I^A^-N*P*C), −130.8 (br. d, ^1^*J*(^31^P,^31^P) = 62 Hz, I^B^-N*P*C), −67.7 (br. d, ^1^*J*(^31^P,^31^P) = 62 Hz, I^B^-N*P*N), −67.1 (br. d, ^1^*J*(^31^P,^31^P) = 62 Hz, I^A^-N*P*N). ^19^F{^1^H} NMR (−40 °C, THF-*d*_8_, 235.36 MHz): *δ* = −111.4 (s, *F*C).

### Generation of *Z*-5H

50 mg of *E*-5B (50 mmol) were dissolved in 0.4 mL of THF-*d*_8_ in an NMR tube equipped with a coaxial insert. The sample was prepared inside an argon filled drybox in the dark, covering the NMR tube thoroughly with aluminium foil. Afterwards the sample was transferred to the spectrometer, cooled to −40 °C and irradiated with light from a green laser diode (520 nm) yielding a mixture of *E*-5H and *Z*-5H in solution. Analytical data for *Z*-5H is presented. ^31^P{^1^H} NMR (−40 °C, THF-*d*_8_, 101.25 MHz): *δ* = −132.5 (br. d, ^1^*J*(^31^P,^31^P) = 62 Hz, I^A^-N*P*C), −129.2 (br. d, ^1^*J*(^31^P,^31^P) = 62 Hz, I^B^-N*P*C), −67.2 (br. d, ^1^*J*(^31^P,^31^P) = 62 Hz, I^A^-N*P*N), −55.9 (br. d, ^1^*J*(^31^P,^31^P) = 62 Hz, I^B^-N*P*N). ^19^F{^1^H} NMR (−40 °C, THF-*d*_8_, 235.36 MHz): *δ* = −114.3 (s, I^A^-*F*C), −113.3 (s, I^B^-*F*C).

### Generation of *Z*-5B

A solution containing *E*-5H and *Z*-5H (*vide supra*) is left in the dark at room temperature for approx. 30 minutes, yielding a mixture of *E*-5B and *Z*-5B in solution. Analytical data for *Z*-5B is presented. ^31^P{^1^H} NMR (−40 °C, THF-*d*_8_, 101.25 MHz): *δ* = 219.4 (br. d, ^2^*J*(^31^P,^31^P) = 120 Hz, I^A^-N*P*C), 221.5 (br. d, ^2^*J*(^31^P,^31^P) = 130 Hz, I^B^-N*P*C), 253.3 (br. d, ^2^*J*(^31^P,^31^P) = 120 Hz, I^A^-N*P*N), 255.2 (br. d, ^2^*J*(^31^P,^31^P) = 130 Hz, I^B^-N*P*N). ^19^F{^1^H} NMR (−40 °C, THF-*d*_8_, 235.36 MHz): *δ* = −114.9 (s, I^A^-*F*C), −114.5 (s, I^B^-*F*C).

## Author contributions

Y. P. carried out the experimental work on the fluorinated and H. B. on the non-fluorinated compounds. Y. P. and J. B. performed the computational studies. Y. P. and D. M. performed the photoswitching reactions. A. V. recorded the SCXRD data and solved the structures. Y. P., H. B., J. B. and A. S. conceptualised the project. Y. P. wrote the ESI,† Y. P. and A. S. wrote the manuscript. All authors contributed for further revision of the ESI† and manuscript.

## Conflicts of interest

There are no conflicts to declare.

## Supplementary Material

SC-016-D4SC07247B-s001

SC-016-D4SC07247B-s002

## Data Availability

Further information on experimental procedures, data acquisition and processing, purification of starting materials and solvents, and on computational investigations as well as a full set of analytical data for each compound and crystallographic information can be found in the ESI.†
