# Supplementary material for: Designing a visible light-mediated double photoswitch: a combination of biradical and azobenzene structural motifs that can be switched independently
Source: Chem Sci. 2024 Dec 3;16(2):876–88. doi: 10.1039/d4sc07247b (PMC11626401; doi:10.1039/d4sc07247b)
Supplement: SC-016-D4SC07247B-s001 [file SC-016-D4SC07247B-s001.pdf]

Supplementary Information (SI) for Chemical Science.
This journal is © The Royal Society of Chemistry 2024

SUPPORTING INFORMATION

Designing a visible light-mediated double photoswitch: A combination of biradical and azobenzene structural motifs that can be switched independently

Y. Pilopp, H. Beer, J. Bresien, D. Michalik, A. Villinger, A. Schulz

This file includes:

1 Experimental 3

2 Structure elucidation 6

3 Syntheses of starting materials 11

4 Syntheses of compounds 32

5 Additional spectroscopic details 41

6 Computational details 70

7 References 136

1 Experimental

General Information. If not stated otherwise, all manipulations were carried out under oxygen- and moisture-free conditions under an inert atmosphere of argon using standard Schlenk or Drybox techniques. All glassware was heated three times *in vacuo* using a heat gun and cooled under argon atmosphere. Solvents were transferred using syringes, which were purged three times with argon prior to use. Solvents and reactants were either obtained from commercial sources or synthesised as detailed in Table S1.

Table S1: Origin and purification of solvents and reactants.

Substance	Origin	Purification
CH_2Cl_2	local trade	purified according to literature procedure[1]<br>dried over P_4O_{10}, stored over CaH_2<br>freshly distilled and degassed (freeze-pump-thaw)
CH_2Cl_2 (for syntheses under non-inert conditions)	local trade	used as received
n-pentane,<br>*n*-hexane	local trade	dried over Na/benzophenone/tetraglyme<br>freshly distilled prior to use
C_6H_6, toluene	local trade	dried over Na/benzophenone<br>freshly distilled prior to use
petroleum ether 40/60	local trade	used as received
toluene	local trade	used as received
HOAc	local trade, 100%	used as received
EtOAc	local trade	used as received
CD_2Cl_2	euriso-top	used as received
C_6D_6	euriso-top	dried over Na<br>freshly distilled prior to use
THF-d_8	euriso-top	dried over Na<br>distilled and stored over molecular sieves (4 Å)
NEt_3	Sigma Aldrich, 99%	dried over Na<br>freshly distilled prior to use
$POCl_3$	old stock	dried over P_4O_{10}, freshly distilled and degassed (freeze-pump-thaw)

Table S1 continued.

Substance	Origin	Purification
2,3,5,6-tetramethyl-1,4-phenylene-diamine	TCI	used as received
4-fluoroaniline, aniline	old stock	freshly distilled prior to use
Oxone, monopersulfate	abcr	used as received
formic acid	old stock	used as received
$NaHCO_3$	J. T. Baker	used as received
$[P(\mu\text{-NTer})]_2$	synthesised[2–4]	re-crystallised as described in literature[2–4]

NMR spectra were recorded on Bruker spectrometers (AVANCE 250, AVANCE 300 or AVANCE 500) and were referenced internally to the deuterated solvent (^{13}C: CD_2Cl_2 δ_{ref} = 54.0 ppm, C_6D_6 δ_{ref} = 128.4 ppm, THF-d_7 $\delta_{ref,1}$ = 25.3 ppm, $\delta_{ref,2}$ = 67.2 ppm), to the protonated species in the deuterated solvent (^{1}H: $CHDCl_2$ δ_{ref} = 5.32 ppm, C_6HD_5 δ_{ref} = 7.16 ppm, THF-d_7 $\delta_{ref,1}$ = 1.73 ppm, $\delta_{ref,2}$ = 3.58 ppm) or externally (^{19}F: $FCCl_3$ δ_{ref} = 0 ppm, ^{31}P: 85% H_3PO_4 $\delta_{ref.}$ = 0 ppm). All measurements were carried out at ambient temperature unless denoted otherwise. NMR signals were assigned using experimental data (e.g. chemical shifts, coupling constants, integrals where applicable) in conjunction with computed NMR data (GIAO method, *cf.* section 6.1). The signs of $^{n}J(^{31}P,^{31}P)$ coupling constants were derived from calculated values.

IR spectra of crystalline samples were recorded on a Bruker Alpha II FT-IR spectrometer equipped with an ATR unit at ambient temperature under argon atmosphere. Relative intensities are reported according to the following intervals: very weak (vw, 0–10%), weak (w, 10–30%), medium (m, 30–60%), strong (s, 60–90%), very strong (vs, 90–100%).

Raman spectra of crystalline samples were recorded using a LabRAM HR 800 Horiba Jobin YVON Raman spectrometer equipped with an Olympus BX41 microscope with variable lenses. The samples were excited by a red laser (633 nm, 17 mW, air-cooled

HeNe laser). All measurements were carried out at ambient temperature unless stated otherwise.

Elemental analyses were obtained using an Elementar vario Micro cube CHNS analyser.

Melting points (uncorrected) were determined using a Stanford Research Systems EZ Melt at a heating rate of 20 °C/min. Clearing points are reported.

Mass spectra were recorded on a Thermo Electron MAT 95-XP sector field mass spectrometer using crystalline samples.

UV-Vis spectra were acquired on an Agilent Cary 60 UV-Vis spectrometer. 6Q quartz cuvettes (d = 10 mm) were used as sample vessels.

2 Structure elucidation

X-ray Structure Determination: X-ray quality crystals were selected in Fomblin YR-1800 perfluoroether (Alfa Aesar) at ambient temperature. The samples were cooled to 123(2) K during measurement. The data were collected on a Bruker D8 Quest diffractometer or a Bruker Kappa Apex II diffractometer using Mo K_α radiation (λ = 0.71073 Å). The structures were solved by iterative methods (SHELXT)[5] and refined by full matrix least squares procedures (SHELXL).[6] Semi-empirical absorption corrections were applied (SADABS).[7] All non-hydrogen atoms were refined anisotropically, hydrogen atoms were included in the refinement at calculated positions using a riding model.

Figure S1. Molecular structure of **4-Amino-2,3,5,6-tetramethyl-azobenzene (*E*-6)** in the single crystal. Ellipsoids are set at 50% probability (123 K). Colour code: grey = carbon; white = hydrogen; blue = nitrogen.

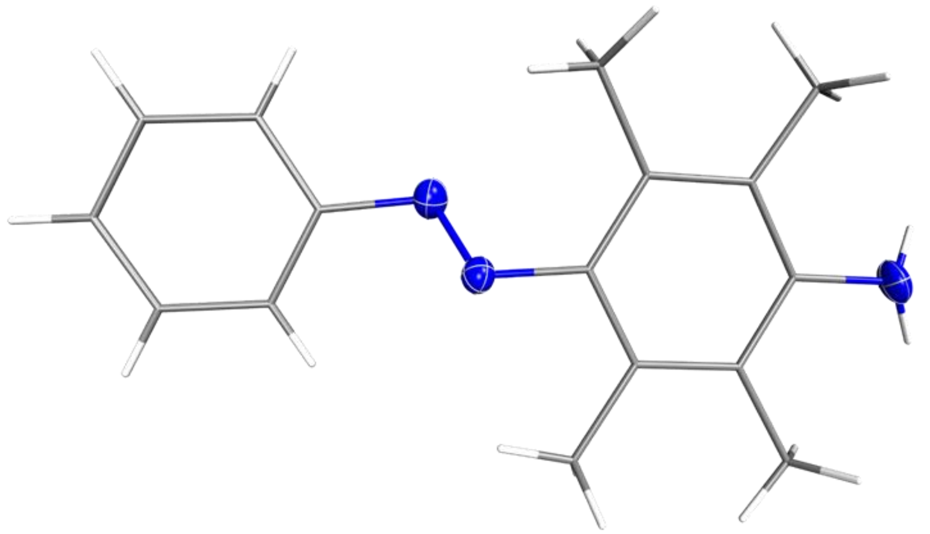

Figure S2. Molecular structure of **4-Formamido-2,3,5,6-tetramethyl-azobenzene (*E*-8)** in the single crystal. Ellipsoids are set at 50% probability (123 K). Colour code: grey = carbon; white = hydrogen; blue = nitrogen; red = oxygen.

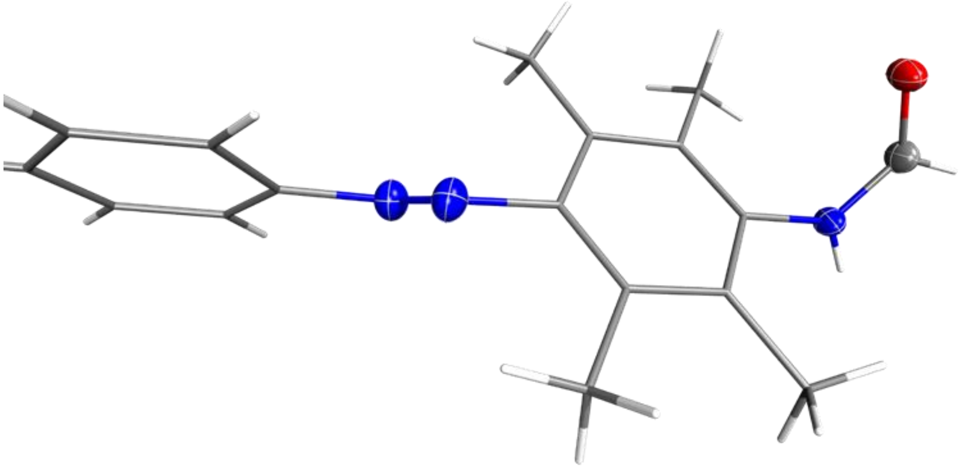

Figure S3. Molecular structure of **4-Isocyano-2,3,5,6-tetramethyl-azobenzene (*E*-10)** in the single crystal. Ellipsoids are set at 50% probability (123 K). Colour code: grey = carbon; white = hydrogen; blue = nitrogen.

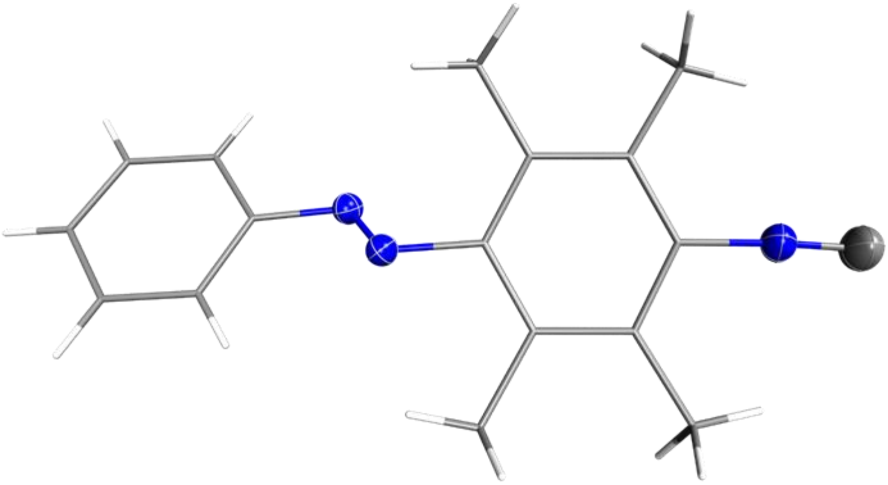

Figure S4. Molecular structure of ***E*-4B** in the single crystal. Ellipsoids are set at 50% probability (123 K). Colour code: grey = carbon; white = hydrogen; blue = nitrogen; orange = phosphorus.

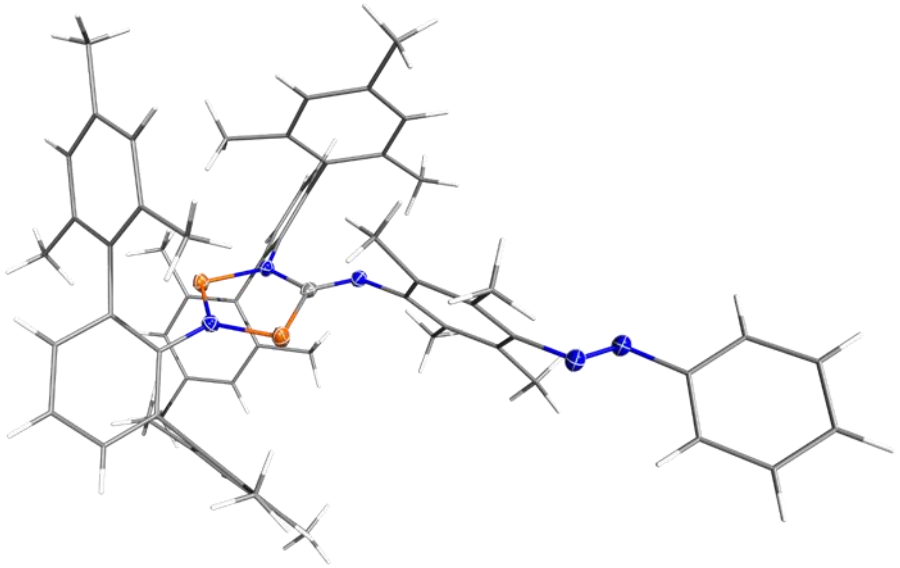

Figure S5. Molecular structure of ***E*-5B** in the single crystal. Ellipsoids are set at 50% probability (123 K). Colour code: grey = carbon; white = hydrogen; blue = nitrogen; orange = phosphorus; turquoise = fluorine.

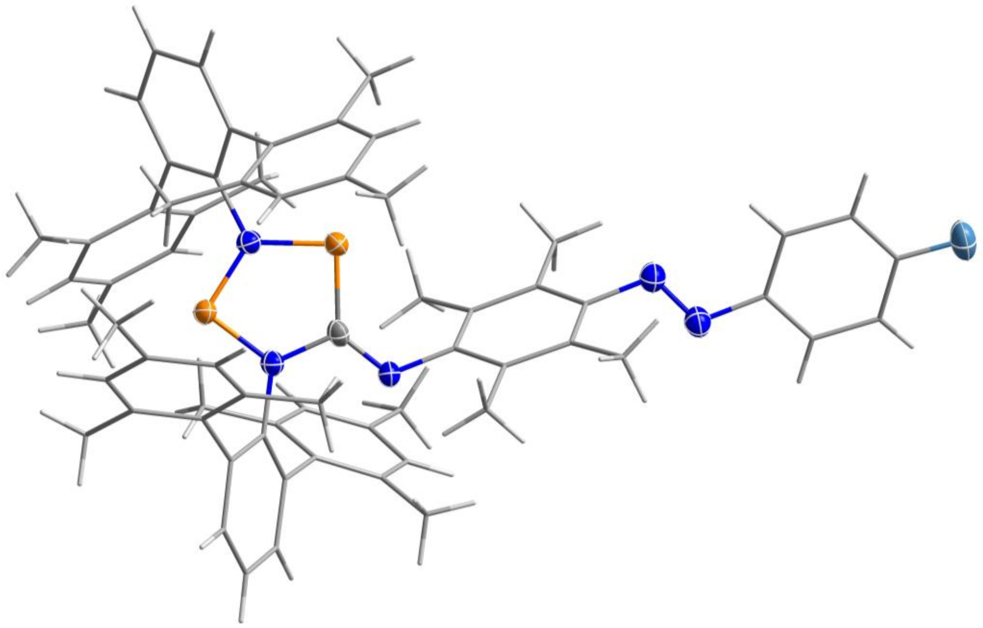

Table S2: Crystallographic details.

Compound	***E*-6**	***E*-8**	***E*-10**
Chem. Formula	$C_{16}H_{19}N_3$	$C_{17}H_{19}N_3O$	$C_{17}H_{17}N_3$
Formula weight [g/mol]	253.34	281.35	263.33
Colour	orange	orange	orange
Crystal system	monoclinic	orthorhombic	monoclinic
Space group	$P2_1$	$P2_12_12_1$	$P2_1/c$
a [Å]	7.8385(6)	4.6237(6)	7.2792(8)
b [Å]	5.2530(4)	9.0404(11)	9.3758(10)
c [Å]	16.8163(13)	35.034(5)	20.838(2)
α [°]	90	90	90
β [°]	103.167(2)	90	99.559(2)
γ [°]	90	90	90
V [Å^3]	674.22(9)	1464.4(3)	1402.4(3)
Z	2	4	4
$\rho_{calcd.}$ [g/cm^3]	1.248	1.276	1.247
μ [mm^{-1}]	0.076	0.082	0.076
T [K]	123(2)	123(2)	123(2)
Measured reflections	15179	19703	29154
Independent reflections	3265	3903	4472
Reflections with $I > 2\sigma(I)$	2350	3277	3479
R_{int}	0.0584	0.0588	0.0432
$F(000)$	272	600	560
$R_1(R[F^2>2\sigma(F^2)])$	0.0504	0.0439	0.0474
w$R_2(F^2)$	0.1231	0.1135	0.1374
GooF	1.048	1.052	1.053
No. of Parameters	185	199	185
CCDC #	2291635	2291636	2291637

Table S2 continued.

Compound	***E*-4B**	***E*-5B**
Chem. Formula	$C_{65}H_{67}N_5P_2$	$C_{65}H_{66}FN_5P_2$
Formula weight [g/mol]	980.17	998.16
Colour	black	black
Crystal system	monoclinic	monoclinic
Space group	$P2_1/c$	$P2_1/n$
a [Å]	10.8196(11)	10.8647(7)
b [Å]	21.236(2)	21.2993(15)
c [Å]	24.229(3)	24.1947(18)
α [°]	90	90
β [°]	102.703(3)	102.911(2)
γ [°]	90	90
V [Å^3]	5430.8(10)	5457.4(7)
Z	4	4
$\rho_{calcd.}$ [g/cm^3]	1.199	1.215
μ [mm^{-1}]	0.126	0.129
T [K]	123(2)	123(2)
Measured reflections	83011	10466
Independent reflections	13083	10466
Reflections with $I > 2\sigma(I)$	9484	7926
R_{int}	0.1107	0.247
F(000)	2088	2120
$R_1(R[F^2>2\sigma(F^2)])$	0.0523	0.0852
$wR_2(F^2)$	0.1427	0.1943
GooF	1.034	1.080
No. of Parameters	665	674
CCDC #	2291638	2291639

3 Syntheses of starting materials

3.1 4-Amino-2,3,5,6-tetramethyl-azobenzene (*E*-6)

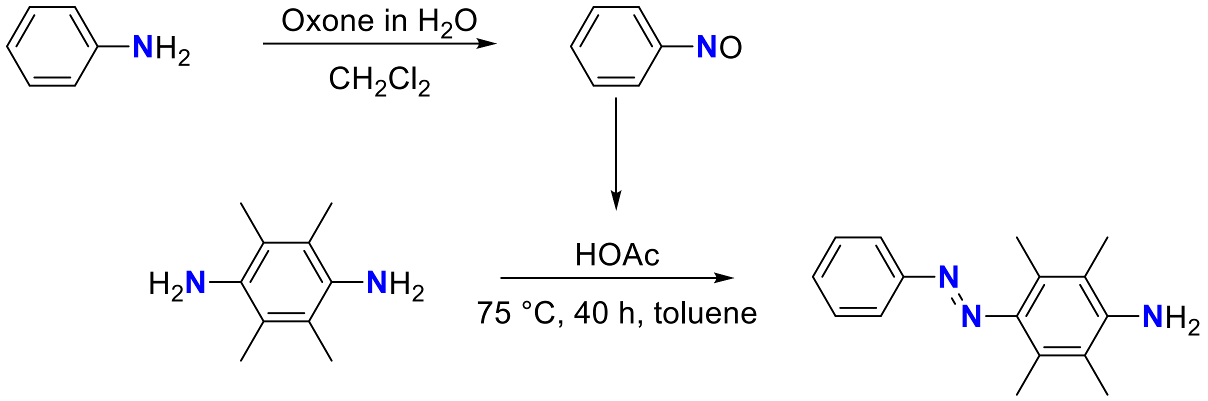

4-Amino-2,3,5,6-tetramethyl-azobenzene was synthesised according to a modified literature procedure.[8] *The synthesis was carried out under non-inert conditions.*

Oxone (Potassium peroxymonosulfate, 31.1 g, 50.6 mmol) was dissolved in 120 mL of water and added dropwise to a stirred solution of aniline (4.77 g, 51.9 mmol) in 120 mL of dichloromethane. The mixture was stirred for 1 h upon which the colour of the organic phase changed to an intense green. The resulting suspension was diluted with water (300 mL) and after separation of the phases the aqueous phase was extracted three times with CH_2Cl_2 (total amount of 300 mL). The solvent of the combined organic layers was removed using a rotary evaporator at 40 °C. The green oil of nitrosobenzene was dissolved in 20 mL of toluene and added to a stirred suspension of 2,3,5,6-tetramethyl-1,4-phenylenediamine (5.62 g, 34.2 mmol) in 100 mL of toluene whereupon HOAc (100%, 7.80 g, 130 mmol) was added. The mixture was stirred at 75 °C (oil bath) for further 35 h while a change of colour to brown occurred. The suspension was diluted with dichloromethane (150 mL) and after separation of the phases the organic phase was washed with water (200 mL). After repeated separation of the phases the aqueous phase was extracted three times with CH_2Cl_2 (total amount

of 500 mL). The solvent of the combined organic layers was removed using a rotary evaporator (40 °C) and the crude product purified by column chromatography (petroleum ether/EtOAc 10:1). The solvent of the intensely red coloured organic phase was removed using a rotary evaporator yielding a red solid of 4-amino-2,3,5,6-tetramethyl-azobenzene (***E*-6**). Yield: 0.86 g (3.4 mmol, 10%).

$C_{16}H_{19}N_3$ (253.16 g/mol) **Mp**. 108 °C. **CHN** calc. (found) in %: C 75.85 (76.15), 7.56 (7.60), 16.59 (16.14). **^{1}H NMR** (25 °C, CD_2Cl_2, 300.1 MHz): δ = 2.15 (s, 6 H, C*H*$_3$), 2.31 (s, 6 H, C*H*$_3$), 3.89 (broad s, 2 H, N*H*$_2$), 7.40-7.56 (m, 3 H, *m*/*p*-C*H*), 7.80-7.86 (m, 2 H, *o*-C*H*). **^{13}C{^{1}H} NMR** (25 °C, CD_2Cl_2, 75.5 MHz): δ = 13.5 (s, *C*H$_3$), 15.85 (s, *C*H$_3$), 118.9 (s, quart. *C*), 122.6 (s, *o*-CH), 128.9 (s, quart. *C*), 129.5 (s, *m*-CH), 130.4 (s, *o*-CH*),* 144.4 (s, quart. *C*), 145.3 (s, quart. *C*), 153.8 (s, quart. *C*). **IR** (ATR, 32 scans, cm^{-1}): $\tilde{\nu}$ = 3433 (w), 3346 (w), 3062 (w), 2994 (w), 2918 (w), 2873 (w), 2854 (w), 1626 (w), 1585 (w), 1562 (m), 1465 (m), 1447 (m), 1399 (w), 1296 (m), 1257 (m), 1191 (w), 1166 (w), 1107 (m), 1074 (m), 1018 (m), 999 (m), 985 (w), 917 (w), 905 (w), 841 (w), 824 (w), 762 (s), 746 (s), 684 (vs), 637 (s), 620 (s), 610 (s), 581 (s), 550 (s), 521 (s), 486 (m), 470 (s), 433 (m), 406 (s). **Raman** (633 nm, 5 s, 5 scans, cm^{-1}): $\tilde{\nu}$ = 3439 (1), 3352 (1), 3062 (1), 2917 (1), 2914 (1), 2899 (1), 1624 (1), 1593 (2), 1584 (1), 1567 (1), 1485 (7), 1470 (10), 1445 (8), 1429 (7), 1366 (1), 1302 (7), 1260 (2), 1194 (10), 1153 (9), 1111 (1), 1074 (2), 1063 (3), 1019 (1), 998 (6), 986 (1), 966 (1), 918 (1), 837 (1), 764 (1), 697 (1), 637 (1), 612 (1), 579 (1), 571 (1), 553 (1), 522 (1), 473 (2), 449 (1), 425 (1), 408 (1), 280 (2), 233 (2).

Figure S6: NMR, IR and Raman spectra of ***E*-6** (solvent signals indicated by asterisks).

^{1}H NMR spectrum

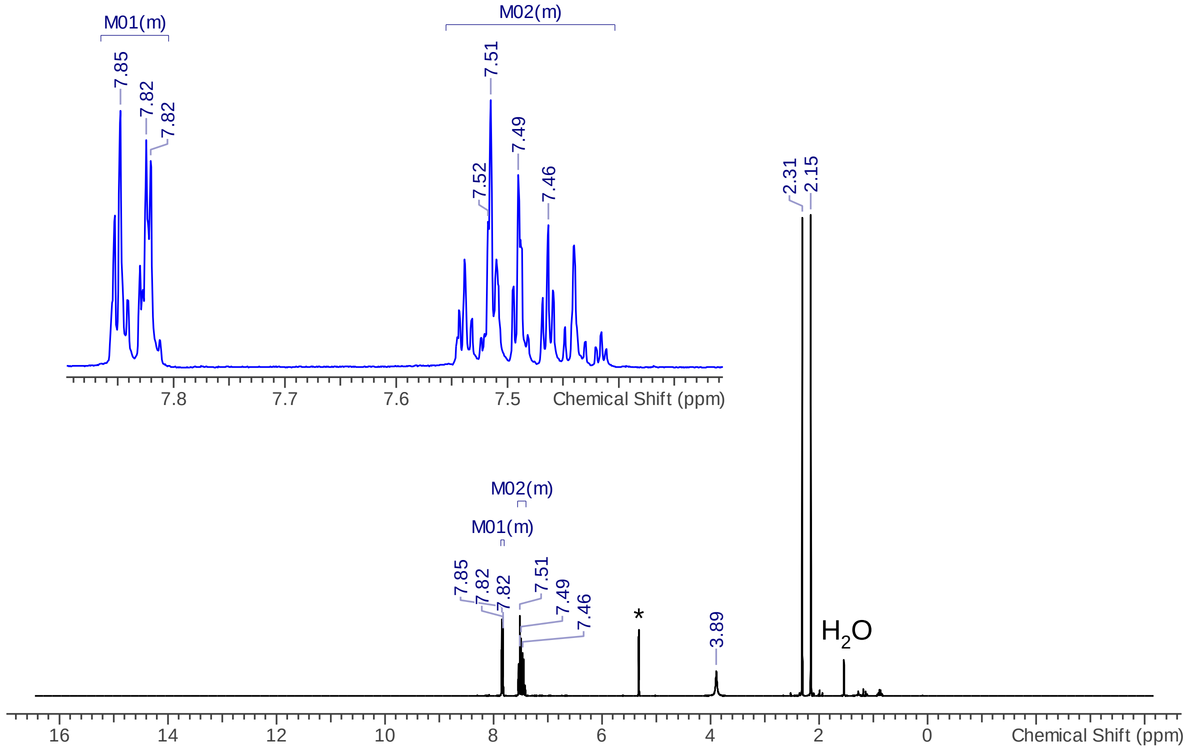

$^{13}C\{^{1}H\}$ NMR spectrum

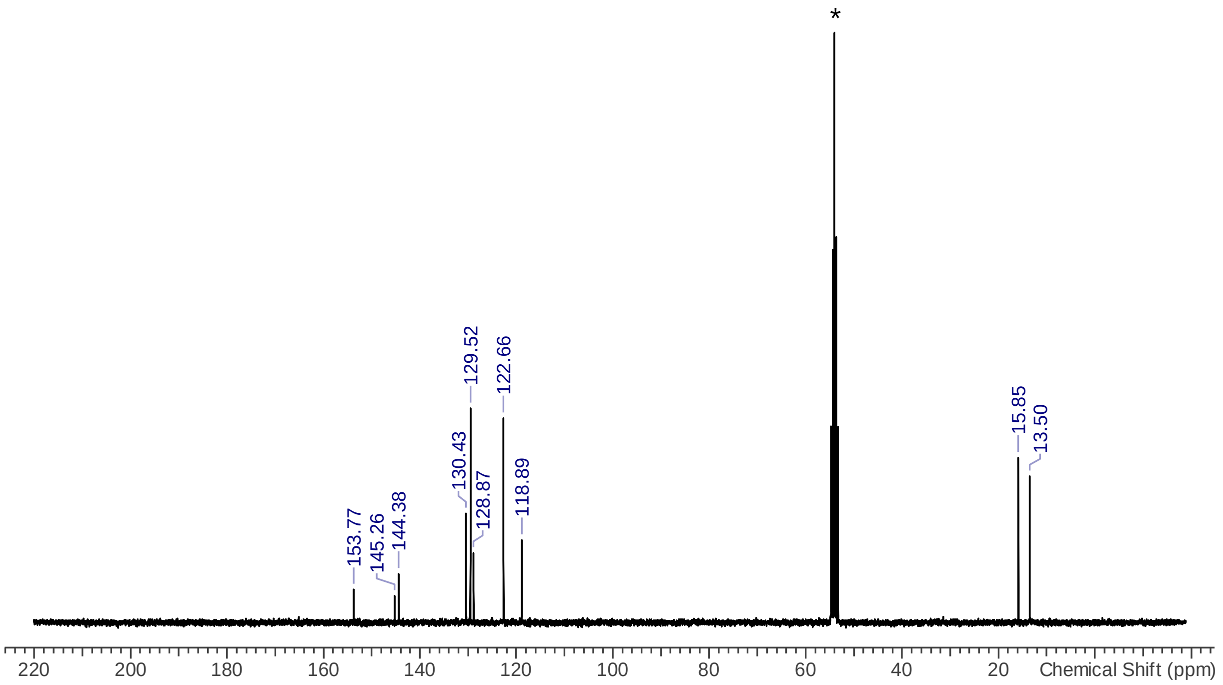

Figure S6 continued.

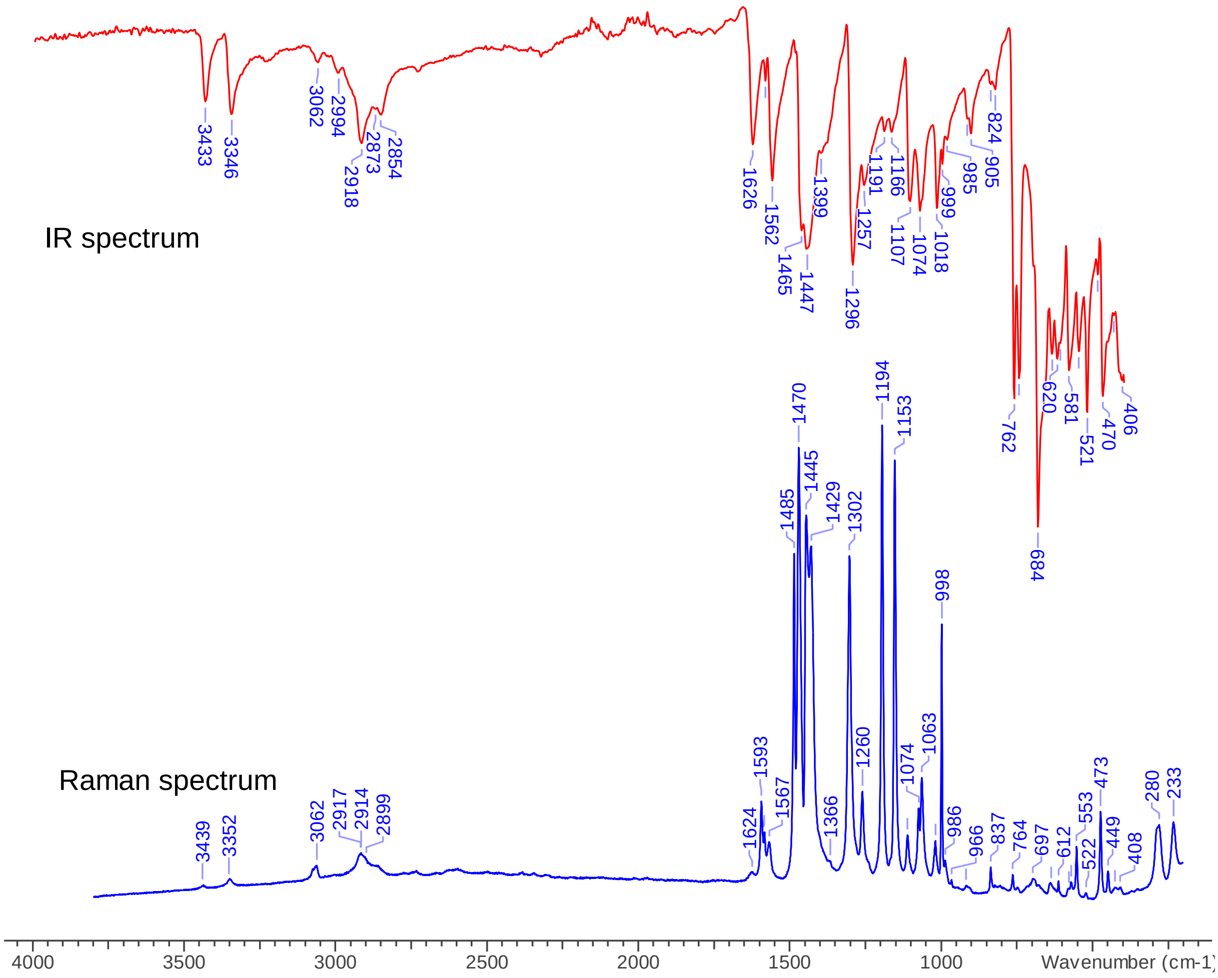

3.2 4-Formamido-2,3,5,6-tetramethyl-azobenzene (*E*-8)

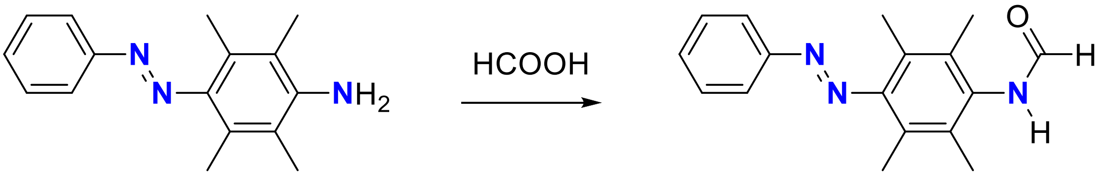

***E*-8** *was synthesised according to a modified literature procedure.*[9] *The synthesis was carried out under non-inert conditions.*

***E*-6** (0.64 g, 2.5 mmol) was dissolved in formic acid (3.0 g, 65 mmol) and degassed completely *via* two pump-freeze-thaw cycles. Afterwards the reaction mixture was stored in a drying oven at 60 °C for 70 h resulting in red needles. The crude product was washed three times with 25 mL of water and dried *in vacuo* (1×10^{-3} mbar, 100 °C, oil bath). Yield: 0.47 g (1.7 mmol, 66 %). ***E*-8** is present in two sets of isomers (a and b) due to rotation around the N−CO bond.

$C_{17}H_{19}N_3O$ (281.15 g/mol) **Mp**. 105 °C. **CHN** calc. (found) in %: 72.57 (72.57), 6.81 (6.83), 14.94 (14.66). **^{1}H NMR** (25 °C, CD_2Cl_2, 500.1 MHz): δ = 2.09 (s, 6 H, CH_3, (a)), 2.09 (s, 6 H, CH_3, (b)), 2.18 (s, 6 H, CH_3, (a)), 2.26 (s, 6 H, CH_3, (b)), 7.05 (broad s, 1 H, N*H*, (a)), 7.05 (broad s, 1 H, N*H*, (b)), 7.47-7.62 (m, 5 H, C*H,* (a)), 7.87-7.94 (m, 5 H, C*H,* (b)), 8.00 (m, 1 H, C*H*O, (a)), 8.42 (s, 1 H, C*H*O, (b)). **^{13}C{^{1}H} NMR** (25 °C, CD_2Cl_2, 125.8 MHz): δ = 14.5 (s, CH_3), 14.8 (s, CH_3), 15.0 (s, CH_3), 122.5 (s, CH_3), 122.5 (s, CH_3), 125.6 (s, quart. *C*), 125.9 (s, quart. *C*), 129.1 (s, arom. *C*), 129.2 (s, arom. *C*), 131.3 (s, arom. *C*), 131.5 (s, arom. *C*), 131.7 (s, quart. *C*), 132.1 (s, quart. *C*), 132.6 (s, quart. *C*), 133.0 (s, quart. *C*), 152.6 (s, quart. *C*), 152.6 (s, quart. *C*), 152.7 (s, quart. *C*), 159.6 (s, *C*), 164.9 (s, *C*). **IR** (ATR, 32 scans, cm^{-1}): $\tilde{\nu}$ = 3239 (m), 3175 (w), 3056 (w), 2990 (w), 2955 (w), 2920 (m), 2891 (w), 2732 (w), 1642 (vs), 1587 (m), 1568 (w), 1515 (s), 1492 (m), 1471 (m), 1451 (s), 1436 (m), 1403 (m), 1391 (s), 1381 (s), 1302 (m), 1271 (m), 1259 (m), 1232 (w), 1195 (s), 1148 (m), 1076 (m), 1065 (m), 1034 (m), 1014 (m), 1001 (m), 968 (w), 952 (w), 927 (w), 886 (m), 865 (w), 839 (w), 822 (w), 800 (m), 764 (s), 740 (s), 686 (vs), 647 (m), 626 (s), 614 (m), 587 (m), 567 (s), 548 (m), 519 (m), 482 (m), 470 (m), 429 (s), 408 (m). **Raman** (633 nm,

5 s, 5 scans, cm^{-1}): $\tilde{\nu}$ = 3246 (2), 3067 (2), 3057 (2), 2922 (4), 2865 (2), 2730 (2), 1644 (4), 1596 (5), 1588 (4), 1572 (4), 1493 (8), 1476 (8), 1451 (7), 1393 (4), 1383 (3), 1373 (3), 1313 (6), 1305 (5), 1261 (3), 1190 (7), 1163 (4), 1149 (9), 1078 (4), 1067 (4), 1041 (3), 1022 (3), 1003 (10), 989 (3), 909 (3), 889 (3), 840 (3), 803 (2), 767 (2), 739 (2), 696 (2), 649 (2), 614 (3), 573 (2), 549 (4), 522 (2), 486 (3), 471 (2), 452 (2), 432 (2), 410 (1), 384 (2), 356 (3), 289 (1), 265 (2), 252 (2).

Figure S7: NMR, IR and Raman spectra of ***E*-8** (solvent signals indicated by asterisks).

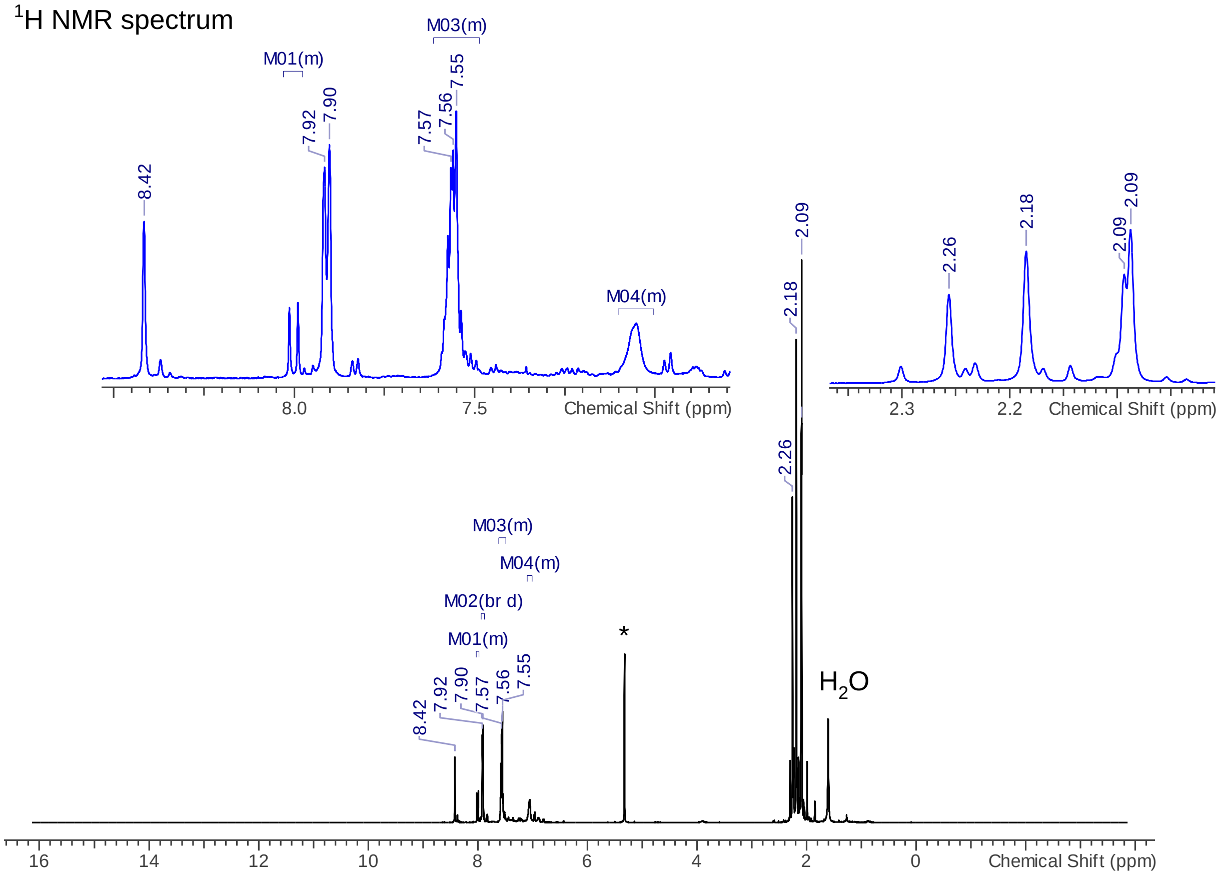

Figure S7 continued.

$^{13}C\{^{1}H\}$ NMR spectrum

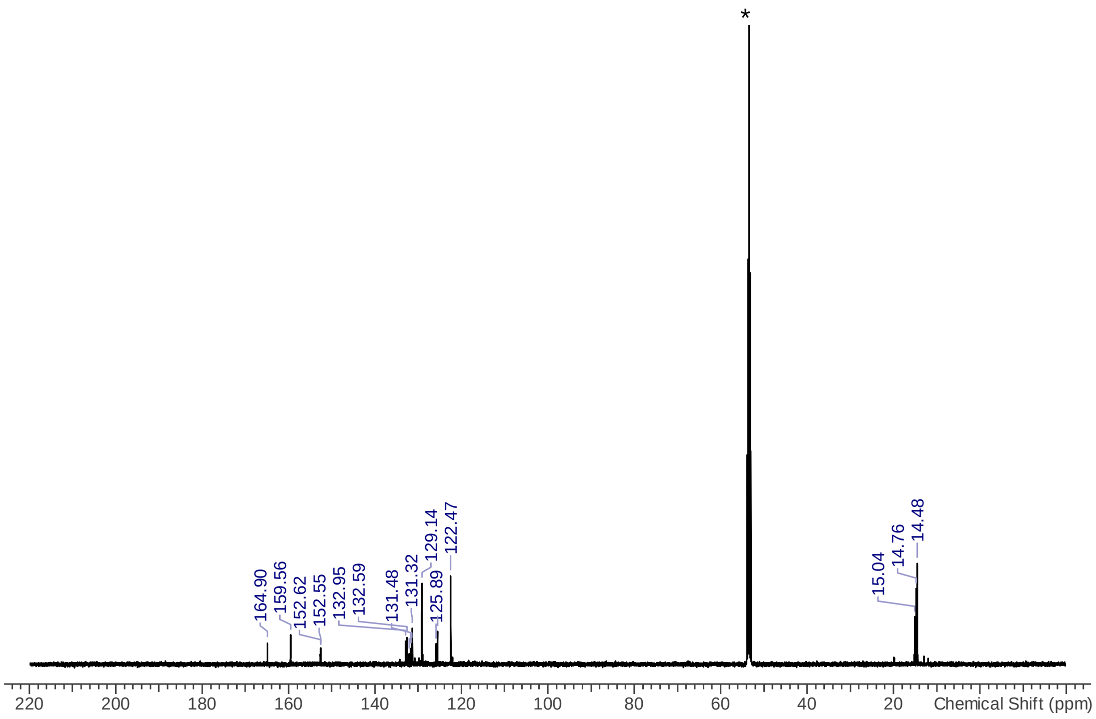

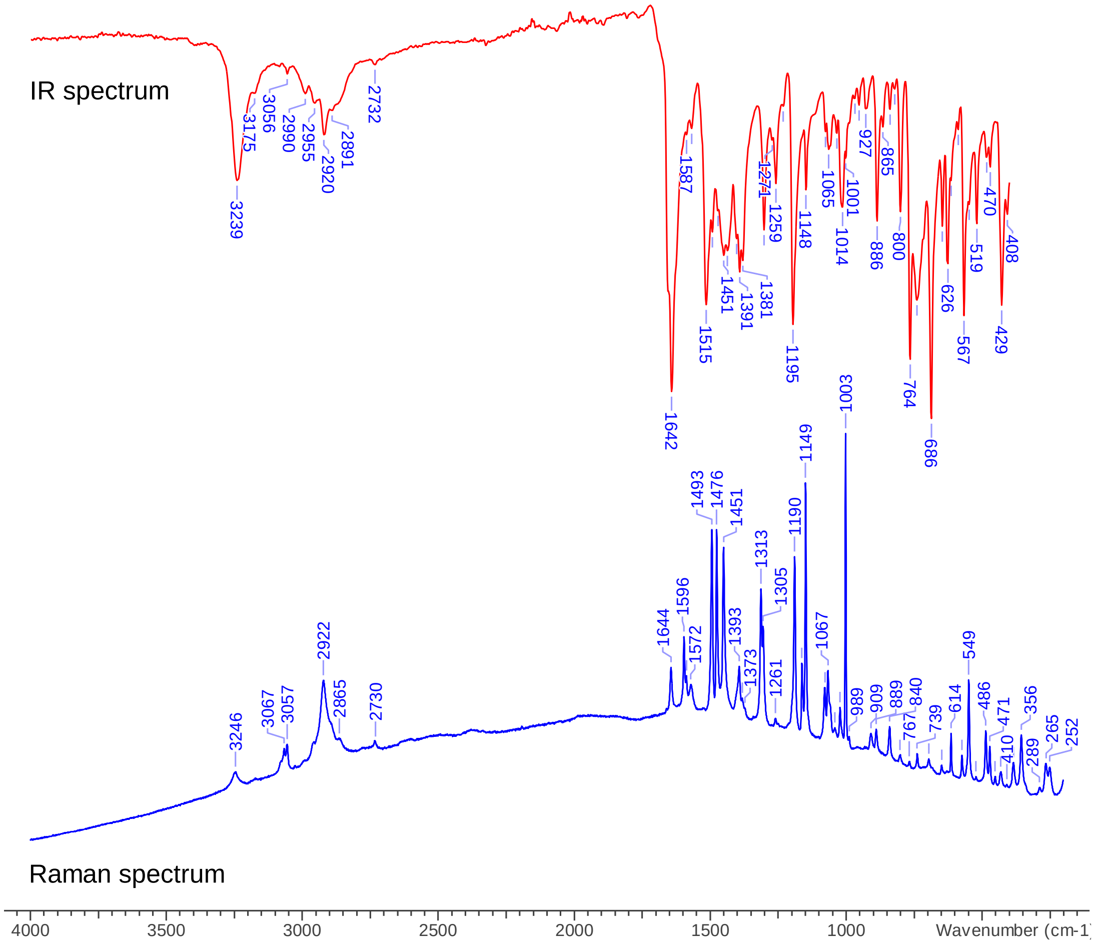

3.3 4-Isocyano-2,3,5,6-tetramethyl-azobenzene (*E*-10)

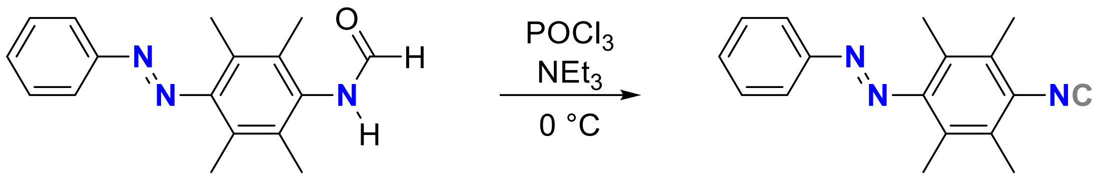

***E*-10** *was synthesised according to a modified literature procedure.*[9]

***E*-8** (0.30 g, 1.1 mmol) was dissolved in 5 mL of CH_2Cl_2. Approximately two equivalents of phosphoryl trichloride (0.330 g, 2.15 mmol) were added under stirring at 0 °C followed by the dropwise addition of ten equivalents of NEt_3 (1.08 g, 10.7 mmol). The reaction mixture was stirred for 48 h at room temperature, turning brownish and becoming cloudy in the process. Afterwards the brown solution was diluted in 100 mL of ice-water. The aqueous phase was extracted three times with CH_2Cl_2 (total amount of 90 mL). The combined organic layers were extracted two times with 50 mL water and two times with 50 mL of a saturated $NaHCO_3$ solution. After separation of the phases the solvent of the organic layer was removed *in vacuo* (1×10^{-3} mbar, 30 °C, water bath). The crude product was purified by sublimation *in vacuo* (1×10^{-3} mbar, 120 °C, oil bath) yielding a red solid of ***E*-10**. Yield: 0.13 g (0.48 mmol, 45 %).

$C_{17}H_{17}N_3$ (263.15 g/mol) **Mp**. 131 °C **CHN** calc. (found) in %: C 77.24 (77.46), 6.86 (6.55), 15.90 (16.05). **^{1}H NMR** (25 °C, CD_2Cl_2, 500.1 MHz): δ = 2.08 (s, 6 H, C*H*3), 2.40 (s, 6 H, C*H*3), 7.48-7.66 (m, 3 H, *m*/*p*-C*H*), 7.84-7.97 (m, 2 H, *o*-C*H*). **^{13}C{^{1}H} NMR** (25 °C, CD_2Cl_2, 125.8 MHz): δ = 14.9 (s, *C*H3), 16.2 (s, *C*H3), 121.0 (s, quart. *C*), 123.1 (s, arom. *C*), 126.5 (s, quart. *C*), 129.8 (s, arom. *C*), 132.3 (s, arom. *C*), 132.4 (s, quart. *C*), 153.1 (s, quart. *C*), 153.7 (s, quart. *C*), 168.4 (s, N*C*). **IR** (ATR, 32 scans, cm^{-1}): $\tilde{\nu}$ = 3233 (w), 3066 (w), 3009 (w), 2955 (w), 2922 (m), 2862 (w), 2730 (w), 2112 (w), 1896 (vw), 1809 (vw), 1766 (vw), 1692 (w), 1657 (s), 1616 (w), 1587 (w), 1525 (m), 1490 (m), 1473 (m), 1447 (m), 1381 (m), 1341 (w), 1302 (m), 1273 (w), 1255 (w), 1228 (w), 1191 (m), 1146 (m), 1105 (w), 1074 (w), 1053 (m), 1016 (m), 925 (m), 880 (w), 863 (w), 822 (w), 771 (vs), 742 (m), 688 (vs), 645 (m), 614 (w), 587 (w), 558 (m), 548 (w), 538 (w), 519 (m), 497 (w), 480 (w), 462 (w), 431 (m), 408 (m). **Raman** (633 nm, 10 s, 10 scans, cm^{-1}): $\tilde{\nu}$ = 3072 (1), 3050 (1), 2925 (1),

2115 (2), 1594 (3), 1588 (2), 1570 (2), 1493 (9), 1474 (6), 1449 (7), 1400 (1), 1386 (2), 1373 (2), 1310 (5), 1297 (3), 1255 (1), 1190 (8), 1156 (2), 1147 (10), 1075 (5), 1066 (3), 1039 (1), 1019 (1), 1001 (5), 928 (1), 903 (2), 852 (1), 742 (1), 697 (1), 650 (1), 615 (1), 569 (1), 539 (2), 520 (1), 499 (1), 463 (1), 454 (1), 431 (1), 352 (1), 326 (1), 265 (1), 209 (1).

Figure S8: NMR, IR and Raman spectra of ***E*-10** (solvent signals indicated by asterisks).

^{1}H NMR spectrum

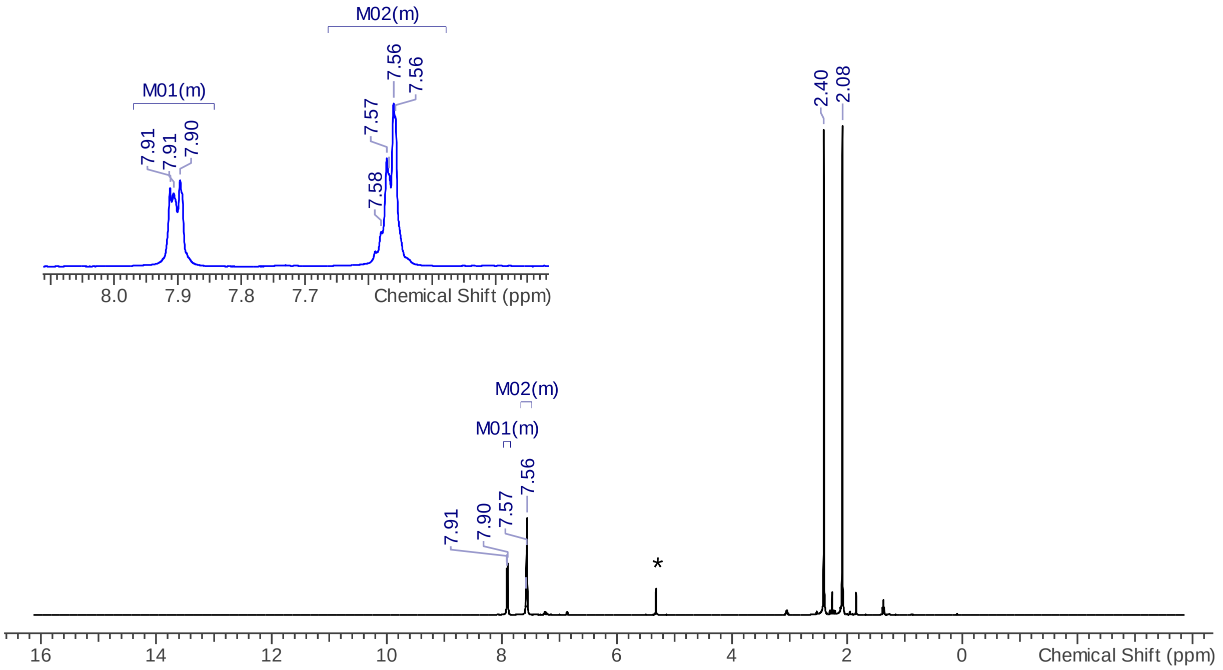

Figure S8 continued.

$^{13}C\{^{1}H\}$ NMR spectrum

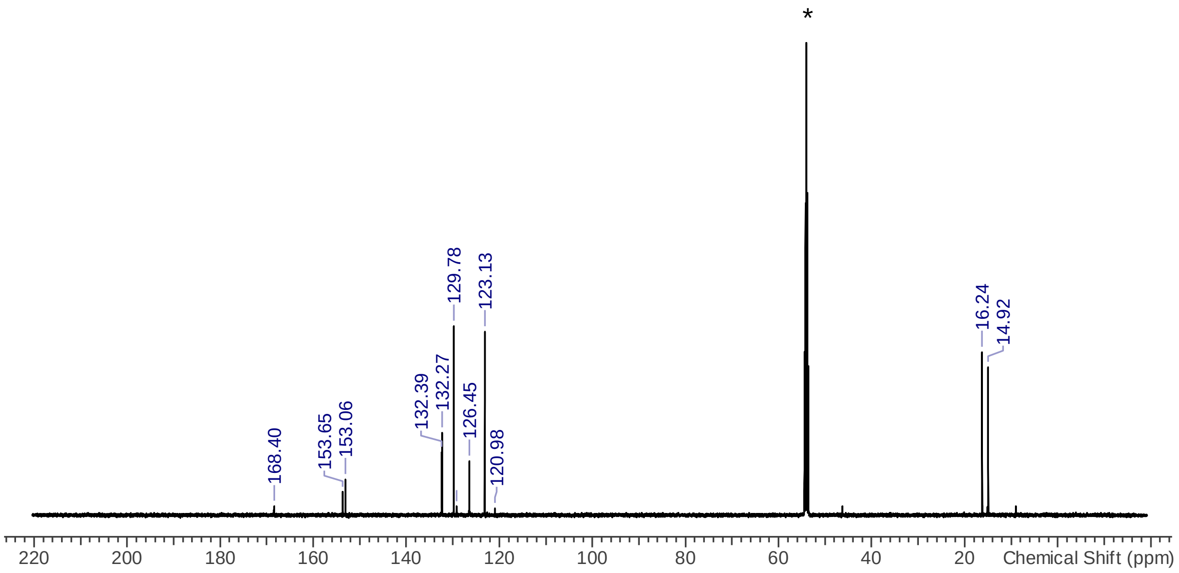

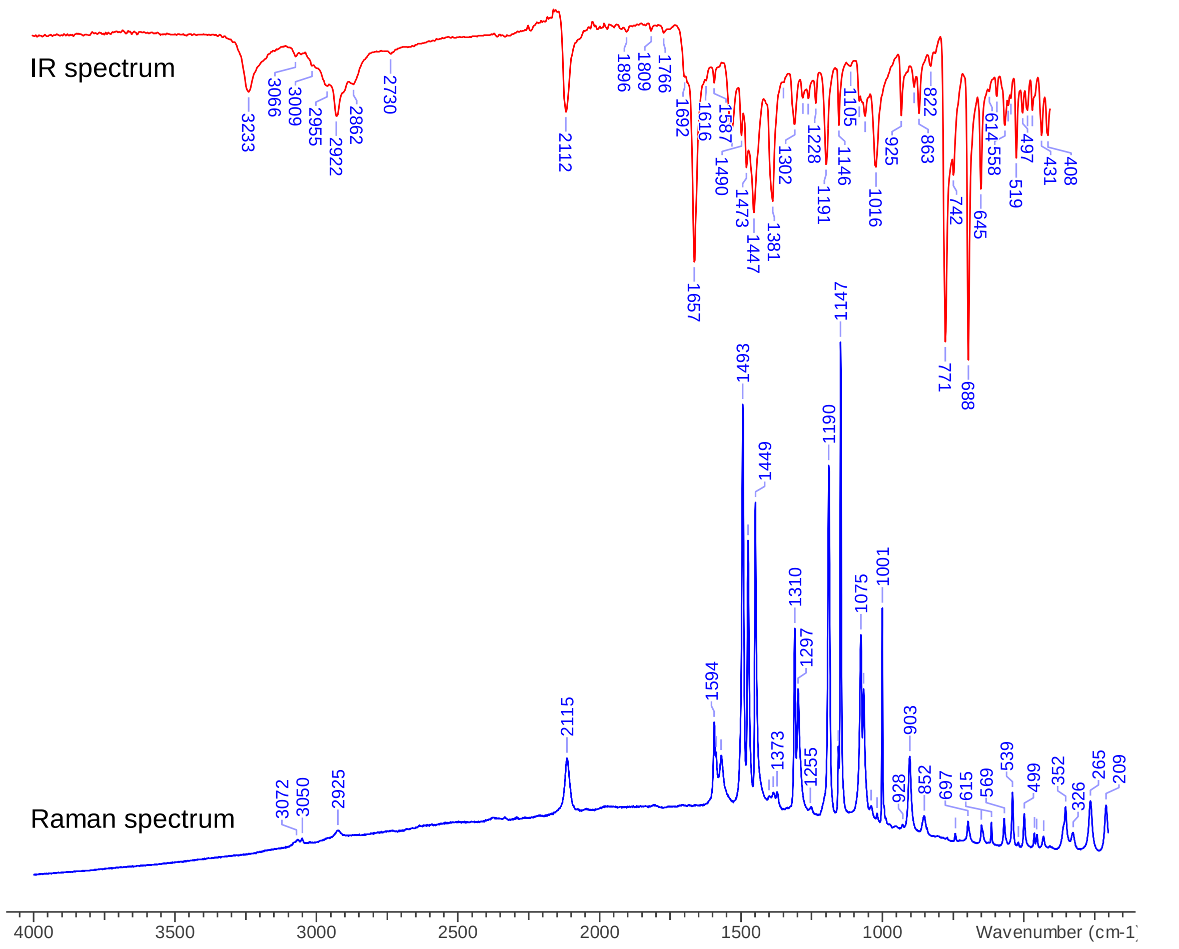

3.4 4-Amino-4-fluoro-2,3,5,6-tetramethyl-azobenzene (*E*-7)

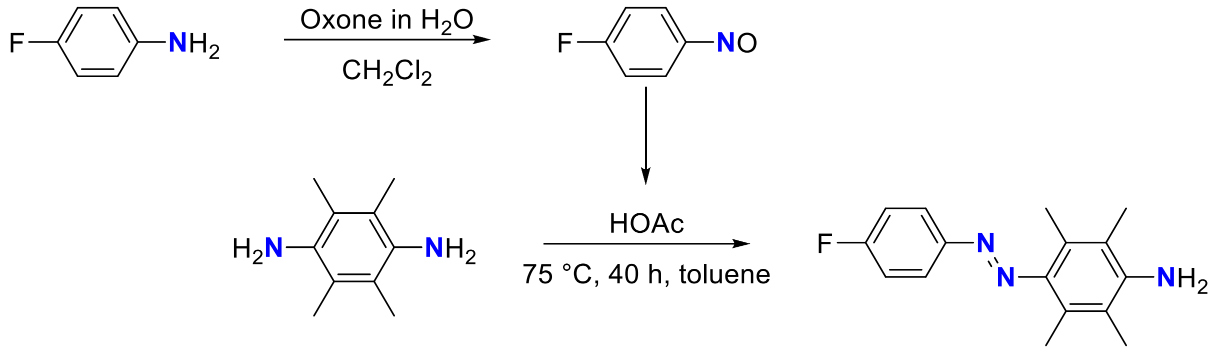

4-Amino-4-fluoro-2,3,5,6-tetramethyl-azobenzene was synthesised according to a modified literature procedure.[8] *The synthesis was carried out under non-inert conditions.*

Oxone (Potassium peroxymonosulfate, 32.1 g, 52.2 mmol) was dissolved in 120 mL of water and added dropwise to a stirred solution of 4-fluoroaniline (5.80 g, 52.2 mmol) in 120 mL of dichloromethane. The mixture was stirred for 1 h upon which the colour of the organic phase changed to an intense green. Afterwards the suspension was diluted with water (300 mL) and after separation of the phases the aqueous phase was extracted three times with CH_2Cl_2 (total amount of 300 mL). The solvent of the combined organic layers was removed using a rotary evaporator at 40 °C. The green oil of 4-fluoronitrosobenzene was dissolved in 20 mL of toluene and added to a stirred suspension of 2,3,5,6-tetramethyl-1,4-phenylenediamine (5.75 g, 35.0 mmol) in 100 mL of toluene whereupon HOAc (100%, 7.80 g, 130 mmol) was added. The mixture was stirred at 75 °C (oil bath) for further 35 h while a change of colour to brown occurred. The suspension was diluted with dichloromethane (150 mL) and after separation of the phases the organic phase was washed with water (200 mL). After repeated separation of the phases the aqueous phase was extracted three times with CH_2Cl_2 (total amount of 500 mL). The solvent of the combined organic layers was removed using a rotary evaporator (40 °C) and the crude product purified by column chromatography (petroleum ether/EtOAc 6:1). The solvent of the intensely red coloured organic phase was removed using a rotary evaporator yielding a red solid of 4-amino-4-fluoro-2,3,5,6-tetramethyl-azobenzene (***E*-7**). Yield: 1.01 g (3.72 mmol, 11%).

$C_{16}H_{18}N_3F$ (271.37 g/mol) **Mp**. 103.3 °C. **CHN** calc. (found) in %: C 70.82 (70.15), H 6.69 (6.43), N 15.49 (15.16). **^{1}H NMR** (25 °C, CD_2Cl_2, 300.1 MHz): δ = 2.15 (s, 6 H, C*H*$_3$), 2.30 (s, 6 H, C*H*$_3$), 3.90 (broad s, 2 H, N*H*$_2$), 7.15-7.24 (m, 2 H, *o*-C*H*), 7.81-7.90 (m, 2 H, *m*-C*H*). **^{13}C{^{1}H} NMR** (25 °C, CD_2Cl_2, 75.5 MHz): δ = 13.5 (s, *C*H$_3$), 15.9 (s, *C*H$_3$), 116.3 (d, *m*-*C*H, $^2J(^{13}C,^{19}F)$ = 23 Hz), 118.9 (s, quart. *C*), 124.5 (d, *o*-*C*H, $^3J(^{13}C,^{19}F)$ = 9 Hz), 129.0 (s, quart. *C*), 144.5 (s, quart. *C*), 145.0 (s, quart. *C*), 150.4 (d, *i*-*C*N, $^4J(^{13}C,^{19}F)$ = 3 Hz), 164.3 (d, *p*-*C*F, $^1J(^{13}C,^{19}F)$ = 249 Hz).**^{19}F NMR** (25 °C, CD_2Cl_2, 282.4 MHz): δ = −112.4 (s, *F*C). **IR** (ATR, 32 scans, cm^{-1}): $\tilde{\nu}$ = 3503 (m), 3417 (m), 2916 (m), 2856 (m), 1622 (m), 1591 (m), 1564 (m), 1496 (m), 1459 (m), 1442 (s), 1405 (m), 1379 (m), 1304 (s), 1261 (m), 1212 (s), 1179 (m), 1133 (m), 1117 (m), 1088 (m), 1039 (m), 1022 (m), 1008 (m), 983 (m), 843 (s), 837 (vs), 783 (m), 742 (m), 715 (m), 670 (m), 649 (m), 628 (m), 612 (m), 598 (m), 565 (m), 523 (s), 488 (m), 453 (s), 439 (s), 424 (s). **Raman** (633 nm, 10 s, 15 scans, cm^{-1}): $\tilde{\nu}$ = 3419 (1), 3069 (1), 1624 (1), 1595 (1), 1582 (1), 1570 (1), 1498 (1), 1463 (7), 1407 (5), 1384 (1), 1309 (5), 1285 (1), 1276 (1), 1264 (2), 1218 (1), 1181 (10), 1137 (5), 1120 (1), 1068 (3), 1024 (1), 1007 (1), 914 (1), 787 (1), 716 (1), 674 (1), 635 (1), 567 (1), 534 (1), 528 (1), 492 (1), 456 (1), 441 (1), 423 (1), 377 (1), 286 (1), 272 (1), 216 (1), 179 (1), 142 (1), 79 (1). **MS** (EI, 70 eV, m/z): 148 (87, $[M-FC_6H_4N_2]^+$); 149 (10); 255 (11, $[M-NH_2]^+$); 270 (46, $[M-H]^+$); 271 (100, $[M]^+$); 272 (17, $[M+H]^+$).

Figure S9: NMR, IR and Raman spectra of ***E*-7** (solvent signals indicated by asterisks).

^{1}H NMR spectrum

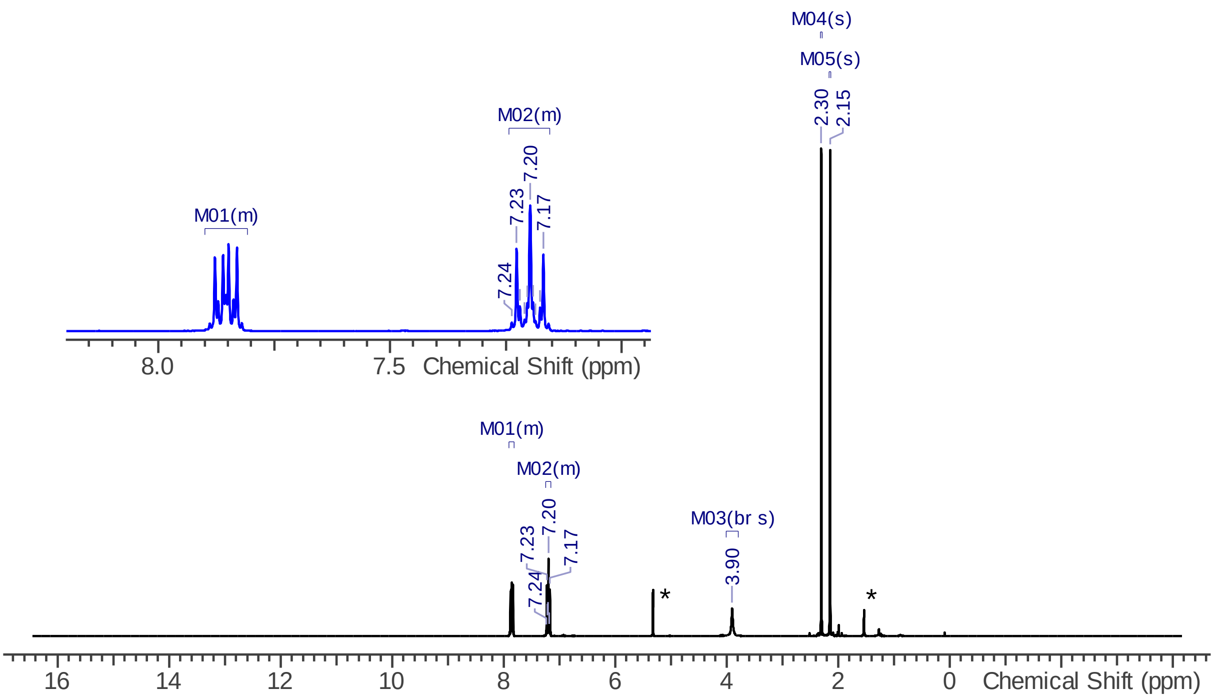

$^{13}C\{^{1}H\}$ NMR spectrum

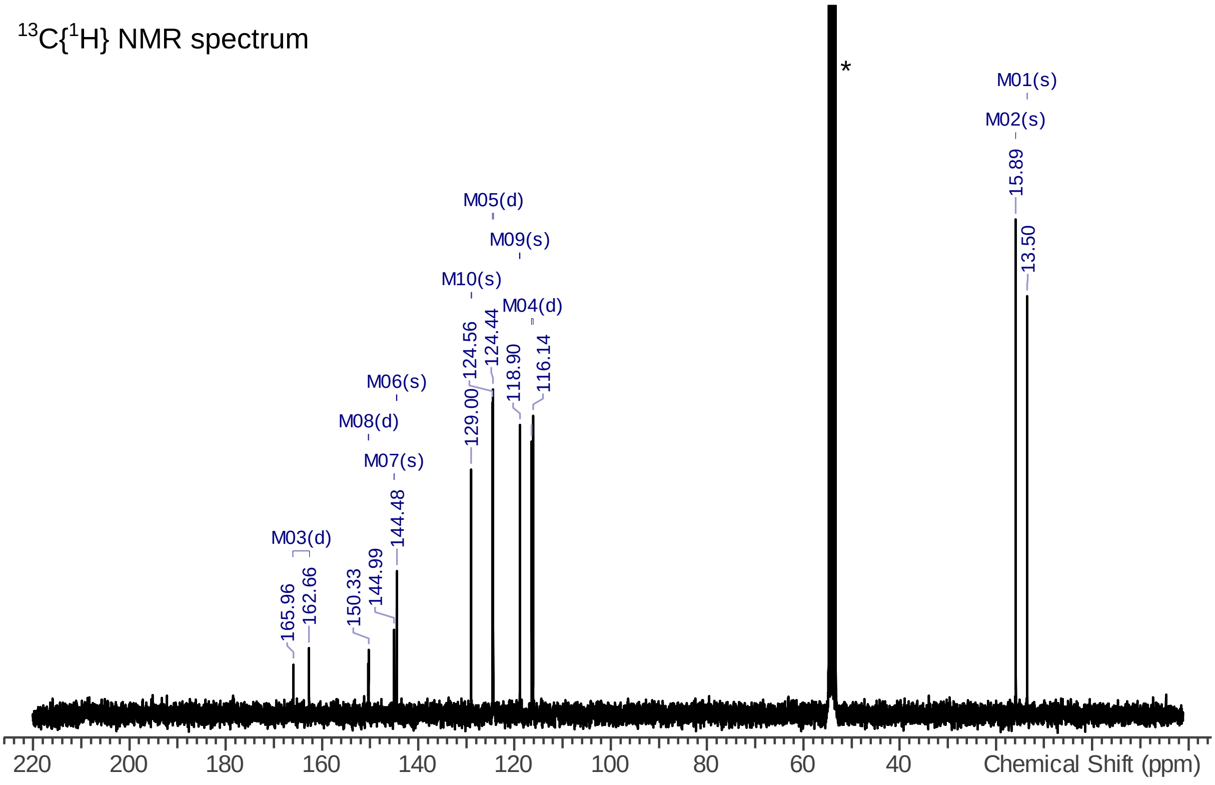

Figure S9 continued.

$^{19}F\{^1H\}$ NMR spectrum

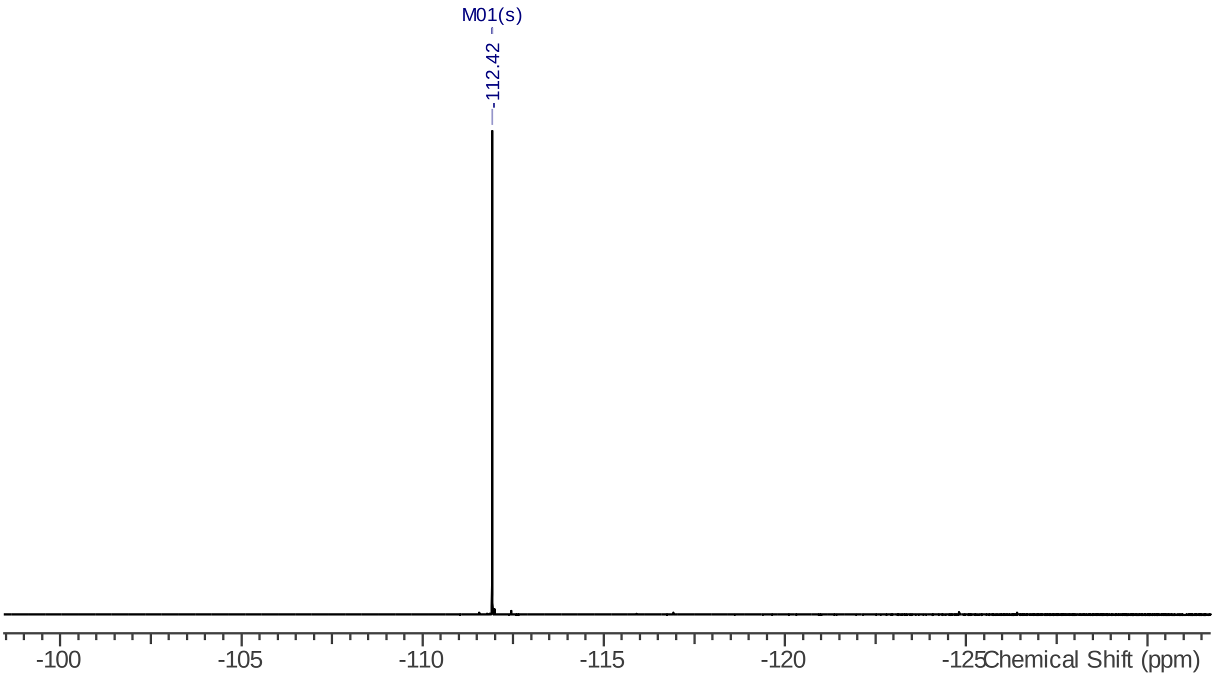

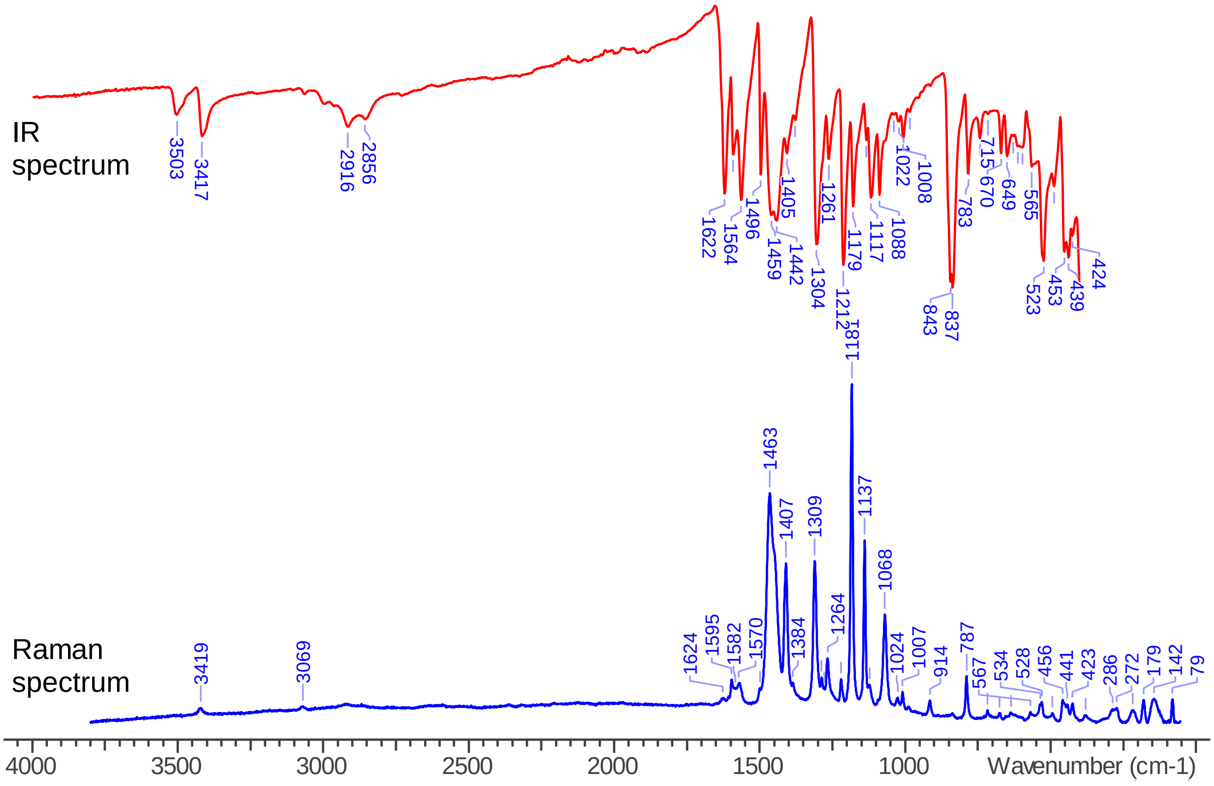

3.5 4-Formamido-2,3,5,6-tetramethyl-azo-4-fluorobenzene (*E*-9)

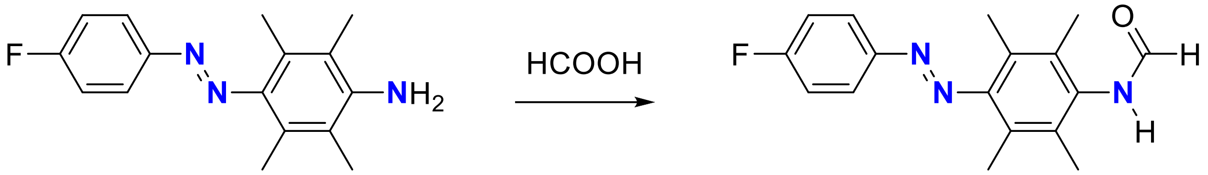

***E*-9** *was synthesised according to a modified literature procedure.*[9] *The synthesis was carried out under non-inert conditions.*

***E*-7** (1.0 g, 3.7 mmol) was dissolved in formic acid (10.0 g, 217 mmol) and degassed completely *via* two pump-freeze-thaw cycles. Afterwards the reaction mixture was stored in a drying oven at 60 °C for 70 h resulting in red needles. The crude product was washed three times with 25 mL of water and dried *in vacuo* (1×10^{-3} mbar, 100 °C, water bath). Yield: 0.95 g (3.2 mmol, 85 %). ***E*-9** is present in two sets of isomers (a and b) due to rotation around the N−CO bond.

$C_{17}H_{18}N_3FO$ (299 g/mol) **Mp**. 236.6 °C (decomposition). **CHN** calc. (found) in %: C 68.21 (67.92), H 6.06 (6.43), N 14.04 (12.63). **^{1}H NMR** (25 °C, CD_2Cl_2, 300.1 MHz): δ = 2.09 (s, 12 H, C*H*$_3$, (a)), 2.15-2.30 (m, 12 H, C*H*$_3$, (b)), 6.86-7.01 (m, 2 H, N*H*, (a+b)), 7.19-7.31 (m, 4 H, C*H,* (a)), 7.90-7.97 (m, 4 H, C*H,* (b)), 7.98 (m, 1 H, C*H*O, (a)), 8.43 (s, 1 H, C*H*O, (b)). **^{13}C{^{1}H} NMR** (25 °C, CD_2Cl_2, 75.5 MHz): δ = 15.1 (s, CH_3, (a+b)), 15.4 (s, CH_3, (a)), 15.6 (s, CH_3, (b)), 116.4-116.8 (br. d, *m*-CH, $^2J(^{13}C,^{19}F)$ = 23 Hz, (a)), 116.5-116.8 (br. d, *m*-CH, $^2J(^{13}C,^{19}F)$ = 23 Hz, (b)), 116.8 (s, quart. *C*, (a)), 117.2 (s, quart. *C*, (b)), 125.1 (d, *o*-CH, $^3J(^{13}C,^{19}F)$ = 9 Hz, (a)), 125.2 (d, *o*-CH, $^3J(^{13}C,^{19}F)$ = 9 Hz, (b)), 126.3 (s, quart. *C*, (a)), 126.6 (s, quart. *C*, (b)), 132.0 (s, quart. *C*, (a)), 132.3 (s, quart. *C*, (b)), 133.2 (s, quart. *C*, (a)), 133.6 (s, quart. *C*, (b)), 149.8 (d, *i*-CN, $^4J(^{13}C,^{19}F)$ = 3 Hz, (a)), 149.9 (d, *i*-CN, $^4J(^{13}C,^{19}F)$ = 3 Hz, (b)), 160.0 (s, *p*-CF) 165.3 (s, CHO).**^{19}F NMR** (25 °C, CD_2Cl_2, 282.4 MHz): δ = −109.6 (s, *F*C), −110.0 (s, *F*C). **IR** (ATR, 32 scans, cm^{-1}): $\tilde{\nu}$ = 3210 (m), 3169 (w), 2990 (w), 2908 (m), 1651 (vs), 1591 (m), 1517 (m), 1498 (s), 1475 (m), 1442 (m), 1403 (m), 1381 (m), 1300 (m), 1259 (w), 1222 (s), 1197 (m), 1131 (m), 1088 (m), 1074 (m), 1059 (m), 1039 (w), 1022 (m), 1006 (m), 886 (m), 841 (s), 820 (m), 810 (m), 783 (m), 734 (m), 668 (m), 641 (m), 620 (w), 563 (m), 523 (m), 447 (m), 427 (w), 406 (m). **Raman**

(633 nm, 10 s, 20 scans, cm^{-1}): $\tilde{\nu}$ = 3082 (1), 3064 (1), 2922 (1), 2917 (1), 1606 (1), 1594 (1), 1572 (1), 1501 (3), 1480 (10), 1444 (1), 1426 (1), 1410 (2), 1389 (1), 1376 (1), 1310 (2), 1302 (2), 1258 (1), 1240 (1), 1224 (2), 1183 (7), 1166 (1), 1158 (1), 1135 (5), 1090 (1), 1074 (4), 1046 (1), 1037 (1), 1026 (1), 965 (1), 958 (1), 951 (1), 941 (1), 913 (1), 871 (1), 867 (1), 860 (1), 857 (1), 850 (1), 847 (1), 843 (1), 820 (1), 733 (1), 726 (1), 718 (1), 712 (1), 704 (1), 648 (1), 638 (1), 633 (1), 630 (1), 620 (1), 575 (1), 561 (1), 532 (1), 527 (1), 502 (1), 485 (1), 447 (1), 424 (1), 368 (1), 364 (1), 354 (1), 300 (1), 206 (2), 175 (1), 90 (1).
MS (EI, 70 eV, m/z): 77 (23); 91 (61); 95 (91, $[FC_6H_4]^+$); 96 (21); 105 (34); 115 (22); 117 (27); 119 (21); 123 (40); 131 (77); 132 (62, $[C_6Me_4]^+$); 133 (59); 134 (47); 146 (30); 147 (21); 148 (91); 149 (60); 159 (28); 160 (32); 161 (21); 164 (47); 176 (99, $[M-FC_6H_4N_2]^+$); 177 (80); 253 (29); 254 (33); 255 (45); 270 (40); 271 (28); 298 (99, $[M-H]^+$); 299 (100, $[M]^+$); 300 (95, $[M+H]^+$).

Figure S10: NMR, IR and Raman spectra of ***E*-9** (solvent signals indicated by asterisks).

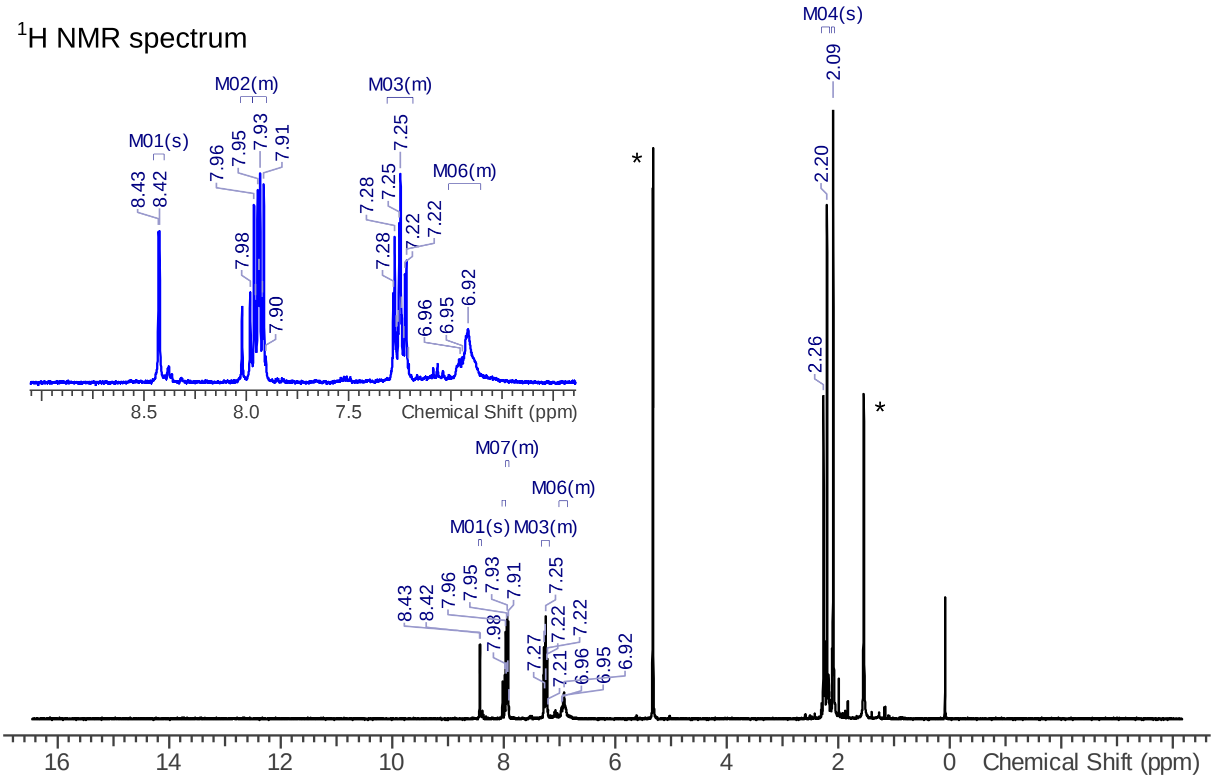

Figure S10 continued.

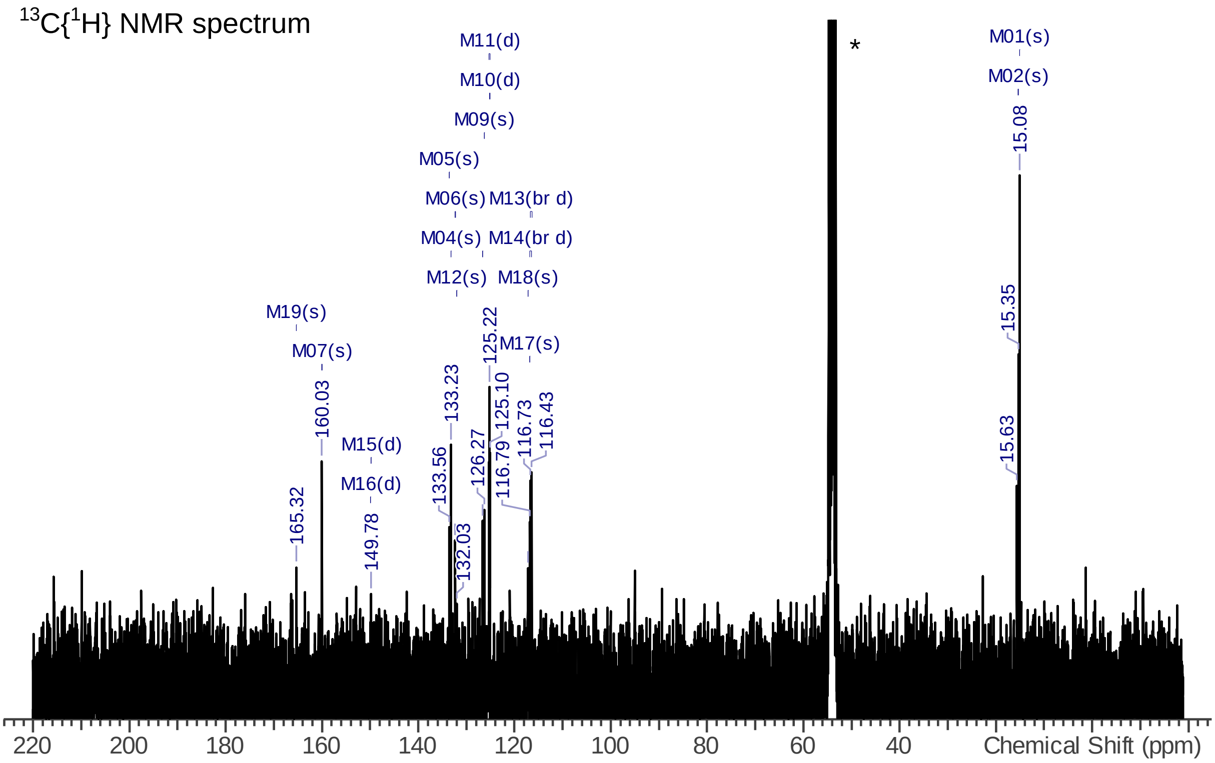

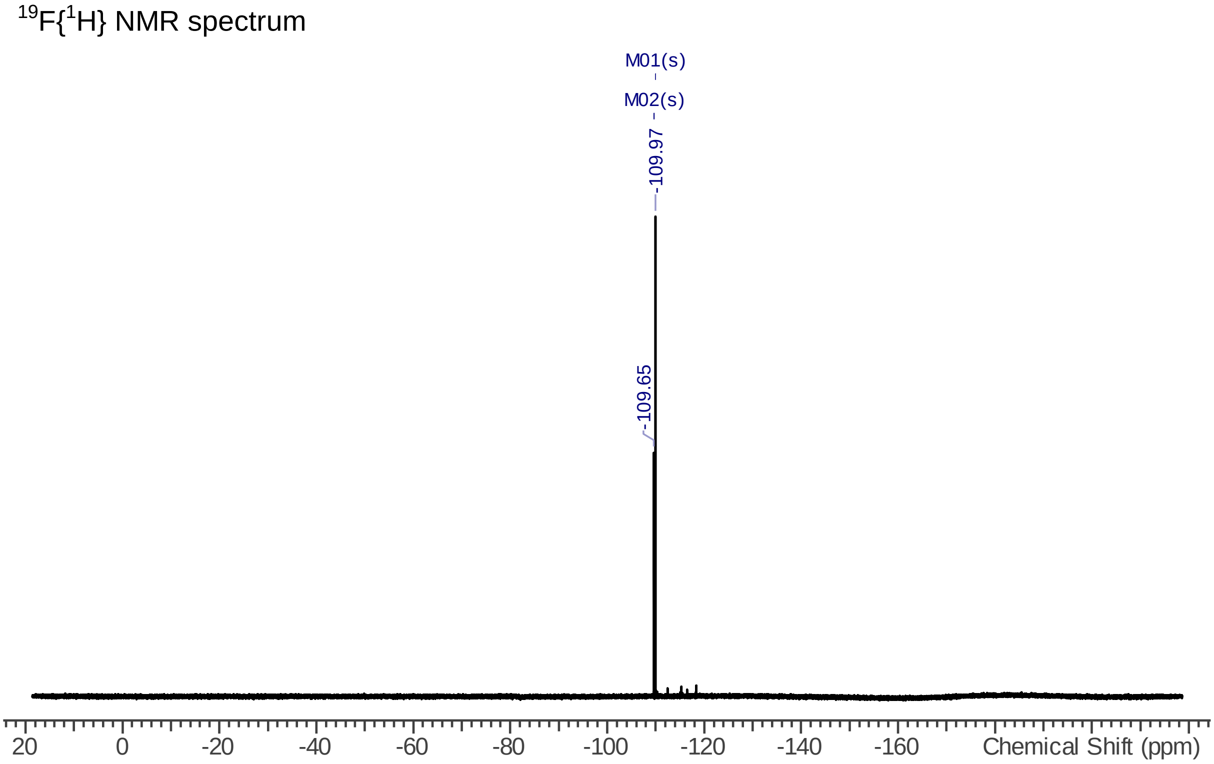

Figure S10 continued.

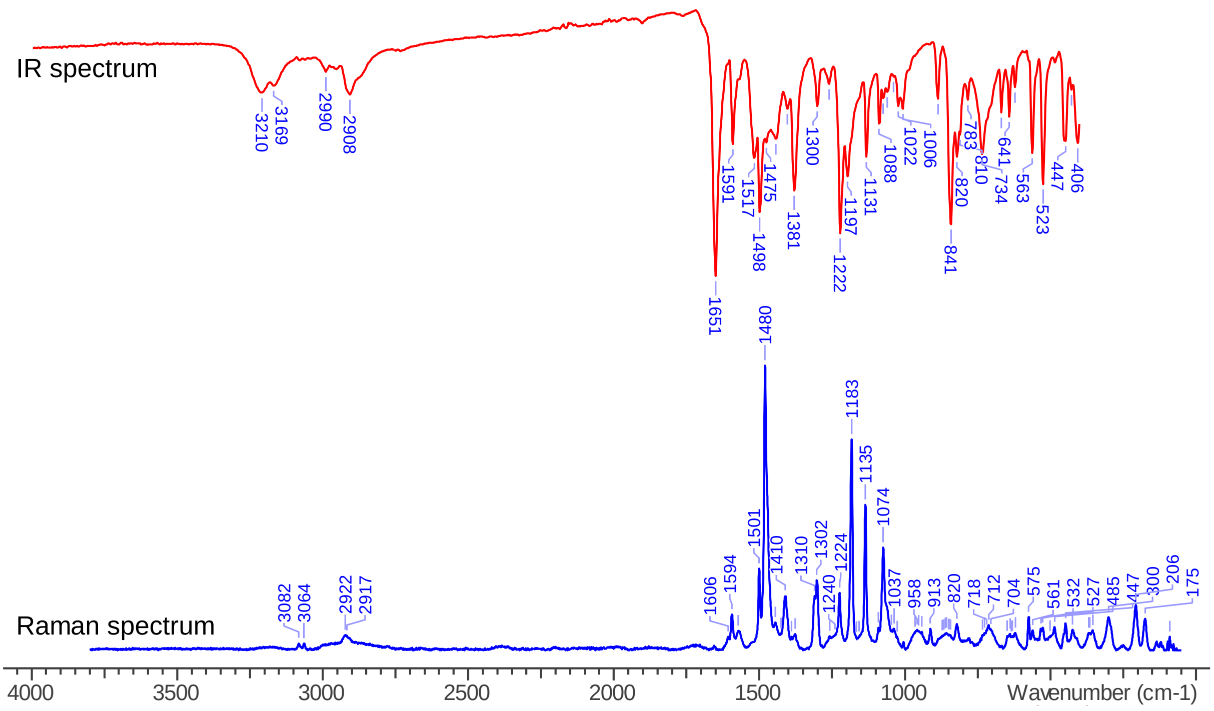

3.6 4-Isocyano-2,3,5,6-tetramethyl-azo-4-fluorobenzene (*E*-11)

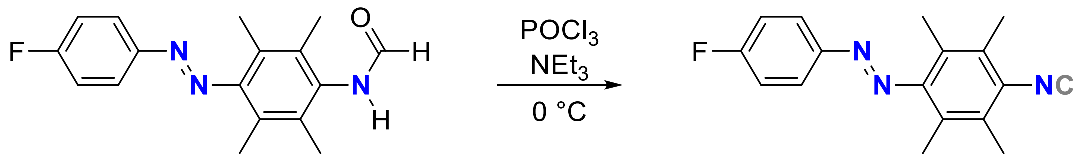

***E*-11** *was synthesised according to a modified literature procedure.*[9]

***E*-9** (0.95 g, 3.2 mmol) was dissolved in 10 mL of CH_2Cl_2. Approximately two equivalents of phosphoryl trichloride (1.00 g, 6.52 mmol) were added under stirring at 0 °C followed by the dropwise addition of ten equivalents of NEt_3 (3.24 g, 32.0 mmol). The reaction mixture was stirred for 48 h at room temperature, turning brownish and becoming cloudy in the process. Afterwards the brown solution was diluted in 100 mL of ice-water. The aqueous phase was extracted three times with CH_2Cl_2 (total amount of 90 mL). The combined organic layers were extracted two times with 50 mL water and

two times with 50 mL of a saturated $NaHCO_3$ solution. After separation of the phases the solvent of the organic layer was removed *in vacuo* (1×10^{-3} mbar, 30 °C, water bath). The crude product was purified by sublimation *in vacuo* (1×10^{-3} mbar, 150 °C, heat gun) yielding a red solid of ***E*-11**. Yield: 0.30 g (1.3 mmol, 41 %).

$C_{17}H_{16}N_3F$ (281 g/mol) **Mp**. 103.4 °C. **CHN** calc. (found) (x 0.5 C_6H_6) in %: C 74.98 (73.79), H 5.98 (6.01), N 13.12 (11.53). **^{1}H NMR** (25 °C, THF-d_8, 300.1 MHz): δ = 1.97 (s, 2 H, C*H*$_3$), 2.10 (s, 6 H, C*H*$_3$), 2.23 (s, 2 H, C*H*$_3$), 2.29 (s, 2 H, C*H*$_3$), 2.40 (s, 6 H, C*H*$_3$), 7.25-7.39 (m, 2 H, *o*-C*H*), 7.89-8.03 (m, 2 H, *m*-C*H*). **^{13}C{^{1}H} NMR** (25 °C, THF-d_8, 75.5 MHz): δ = 12.2 (s, *C*H$_3$), 14.8 (s, *C*H$_3$), 15.5 (s, *C*H$_3$), 16.0 (s, *C*H$_3$), 19.9 (s, *C*H$_3$), 117.0 (d, *m*-*C*H, $^2J(^{13}C,^{19}F)$ = 23 Hz), 125.7 (d, *o*-*C*H, $^3J(^{13}C,^{19}F)$ = 9 Hz), 127.1 (s, quart. *C*), 131.1 (s, quart. *C*), 132.5 (s, quart. *C*), 140.9 (s, quart. *C*), 150.3 (d, *i*-*C*N, $^4J(^{13}C,^{19}F)$ = 3 Hz), 153.7 (s, quart. *C*), 166.0 (d, *p*-*C*F, $^1J(^{13}C,^{19}F)$ = 251 Hz), 170.9 (s, N*C*).**^{19}F NMR** (25 °C, THF-d_8, 282.4 MHz): δ = −109.8 (s, *F*C). **IR** (ATR, 32 scans, cm^{-1}): $\tilde{\nu}$ = 3066 (w), 2992 (w), 2924 (w), 2737 (w), 2108 (m, NC), 1896 (w), 1758 (w), 1677 (w), 1638 (w), 1595 (m), 1500 (s), 1484 (m), 1453 (m), 1414 (w), 1383 (m), 1306 (w), 1284 (w), 1222 (s), 1135 (m), 1092 (m), 1057 (m), 1022 (m), 1008 (m), 956 (w), 942 (w), 907 (w), 837 (vs), 783 (m), 738 (w), 703 (w), 672 (m), 653 (m), 645 (m), 626 (w), 591 (w), 573 (w), 556 (w), 544 (w), 521 (s), 499 (w), 455 (w), 443 (m), 422 (m). **Raman** (633 nm, 10 s, 20 scans, cm^{-1}): $\tilde{\nu}$ = 3076 (1), 2929 (1), 2113 (3, NC), 1598 (3), 1570 (3), 1504 (5), 1483 (10), 1451 (3), 1414 (4), 1309 (4), 1301 (4), 1223 (4), 1185 (7), 1140 (6), 1070 (5), 959 (2), 911 (2), 865 (2), 826 (2), 784 (2), 716 (1), 627 (1), 573 (1), 544 (1), 529 (1), 504 (1), 347 (1), 328 (1), 201 (1), 166 (1). **MS** (EI, 70 eV, m/z): 51 (22); 65 (22); 75 (52); 77 (38); 91 (58); 95 (100, $[FC_6H_4]^+$); 96 (37); 103 (24); 115 (75); 116 (75); 117 (32); 123 (76, $[FC_6H_4N_2]^+$); 128 (26); 130 (44); 131 (23); 142 (46); 143 (82); 144 (36); 156 (23); 157 (44); 158 (95, $[M-FC_6H_4N_2]^+$); 159 (71); 186 (45, $[M-FC_6H_4]^+$); 264 (29); 265 (27); 279 (30); 280 (95, $[M-H]^+$); 281 (98, $[M]^+$); 282 (82, $[M+H]^+$).

Figure S11: NMR, IR and Raman spectra of ***E*-11** (solvent signals indicated by asterisks).

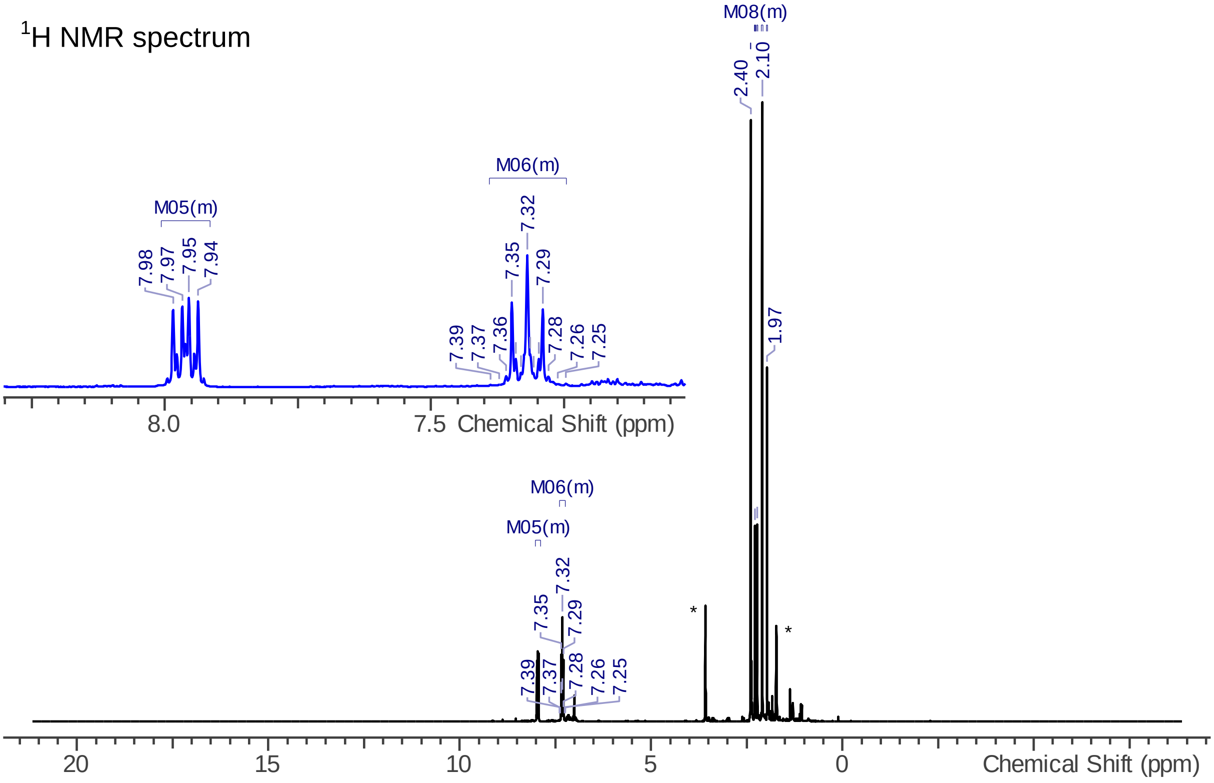

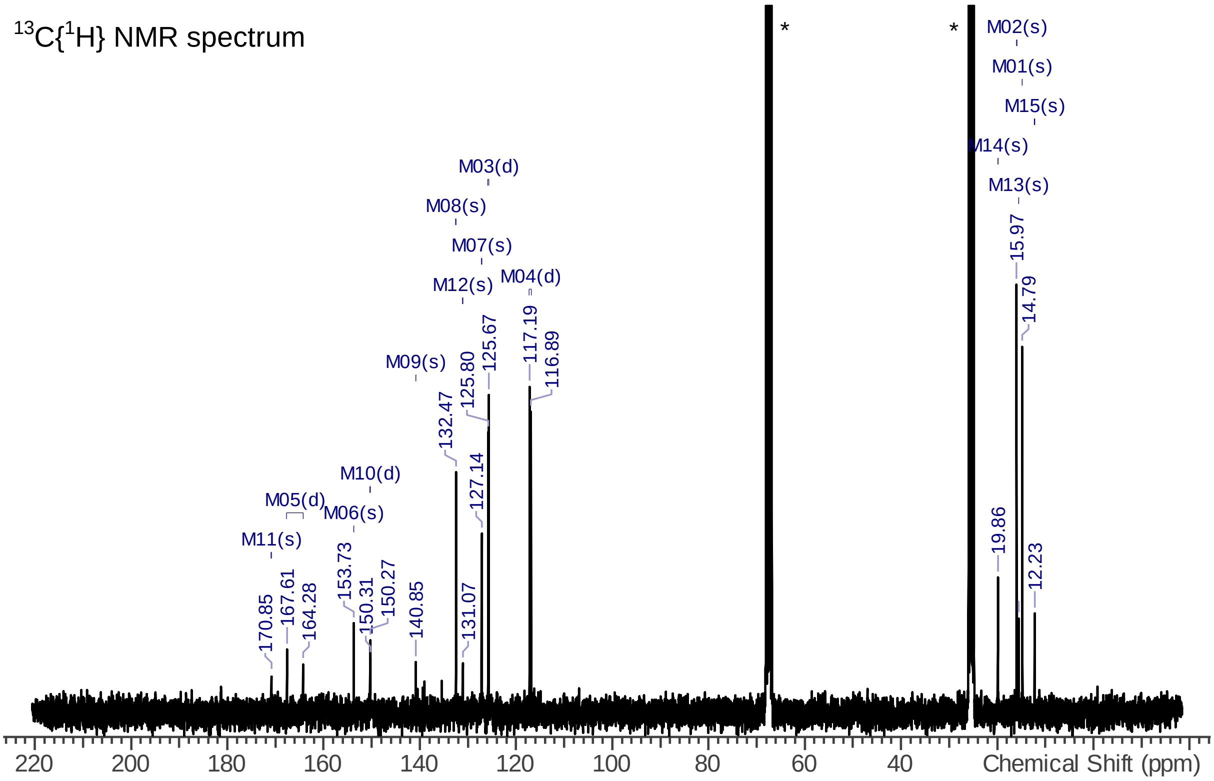

Figure S11 continued.

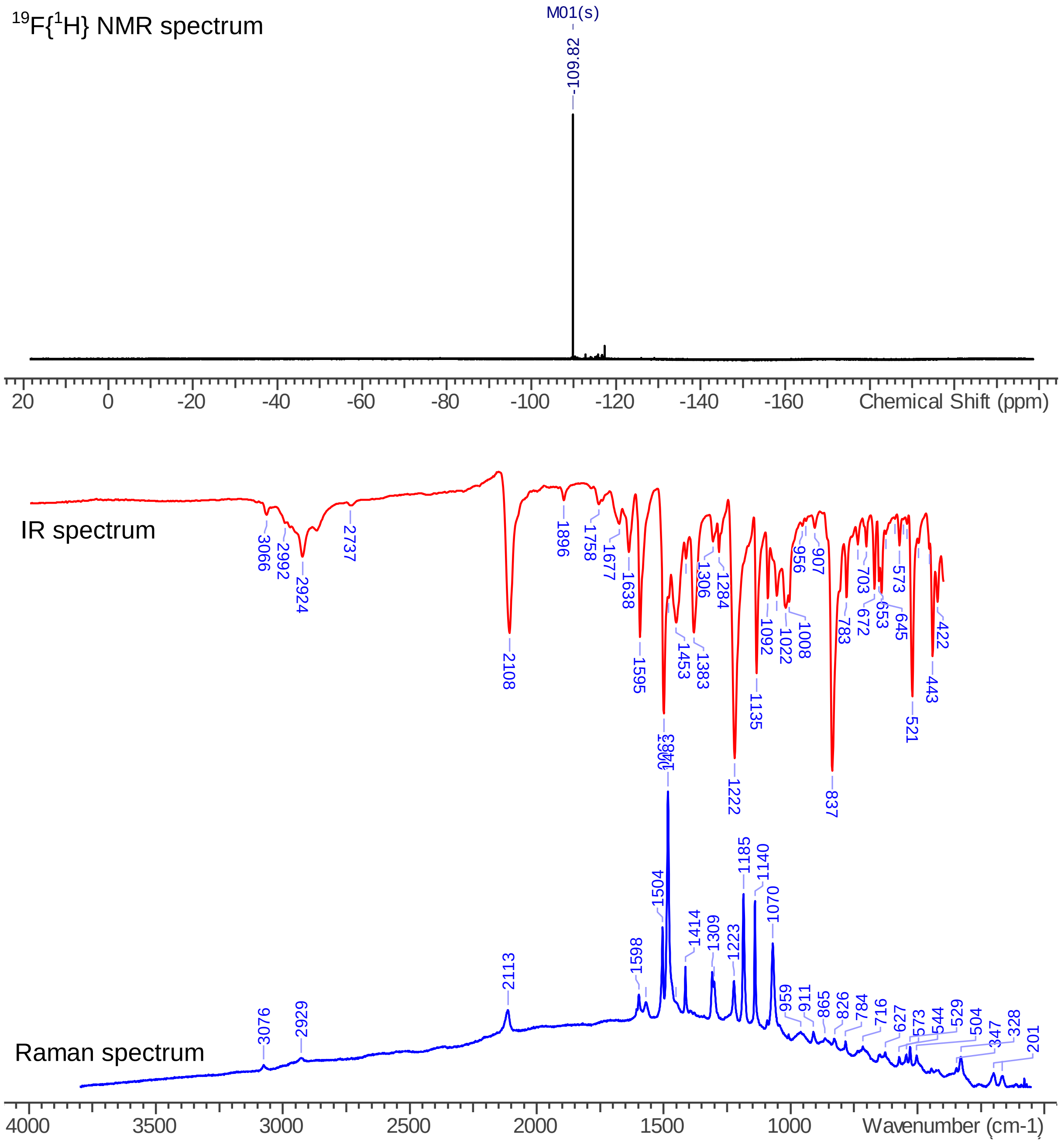

4 Syntheses of compounds

4.1 *E*-4B ([P(*μ*-NTer)]$_2$ + *E*-10)

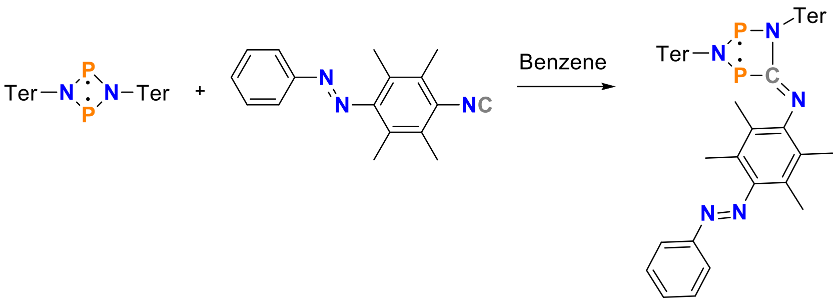

Attention: To prevent housane formation the reaction must be carried out in the dark, thoroughly covering the reaction vessels with aluminium foil!

Biradical [P(*μ*-NTer)]$_2$ (**1**, 0.18 g, 0.25 mmol) and ***E*-10** (0.066 g, 0.25 mmol) were added together in a glass vial inside an argon filled drybox and dissolved in 10 mL of benzene. The colour of the solution turned from red to an intense blue/black immediately and the mixture was stirred for 30 min at room temperature with a glass stir bar. Subsequently, the solvent of the solution was removed *in vacuo* (1×10^{-3} mbar, 50 °C, water bath) yielding product ***E*-4B** as a blue/black solid. Yield: 0.221 g (0.225 mmol, 90%).

Compound ***E*-4B** should be stored in the dark in an argon filled drybox inside a sealed glass tube at low temperatures (−20 °C). Single crystals suitable for X-ray structure elucidation can be grown in an argon filled drybox from a freshly filtered (syringe filter, 0.2 µm pore size) saturated benzene solution at ambient temperature.

$C_{65}H_{67}N_5P_2$ (980.23 g/mol) **Mp**. 203 °C (decomp.). **CHN** calc. (found) in % (deviations due to extreme sensitivity of compound towards moisture): C 79.65 (76.04), H 6.89 (6.45), N 7.14 (6.52). **^{1}H NMR** (25 °C, C_6D_6, 500.1 MHz): δ = 1.64 (s, 6 H, *Azo*-C*H*$_3$), 1.74 (s, 6 H, *Azo*-C*H*$_3$), 1.96 (s, 12 H, Mes-*o*-C*H*$_3$), 2.28 (s, 6 H, *p*-C*H*$_3$), 2.31 (s, 12 H, Mes-*o*-C*H*$_3$), 2.35 (s, 6 H, *p*-C*H*$_3$), 6.64-7.03 (m, 19 H, arom. C*H*).**^{13}C{^{1}H} NMR** (25 °C, C_6D_6,

125.8 MHz): δ = 14.9 (s, CH_3), 15.8 (s, CH_3), 15.8 (s, CH_3), 16.0 (s, CH_3), 16.4 (s, CH_3), 20.7 (s, CH_3), 21.2 (s, CH_3), 21.3 (s, CH_3), 21.4 (s, CH_3), 21.5 (s, CH_3), 21.9 (s, CH_3), 22.0 (s, CH_3), 123.1 (s, arom. *C*H), 123.3 (s, arom. *C*H), 126.6 (s, arom. *C*H), 128.3 (s, arom. *C*H), 128.5 (s, arom. *C*H), 128.7 (s, arom. *C*H), 128.8 (s, arom. *C*H), 128.9 (s, arom. *C*H), 129.0 (s, arom. *C*H), 129.1 (s, arom. *C*H), 129.2 (s, arom. *C*H), 129.2 (s, arom. *C*H), 129.5 (s, arom. *C*H), 129.9 (s, arom. *C*H), 130.2 (s, arom. *C*H), 130.3 (s, arom. *C*H), 131.0 (s, arom. *C*H), 131.6 (s, arom. *C*H), 130.2 (s, arom. *C*H), 132.1 (s, arom. *C*H), 132.3 (s, arom. *C*H), 132.5 (s, arom. *C*H), 135.1 (s, quart. *C*), 135.2 (s, quart. *C*), 136.0 (s, quart. *C*), 136.6 (s, quart. *C*), 137.2 (s, quart. *C*), 137.3 (s, quart. *C*), 137.6 (s, quart. *C*), 138.2 (s, quart. *C*), 139.6 (s, quart. *C*), 139.6 (s, quart. *C*), 140.8 (s, quart. *C*), 140.8 (s, quart. *C*), 148.1 (s, quart. *C*), 151.5 (s, quart. *C*), 151.6 (s, quart. *C*), 153.4 (s, quart. *C*), 154.4 (s, quart. *C*), 176.5 (s, N*C*P). **$^{31}P\{^1H\}$ NMR** (25 °C, C_6D_6, 101.3 MHz): δ = 221.0 (d, 1 P, N*PC*, $^2J(^{31}P,^{31}P)$ = 127 Hz), 257.0 (d, 1 P, N*P*N, $^2J(^{31}P,^{31}P)$ = 127 Hz). **IR** (ATR, 32 scans, cm^{-1}): $\tilde{\nu}$ = 2994 (w), 2953 (m), 2916 (m), 2856 (w), 2728 (w), 1714 (vw), 1632 (m), 1609 (m), 1558 (m), 1447 (s), 1405 (m), 1377 (m), 1267 (m), 1234 (vs), 1191 (s), 1164 (m), 1148 (m), 1109 (m), 1090 (m), 1018 (m), 1006 (m), 989 (m), 964 (s), 880 (s), 845 (vs), 802 (s), 767 (s), 750 (s), 711 (s), 688 (s), 674 (s), 620 (m), 598 (m), 567 (s), 538 (m), 528 (m), 511 (m), 499 (m), 482 (s), 462 (s), 449 (s). **Raman** (633 nm, 10 s, 10 scans, cm^{-1}): $\tilde{\nu}$ = 3055 (1), 3007 (1), 2918 (1), 2854 (1), 2727 (1), 1633 (2), 1612 (2), 1596 (2), 1586 (2), 1559 (3), 1487 (6), 1472 (10), 1447 (9), 1391 (1), 1381 (2), 1305 (7), 1264 (2), 1247 (1), 1193 (6), 1153 (5), 1092 (2), 1072 (3), 1063 (4), 1035 (1), 1019 (1), 1000 (3), 955 (1), 940 (2), 900 (1), 852 (1), 843 (1), 756 (1), 739 (1), 702 (1), 680 (1), 671 (1), 648 (1), 623 (1), 611 (1), 605 (1), 578 (2), 552 (2), 542 (1), 530 (1), 522 (1), 511 (1), 494 (1), 489 (1), 480 (1), 474 (1), 445 (1), 414 (1), 390 (1), 341 (1), 267 (1), 258 (1), 229 (1), 216 (1). **MS** (EI, 70 eV, m/z): 352 (100; $[M-Ter_2H]^+$); 598 (1; $[M-Mes-Isonitrile]^+$).

Figure S12: NMR, IR and Raman spectra of ***E*-4B** (solvent signals indicated by asterisks).

$^{31}P\{^1H\}$ NMR spectrum

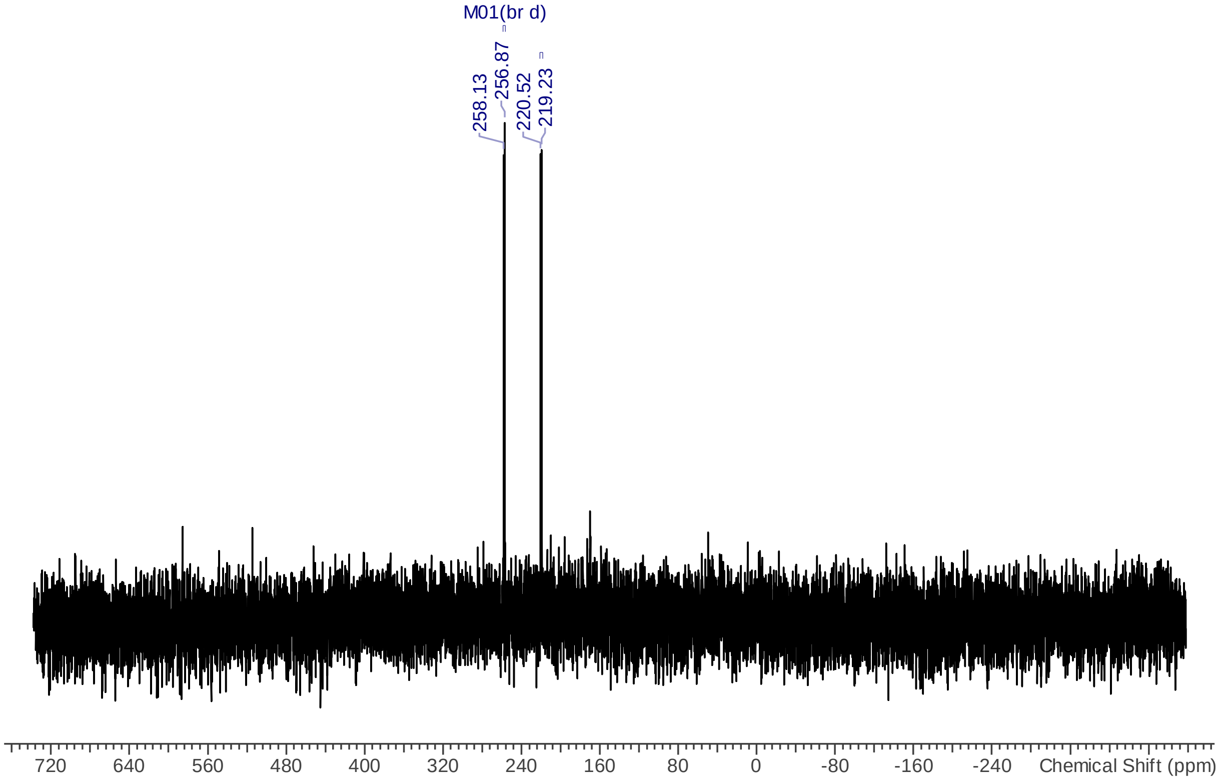

^1H NMR spectrum

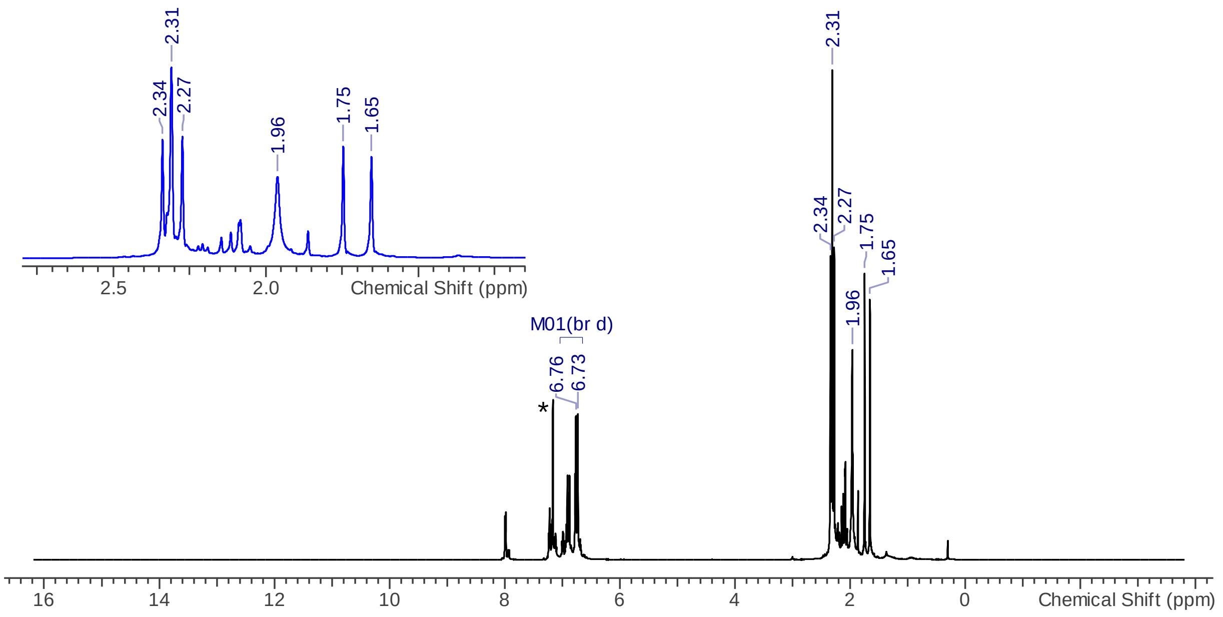

Figure S12 continued.

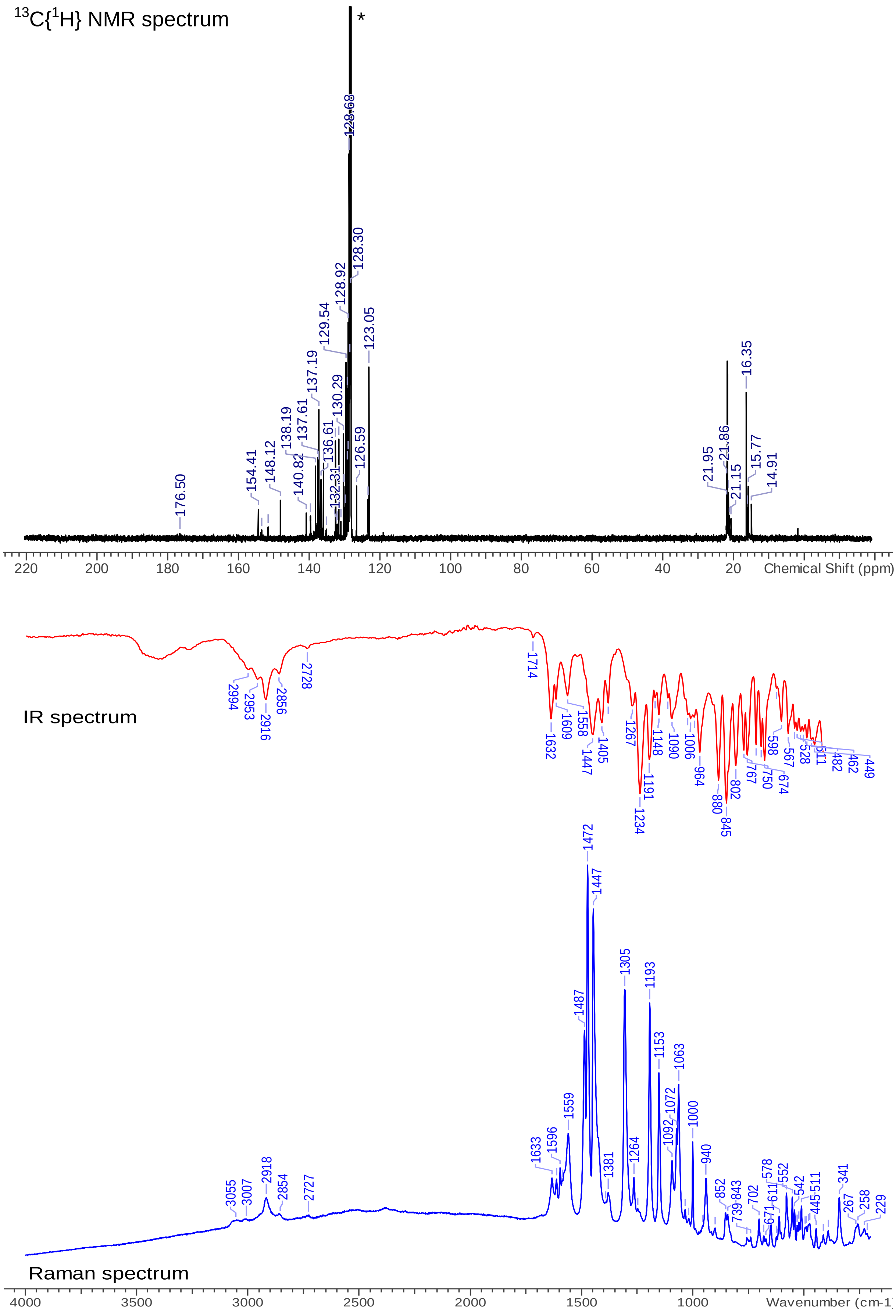

4.2 *E*-5B ([P(*µ*-NTer)]$_2$ + *E*-11)

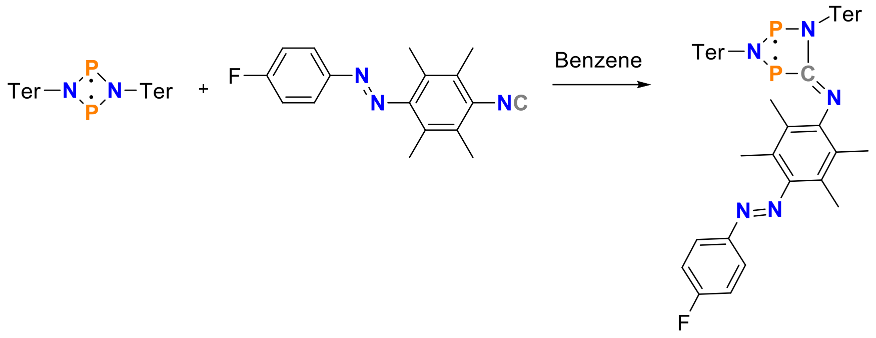

Attention: To prevent housane formation the reaction must be carried out in the dark, thoroughly covering the reaction vessels with aluminium foil!

Biradical (**1**) [P(*µ*-NTer)]$_2$ (0.18 g, 0.25 mmol) and ***E*-11** (0.071 g, 0.25 mmol) were added together in a glass vessel inside an argon filled drybox and dissolved in 10 mL of benzene. The colour of the solution turned from red to an intense blue/black immediately and the mixture was stirred for 30 min at room temperature with a glass stir bar. Subsequently, the solvent of the solution was removed *in vacuo* (1×10^{-3} mbar, 50 °C, water bath) yielding product ***E*-5B** as a blue/black solid. Yield: 0.23 g (0.23 mmol, 92%).

Compound ***E*-5B** should be stored in the dark in an argon filled drybox inside a sealed glass tube at low temperatures (−20 °C). Single crystals suitable for X-ray structure elucidation can be grown in an argon filled drybox from a freshly filtered saturated *n*-hexane solution (syringe filter, 0.2 µm pore size) at ambient temperature. Compound ***E*-5B** is present in two sets of isomers (I^A and I^B).

$C_{65}H_{66}FN_5P_2$ (998 g/mol) **Mp**. 200.1 °C. **CHN** calc. (found) in %: C 78.21 (78.14), H 6.66 (6.59), N 7.02 (5.73). **$^{31}P\{^1H\}$ NMR** (25 °C, THF-*d*$_8$, 202.5 MHz): δ = 219.7 (br. d, 1P, $^2J(^{31}P,^{31}P)$ = 127 Hz, I^A-N*P*C), 222.0 (br. d, 1P, $^2J(^{31}P,^{31}P)$ = 136 Hz, I^B-N*P*C), 257.5 (br. d, 1P, $^2J(^{31}P,^{31}P)$ = 127 Hz, I^A-N*P*N), 258.8 (br. d, 1P, $^2J(^{31}P,^{31}P)$ = 136 Hz, I^B-N*P*N). **^1H NMR** (25 °C, THF-*d*$_8$, 500.1 MHz): δ = 1.16-1.32 (m, 6 H, *Azo*-C*H*$_3$), 1.57 (m, 6 H, Mes-*p*-C*H*$_3$), 1.82 (m, 12 H, Mes-*o*-C*H*$_3$), 1.98 (s, 6 H, Mes-*p*-C*H*$_3$), 2.13 (s, 6 H, *Azo*-C*H*$_3$), 2.34 (m, 12 H, Mes-*o*-C*H*$_3$), 6.67-6.81 (m, 8 H, Ter-C*H*), 6.90-6.98 (m, 2 H, Ter-C*H*), 7.01-7.07 (m,

2 H, Ter-C*H*), 7.22 (t, 2 H, FC(C*H*)$_2$), 7.33 (m, 1 H, Ter-C*H*), 7.39-7.47 (m, 1 H, Ter-C*H*), 7.83 (m, 2 H, N_2C(C*H*)$_2$). **^{13}C{^{1}H} NMR** (25 °C, THF-d_8, 125.8 MHz): δ = 15.0 (d, I^B-*Azo*-*C*H$_3$), 15.6 (d, I^A-*Azo*-*C*H$_3$), 16.1 (s, *Azo*-*C*H$_3$), 20.7 (s, Ter-*C*H$_3$), 21.4 (br. d, Ter-*C*H$_3$), 21.6 (s, Ter-*C*H$_3$), 21.7 (s, Ter-*C*H$_3$), 21.8 (br. d, Ter-*C*H$_3$), 116.6 (d, FC(*C*H)$_2$, $^2J(^{13}C,^{19}F)$ = 23 Hz), 124.9 (d, I^A-N_2C(*C*H)$_2$, $^3J(^{13}C,^{19}F)$ = 9 Hz), 125.5 (d, I^B-N_2C(*C*H)$_2$, $^3J(^{13}C,^{19}F)$ = 9 Hz), 126.8 (s, quart. *C*), 128.4 (s, Ter-*C*H), 128.5 (m, Ter-*C*H), 128.9 (s, Ter-*C*H), 129.3 (s, Ter-*C*H), 129.5 (s, Ter-*C*H), 129.7 (s, Ter-*C*H), 130.3 (s, Ter-*C*H), 132.2 (s, Ter-*C*H), 132.9 (s, Ter-*C*H), 133.7 (s, quart. *C*), 136.3 (m, quart. *C*), 137.1 (m, quart. *C*), 137.4 (s, quart. *C*), 137.6 (s, quart. *C*), 137.8 (s, quart. *C*), 138.0 (s, quart. *C*), 138.2 (m, quart. *C*), 139.8 (br. s, quart. *C*), 141.1 (br. s, quart. *C*), 147.4 (s, quart. *C*), 151.2 (d, *i*-*C*N, $^4J(^{13}C,^{19}F)$ = 3 Hz), 164.9 (d, *p*-*C*F, $^1J(^{13}C,^{19}F)$ = 249 Hz).**^{19}F{^{1}H} NMR** (25 °C, THF-d_8, 470.6 MHz): δ = −112.3 (s, *F*C). **IR** (ATR, 32 scans, cm^{-1}): $\tilde{\nu}$ = 3032 (w), 2997 (w), 2943 (w), 2914 (m), 2855 (w), 1610 (w), 1593 (w), 1571 (w), 1515 (s), 1480 (m), 1449 (m), 1404 (m), 1375 (m), 1288 (m), 1220 (s), 1195 (m), 1165 (m), 1136 (m), 1119 (m), 1084 (m), 1062 (m), 1033 (m), 1002 (m), 954 (w), 940 (w), 897 (w), 843 (s), 802 (m), 785 (m), 750 (m), 715 (w), 693 (m), 674 (vs), 651 (m), 604 (m), 563 (m), 550 (m), 521 (m), 511 (m), 470 (m), 441 (w), 408 (m). **Raman** (633 nm, 2 s, 5 scans, cm^{-1}): $\tilde{\nu}$ = 2921 (1), 2916 (1), 2858 (1), 2852 (1), 1613 (1), 1596 (1), 1593 (1), 1579 (2), 1575 (1), 1561 (2), 1501 (2), 1473 (10), 1438 (2), 1429 (2), 1425 (2), 1421 (2), 1412 (4), 1379 (1), 1376 (1), 1306 (5), 1286 (2), 1277 (1), 1267 (1), 1250 (1), 1245 (1), 1241 (1), 1226 (1), 1223 (2), 1184 (7), 1137 (4), 1093 (2), 1086 (1), 1066 (5), 1047 (1), 1036 (1), 1033 (1), 1008 (1), 969 (1), 966 (1), 957 (1), 954 (1), 948 (1), 942 (2), 933 (1), 875 (1), 857 (1), 820 (1), 714 (1), 709 (1), 577 (3), 564 (1), 559 (1), 551 (1), 543 (1), 525 (1), 522 (1), 514 (1), 497 (1), 494 (1), 488 (1), 342 (1), 338 (1). **MS** (EI, 70 eV, m/z): 281 (21, [M−(TerNP)$_2$]$^+$); 282 (25, [M−(TerNP)$_2$+H]$^+$); 296 (29); 297 (30); 298 (28); 299 (30); 310 (62); 311 (27); 312 (51, [Ter−H]$^+$); 313 (52, [Ter]$^+$); 314 (62); 324 (45, [M−(Ter)(Mes)$_2$]$^+$); 326 (65); 327 (33, [TerN]$^+$), 328 (72): 329 (100); 330 (75); 342 (37); 344 (41); 358 (94, [TerNP]$^+$); 359 (48); 608 (47); 609 (22); 671 (69); 672 (93, [Ter$_2$NP]$^+$); 673 (48); 686 (67, [TerNPNTer]$^+$); 687 (35); 733 (36); 734 (20); 748 (38); 749 (20); 971 (7, [M−N_2+H]$^+$).

Figure S13: NMR, IR and Raman spectra of ***E*-5B** (solvent signals indicated by asterisks).

$^{31}P\{^{1}H\}$ NMR spectrum

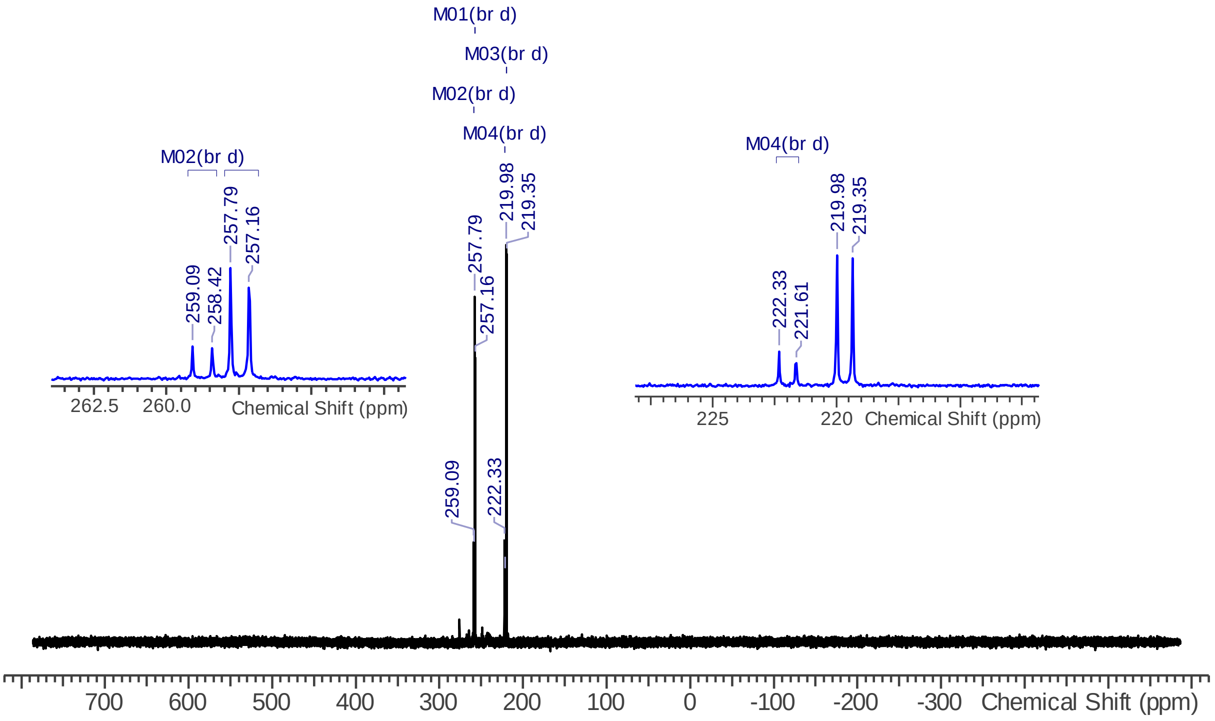

^{1}H NMR spectrum

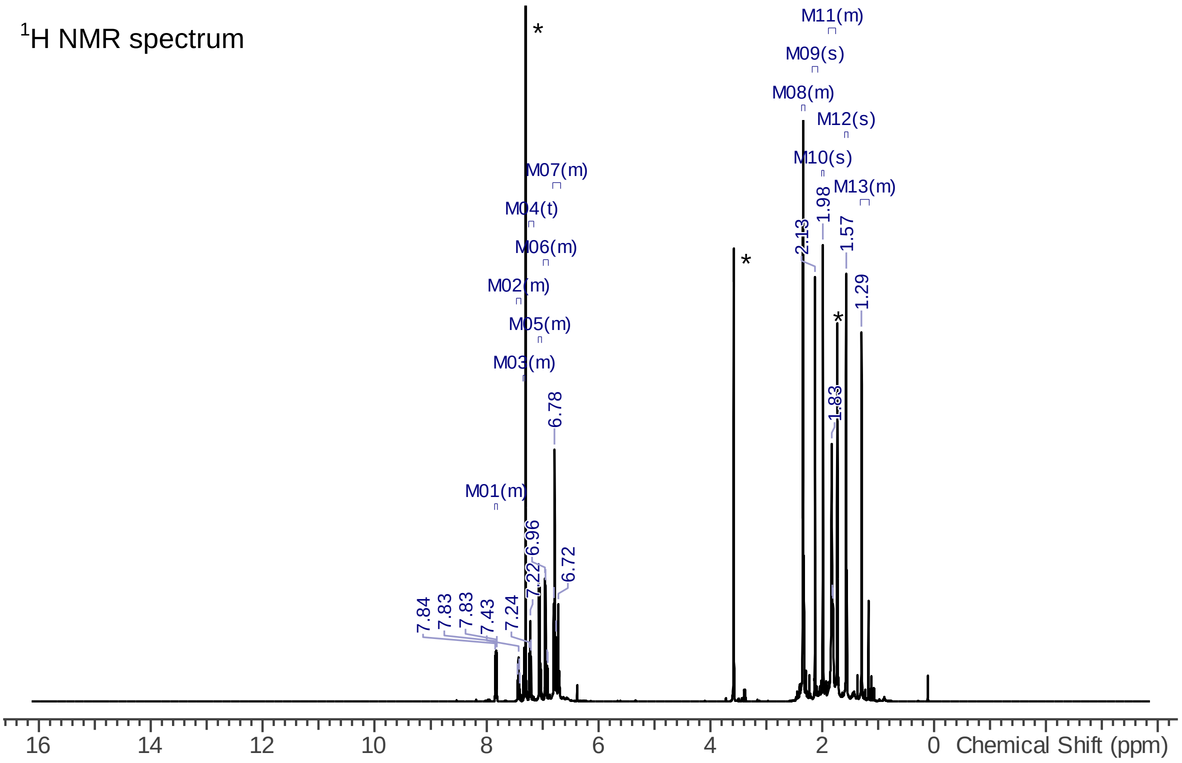

Figure S13 continued.

$^{13}C\{^1H\}$ NMR spectrum

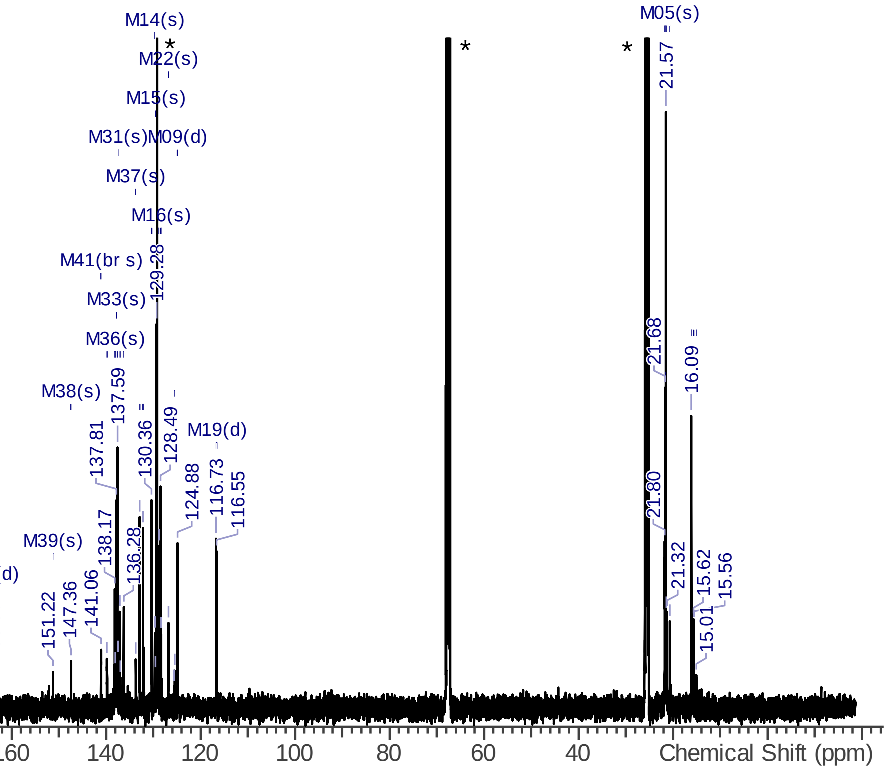

$^{19}F\{^1H\}$ NMR spectrum

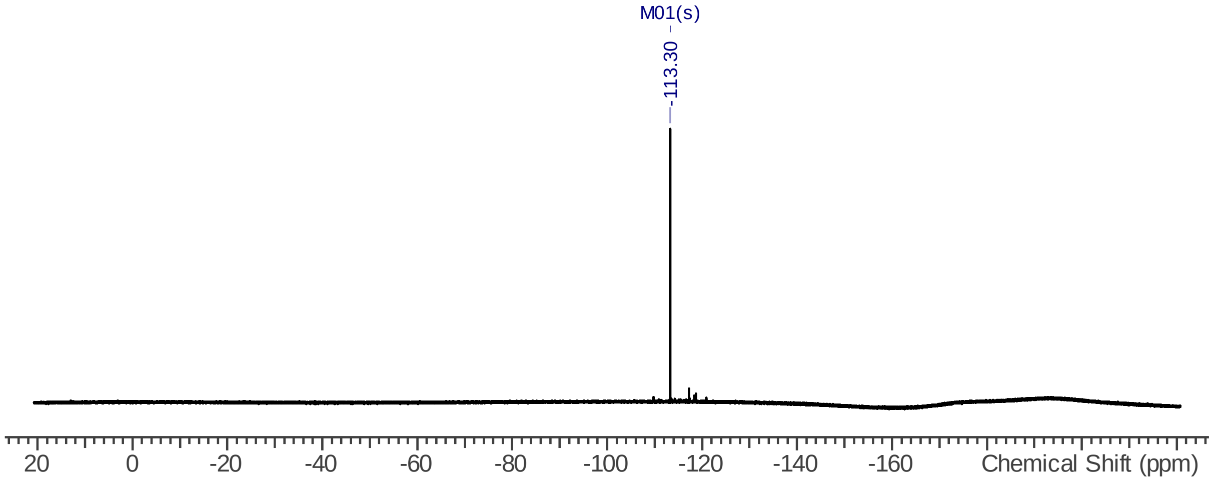

Figure S13 continued.

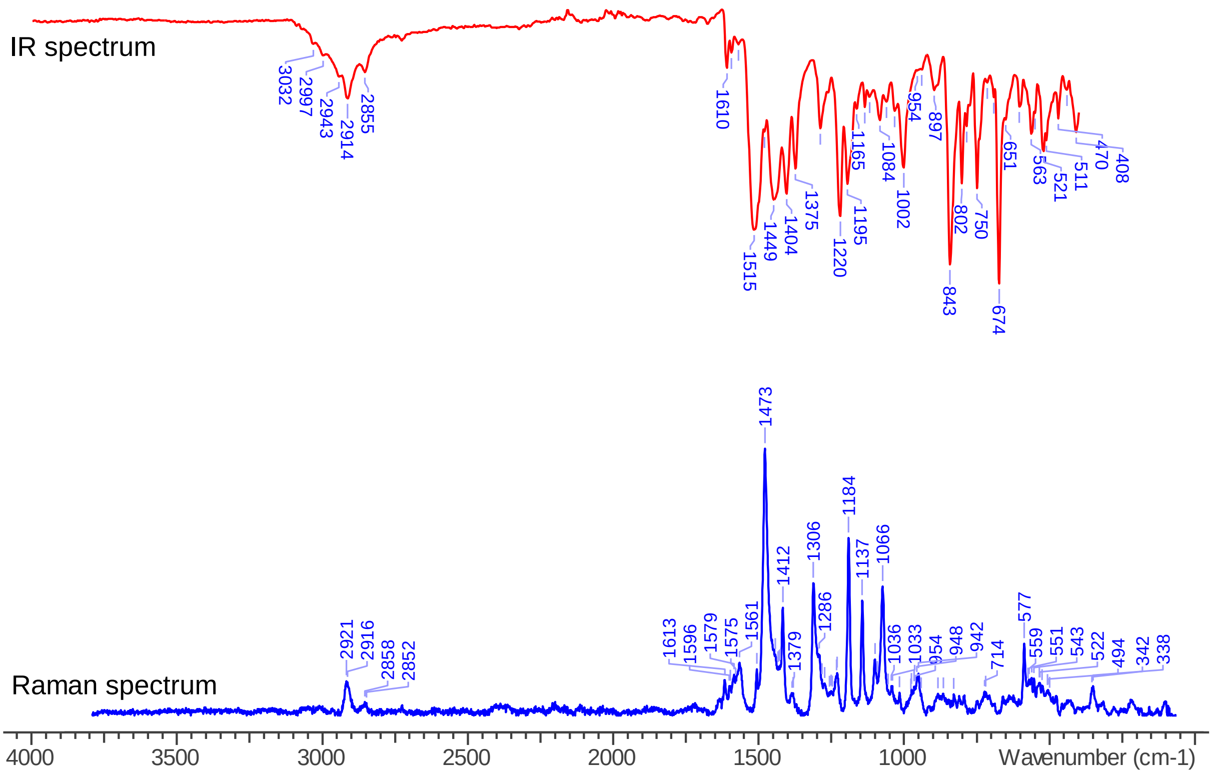

5 Additional spectroscopic details

5.1 UV/Vis spectra of azobenzenes *E*-10 and *E*-11

UV/Vis spectra of compounds ***E*-10** and ***E*-11** were recorded in order to verify the possible *E* to *Z* isomerisation reaction upon irradiation as depicted below.

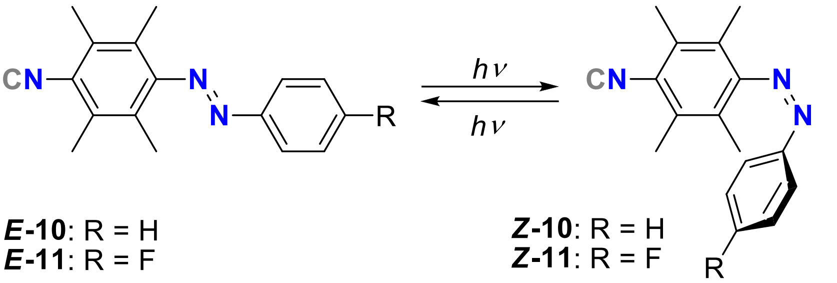

***E*-10**: R = H
***E*-11**: R = F

***Z*-10**: R = H
***Z*-11**: R = F

Spectra were recorded in benzene at room temperature in the dark first, subsequent irradiation with light from a commercial flashlight led to a slight change in the UV/Vis spectra (Figure S14 and Figure S15).

Figure S14: UV/Vis spectra of ***E*-10**: before (black) and after irradiation (red), (c = 1.16 mmol/L).

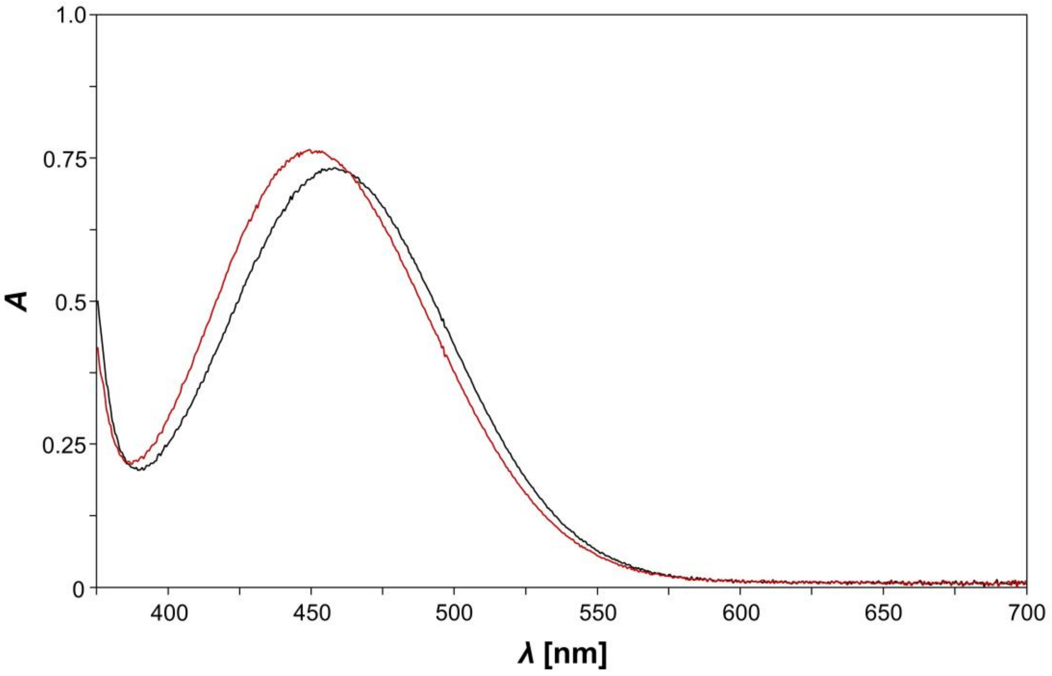

Azobenzene ***E*-10** exhibits one broad absorption maximum for the corresponding nπ* band. It appears at 458 nm for ***E*-10** (measured in the dark) and at 453 nm for the mixture with *Z*-azobenzene ***Z*-10** after irradiation.

Figure S15: UV/Vis spectra of ***E*-11**: before (black) and after irradiation (red), (c = 1.25 mmol/L).

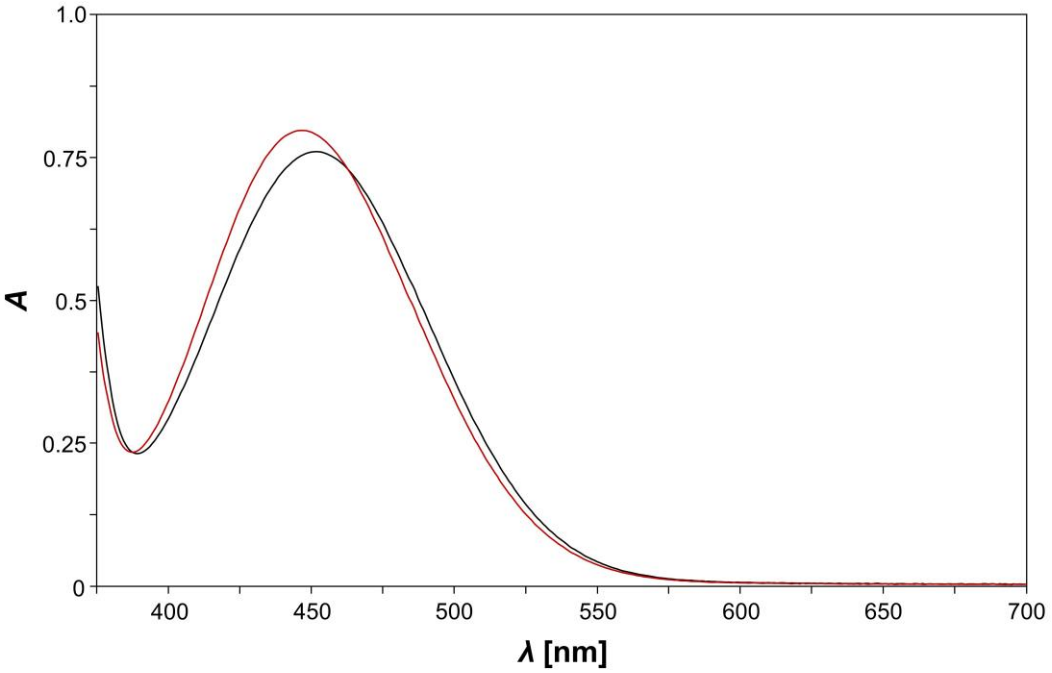

Azobenzene ***E*-11** also exhibits one broad absorption maximum for the corresponding nπ* band. It appears at 451 nm for ***E*-11** (measured in the dark) and 446 nm for the mixture with *Z*-azobenzene ***Z*-11** after irradiation.

5.2 ^{19}F NMR data of switching process of azobenzene *E*-11

The switching process of azobenzene ***E*-11** to ***Z*-11** was also investigated using ^{19}F{^{1}H} NMR spectroscopy at 235.36 MHz as depicted below.

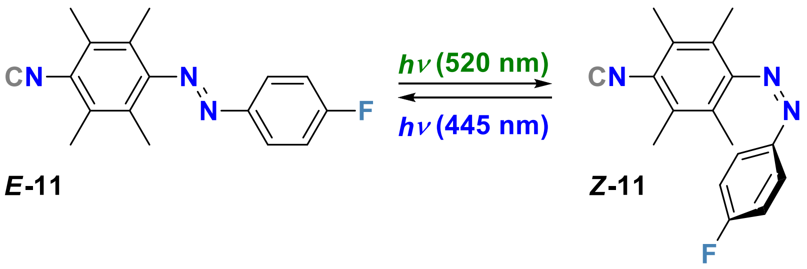

NMR spectra under irradiation were recorded using our previously published setup (which was adopted from a setup published by the Gschwind group) using a fibre-coupled (10 m multimode fibre, 0.39 NA, high OH, 1000 µm core diameter, ThorLabs FT1000UMT) laser diode (red: Oclaro HL63193MG, 638 nm, bandwidth 632-643 nm, 700 mW; green: Osram PL520, 520 nm, bandwidth 510-530 nm, 50 mW; blue: Nichia NDB7875, 445 nm, bandwidth 435-455 nm, 1600 mW; UV: Sony SLD3235VF, 405 nm, bandwidth 400-410 nm, 100 mW).[4,10]

A solution of ***E*-11** (7 mg, 0.02 mmol) in THF-d_8 (0.4 mL) was prepared and transferred to the spectrometer. $^{19}F\{^1H\}$ NMR spectra were recorded at room temperature without irradiation in the dark first, followed by irradiation with green light (520 nm) and blue light (455 nm). The spectra can be seen in Figure S16.

Figure S16: $^{19}F\{^1H\}$ NMR spectra of ***E*-11** at room temperature before irradiation, after irradiation with green light and after irradiation with blue light.

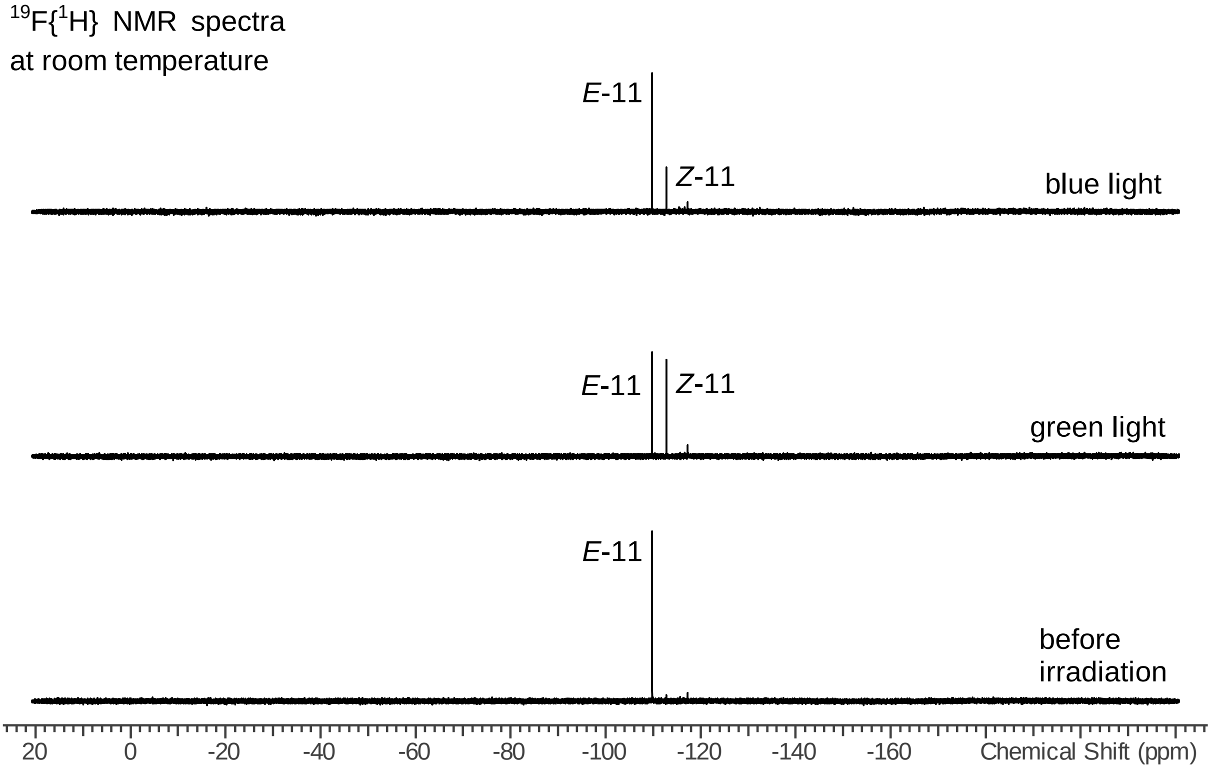

Before the irradiation process one singlet at −109.8 ppm can be seen for the *E* isomer ***E*-11**. Therefore, no *Z* isomer ***Z*-11** is formed when synthesizing or storing compound ***E*-11**. Upon irradiation with green light (for 15 min) ***Z*-11** is formed (singlet at

−112.8 ppm); however, no complete conversion can be observed. In the photo stationary state (PSS) ***E*-11** and ***Z*-11** are present approximately in a 1:1 ratio. Irradiation with blue light (also for 15 min) induces the reverse reaction to form ***E*-11**. Again, no complete conversion can be achieved, significant amounts of ***Z*-11** are still present in the PSS.

The solution was then cooled to low temperatures (−40 °C) to conduct the same experiment, starting with the PSS containing ***E*-11** and small amounts of ***Z*-11** from the previous experiment under irradiation. The spectra for the irradiation process at −40 °C can be seen in Figure S17.

Figure S17: $^{19}F\{^{1}H\}$ NMR spectra of ***E*-11** at −40 °C before irradiation, after irradiation with green light and after irradiation with blue light.

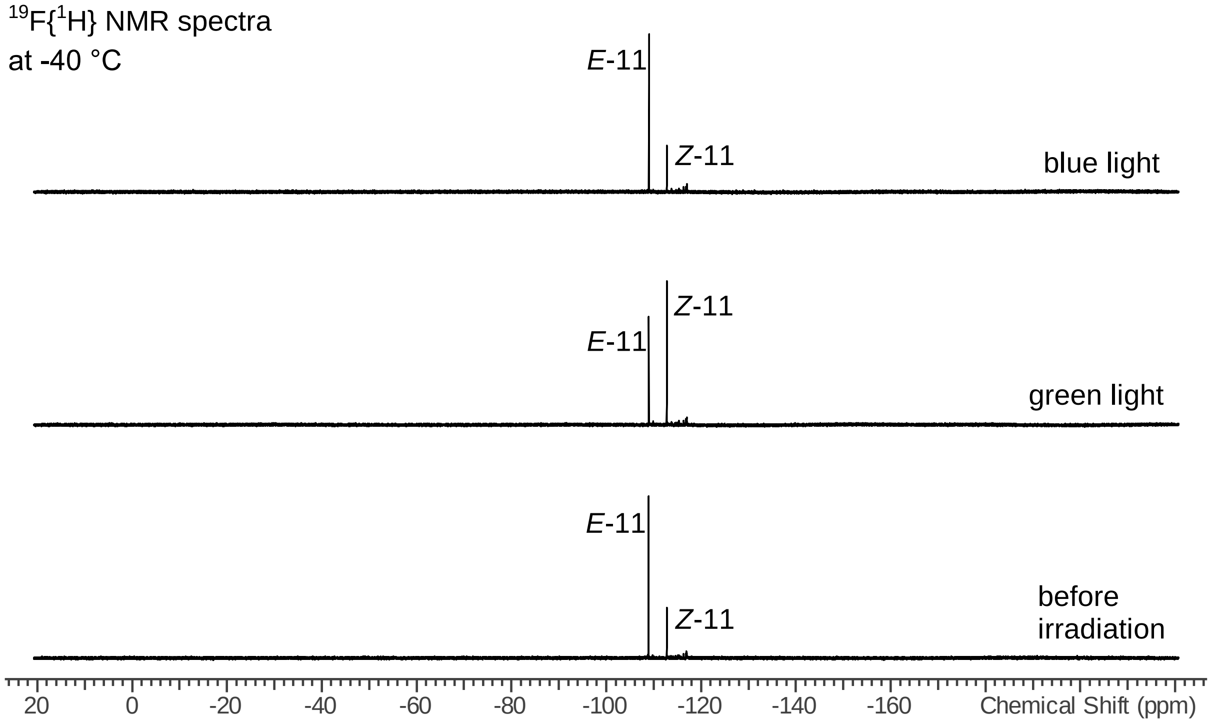

After irradiating the solution with green light at −40 °C the PSS contains a slightly larger amount of ***Z*-11** (singlet at −112.8 ppm) but again no complete conversion can be achieved. The same applies for the reverse reaction when irradiating the sample with blue light: the amount of ***E*-11** increases significantly (singlet at −109.0 ppm) but the PSS still contains considerable amounts of ***Z*-11**.

5.3 UV/Vis spectra of *E*-4B

Compound ***E*-4B** was also investigated using UV/Vis spectroscopy. As with compounds ***E*-10** and ***E*-11** spectra were recorded in benzene at room temperature in the dark first. Subsequent irradiation with light presumably led to the formation of a mixture of compounds ***E*-4B**, ***E*-4H**, ***Z*-4H** and ***Z*-4B**.

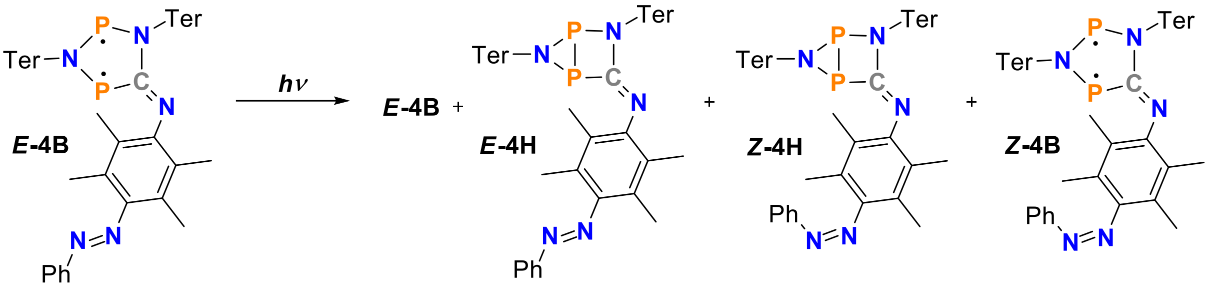

The recorded UV/Vis spectra can be seen in Figure S18.

Figure S18: UV/Vis spectra of ***E*-4B**: before (black) and after irradiation (red), (c = 0.16 mmol/L).

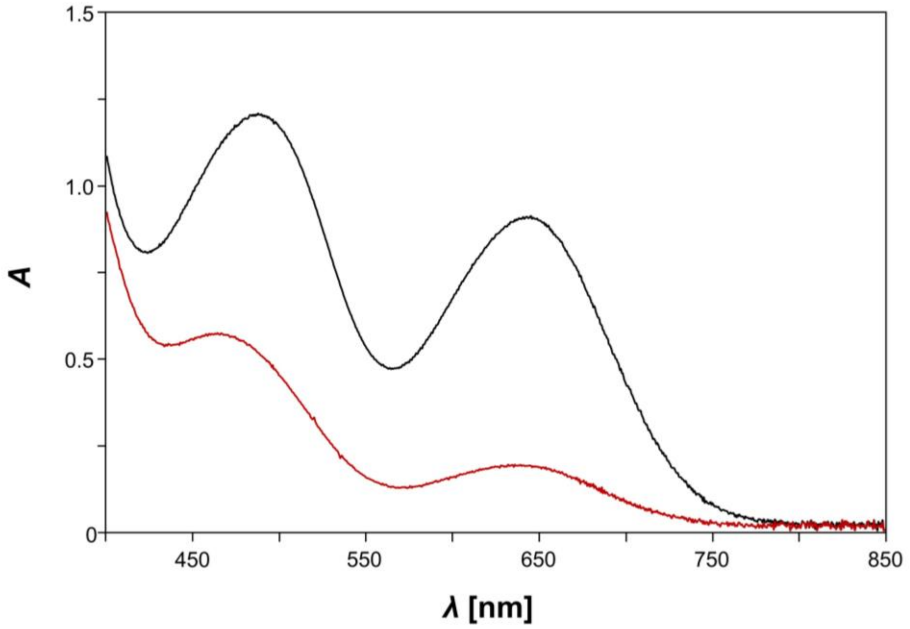

As shown compound ***E*-4B** exhibits broad absorption maxima with the first centred around 645 nm. Therefore, absorption of red light of the electromagnetic spectrum should lead to the formation of the housane type species due to population of the corresponding transannular bonding orbital between the P atoms.[4] A typical

absorption spectrum for a 1,3-biradical (Dmp-derivative **2B**) can be seen in Figure S19 with the absorption maximum centred at 643 nm.[4]

Figure S19: UV/Vis spectrum of 1,3-biradical **2B**.[4]

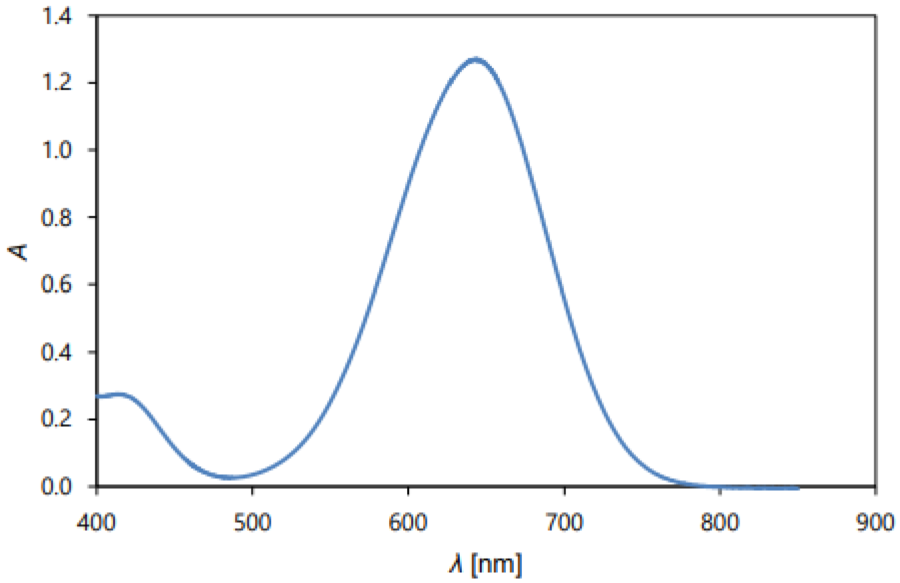

The second absorption maximum is centred around 487 nm and can be attributed to the nπ* transition of the azobenzene (*vide supra*). However, in contrast to starting material ***E*-10** a significant red shift of the absorption maximum can be observed (29 nm).

After irradiation with light the absorption maxima for the mixture of compounds ***E*-4B**, ***E*-4H**, ***Z*-4H** and ***Z*-4B** differ significantly. While the maximum at 645 nm almost completely disappears due to the subsequent housane formation, the maximum of the nπ* band of the azobenzene experiences a blue shift from 487 nm to 464 nm due to *E* to *Z* isomerisation and housane formation (alongside decreasing absorbance). However, no clear identification of the switching products can be carried out.

To investigate the thermal reverse reaction the freshly irradiated mixture of ***E*-4B**, ***E*-4H**, ***Z*-4H** and ***Z*-4B** is left in the dark in the spectrometer and UV/Vis spectra are recorded every 3 minutes. After 30 minutes the absorption maximum at 645 nm can be observed again due to re-formation of the biradical species, the maximum of the

nπ* band of the azobenzene however is located around 482 nm as probably small amounts of *Z* isomer are still present alongside the *E* isomer. Again, no clear identification of the *E*- and *Z*-isomers can be carried out. (Figure S20).

Figure S20: UV/Vis spectra of the thermal reverse reaction to re-form biradical species: after irradiation (red) towards storage for 30 min in the dark (black), spectra recorded every 3 min, (c = 0.16 mmol/L).

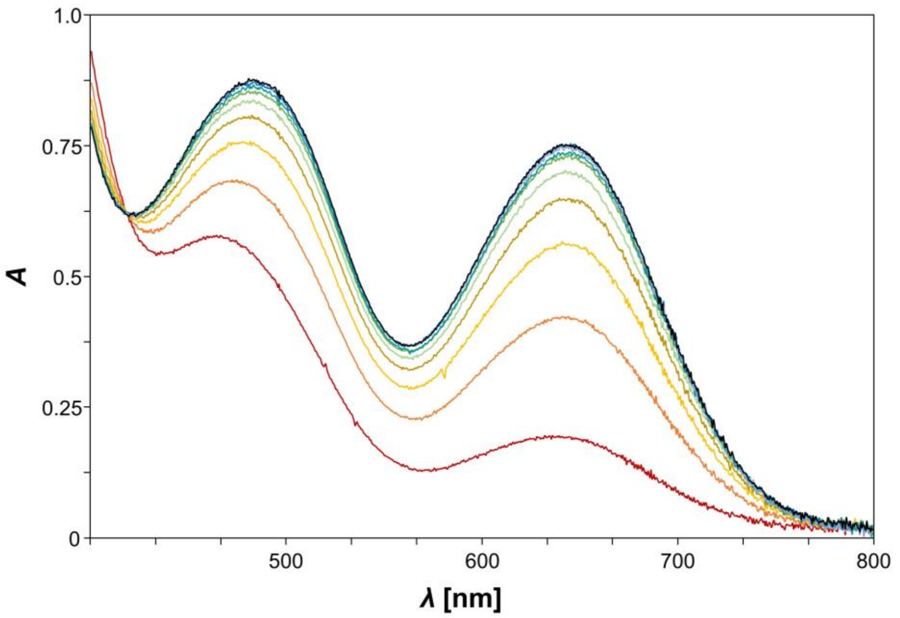

5.4 UV/Vis spectra of *E*-5B

Compound ***E*-5B** was similarly investigated using UV/Vis spectroscopy. Spectra were also recorded in the dark first (in benzene). Subsequent irradiation with light presumably led to the formation of a mixture of compounds ***E*-5B**, ***E*-5H**, ***Z*-5H** and ***Z*-5B**.

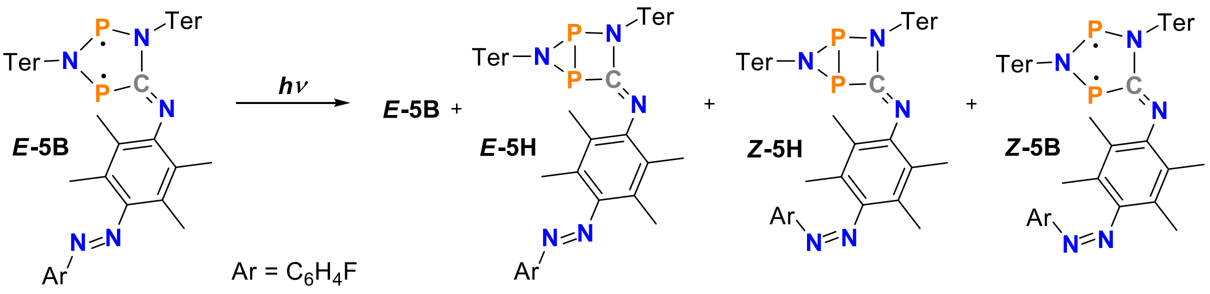

The recorded UV/Vis spectra can be seen in Figure S21. In this instance concentrations were chosen such that the absorption maxima located above 400 nm not exceed an absorbance of 1.0.

Figure S21: UV/Vis spectra of ***E*-5B**: before (black) and after irradiation (red), (c = 0.14 mmol/L).

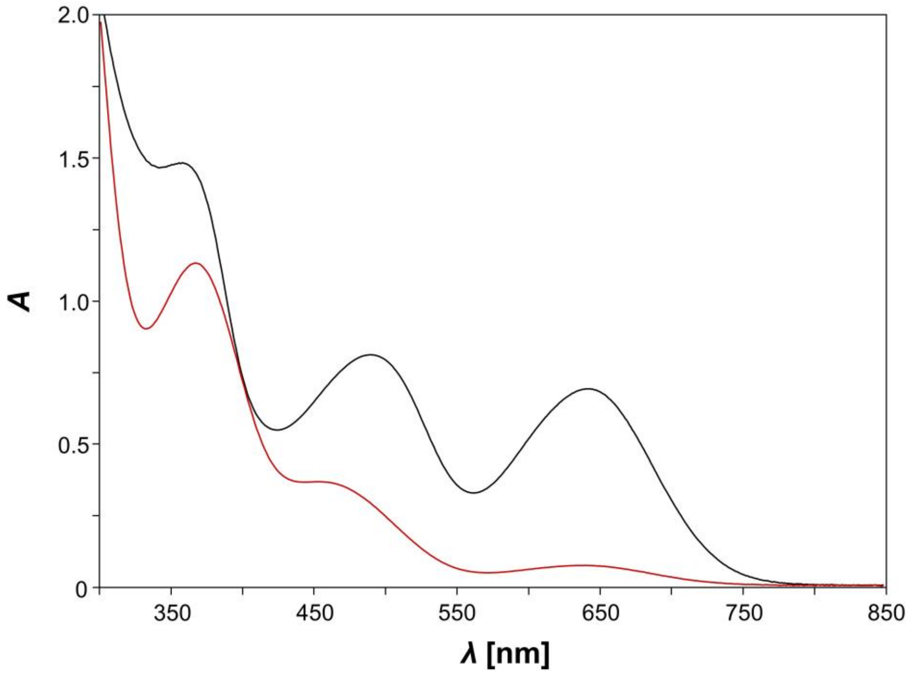

Compound ***E*-5B** also exhibits broad absorption maxima, the first centred around 644 nm. Therefore, absorption of red light of the visible spectrum should also lead to the formation of the housane type species due to population of the corresponding transannular bonding orbital between the P atoms.[4] The second absorption maximum is centred around 490 nm and again can be attributed to the nπ* transition band of the azobenzene (*vide supra*). However, in contrast to starting material ***E*-11** a significant red shift of the absorption maximum can be observed (39 nm). The absorption maximum corresponding to the ππ* transition is located at around 358 nm.

After irradiation with light the absorption maxima for the mixture of compounds ***E*-5B**, ***E*-5H**, ***Z*-5H** and ***Z*-5B** differ significantly. While the maximum at 644 nm almost completely disappears due to the subsequent housane formation, the maximum of the nπ* band of the azobenzene experiences a blue shift from 490 nm to 454 nm, probably due to *E* to *Z* isomerisation and housane formation (alongside decreasing absorbance).

The absorption maximum corresponding to the ππ* transition experiences a slight red shift and is now located at 368 nm. Again, no clear identification of the switching products can be carried out.

To investigate the thermal reverse reaction the freshly irradiated mixture of ***E*-5B**, ***E*-5H**, ***Z*-5H** and ***Z*-5B** is left in the dark in the spectrometer and UV/Vis spectra are recorded every 3 minutes. After 30 minutes the absorption maximum at 644 nm can be observed again due to re-formation of the biradical species. The maximum of the nπ* band of the azobenzene is now located around 488 nm as probably small amounts of *Z* isomer are still present alongside the *E* isomer. The same applies for the absorption maximum of the ππ* band: after leaving the sample in the dark for 30 minutes the maximum can no longer be identified clearly as it overlaps with other absorption bands below wavelengths of 350 nm (most likely caused by aromatic ring systems of the terphenyl substituents).

The spectra for the thermal reverse reaction are depicted in Figure S22.

Figure S22: UV/Vis spectra of the thermal reverse reaction to re-form biradical species: after irradiation (red) towards storage for 30 min in the dark (black), spectra recorded every 3 min, (c = 0.14 mmol/L).

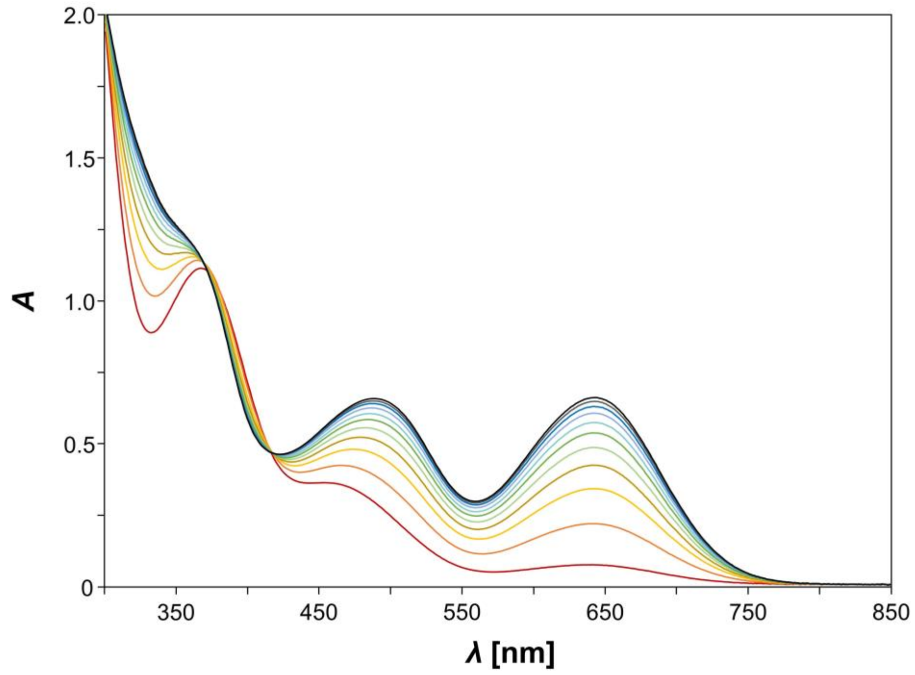

The molar extinction coefficient ε of compound ***E*-5B** was determined from differently concentrated solutions by linear regression (Figure S23) according to the Beer-Lambert law (with absorbance A, concentration of the analyte c, optical path length L).

$$A = \varepsilon \cdot c \cdot L$$

Figure S23: Absorbance *A* vs concentration at λ = 644 nm.

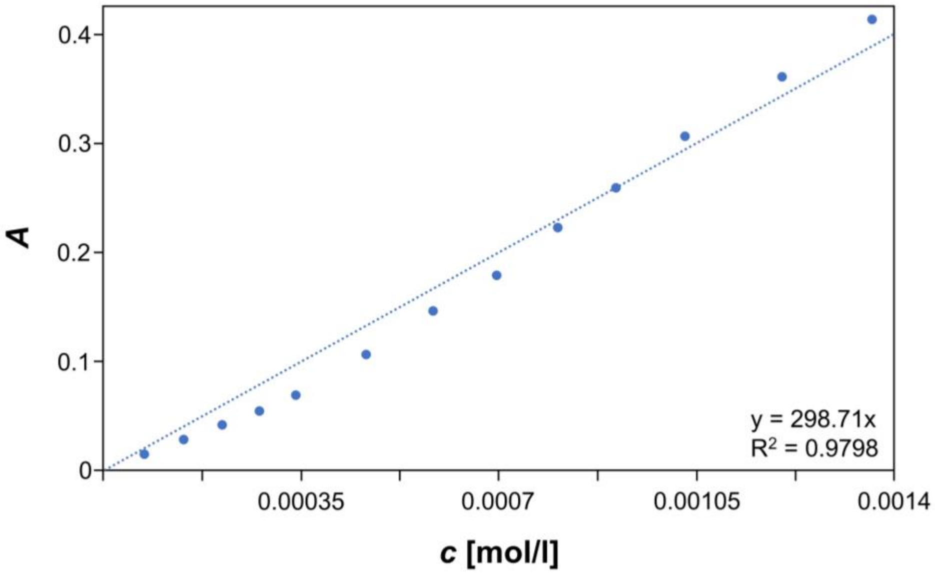

The extinction coefficient was determined for the absorption maximum at 644 nm and has a value of ε_{644} = 2.9(8)×10^5 L mol^{-1} m^{-1}. The margin of error can be attributed to the extreme sensitivity of ***E*-5B** towards air and moisture and the small concentrations of the sample solutions. The spectra were recorded using toluene as a solvent. A solution of ***E*-5B** in toluene was also irradiated with light from different wavelengths while measuring UV/Vis spectra. These spectra were acquired on an Analytik Jena UV-VIS spectrophotometer Specord s600 using a Hellma Excalibur Q Lab all-quartz immersion probe (optical pathlength of 1 mm). Irradiation was performed with a Superlite 400 by LUMATEC with different wavelength filters (recorded spectra depicted in Figure S24).

Figure S24: UV/Vis spectra during irradiation of ***E*-5B** with green light (green line), blue light (blue line) and UV light (purple line) (c = 0.0014 mol/L).

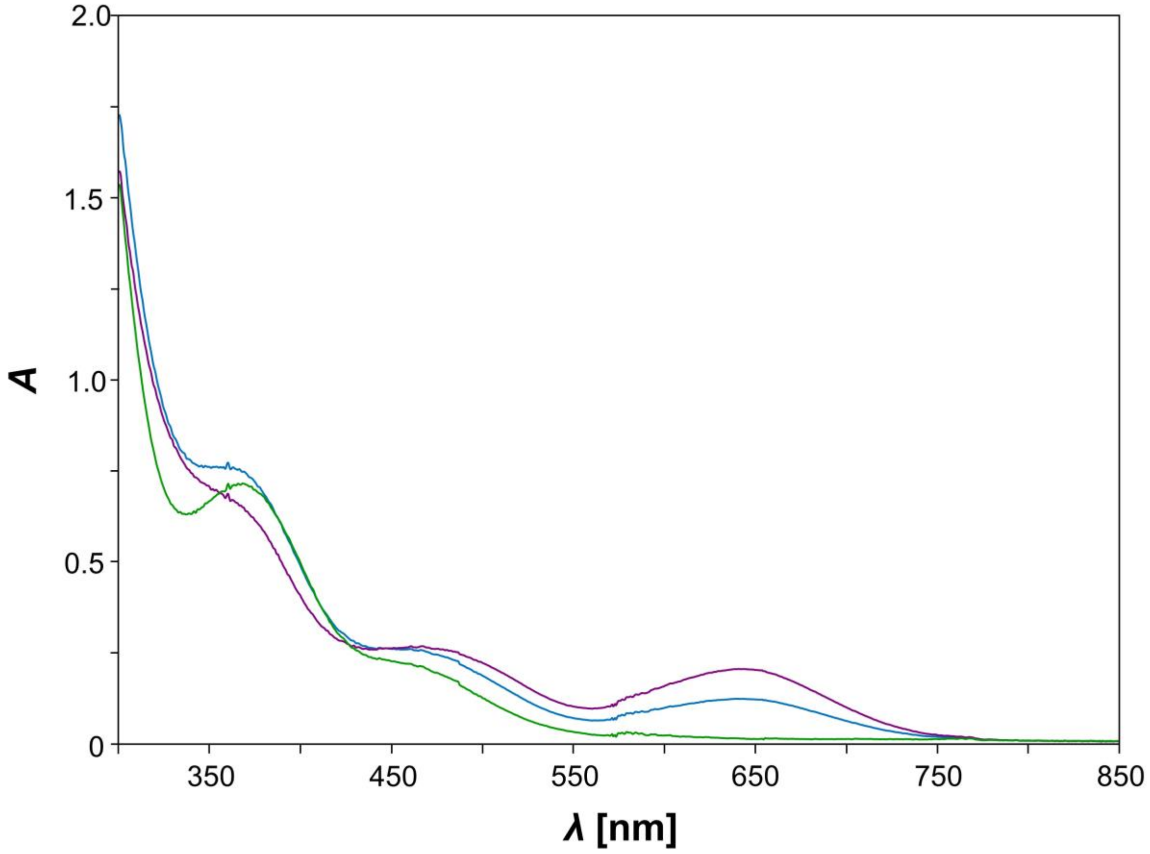

While only housanes ***E*-5H** and ***Z*-5H** are present when irradiating the solution with green light (λ = 570 nm, lack of absorption maximum around 644 nm) the irradiation with blue (λ = 440 nm) or UV light (λ = 320-400 nm) at room temperature does not lead to significant changes of the nπ* and ππ* bands. In fact, irradiation with blue or UV light is not sufficient to prevent the thermal reverse reaction from happening and biradical compounds ***E*-5B** and probably ***Z*-5B** are formed in the mixture alongside ***E*-5H** and ***Z*-5H**.

5.5 ^{31}P NMR data of switching process of *E*-4B

The switching process of compound ***E*-4B** was investigated at first. 50 mg of ***E*-4B** (49 mmol) were dissolved in 0.4 mL of THF-d_8 in an NMR tube equipped with a coaxial insert. The sample was prepared inside an argon filled drybox in the dark, covering the NMR tube thoroughly with aluminium foil. Afterwards the sample was transferred to the spectrometer and ^{31}P{^{1}H} NMR spectra were recorded at room temperature (approx. 25 °C) at a frequency of 101.25 MHz.

As with compound ***E*-11** all NMR spectra under irradiation were recorded using our previously published setup (which was adopted from a setup published by the Gschwind group) using a fibre-coupled (10 m multimode fibre, 0.39 NA, high OH, 1000 µm core diameter, ThorLabs FT1000UMT) laser diode (red: Oclaro HL63193MG, 638 nm, bandwidth 632-643 nm, 700 mW; green: Osram PL520, 520 nm, bandwidth 510-530 nm, 50 mW; blue: Nichia NDB7875, 445 nm, bandwidth 435-455 nm, 1600 mW; UV: Sony SLD3235VF, 405 nm, bandwidth 400-410 nm, 100 mW).[4,10]

For this compound, only the switching process from the biradical ***E*-4B** to the housane type species ***E*-4H** is discussed, as no proof for the *E* to *Z* isomerisation at the azobenzene moiety could be provided using ^{31}P{^{1}H} NMR spectroscopy.

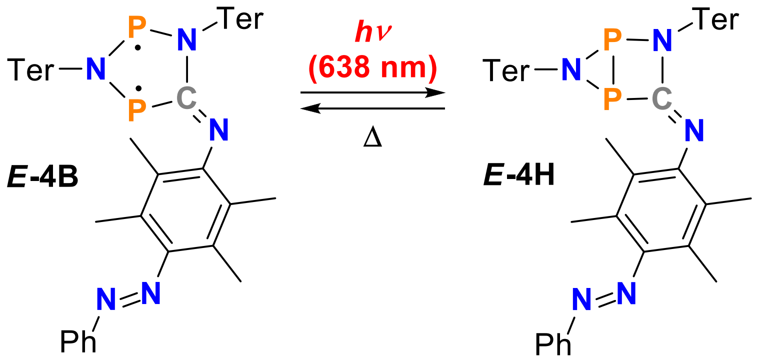

The recorded spectra are depicted in Figure S25.

Figure S25: $^{31}P\{^1H\}$ NMR spectra of switching process of ***E*-4B** to ***E*-4H** at room temperature under irradiation with red light.

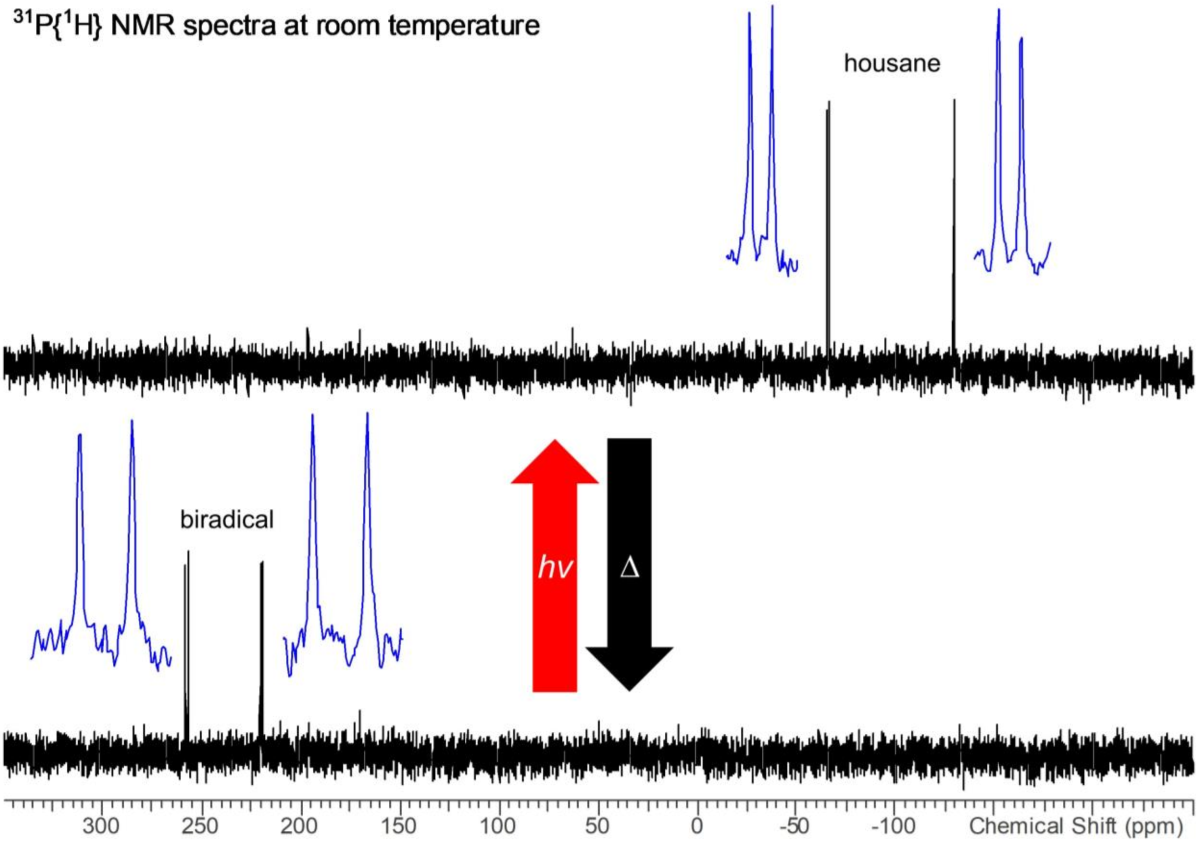

Biradical ***E*-4B** shows two doublets with chemical shifts of 221.0 ppm (N*P*C) and 257.0 ppm (N*P*N) with a coupling constant of $^2J(^{31}P,^{31}P)$ = 127 Hz. When irradiating biradical ***E*-4B** (bottom) with red light, the corresponding housane type species (top) is formed. The housane type species ***E*-4H** also shows two doublets in the $^{31}P\{^1H\}$ NMR spectrum however these experience a strong upfield shift. The signals are located at chemical shifts of −129.8 ppm (N*P*C) and −66.2 ppm (N*P*N) with a coupling constant of $^1J(^{31}P,^{31}P)$ = 65 Hz. If the light is switched off the thermal reverse reaction takes place and biradical ***E*-4B** is formed again in the dark. The switching process can be carried out numerous times without decomposition of the biradical or the housane type species.

5.6 ^{31}P NMR data of switching process starting with *E*-5B at −40 °C

The switching process of compound ***E*-5B** was investigated at low temperatures (−40 °C) at 101.25 MHz first. To this end, 50 mg of ***E*-5B** (50 mmol) were dissolved in 0.4 mL of THF-d_8 in an NMR tube equipped with a coaxial insert. The sample was prepared inside an argon filled drybox in the dark, covering the NMR tube thoroughly with aluminium foil.

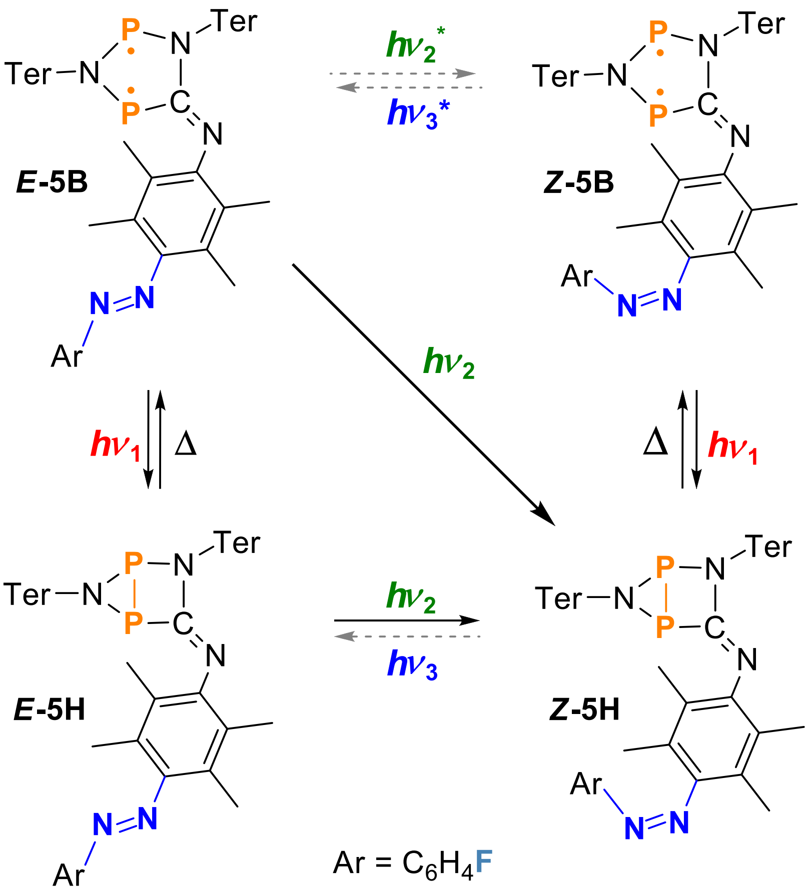

The recorded spectra can be seen below in Figure S26. Spectra showing the signals in further detail will be discussed below starting with Figure S27.

Figure S26: $^{31}P\{^1H\}$ NMR spectra starting with ***E*-5B** at −40 °C before irradiation, after irradiation with red light, after irradiation with green light and after heating the sample to room temperature with re-cooling.

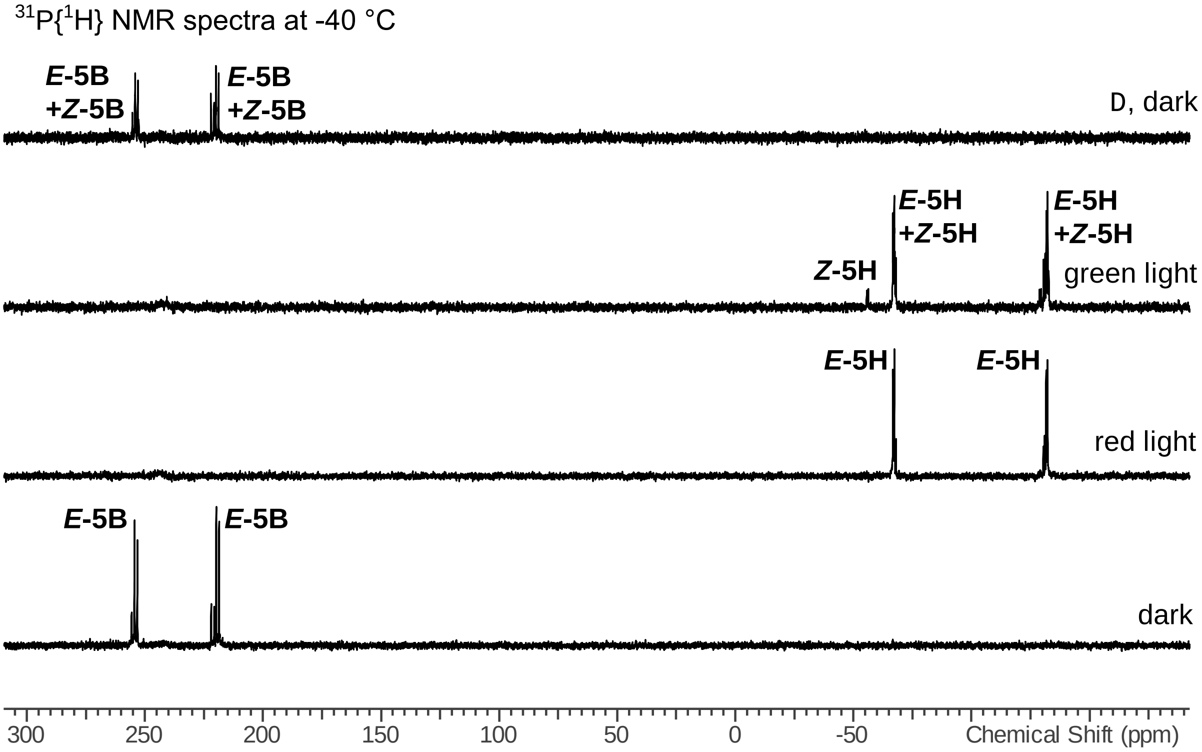

After transferring the sample to the NMR spectrometer, it was cooled to −40 °C and $^{31}P\{^1H\}$ NMR spectra were recorded in the dark showing the two isomers (I^A and I^B) of ***E*-5B** with four doublets as expected: 219.2 ppm (br. d, $^2J(^{31}P,^{31}P)$ = 120 Hz, I^A-N*P*C), 221.3 ppm (br. d, $^2J(^{31}P,^{31}P)$ = 130 Hz, I^B-N*P*C), 253.8 ppm (br. d, $^2J(^{31}P,^{31}P)$ = 120 Hz, I^A-N*P*N), 255.1 ppm (br. d, $^2J(^{31}P,^{31}P)$ = 130 Hz, I^B-N*P*N). Afterwards the sample was irradiated with light from a red laser diode, the spectra for the switching process under red light can be seen in Figure S27.

Figure S27: $^{31}P\{^1H\}$ NMR spectra of switching process from ***E*-5B** to ***E*-5H** at −40 °C under irradiation with red light.

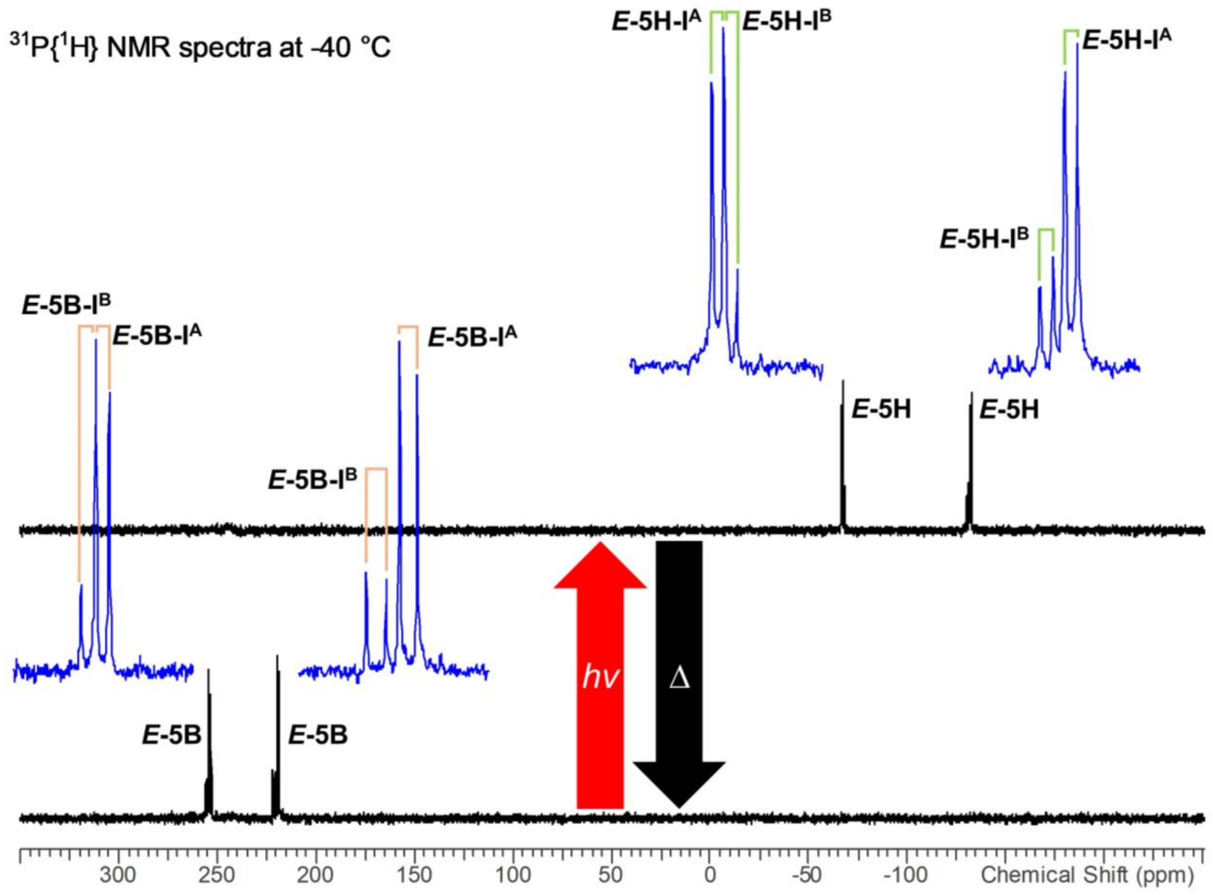

When irradiating compound ***E*-5B** (bottom) with red light, the corresponding housane type species ***E*-5H** (top) is formed. The resonances of the doublets in Figure S27 can be assigned on the basis of the coupling constants of 120 Hz for I^A and 130 Hz for I^B in case of ***E*-5B**. As the doublets in the spectra occasionally overlap the resonances of the doublets of ***E*-5B** are indicated by orange brackets while the resonances of ***E*-5H** are indicated by green brackets. For the two isomers of compound ***E*-5H** also four doublet resonances can be seen and assigned with the coupling constants: −131.9 ppm (br. d, $^1J(^{31}P,^{31}P)$ = 62 Hz, I^A-N*P*C), −130.8 ppm (br. d, $^1J(^{31}P,^{31}P)$ = 62 Hz, I^B-N*P*C), −67.7 ppm (br. d, $^1J(^{31}P,^{31}P)$ = 62 Hz, I^B-N*P*N), −67.1 ppm (br. d, $^1J(^{31}P,^{31}P)$ = 62 Hz, I^A-N*P*N).

To further induce the *E* to *Z* isomerisation, compound ***E*-5H** was irradiated with green light, the spectra for the switching process can be seen in Figure S28.

Figure S28: $^{31}P\{^{1}H\}$ NMR spectra of switching process from ***E*-5H** to ***Z*-5H** at −40 °C under irradiation with green light.

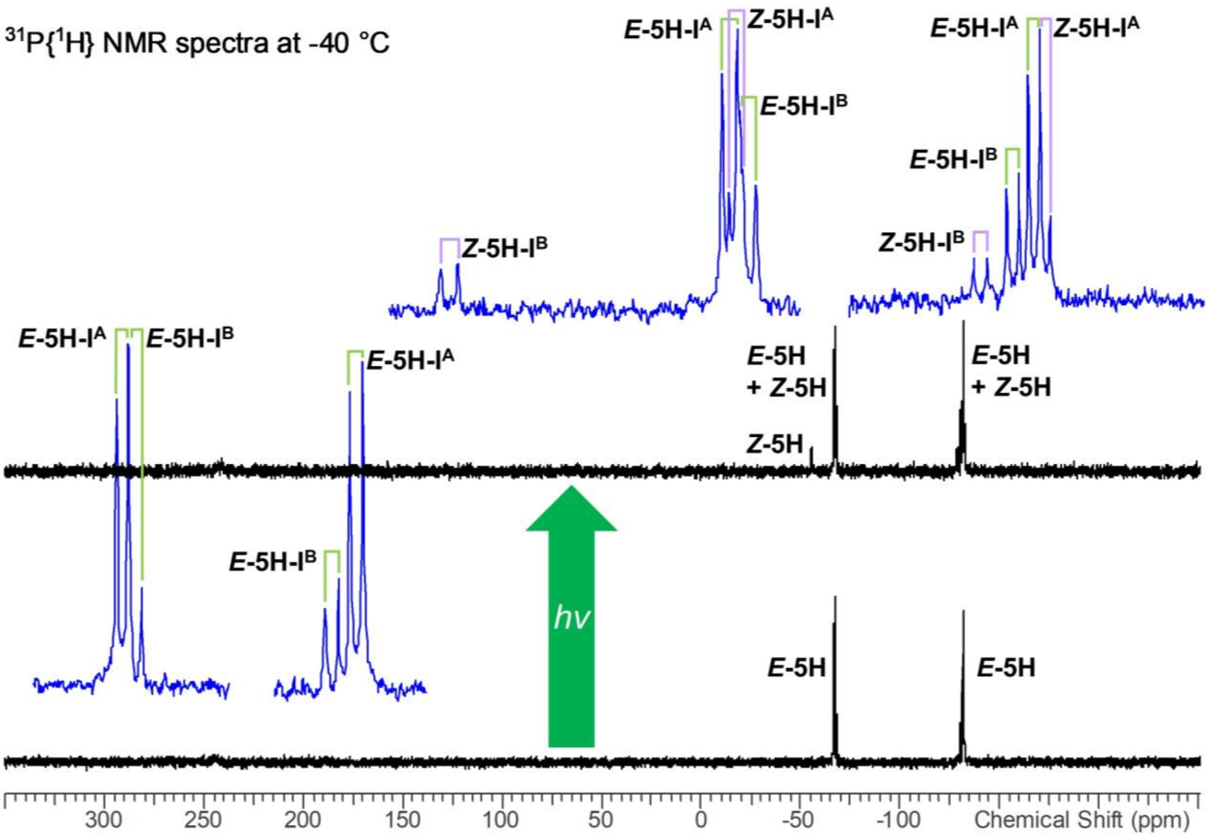

Upon irradiation of the sample with green light partial formation of the housane *Z* isomer ***Z*-5H** takes place. In the $^{31}P\{^{1}H\}$ NMR spectra shown above this can be seen by the appearance of four doublets of compound ***Z*-5H** (isomers A and B). As these doublets overlap with resonances of remaining ***E*-5H** (indicated by green brackets), the signals for ***Z*-5H** are highlighted by purple brackets. The doublets can be assigned again by the use of the coupling constant of 62 Hz. The signals of compound ***Z*-5H** show the following chemical shifts: −132.5 ppm (br. d, $^{1}J(^{31}P,^{31}P)$ = 62 Hz, I^{A}-N*P*C), −129.2 ppm (br. d, $^{1}J(^{31}P,^{31}P)$ = 62 Hz, I^{B}-N*P*C), −67.2 ppm (br. d, $^{1}J(^{31}P,^{31}P)$ = 62 Hz, I^{A}-N*P*N), −55.9 ppm (br. d, $^{1}J(^{31}P,^{31}P)$ = 62 Hz, I^{B}-N*P*N).

In order to synthesize the biradical *Z* isomer ***Z*-5B**, the sample mixture of ***E*-5H** and ***Z*-5H** was left in the dark at 25 °C to perform the thermal reverse reaction and then re-cooled back to −40 °C, the spectra for the switching process can be seen in Figure S29.

Figure S29: $^{31}P\{^1H\}$ NMR spectra of switching process from ***Z*-5H** to ***Z*-5B** and ***E*-5H** to ***E*-5B** at −40 °C after performing the thermal reverse reaction in the dark at room temperature.

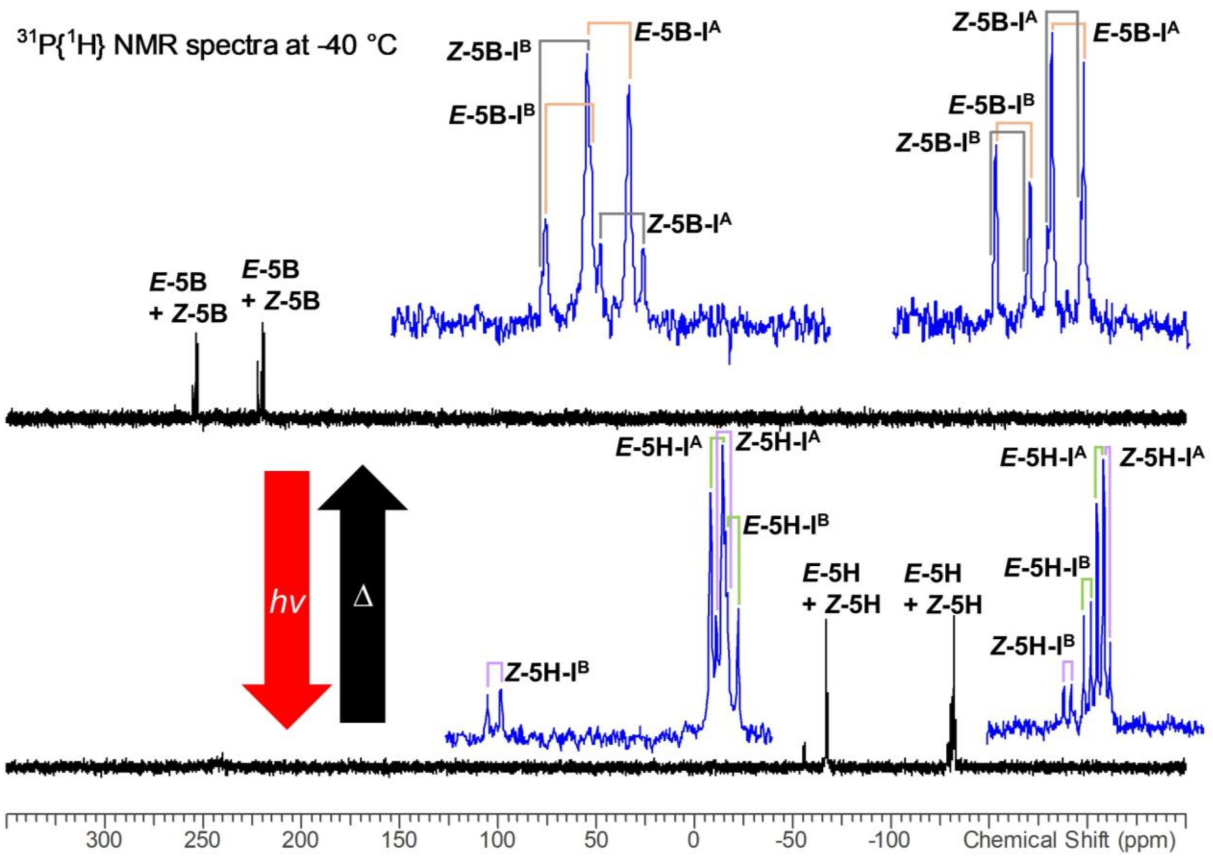

When the thermal reverse reaction is performed (at room temperature) both housane species (***E*-5H** and ***Z*-5H**) react back to the corresponding isomers of the biradical ***Z*-5B** and ***E*-5B**. For better comparability of the spectra, the solution was re-cooled to −40 °C. The four newly appearing doublets of compound ***Z*-5B** overlap significantly with the signals of the re-formed starting material ***E*-5B** and are indicated with grey brackets. The doublet resonances of ***Z*-5B** can be assigned by coupling constants of 120 Hz for I^A and 130 Hz for I^B and are in the same range as the coupling constants of starting material ***E*-5B**. The signals for the isomers of ***Z*-5B** show the following chemical shifts: 219.4 ppm (br. d, $^2J(^{31}P,^{31}P)$ = 120 Hz, I^A-N*P*C), 221.5 ppm (br. d, $^2J(^{31}P,^{31}P)$ = 130 Hz, I^B-N*P*C), 253.3 ppm (br. d, $^2J(^{31}P,^{31}P)$ = 120 Hz, I^A-N*P*N), 255.2 ppm (br. d, $^2J(^{31}P,^{31}P)$ = 130 Hz, I^B-N*P*N).

The reverse reaction to form ***E*-5H** and ***Z*-5H** can also be performed by irradiation with red light, showing the reversibility of the switching process (Figure S29).

In contrast to the stepwise reaction discussed above both the biradical to housane isomerisation and *E* to *Z* isomerisation can be performed in one step simply by irradiating the solution containing starting material ***E*-5B** with green light. As green light induces both isomerisation reactions, a mixture of ***E*-5H** (green brackets) and ***Z*-5H** (purple brackets) can be obtained via this reaction pathway as well, the species show similar resonances as discussed before. The $^{31}P\{^{1}H\}$ NMR spectra for this switching process (−40 °C) are depicted below in Figure S30.

Figure S30: $^{31}P\{^{1}H\}$ NMR spectra of switching process from ***E*-5B** to ***E*-5H** and ***Z*-5H** at −40 °C under irradiation with green light.

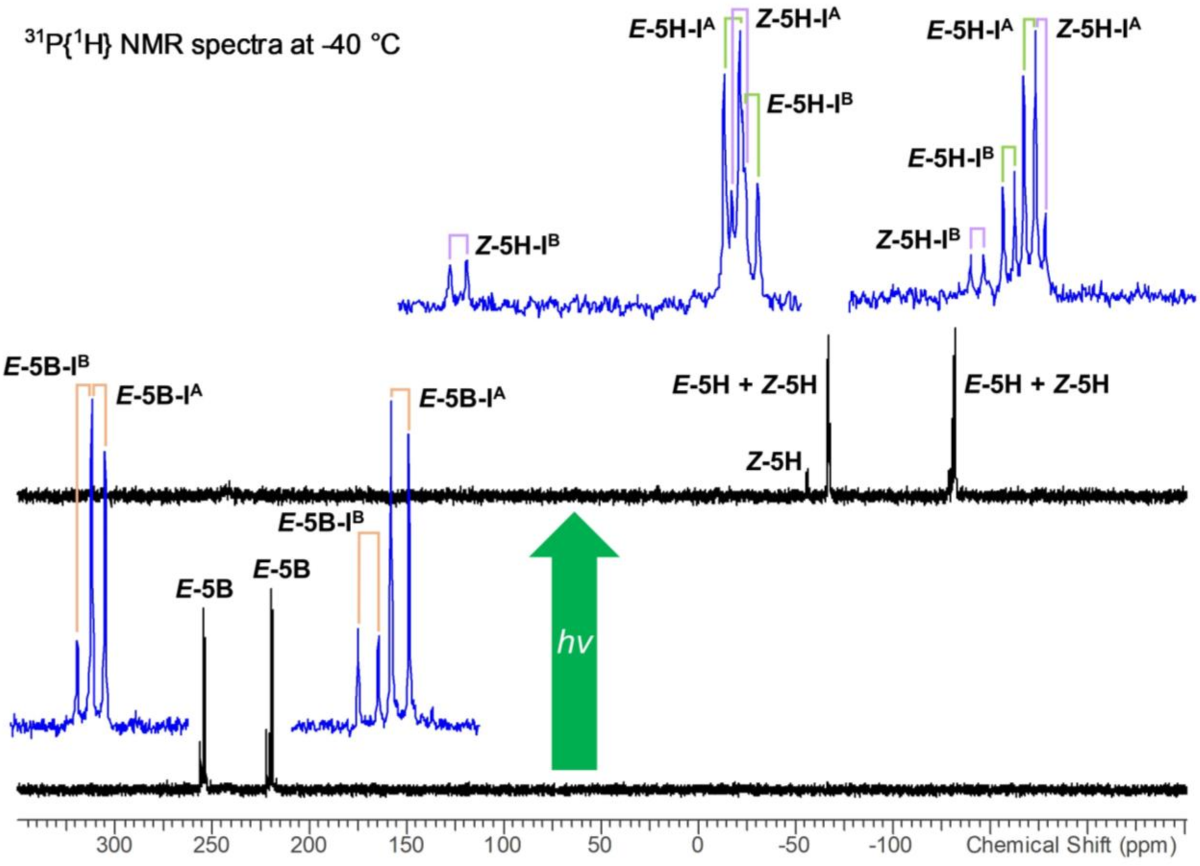

As the *Z* to *E* isomerisation reaction could be induced under irradiation with blue light on isonitrile ***E*-11**, the same reaction was attempted on system ***Z*-5H**. For this the reaction mixture of compounds ***E*-5H** and ***Z*-5H** was irradiated with blue light at −40 °C to see if the signals for compound ***Z*-5H** disappear at the expense of housane ***E*-5H**. The spectra are depicted in Figure S31.

Figure S31: $^{31}P\{^1H\}$ NMR spectra of attempted switching process from ***Z*-5H** to ***E*-5H** at −40 °C under irradiation with blue light.

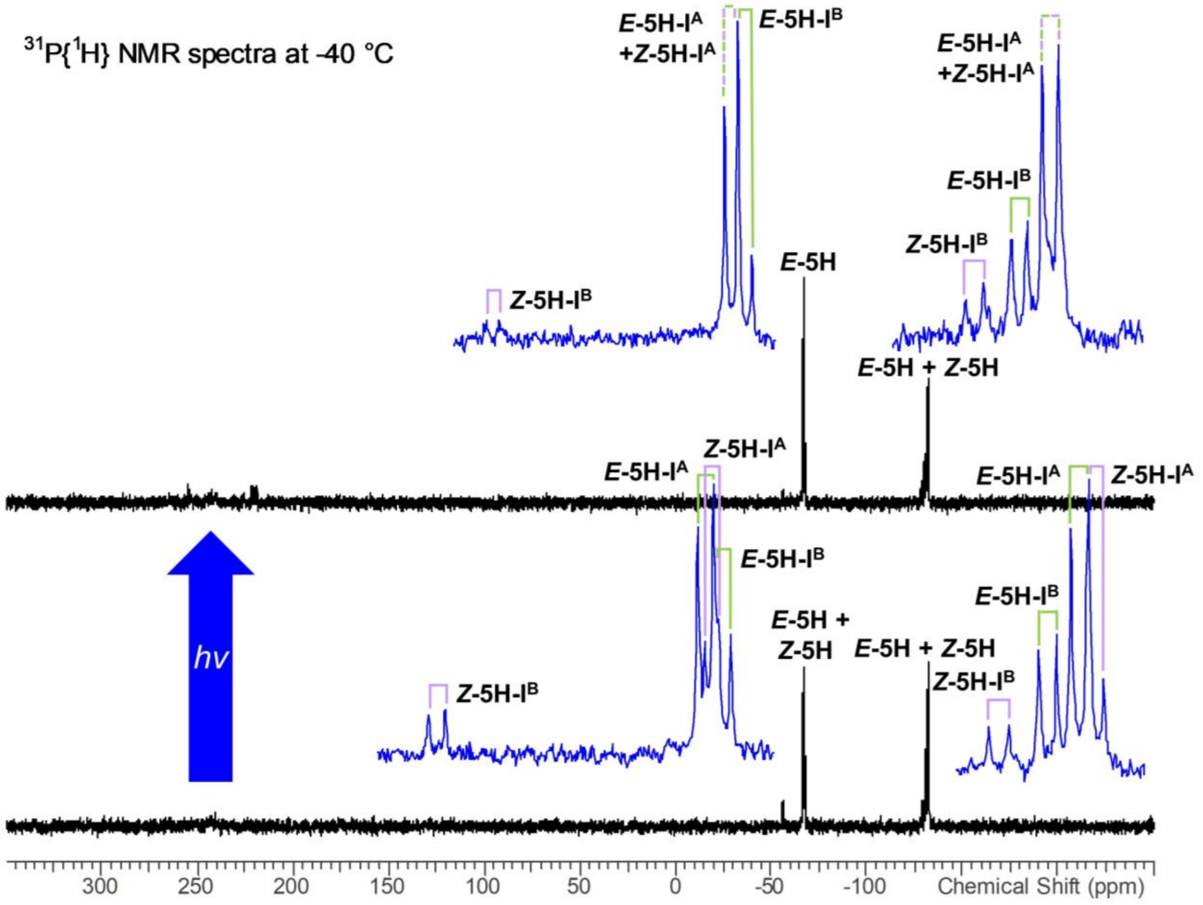

In the spectra the intensity of the signals of ***Z*-5H** decreases slightly but two doublets of species ***Z*-5H** can still be identified clearly, indicating, that the conversion is incomplete. The signals of ***Z*-5H-I^A** overlap heavily with the signals of ***E*-5H-I^A**. Most likely the creation of a photo stationary state (PSS) occurs in which both *Z* to *E* as well as *E* to *Z* isomerisation reactions take place at the same time (*i.e.* both species ***E*-5H** and ***Z*-5H** are constantly present in the mixture and therefore detectable in the $^{31}P\{^1H\}$ NMR spectra).

5.7 ^{31}P NMR data of switching process starting with *E*-5B at 25 °C

The same experiments were carried out at room temperature at 101.25 MHz. For this also 50 mg of ***E*-5B** (50 mmol) were dissolved in 0.4 mL of THF-d_8 in an NMR tube equipped with a coaxial insert. The sample was prepared inside an argon filled drybox in the dark, covering the NMR tube thoroughly with aluminium foil. ^{31}P{^{1}H} NMR spectra were recorded in the dark, followed by spectra recorded under irradiation with red and green light (spectra in Figure S32).

Figure S32: ^{31}P{^{1}H} NMR spectra starting with ***E*-5B** at room temperature before irradiation, after irradiation with red light, after irradiation with green light and after heating the sample to room temperature with re-cooling.

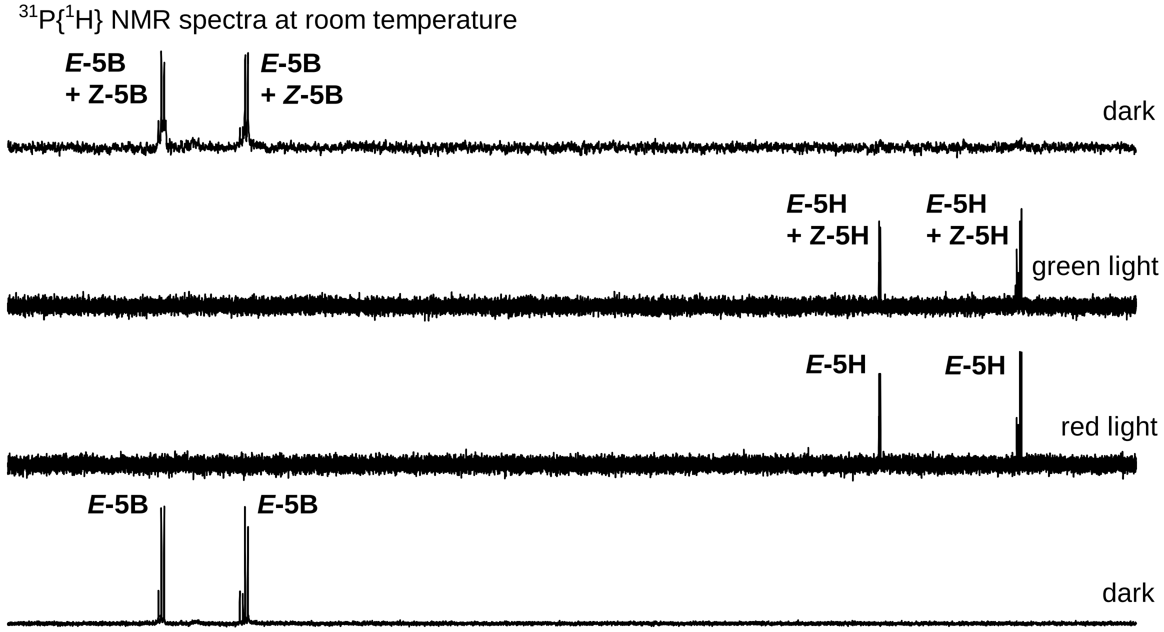

The ^{31}P{^{1}H} NMR spectrum in the dark again shows the two isomers (I^A and I^B) of ***E*-5B**: 219.7 ppm (br. d, $^{2}J(^{31}P,^{31}P)$ = 127 Hz, I^A-N*P*C), 222.0 ppm (br. d, $^{2}J(^{31}P,^{31}P)$ = 136 Hz, I^B-N*P*C), 257.5 ppm (br. d, $^{2}J(^{31}P,^{31}P)$ = 127 Hz, I^A-N*P*N), 258.8 ppm (br. d, $^{2}J(^{31}P,^{31}P)$ = 136 Hz, I^B-N*P*N). Afterwards the sample was irradiated with light from a red laser diode, the spectra for the switching process under red light can be seen in Figure S33.

Figure S33: $^{31}P\{^1H\}$ NMR spectra of switching process from ***E*-5B** to ***E*-5H** at room temperature under irradiation with red light.

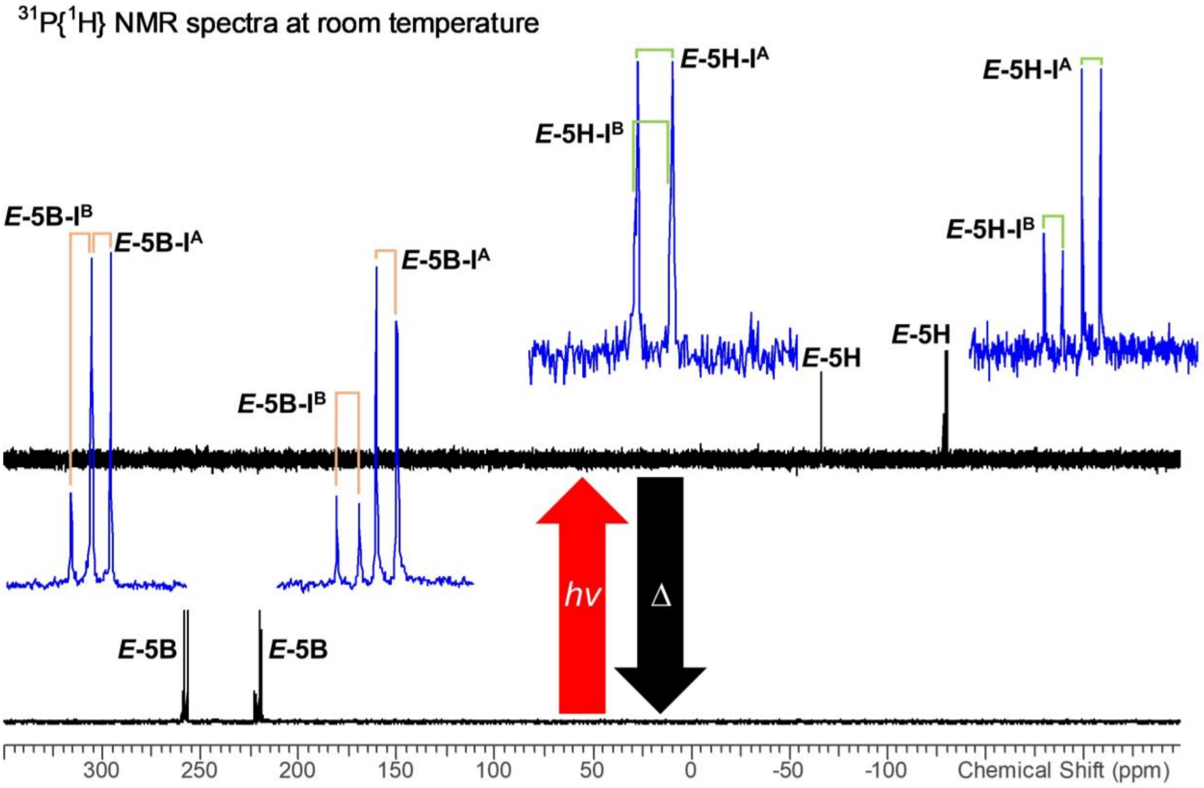

When irradiating compound ***E*-5B** (bottom) with red light, housane type species ***E*-5H** (top) is formed. Again, the resonances of the doublets in Figure S33 can be assigned on the basis of the coupling constants of 127 Hz for I^A and 136 Hz for I^B in case of ***E*-5B**. As the doublets in the spectra occasionally overlap the resonances of the doublets of ***E*-5B** are indicated by orange brackets while the resonances of ***E*-5H** are indicated by green brackets. For the two isomers of compound ***E*-5H** also four doublet resonances can be seen and assigned with the coupling constants: −129.9 ppm (br. d, $^1J(^{31}P,^{31}P)$ = 65 Hz, I^A-N*P*C), −128.5 ppm (br. d, $^1J(^{31}P,^{31}P)$ = 65 Hz, I^B-N*P*C), −66.3 ppm (br. d, $^1J(^{31}P,^{31}P)$ = 65 Hz, I^A-N*P*N), −66.2 ppm (br. d, $^1J(^{31}P,^{31}P)$ = 65 Hz, I^B-N*P*N).

To further induce the *E* to *Z* isomerisation, compound ***E*-5H** was irradiated with green light, the spectra for the switching process can be seen in Figure S34.

Figure S34: $^{31}P\{^{1}H\}$ NMR spectra of switching process from ***E*-5H** to ***Z*-5H** at room temperature under irradiation with green light.

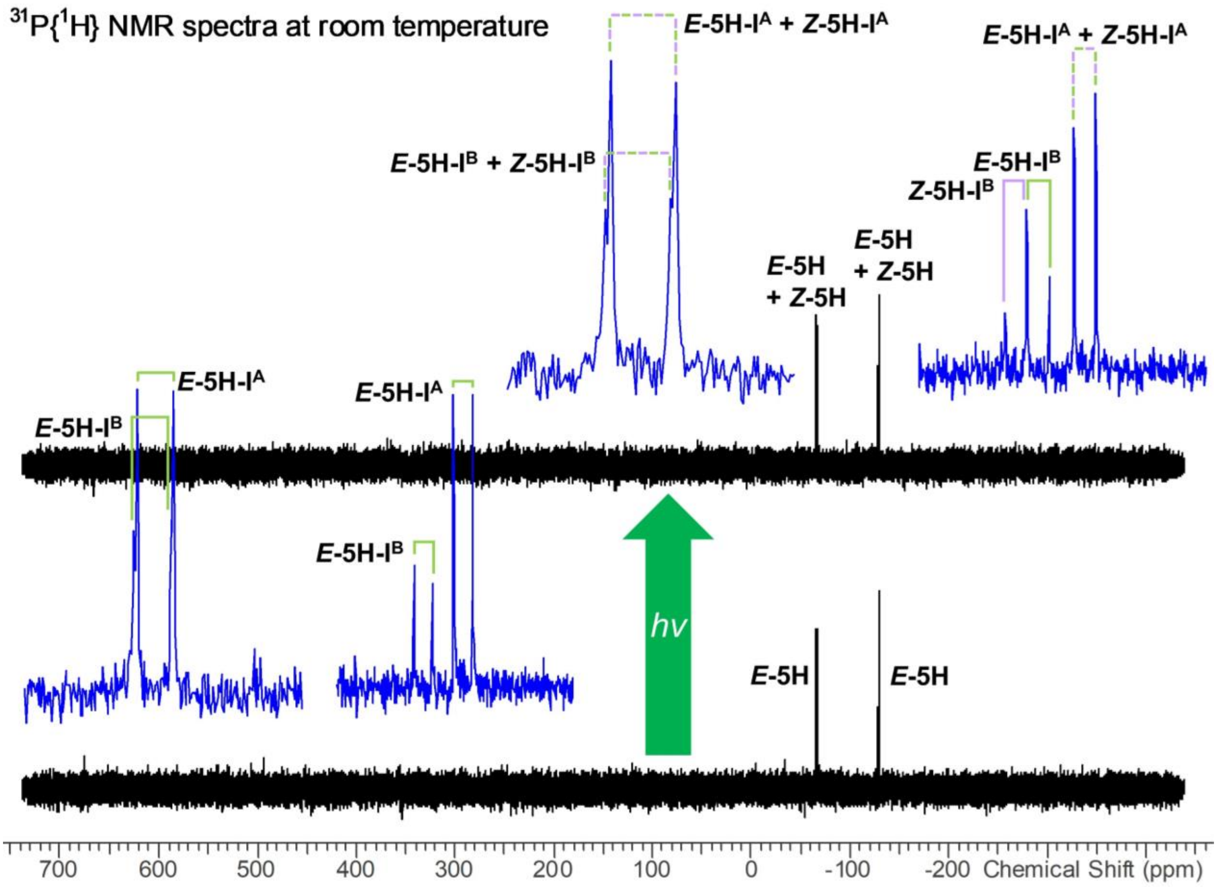

Upon irradiation of the sample with green light partial formation of the housane *Z* isomer ***Z*-5H** takes place. In the $^{31}P\{^{1}H\}$ NMR spectra shown above this can be seen by the appearance of the signals for I^A and I^B of compound ***Z*-5H**. The doublets almost perfectly overlap with resonances of remaining ***E*-5H** (indicated by green brackets). The signals for ***Z*-5H** are highlighted by purple brackets. The doublets can be assigned again by the use of the coupling constant of 65 Hz. The signals of compound ***Z*-5H** show the following chemical shifts (almost identical to compound ***E*-5H**): −129.9 ppm (br. d, $^{1}J(^{31}P,^{31}P)$ = 65 Hz, I^A-N*P*C), −127.9 ppm (br. d, $^{1}J(^{31}P,^{31}P)$ = 65 Hz, I^B-N*P*C), −66.3 ppm (br. d, $^{1}J(^{31}P,^{31}P)$ = 65 Hz, I^A-N*P*N), −66.2 ppm (br. d, $^{1}J(^{31}P,^{31}P)$ = 65 Hz, I^B-N*P*N). Due to the heavily overlapping signals of ***E*-5H** and ***Z*-5H** no irradiation with blue light was attempted in this regard to induce *Z* to *E* isomerisation. To synthesize the biradical *Z* isomer ***Z*-5B**, the sample mixture of ***E*-5H** and ***Z*-5H** was left in the dark to

perform the thermal reverse reaction, the spectra for the switching process can be seen in Figure S35.

Figure S35: $^{31}P\{^{1}H\}$ NMR spectra of switching process from ***Z*-5H** to ***Z*-5B** and ***E*-5H** to ***E*-5B** at room temperature in the dark.

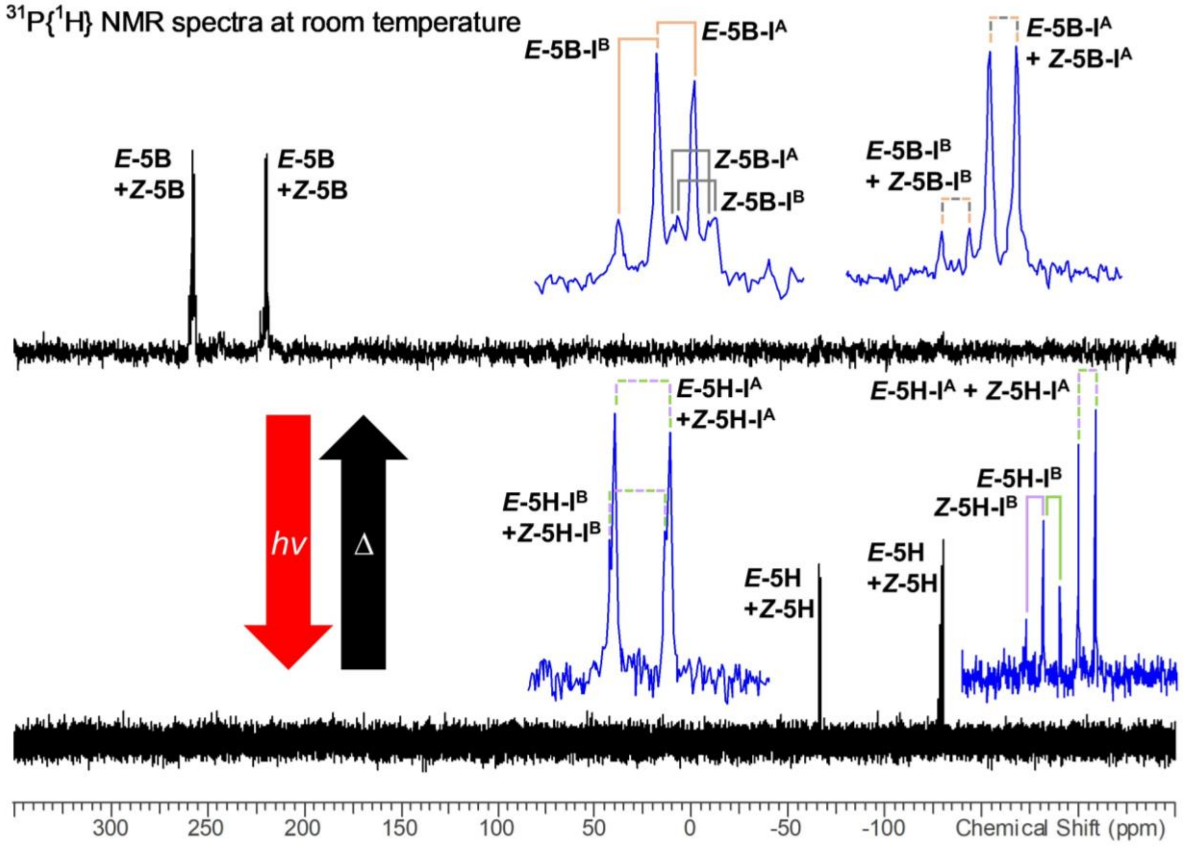

When the thermal reverse reaction is performed in the dark both housane species (***E*-5H** and ***Z*-5H**) react back to the corresponding isomers of the biradical species: ***Z*-5B** and ***E*-5B**. The four newly appearing doublets of compound ***Z*-5B** again overlap heavily with the signals of the re-formed starting material ***E*-5B** and are indicated with grey brackets. The doublet resonances of ***Z*-5B** can be assigned by coupling constants of 127 Hz for I^A and 136 Hz for I^B and are in the same range as the coupling constants of starting material ***E*-5B**. The signals of ***Z*-5B** show the following chemical shifts: 219.4 ppm (br. d, $^{2}J(^{31}P,^{31}P)$ = 127 Hz, I^A-N*P*C), 221.5 ppm (br. d, $^{2}J(^{31}P,^{31}P)$ = 136 Hz, I^B-N*P*C), 256.7 ppm (br. d, $^{2}J(^{31}P,^{31}P)$ = 136 Hz, I^B-N*P*N), 257.0 ppm (br. d, $^{2}J(^{31}P,^{31}P)$ = 127 Hz, I^A-N*P*N).

5.8 ^{19}F NMR data of switching process starting with *E*-5B at −40 °C

The same experiment to verify the switching process of compound ***E*-5B** was also monitored by the use of $^{19}F\{^1H\}$ NMR spectroscopy at 235.36 MHz. The spectra were recorded alongside the $^{31}P\{^1H\}$ NMR spectra at −40 °C using the same solution of ***E*-5B** (50 mg, 50 mmol) in 0.4 mL of THF-d_8 (see section 5.6). Spectra were also recorded in the dark first, followed by measurements under irradiation with red light, green light and after the thermal reverse reaction in the dark at room temperature with subsequent re-cooling of the reaction mixture. The recorded spectra can be seen in Figure S36.

Figure S36: $^{19}F\{^1H\}$ NMR spectra starting with ***E*-5B** at −40 °C before irradiation, after irradiation with red light, after irradiation with green light and after heating the sample to room temperature with re-cooling.

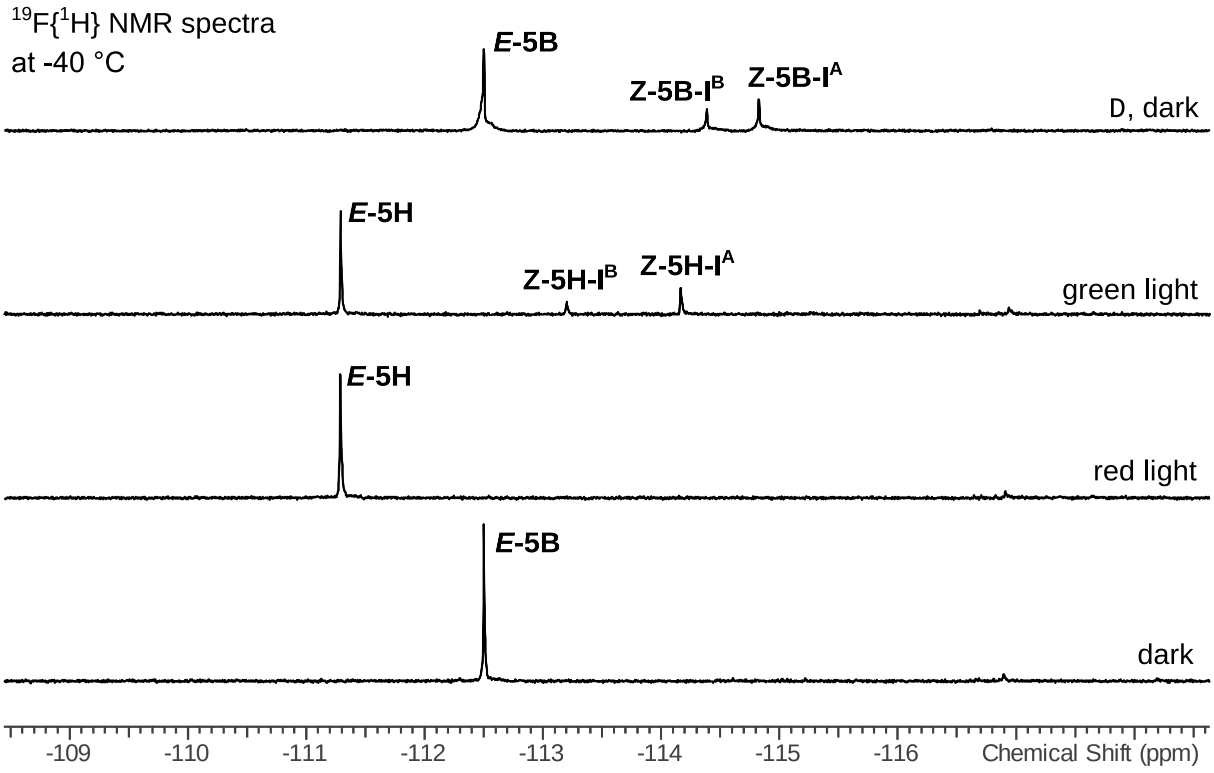

The first recorded spectrum (in the dark at −40 °C) clearly shows one singlet at −112.6 ppm that can be assigned to starting material ***E*-5B**, the different isomers I^A and I^B observed in the $^{31}P\{^1H\}$ NMR spectra (section 5.6) cannot be distinguished in this instance. Irradiation with red light leads to the formation of housane type species ***E*-**

5H, indicated by the presence of one singlet at −111.4 ppm, the thermal reverse reaction to form ***E*-5B** again can also be carried out in the dark. If the irradiation is continued with light from a green laser diode the partial *E* to *Z* isomerisation at the azobenzene takes place and compounds ***E*-5H** and ***Z*-5H** can be observed in the corresponding spectrum. Interestingly for ***Z*-5H** two singlets with chemical shifts of −114.3 ppm and −113.3 ppm can be observed as the result of different isomers of ***Z*-5H** forming during the switching process (for further detail on the possible isomers see section 6). After performing the thermal reverse reaction in the dark at room temperature and re-cooling of the reaction mixture to −40 °C the $^{19}F\{^{1}H\}$ NMR spectrum shows that a mixture of compounds ***E*-5B** and ***Z*-5B** is present at this stage as the housane type species react back towards the biradical compounds. As the *Z* to *E* isomerisation at the azobenzene moiety is not induced thermally at this temperature both species can be observed. Newly formed ***Z*-5B** also shows two singlets at −114.9 ppm and −114.5 ppm as there are still two isomers present as a result of the switching process conducted earlier (again for further details see section 6). Irradiation of the mixture of ***E*-5B** and ***Z*-5B** with red light can then lead to the re-formation of housanes ***E*-5H** and ***Z*-5H**.

The attempt to induce the *Z* to *E* isomerisation reaction on the housane type species (***Z*-5H→*E*-5H**) via irradiation of the sample with blue light was also monitored with $^{19}F\{^{1}H\}$ NMR spectroscopy. For this also the mixture of compounds ***E*-5H** and ***Z*-5H** (after irradiation with green light) was treated with blue light in order to receive ***E*-5H** as the reaction product, the recorded spectra are depicted in Figure S37.

As with the $^{31}P\{^{1}H\}$ NMR spectra (Figure S31) the intensity of the signals of ***Z*-5H** also decreases slightly in the $^{19}F\{^{1}H\}$ NMR spectra. The two singlets of species ***Z*-5H** at −114.3 ppm and −113.3 ppm however still can be identified, again indicating that the conversion is incomplete. Most likely the creation of a photo stationary state (PSS) occurs in which both *Z* to *E* as well as *E* to *Z* isomerisation reactions take place at the same time (*i.e.* both species ***E*-5H** and ***Z*-5H** are constantly present in the mixture and therefore detectable in the $^{19}F\{^{1}H\}$ NMR spectra).

Figure S37: $^{19}F\{^1H\}$ NMR spectra of attempted switching process from ***Z*-5H** to ***E*-5H** at −40 °C under irradiation with blue light.

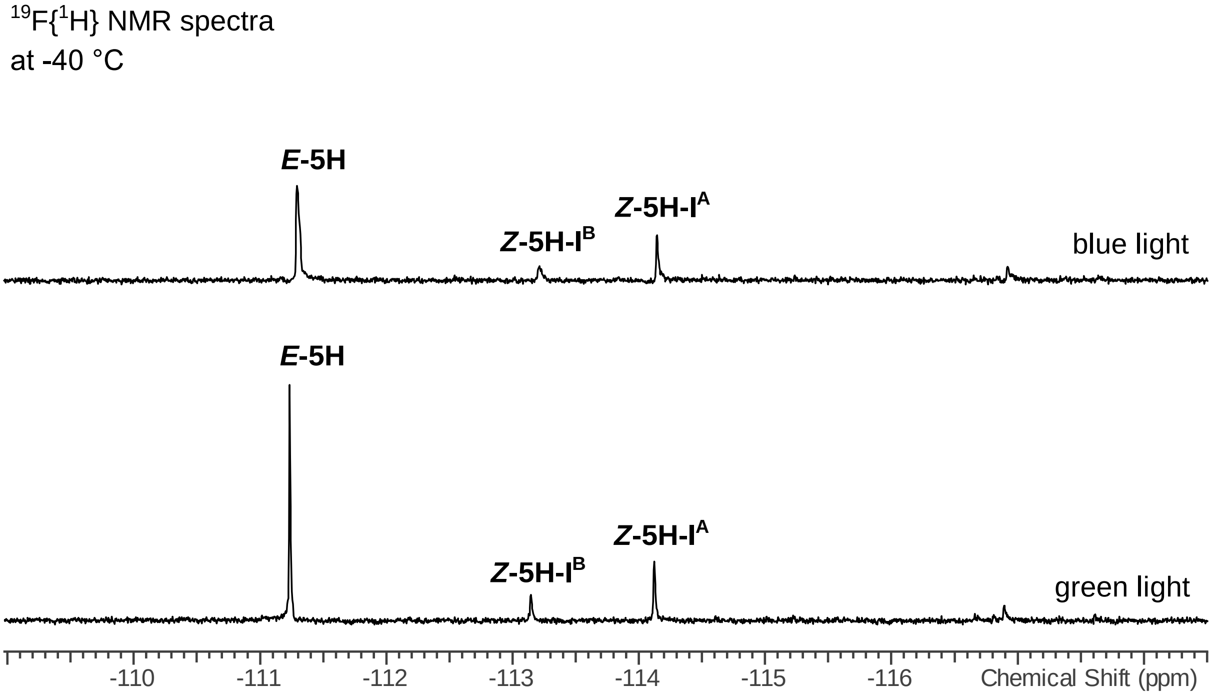

5.9 ^{19}F NMR data of switching process starting with *E*-5B at room temperature

The same solution as with the experiments described in section 5.7 was used to record $^{19}F\{^1H\}$ NMR spectra at 235.36 MHz during the switching process at room temperature (approx. 25 °C). The switching process was conducted in the same way as the experiments carried out at low temperatures (see section 5.8). The recorded spectra are depicted in Figure S36.

The first recorded spectrum (in the dark) again shows one singlet at −113.1 ppm that can be assigned to starting material ***E*-5B**, the different isomers I^A and I^B observed in the $^{31}P\{^1H\}$ NMR spectra (section 5.7) also cannot be distinguished in this instance.

Figure S38: $^{19}F\{^1H\}$ NMR spectra starting with ***E*-5B** at room temperature before irradiation, after irradiation with red light, after irradiation with green light and after performing the thermal reverse reaction in the dark.

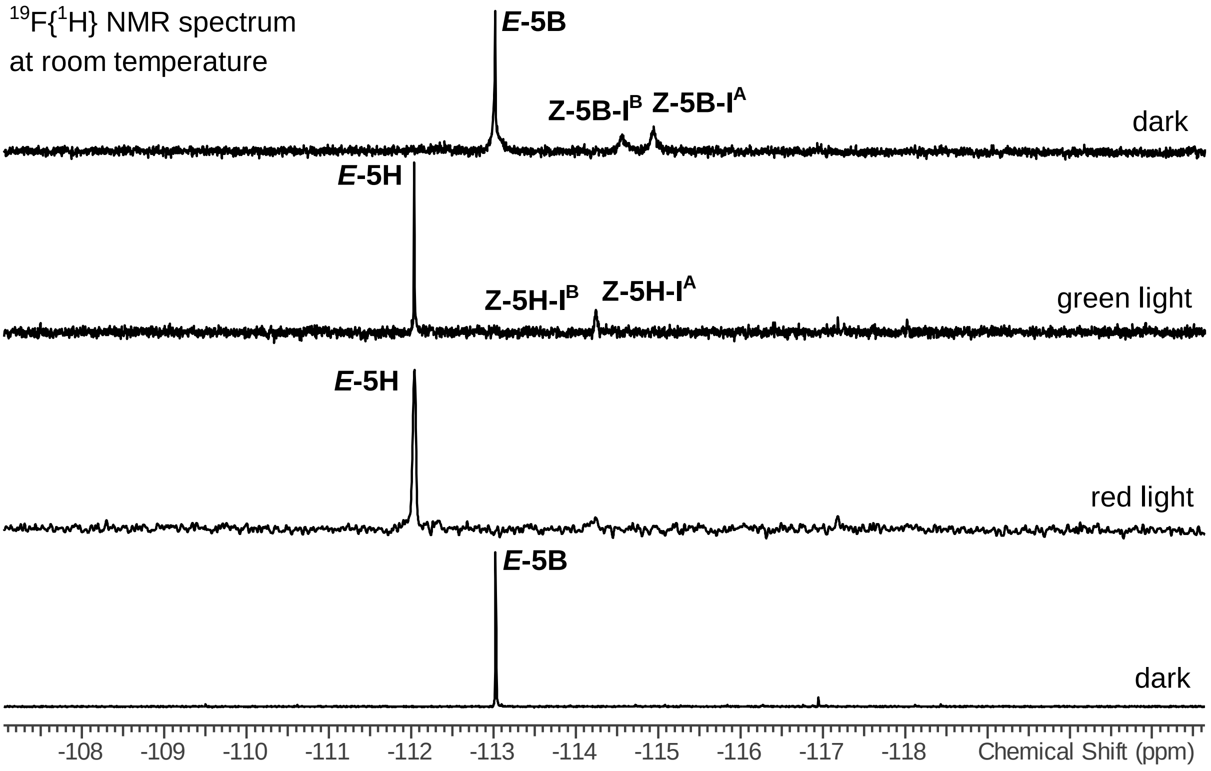

Again, irradiation with red light leads to the formation of housane type species ***E*-5H** (singlet at −112.1 ppm), the thermal reverse reaction at this stage can also be carried out. The input of green light leads to partial *E* to *Z* isomerisation at the azobenzene and compounds ***E*-5H** and ***Z*-5H** can be observed in the corresponding spectrum. The signals are significantly broadened at room temperature. Again, for ***Z*-5H** two singlets with chemical shifts of −114.3 ppm and approx. −113.9 ppm can be observed as the result of different isomers of ***Z*-5H** forming during the switching process (for further detail on the possible isomers see section 6). After performing the thermal reverse reaction in the dark the corresponding spectrum shows the mixture of compounds ***E*-5B** and newly formed ***Z*-5B**. As the *Z* to *E* isomerisation at the azobenzene moiety is not induced thermally in this timeframe both species can be observed. ***Z*-5B** also shows two singlets at −115.1 ppm and −114.7 ppm as there are still two isomers present as a result of the switching process conducted earlier (see section 6).

5.10 ^{31}P and ^{19}F NMR data of *E*-5B, *E*-5H, *Z*-5H and *Z*-5B.

The following tables contain experimental ^{31}P NMR and ^{19}F NMR data. Calculated values of the lowest energy isomer (at 25 °C) are given in brackets for comparison (GIAO method, PBE0-D3/def2-SVP, see section 6 for further details).

Table S3. ^{31}P NMR data of isomers of ***E*-5B**, ***E*-5H**, ***Z*-5H** and ***Z*-5B**.

	δ_{exp} (P1, N*P*N) [ppm]	δ_{calc} (P1, N*P*N) [ppm]	δ_{exp} (P2, N*P*C) [ppm]	δ_{calc} (P2, N*P*C) [ppm]	J_{exp} [Hz]	J_{calc} [Hz]
***E*-5B-I^{A}** [1]	257.5	237.9	219.7	219.0	J_{PP} = 127	J_{PP} = 93
***E*-5B-I^{A}** [2]	253.8		219.2		J_{PP} = 120	
***E*-5B-I^{B}** [1]	258.8		222.0		J_{PP} = 136	
***E*-5B-I^{B}** [2]	255.1		221.3		J_{PP} = 130	
***E*-5H-I^{A}** [1]	−66.2	−67.7	−129.9	−155.2	J_{PP} = −65	J_{PP} = −52
***E*-5H-I^{A}** [2]	−67.1		−131.9		J_{PP} = −62	
***E*-5H-I^{B}** [1]	−66.3		−128.5		J_{PP} = −65	
***E*-5H-I^{B}** [2]	−67.7		−130.8		J_{PP} = −62	
***Z*-5H-I^{A}** [1]	−66.2	−66.1	−129.9	−151.6	J_{PP} = −65	J_{PP} = −58
***Z*-5H-I^{A}** [2]	−67.2		−132.5		J_{PP} = −62	
***Z*-5H-I^{B}** [1]	−66.3		−127.9		J_{PP} = −65	
***Z*-5H-I^{B}** [2]	−55.9		−129.2		J_{PP} = −62	
***Z*-5B-I^{A}** [1]	257.0	237.1	219.4	221.9	J_{PP} = 127	J_{PP} = 119
***Z*-5B-I^{A}** [2]	253.3		219.5		J_{PP} = 120	
***Z*-5B-I^{B}** [1]	256.7		221.5		J_{PP} = 136	
***Z*-5B-I^{B}** [2]	255.2		221.5		J_{PP} = 130	

[1] experimental values at room temperature; [2] experimental values at −40 °C.

Table S4. ^{19}F NMR data of isomers of ***E*-5B**, ***E*-5H**, ***Z*-5H** and ***Z*-5B**.

	δ_{exp} [ppm] (r.t.)	δ_{exp} [ppm] (−40 °C)	δ_{calc} [ppm] (r.t.)
***E*-5B**	−113.1	−112.6	−98.8
***E*-5H**	−112.1	−111.4	−96.1
***Z*-5H-I^{A}**	−114.3	−114.3	−99.2
***Z*-5H-I^{B}**	−113.9	−113.3	−99.2
***Z*-5B-I^{A}**	−115.1	−114.9	−100.7
***Z*-5B-I^{B}**	−114.7	−114.5	−100.7

6 Computational details

6.1 General remarks

Computations were carried out using Gaussian09[11] or ORCA 5.0.1[12].

Structure optimizations employed the DFT functional PBE[13–15] in conjunction with Grimme's dispersion correction D3(BJ)[16,17] and the def2-TZVP basis set[18] (notation PBE-D3/def2-TZVP). The resolution-of-identity (RI) approximation was applied for the pure functional PBE, using Weigend's accurate Coulomb-fitting basis set (W06).[19] All structures were fully optimized and confirmed as minima by frequency analyses. Transition state (TS) structures for the *E/Z* isomerisation reactions were optimized at the UPBE-D3/def2-TZVP level of theory and confirmed by frequency analyses (one imaginary frequency).

Chemical shifts and coupling constants were derived by the GIAO method[20–24] at the PBE0-D3/def2-SVP level of theory.[13–15,25] The calculated absolute shifts for ^{31}P nuclei ($\sigma_{calc,X}$) were referenced to the experimental absolute shift of 85% H_3PO_4 in the gas phase ($\sigma_{ref,1}$ = 328.35 ppm),[26] using PH_3 ($\sigma_{ref,2}$ = 594.45 ppm) as a secondary standard:[27]

$$\begin{aligned}\delta_{calc,X} &= (\sigma_{ref,1} - \sigma_{ref,2}) - (\sigma_{calc,X} - \sigma_{calc,PH_3}) \\ &= \sigma_{calc,PH_3} - \sigma_{calc,X} - 266.1 \text{ ppm}\end{aligned}$$

At the PBE0-D3/def2-SVP level of theory, σ_{calc,PH_3} amounts to +629.17 ppm. Spin-spin coupling constants were calculated in a two-step process, using a modified basis set for computation of the Fermi contact term.[28]

The calculated absolute shifts for ^{19}F nuclei ($\sigma_{calc,X}$) were referenced to the calculated absolute shift of $CFCl_3$ (σ_{ref}) in the gas phase. At the PBE0-D3/def2-SVP level of theory, σ_{ref} amounts to +200.53 ppm.

$$\delta_{calc,X} = (\sigma_{ref}) - (\sigma_{calc,X})$$

More accurate electronic energies for optimized structures were computed by single-point DLPNO-CCSD(T)[29–32] calculations employing the def2-TZVP basis set and def2-TZVP/C correlation fitting basis (notation: DLPNO-CCSD(T)/def2-TZVP//PBE-D3/def2-TZVP).[18,25] Thermodynamic quantities at this level of theory were calculated using the DLPNO-CCSD(T) single point energy and the thermal corrections at the PBE-D3/def2-TZVP level of theory. The T_1 diagnostic was evaluated to ensure reliable results (empirically, CCSD(T) results are considered reliable if $T_1 < 0.02$).[33] Nonetheless, it should be noted that many of the species discussed here possess at least a small amount of multi-reference character (biradical character), so the use of single-reference methods always entails some loss of accuracy and should be regarded as an approximation!

Please note that all computations were carried out for single, isolated molecules in the gas phase (ideal gas approximation). There may well be significant differences between gas phase and condensed phase.

6.2 Isomer search

An isomer search was conducted using xTB version 6.5.1[34]. Calculations were carried out with the semiempirical tight-binding based quantum chemistry method GFN2-xTB.[34,35] The starting structure was optimized first at the GFN2-xTB level of theory. For the isomer search the low-energy chemical space was explored using the computer code CREST.[36,37] An evaluation of the found ensemble of isomers was performed with the CENSO algorithm[38]. This included a cheap pre-screening and a pre-screening (B97-D3/def2-SV(P) level of theory)[39] as well as an optimization process (PBE-D3/def2-TZVP level of theory) followed by manual comparison of the different isomers. Finally, the selected structures were optimized using the methods described above in section 6.1. The optimized isomers for each compound (***E*-5B**, ***E*-5H**, ***Z*-5H** and ***Z*-5B**) can be seen below starting with Figure S39.

Figure S39: Found isomers of ***E*-5B** (Gibbs free energies in relation to the lowest energy isomer at the DLPNO-CCSD(T)/def2-TZVP//PBE-D3/def2-TZVP level of theory).

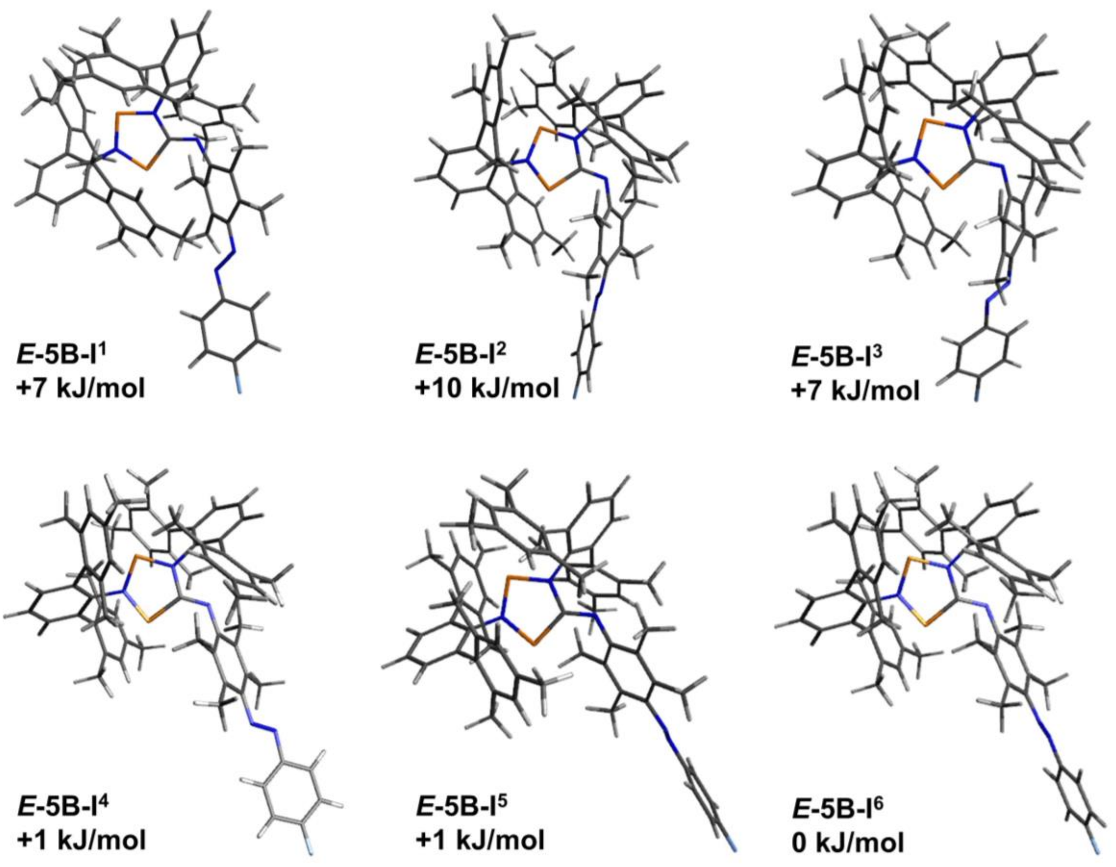

Figure S40: Found isomers of ***E*-5H** (Gibbs free energies in relation to the lowest energy isomer at the DLPNO-CCSD(T)/def2-TZVP//PBE-D3/def2-TZVP level of theory).

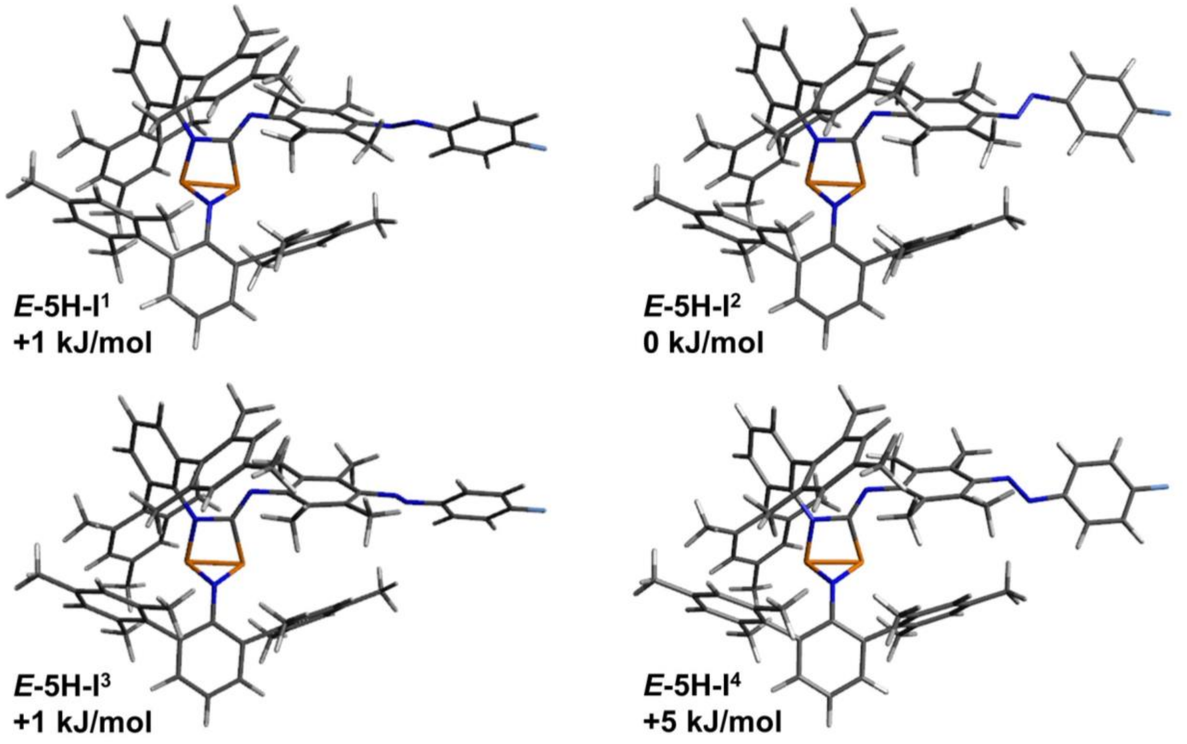

Figure S41: Found isomers of ***Z*-5H** (Gibbs free energies in relation to the lowest energy isomer at the DLPNO-CCSD(T)/def2-TZVP//PBE-D3/def2-TZVP level of theory).

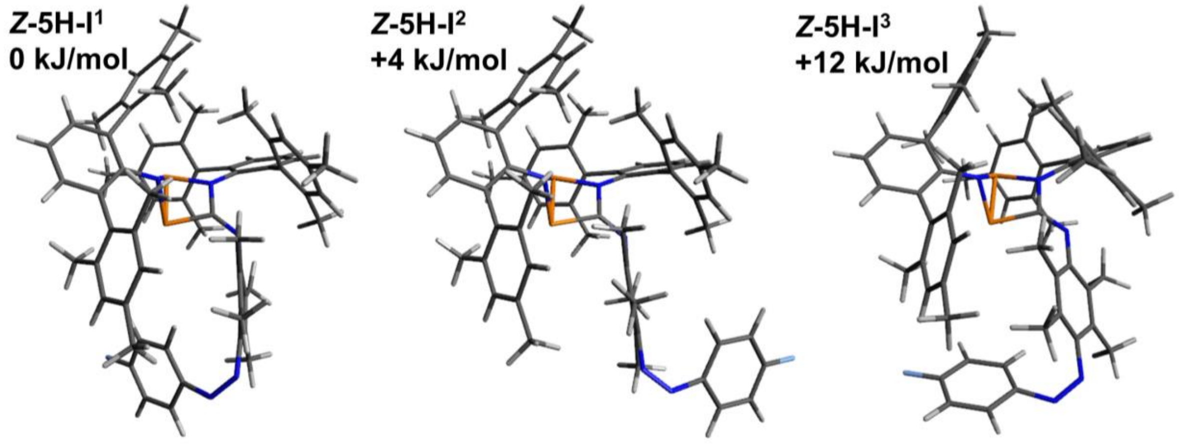

Figure S42: Found isomers of ***Z*-5B** (Gibbs free energies in relation to the lowest energy isomer at the DLPNO-CCSD(T)/def2-TZVP//PBE-D3/def2-TZVP level of theory).

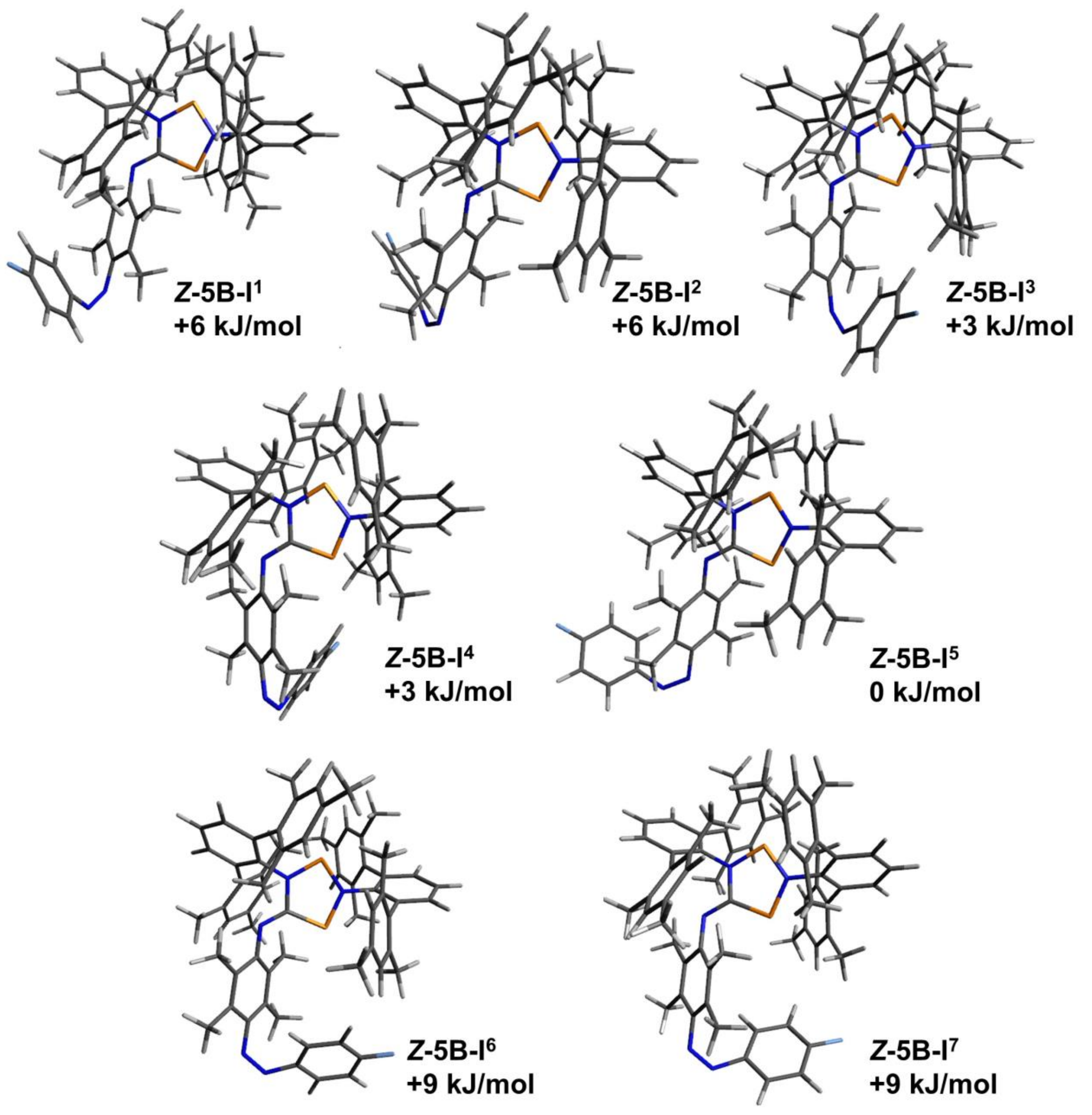

In the following table a comparison of experimental ^{19}F NMR shifts (at room temperature) and calculated chemical shifts for each isomer (at 25 °C) is presented (GIAO method, PBE0-D3/def2-SVP, see section 6 for further details).

Table S5. ^{19}F NMR data of isomers of ***E*-5B**, ***E*-5H**, ***Z*-5H** and ***Z*-5B** and all found isomers.

	**isomer**	***E*-5B**	***E*-5H**	***Z*-5H**	***Z*-5B**
**calc.**	**1**	−98.4	−96.2	−99.2	−104.8
	**2**	−98.3	−96.1	−96.1	−104.8
	**3**	−98.4	−95.9	−105.9	−109.1
	**4**	−98.9	−95.7		−109.1
	**5**	−98.8			−100.7
	**6**	−98.8			−106.4
	**7**				−106.4
**exp.**	**A**	−113.1	−112.1	−114.3	−115.1
	**B**			−113.9	−114.7

6.3 Natural transition orbitals (NTOs)

TD-DFT calculations[40–42] were performed at the PBE0-D3/def2-TZVP level of theory starting with the optimized structures of compounds ***E*-5B**, ***E*-5H**, ***Z*-5H** and ***Z*-5B** (optimized at the PBE-D3/def2-TZVP level of theory as described in section 6.1). Afterwards the donor and acceptor NTOs of states S1 to S4 were plotted using Multiwfn.[43] The NTOs of compound ***E*-5B** are depicted in Figure S43 as an example alongside the corresponding wavelengths for the transition. Only NTOs contributing with a coefficient of more than 0.10 were considered.

For ***E*-5B** the transitions are in good agreement with experimentally observed UV/Vis absorption bands (section 5.4). The transition for the S1 state for example corresponds with a calculated wavelength of 633 nm in contrast to the experimentally observed broad absorption maximum located at around 644 nm for the housane formation (Figure S43, top). The S2 state can be regarded as an nπ* transition with the NTOs located mostly at the azobenzene fragment. This transition comes along with a calculated wavelength of 519 nm (experimental value of 490 nm for the broad absorption maximum), i.e. irradiation with green light (520 nm) should be able to induce this transition (Figure S43, top middle). The calculated excitation wavelengths of the S3 and S4 states are 478 nm and 473 nm, with the NTOs depicted in the bottom of Figure S43.

Figure S43: Natural transition orbitals (NTOs) of ***E*-5B**. States S1 to S4 (with contributing coefficients) are depicted alongside the corresponding wavelengths.

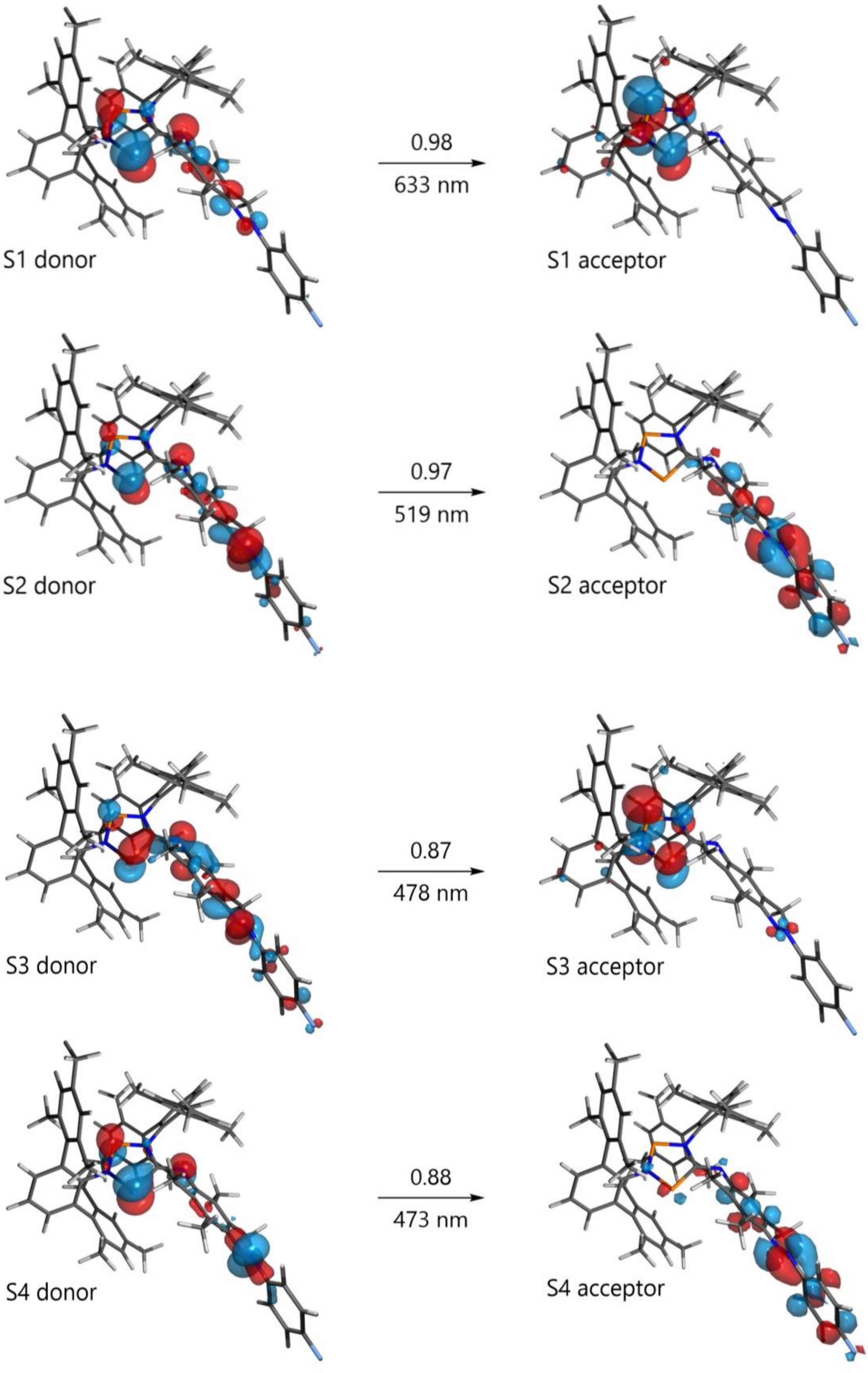

6.4 Summary of calculated data

Table S6. Summary of calculated data, including electronic energies, thermal corrections and chemical shifts.

Compd	PG	Opt. method	E_{tot}[a]	ΔG[b]	$E_{CCSD(T)}$[c]	T_1	δ (P1) [ppm]	δ (P2) [ppm]	δ (F) [ppm]
***E*-5B-I[1]**	C_1	PBE-D3 def2-TZVP	−3570.2889	0.9913	−3566.7448	0.011	245.5	229.2	−98.4
***E*-5B-I[2]**	C_1		−3570.2891	0.9927	−3566.7449	0.011	244.4	228.9	−98.3
***E*-5B-I[3]**	C_1		−3570.2889	0.9914	−3566.7448	0.011	245.5	229.2	−98.4
***E*-5B-I[4]**	C_1		−3570.2884	0.9909	−3566.7466	0.011	237.9	220.7	−98.9
***E*-5B-I[5]**	C_1		−3570.2880	0.9900	−3566.7462	0.011	237.9	219.0	−98.8
***E*-5B-I[6]**	C_1		−3570.2880	0.9900	−3566.7462	0.011	237.9	219.0	−98.8
***E*-5H-I[1]**	C_1		−3570.2769	0.9936	−3566.7357	0.011	−69.4	−157.9	−96.2
***E*-5H-I[2]**	C_1		−3570.2772	0.9929	−3566.7354	0.011	−67.7	−155.2	−96.1
***E*-5H-I[3]**	C_1		−3570.2762	0.9924	−3566.7344	0.011	−69.4	−156.5	−95.9
***E*-5H-I[4]**	C_1		−3570.2771	0.9939	−3566.7345	0.011	−68.9	−151.6	−95.7
***Z*-5H-I[1]**	C_1		−3570.2667	0.9954	−3566.7297	0.011	−66.1	−151.6	−99.2
***Z*-5H-I[2]**	C_1		−3570.2644	0.9941	−3566.7269	0.011	−65.5	−156.6	−96.1
***Z*-5H-I[3]**	C_1		−3570.2649	0.9962	−3566.7260	0.011	−72.5	−133.1	−105.9
***Z*-5B-I[1]**	C_1		−3570.2769	0.9939	−3566.7383	0.011	237.0	219.6	−104.8
***Z*-5B-I[2]**	C_1		−3570.2769	0.9939	−3566.7384	0.011	237.1	219.6	−104.8
***Z*-5B-I[3]**	C_1		−3570.2777	0.9934	−3566.7389	0.011	240.3	220.9	−109.1
***Z*-5B-I[4]**	C_1		−3570.2777	0.9934	−3566.7389	0.011	240.3	220.9	−109.1
***Z*-5B-I[5]**	C_1		−3570.2754	0.9906	−3566.7374	0.011	237.1	221.9	−100.7
***Z*-5B-I[6]**	C_1		−3570.2769	0.9944	−3566.7374	0.011	239.8	222.2	−106.4
***Z*-5B-I[7]**	C_1		−3570.2769	0.9944	−3566.7374	0.011	239.9	222.2	−106.4
***E*-11**	C_1		−920.7224	0.2321	−919.8348	0.011	–	–	−90.8
***Z*-11**	C_1		−920.7101	0.2324	−919.8239	0.011	–	–	−94.3
**TS-11**	C_1		−920.6792	0.2295	–	–	–	–	–
**TS-B**	C_1		−3570.2462	0.9878	–	–	–	–	–
**TS-H**	C_1		−3570.2349	0.9923	–	–	–	–	–

[a] Total SCF energy in a.u.; [b] thermal correction to Gibbs energy in a.u. (298 K unless stated otherwise); [c] single-point DLPNO-CCSD(T)/def2-TZVP energy.

6.5 Optimized structures (.xyz-files)

6.5.1 PH_3

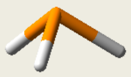

```
4
PH3 @ PBE-D3/def2-TZVP
P           0.00000        0.00000        0.13012
H           0.00000        1.19618       -0.65060
H           1.03592       -0.59809       -0.65060
H          -1.03592       -0.59809       -0.65060
```

6.5.2 $CFCl_3$

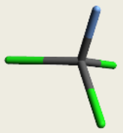

```
5
CFCl3 @ PBE-D3/def2-TZVP
C          -0.00001        0.00011        0.25116
Cl          1.45762        0.84878       -0.31240
Cl         -1.46409        0.83756       -0.31240
F          -0.00001        0.00040        1.60236
Cl          0.00648       -1.68659       -0.31215
```

6.5.3 *E*-5B-I^{1}

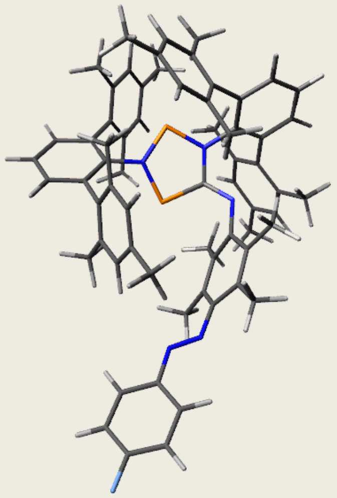

```
139
E-5B-I¹ @ PBE-D3/def2-TZVP
P           0.17988       -0.17667       -0.83002
N          -1.25471       -1.16639       -0.80723
C          -0.51004        0.98357        0.34567
P          -2.61991       -0.67471        0.04635
C          -1.23532       -2.41804       -1.52218
N          -1.87871        0.74701        0.62892
N           0.14617        1.89148        1.02512
C          -2.31598       -2.76870       -2.36165
C          -0.11466       -3.27897       -1.39299
C          -2.62402        1.61252        1.50654
C           1.50444        2.01764        0.78989
C          -2.25549       -3.97651       -3.07068
C          -3.51975       -1.90965       -2.56368
C          -0.10101       -4.47078       -2.12909
C           1.02817       -3.00045       -0.47343
C          -2.72669        2.99394        1.22048
C          -3.26404        1.06180        2.63990
C           2.07478        2.07733       -0.52026
C           2.36130        2.04422        1.92386
H          -3.09101       -4.23207       -3.72470
C          -1.15721       -4.82427       -2.96415
C          -3.46290       -0.83079       -3.47259
C          -4.73220       -2.23286       -1.91790
H           0.76036       -5.13147       -2.01802
C           0.85838       -3.19760        0.91182
C           2.26857       -2.55291       -0.97940
C          -3.43487        3.80672        2.11602
C          -2.19043        3.63746       -0.01177
C          -3.96963        1.91670        3.49892
```

C	-3.23929	-0.39074	2.98610
C	3.44050	1.80765	-0.69659
C	1.24058	2.51911	-1.69725
C	3.72961	1.82688	1.75451
C	1.78163	2.20138	3.30294
H	-1.12569	-5.75785	-3.52771
C	-4.62458	-0.09423	-3.71725
C	-2.17268	-0.46227	-4.15382
C	-5.86302	-1.45027	-2.17484
C	-4.81029	-3.36749	-0.93101
C	1.90907	-2.87190	1.77467
C	-0.43445	-3.73764	1.46092
C	3.28890	-2.23033	-0.08062
C	2.47790	-2.33502	-2.45334
H	-3.51740	4.87041	1.88873
C	-4.04784	3.28295	3.25056
C	-1.19292	4.63494	0.08649
C	-2.76899	3.34403	-1.26532
H	-4.45123	1.48462	4.37779
C	-2.12231	-0.93870	3.65350
C	-4.38058	-1.18708	2.74035
C	4.25071	1.57358	0.45069
C	4.01558	1.85398	-2.08662
H	0.20323	2.71280	-1.41115
H	1.64692	3.45892	-2.10659
H	1.23922	1.78562	-2.51855
C	4.62000	1.75336	2.96537
H	1.88043	1.27709	3.89568
H	2.30590	2.99100	3.86221
H	0.71682	2.44937	3.24569
H	-4.57964	0.73650	-4.42691
C	-5.83207	-0.37686	-3.06992
H	-2.34069	0.30148	-4.92400
H	-1.44427	-0.05811	-3.43178
H	-1.69869	-1.33596	-4.62487
H	-6.79724	-1.69027	-1.65981
H	-4.50888	-4.32487	-1.37978
H	-4.13878	-3.19724	-0.07294
H	-5.83086	-3.47416	-0.54113
H	1.76776	-3.00928	2.85073
C	3.12519	-2.36806	1.30213
H	-1.23968	-2.98786	1.42542
H	-0.78088	-4.61111	0.88904
H	-0.31632	-4.03424	2.51074
H	4.22445	-1.81848	-0.46906
H	1.73376	-1.62846	-2.85552
H	3.47673	-1.92120	-2.64309
H	2.37430	-3.26727	-3.02821
H	-4.59574	3.93610	3.93136
C	-0.77028	5.28586	-1.07597
C	-0.57194	5.00915	1.40630
C	-2.30409	4.01500	-2.40357
C	-3.90841	2.37264	-1.40266
C	-2.16517	-2.27766	4.05096
C	-0.90927	-0.09929	3.93766
C	-4.36807	-2.52813	3.13782
C	-5.58330	-0.62755	2.02842
N	5.56884	1.13101	0.42180
H	3.81819	0.91760	-2.63245
H	3.56029	2.67100	-2.66402

```
H          5.10190       1.98310      -2.07780
H          4.52136       2.66568       3.57348
H          4.33916       0.91130       3.61869
H          5.66675       1.62451       2.67474
C         -7.04938       0.47574      -3.30430
C          4.24129      -2.01403       2.24657
H          0.01513       6.04254      -0.99536
C         -1.30015       4.98285      -2.33408
H         -0.47920       4.13212       2.05761
H          0.42935       5.43305       1.25143
H         -1.17256       5.76546       1.93622
H         -2.75883       3.78300      -3.37091
H         -4.60386       2.43672      -0.55410
H         -4.46954       2.56236      -2.32676
H         -3.56066       1.32741      -1.45332
H         -1.30070      -2.69656       4.57314
C         -3.27006      -3.09500       3.79184
H         -0.14789      -0.68007       4.47430
H         -0.45580       0.28861       3.01386
H         -1.16513       0.78215       4.54545
H         -5.24564      -3.14679       2.93078
H         -5.31874      -0.26717       1.02038
H         -6.36092      -1.39445       1.91783
H         -6.01570       0.23009       2.56357
N          5.90662       0.40382      -0.57211
H         -7.11531       0.80364      -4.35129
H         -7.97300      -0.06222      -3.05156
H         -7.01565       1.38420      -2.68090
H          4.85758      -1.19750       1.84770
H          3.85206      -1.70831       3.22792
H          4.90637      -2.87725       2.41134
C         -0.77914       5.65441      -3.57559
C         -3.25854      -4.55006       4.17375
C          7.26219       0.01201      -0.56803
H         -0.47310       6.69071      -3.37505
H          0.10483       5.12304      -3.96488
H         -1.53302       5.66464      -4.37486
H         -2.73319      -4.71208       5.12549
H         -4.27711      -4.95023       4.26812
H         -2.73759      -5.14960       3.40933
C          8.24890       0.54020       0.28728
C          7.62044      -0.96864      -1.50801
H          7.96255       1.31353       0.99977
C          9.55954       0.08402       0.21236
C          8.92944      -1.43730      -1.58840
H          6.84381      -1.35769      -2.16847
H         10.34249       0.48141       0.85935
C          9.87765      -0.90107      -0.72286
H          9.22320      -2.20218      -2.30718
F         11.15952      -1.34250      -0.79695
```

6.5.4 *E*-5B-I^2

```
139
E-5B-I² @ PBE-D3/def2-TZVP
P         -0.07883      -0.06813      -0.98762
N          1.36207      -1.04420      -0.99276
C          0.48667       0.89182       0.41395
```

P	2.63817	-0.69745	0.04585
C	1.42416	-2.16579	-1.89657
N	1.83034	0.61447	0.77631
N	-0.23754	1.68074	1.16579
C	2.54079	-2.32735	-2.74464
C	0.34251	-3.08233	-1.93965
C	2.47863	1.33327	1.84285
C	-1.57194	1.85213	0.83649
C	2.55584	-3.40984	-3.63592
C	3.70209	-1.38994	-2.76884
C	0.40228	-4.14088	-2.85429
C	-0.82418	-2.98528	-1.01443
C	3.01590	0.61680	2.93543
C	2.58759	2.74231	1.78076
C	-2.52714	1.68366	1.88311
C	-2.02374	2.15582	-0.47985
H	3.41806	-3.52274	-4.29546
C	1.49629	-4.30987	-3.69937
C	4.91117	-1.75130	-2.13701
C	3.61466	-0.18805	-3.50328
H	-0.42873	-4.84777	-2.87790
C	-2.06608	-2.48900	-1.46603
C	-0.66473	-3.37938	0.32949
C	3.62222	1.33234	3.97868
C	2.98747	-0.87150	3.06059
C	3.19233	3.41050	2.85361
C	2.16816	3.56263	0.61002
C	-3.87852	1.50800	1.57753
C	-2.05187	1.63284	3.31145
C	-3.37934	1.97478	-0.79339
C	-1.09837	2.73275	-1.51950
H	1.52394	-5.14228	-4.40404
C	6.00872	-0.88981	-2.23589
C	5.02303	-3.02491	-1.34109
C	4.74500	0.62932	-3.59449
C	2.32834	0.21968	-4.16974
C	-3.09613	-2.30607	-0.53865
C	-2.26828	-2.07951	-2.89961
C	-1.72329	-3.18318	1.22117
C	0.62334	-3.99710	0.80376
H	4.02508	0.77166	4.82390
C	3.70113	2.72044	3.95014
C	1.83432	-1.51744	3.55616
C	4.16231	-1.61443	2.80470
H	3.28008	4.49639	2.79876
C	1.15276	4.53645	0.75212
C	2.87549	3.45719	-0.60691
C	-4.28580	1.53639	0.20790
C	-4.86617	1.34771	2.70313
H	-0.96993	1.79154	3.35616
H	-2.54722	2.40498	3.92034
H	-2.27702	0.66399	3.78482
C	-3.83816	2.18035	-2.21122
H	-1.02369	2.10115	-2.41972
H	-1.47956	3.71272	-1.85075
H	-0.08867	2.88756	-1.12949
H	6.94028	-1.16458	-1.73346
C	5.94874	0.30402	-2.96137
H	4.29261	-3.04895	-0.51567
H	4.82506	-3.91292	-1.95896

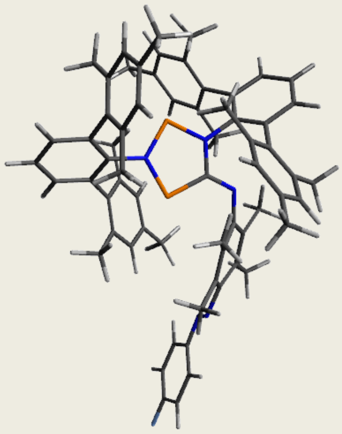

H	6.02569	-3.12450	-0.90555
H	4.67713	1.55634	-4.17060
H	1.55858	0.47633	-3.42333
H	2.48070	1.09776	-4.81044
H	1.91472	-0.59387	-4.78376
H	-4.03956	-1.86888	-0.87574
C	-2.93930	-2.62336	0.81464
H	-1.54425	-1.30181	-3.19215
H	-2.12771	-2.92192	-3.59285
H	-3.27833	-1.67657	-3.04840
H	-1.58869	-3.46920	2.26846
H	0.48507	-4.47835	1.77981
H	0.99277	-4.74997	0.09242
H	1.41979	-3.24648	0.92523
H	4.17015	3.26252	4.77261
C	1.87975	-2.89532	3.78604
C	0.58076	-0.74006	3.83983
C	4.15154	-2.99533	3.02580
C	5.40640	-0.94872	2.27951
C	0.84120	5.35240	-0.33916
C	0.39791	4.71395	2.04310
C	2.51804	4.28845	-1.67614
C	4.04090	2.52034	-0.76616
N	-5.54929	1.18460	-0.25836
H	-4.58048	1.98219	3.55385
H	-5.88311	1.60621	2.39204
H	-4.90924	0.30976	3.06781
H	-4.92290	2.06565	-2.29243
H	-3.54819	3.17879	-2.57322
H	-3.36206	1.45258	-2.88914
C	7.13124	1.23208	-3.02522
C	-4.05916	-2.39461	1.79262
H	0.98798	-3.38974	4.18093
C	3.02199	-3.65611	3.51859
H	0.18598	-0.26261	2.93128
H	-0.20161	-1.39201	4.24939
H	0.76599	0.07343	4.55771
H	5.05758	-3.56958	2.81335
H	5.78377	-0.18181	2.97148
H	6.20195	-1.68688	2.11433
H	5.21432	-0.43793	1.32151
H	0.04195	6.09058	-0.22869
C	1.49885	5.23659	-1.56791
H	-0.58616	5.16273	1.85210
H	0.24746	3.75063	2.54458
H	0.93516	5.37942	2.73746
H	3.07197	4.20136	-2.61528
H	4.69643	2.85520	-1.58011
H	4.63643	2.45089	0.15472
H	3.72081	1.49626	-1.02125
N	-6.19841	0.33562	0.43877
H	7.14144	1.80667	-3.96170
H	7.10343	1.95828	-2.19624
H	8.07952	0.68304	-2.94562
H	-4.72305	-1.58461	1.45777
H	-4.67223	-3.30378	1.90796
H	-3.66910	-2.13728	2.78810
C	3.02074	-5.14726	3.71697
C	1.09643	6.08473	-2.74373
C	-7.48708	0.04171	-0.05575

H	4.03050	-5.52765	3.92347
H	2.36299	-5.44212	4.54636
H	2.65522	-5.66076	2.81247
H	0.25604	5.62360	-3.28814
H	0.76811	7.08431	-2.42602
H	1.92355	6.20273	-3.45729
C	-8.14066	-1.04851	0.54239
C	-8.14245	0.76722	-1.06957
H	-7.62048	-1.59154	1.33296
C	-9.41495	-1.43093	0.13046
C	-9.41644	0.39811	-1.48467
H	-7.63246	1.62242	-1.51253
H	-9.93404	-2.27793	0.57894
C	-10.03070	-0.69857	-0.87961
H	-9.94729	0.94765	-2.26295
F	-11.27690	-1.05505	-1.28396

6.5.5 *E*-5B-I^{3}

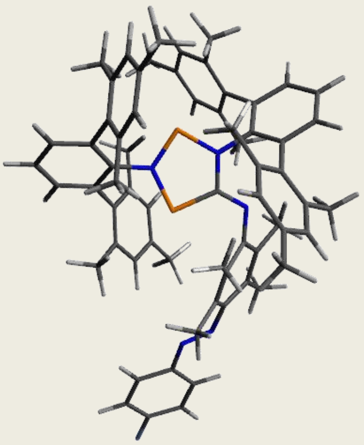

139			
***E*-5B-I^{3}** @ PBE-D3/def2-TZVP			
P	-0.17971	-0.17647	-0.83044
N	1.25477	-1.16629	-0.80750
C	0.51001	0.98347	0.34565
P	2.61981	-0.67491	0.04649
C	1.23541	-2.41782	-1.52267
N	1.87861	0.74679	0.62913
N	-0.14627	1.89128	1.02517
C	2.31602	-2.76830	-2.36227
C	0.11480	-3.27881	-1.39348
C	2.62382	1.61217	1.50693
C	-1.50450	2.01758	0.78992
C	2.25553	-3.97602	-3.07144
C	3.51972	-1.90913	-2.56424
C	0.10111	-4.47049	-2.12982
C	-1.02786	-3.00047	-0.47367
C	3.26367	1.06132	2.64033
C	2.72659	2.99361	1.22100
C	-2.36140	2.04414	1.92387
C	-2.07483	2.07732	-0.52026
H	3.09104	-4.23147	-3.72553
C	1.15726	-4.82380	-2.96500
C	4.73221	-2.23235	-1.91858
C	3.46275	-0.83014	-3.47300
H	-0.76024	-5.13122	-2.01882
C	-2.26831	-2.55274	-0.97929
C	-0.85774	-3.19773	0.91155
C	3.96922	1.91610	3.49950
C	3.23881	-0.39125	2.98642
C	3.43473	3.80626	2.11670
C	2.19048	3.63726	-0.01124
C	-3.72971	1.82689	1.75446
C	-1.78170	2.20109	3.30295
C	-3.44053	1.80767	-0.69663
C	-1.24063	2.51923	-1.69721
H	1.12572	-5.75730	-3.52869
C	5.86297	-1.44965	-2.17549
C	4.81042	-3.36711	-0.93185

C	4.62435	-0.09347	-3.71763
C	2.17246	-0.46157	-4.15409
C	-3.28832	-2.22993	-0.08020
C	-2.47802	-2.33476	-2.45316
C	-1.90810	-2.87182	1.77469
C	0.43511	-3.73810	1.46029
H	4.45070	1.48391	4.37838
C	4.04755	3.28237	3.25125
C	2.12174	-0.93920	3.65372
C	4.38007	-1.18761	2.74072
H	3.51736	4.86997	1.88951
C	1.19296	4.63475	0.08701
C	2.76918	3.34396	-1.26473
C	-4.25081	1.57360	0.45062
C	-4.62016	1.75345	2.96529
H	-1.88006	1.27658	3.89542
H	-0.71698	2.44947	3.24570
H	-2.30623	2.99033	3.86250
C	-4.01556	1.85415	-2.08668
H	-1.23947	1.78598	-2.51871
H	-1.64688	3.45923	-2.10623
H	-0.20323	2.71273	-1.41116
H	6.79725	-1.68963	-1.66053
C	5.83192	-0.37616	-3.07044
H	5.83095	-3.47360	-0.54180
H	4.13869	-3.19724	-0.07389
H	4.50937	-4.32449	-1.38088
H	4.57930	0.73738	-4.42715
H	1.69838	-1.33522	-4.62510
H	1.44414	-0.05738	-3.43196
H	2.34041	0.30220	-4.92427
H	-4.22388	-1.81787	-0.46839
C	-3.12423	-2.36764	1.30248
H	-3.47719	-1.92168	-2.64270
H	-1.73449	-1.62750	-2.85529
H	-2.37371	-3.26681	-3.02822
H	-1.76652	-3.00930	2.85071
H	0.78176	-4.61099	0.88767
H	1.24021	-2.98815	1.42562
H	0.31688	-4.03567	2.50983
H	4.59542	3.93544	3.93214
C	2.16452	-2.27815	4.05116
C	0.90869	-0.09975	3.93774
C	4.36747	-2.52869	3.13813
C	5.58287	-0.62809	2.02894
C	0.77041	5.28575	-1.07542
C	0.57194	5.00889	1.40681
C	2.30437	4.01502	-2.40299
C	3.90866	2.37264	-1.40201
N	-5.56888	1.13102	0.42169
H	-4.33940	0.91140	3.61866
H	-4.52151	2.66580	3.57336
H	-5.66689	1.62463	2.67460
H	-3.56020	2.67120	-2.66399
H	-3.81819	0.91781	-2.63258
H	-5.10188	1.98331	-2.07788
C	7.04919	0.47649	-3.30491
C	-4.23990	-2.01329	2.24732
H	1.29998	-2.69702	4.57325
C	3.26943	-3.09552	3.79213

```
H          1.16457         0.78196         4.54512
H          0.45507         0.28774         3.01383
H          0.14742        -0.68037         4.47471
H          5.24501        -3.14740         2.93112
H          5.31844        -0.26736         1.02099
H          6.01540         0.22932         2.56436
H          6.36038        -1.39509         1.91816
H         -0.01498         6.04243        -0.99482
C          1.30041         4.98283        -2.33353
H         -0.42901         5.43357         1.25189
H          0.47845         4.13166         2.05774
H          1.17298         5.76452         1.93721
H          2.75924         3.78313        -3.37030
H          4.46960         2.56216        -2.32626
H          4.60426         2.43705        -0.55359
H          3.56103         1.32736        -1.45230
N         -5.90667         0.40394        -0.57234
H          7.11667         0.80184        -4.35261
H          7.01396         1.38649        -2.68385
H          7.97265        -0.06040        -3.04937
H         -4.85905        -1.19995         1.84643
H         -4.90221        -2.87781         2.41640
H         -3.85002        -1.70285         3.22694
C          3.25789        -4.55054         4.17418
C          0.77951         5.65455        -3.57499
C         -7.26218         0.01200        -0.56824
H          2.73319        -5.14956         3.41192
H          4.27639        -4.95176         4.26454
H          2.73616        -4.71195         5.12803
H         -0.10648         5.12522        -3.96247
H          0.47655         6.69189        -3.37509
H          1.53228         5.66193        -4.37531
C         -8.24883         0.53957         0.28755
C         -7.62048        -0.96814        -1.50874
H         -7.96249         1.31252         1.00043
C         -9.55942         0.08326         0.21256
C         -8.92945        -1.43689        -1.58922
H         -6.84391        -1.35670        -2.16955
H        -10.34231         0.48015         0.85993
C         -9.87758        -0.90131        -0.72321
H         -9.22322        -2.20136        -2.30844
F        -11.15941        -1.34283        -0.79734
```

6.5.6 *E*-5B-I[4]

```
139
E-5B-I4 @ PBE-D3/def2-TZVP
P          0.24618        -0.38731        -1.17268
N          1.98128        -0.38138        -1.26390
C          0.25239         0.14395         0.54070
P          2.91429         0.03132         0.06526
C          2.60459        -0.69988        -2.52765
N          1.56896         0.33221         1.05831
N         -0.76313         0.27469         1.33865
C          3.35821         0.27949        -3.20830
C          2.40999        -1.98527        -3.08202
C          1.76367         0.67285         2.44587
C         -2.07690         0.22757         0.90437
```

C	3.91106	-0.05597	-4.45417
C	3.58920	1.66902	-2.71098
C	2.96928	-2.26638	-4.33413
C	1.67764	-3.05990	-2.35388
C	2.50522	-0.19967	3.27071
C	1.21154	1.86601	2.96906
C	-2.96368	-0.62550	1.62576
C	-2.57606	1.03620	-0.15630
H	4.48893	0.70427	-4.98256
C	3.71653	-1.31199	-5.02028
C	4.83137	2.00798	-2.13599
C	2.62333	2.67036	-2.95382
H	2.82133	-3.26214	-4.75527
C	0.34626	-3.37786	-2.69507
C	2.34703	-3.78268	-1.34748
C	2.64162	0.11375	4.63231
C	3.18340	-1.44976	2.81152
C	1.36450	2.12558	4.33731
C	0.55140	2.91566	2.14460
C	-4.30538	-0.74531	1.25295
C	-2.41513	-1.41148	2.78836
C	-3.91861	0.91369	-0.53789
C	-1.72434	2.07627	-0.83174
H	4.14822	-1.54748	-5.99421
C	5.08378	3.34545	-1.80618
C	5.88849	0.96295	-1.89287
C	2.92113	3.99218	-2.61144
C	1.30865	2.33867	-3.60562
C	-0.31849	-4.36498	-1.96182
C	-0.35814	-2.67946	-3.82755
C	1.64769	-4.77583	-0.65327
C	3.79197	-3.51276	-1.02354
H	3.20863	-0.57104	5.26520
C	2.06284	1.25711	5.17096
C	2.46282	-2.65865	2.71854
C	4.58806	-1.44811	2.64713
H	0.94087	3.04842	4.73544
C	-0.79771	3.26857	2.38589
C	1.31540	3.65425	1.21571
C	-4.79481	0.04762	0.17112
C	-5.16025	-1.78083	1.93710
H	-3.14083	-1.47433	3.60999
H	-2.15283	-2.44446	2.50324
H	-1.49689	-0.93834	3.15511
C	-4.44288	1.75553	-1.67105
H	-2.27471	3.02434	-0.91697
H	-0.80314	2.27709	-0.27689
H	-1.43880	1.77768	-1.85488
H	6.04625	3.60381	-1.35641
C	4.14635	4.35387	-2.04075
H	5.49578	0.11384	-1.31273
H	6.26916	0.54535	-2.83734
H	6.73704	1.38820	-1.34151
H	2.17209	4.76424	-2.80772
H	0.71219	1.65684	-2.97888
H	0.71627	3.24679	-3.77537
H	1.45729	1.83409	-4.57245
H	-1.35961	-4.59062	-2.20755
C	0.30767	-5.06336	-0.92392
H	0.06002	-2.97519	-4.80240

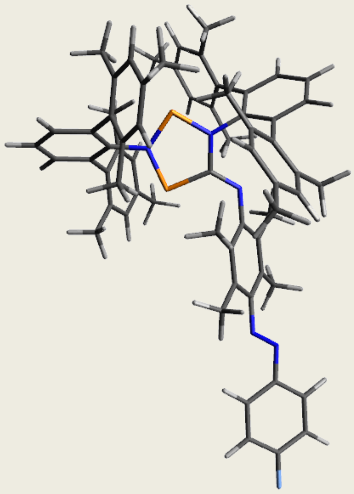

H	-1.42814	-2.92365	-3.82978
H	-0.25855	-1.58666	-3.75538
H	2.17035	-5.33891	0.12359
H	4.37566	-3.28984	-1.92740
H	3.89839	-2.65259	-0.34279
H	4.24428	-4.37555	-0.51947
H	2.17041	1.48064	6.23341
C	3.16541	-3.84703	2.49339
C	0.97018	-2.68195	2.88660
C	5.24413	-2.65762	2.40032
C	5.38023	-0.17071	2.74105
C	-1.36104	4.31982	1.65849
C	-1.64368	2.54791	3.40270
C	0.70277	4.69479	0.50464
C	2.78161	3.39623	1.00518
N	-6.10404	0.03036	-0.30831
H	-5.92965	-2.17393	1.26351
H	-4.54313	-2.61413	2.29763
H	-5.70516	-1.36190	2.79691
H	-5.42082	1.39133	-2.00156
H	-4.56880	2.80669	-1.36087
H	-3.74238	1.75727	-2.51847
C	4.42854	5.78476	-1.67097
C	-0.44644	-6.07970	-0.10947
H	2.60374	-4.78313	2.44207
C	4.55356	-3.87256	2.33971
H	0.67132	-2.28320	3.86800
H	0.46676	-2.05811	2.13510
H	0.58165	-3.70451	2.79534
H	6.32880	-2.64956	2.26146
H	6.43015	-0.34325	2.47100
H	4.97799	0.60614	2.07201
H	5.35207	0.25028	3.75725
H	-2.41046	4.57276	1.83376
C	-0.63463	5.03894	0.70316
H	-1.32607	1.50585	3.51921
H	-1.58467	3.03524	4.38926
H	-2.69817	2.55325	3.09553
H	1.30147	5.25977	-0.21526
H	3.30891	4.32868	0.76704
H	3.25313	2.95309	1.89199
H	2.95895	2.71247	0.15796
N	-7.02883	-0.28038	0.51006
H	3.82635	6.09685	-0.80197
H	5.48540	5.93191	-1.41205
H	4.17789	6.46840	-2.49525
H	0.23101	-6.82193	0.33500
H	-0.98904	-5.59178	0.71675
H	-1.19217	-6.61174	-0.71641
C	5.28806	-5.16818	2.12366
C	-1.28774	6.12557	-0.10713
C	-8.31336	-0.35124	-0.07488
H	5.74998	-5.52147	3.05944
H	4.61205	-5.95975	1.77217
H	6.09692	-5.05383	1.38758
H	-2.00892	6.69843	0.49279
H	-0.54505	6.82468	-0.51559
H	-1.84204	5.69846	-0.95920
C	-8.56234	-0.36460	-1.46077
C	-9.39271	-0.44070	0.81898

```
H          -7.71424         -0.31503         -2.14355
C          -9.86470         -0.44863         -1.93921
C         -10.70252         -0.51787          0.35141
H          -9.17699         -0.43831          1.88845
H         -10.08302         -0.46812         -3.00763
C         -10.91456         -0.51988         -1.02361
H         -11.55269         -0.58018          1.03066
F         -12.18626         -0.60654         -1.49265
```

6.5.7 *E*-5B-I[5]

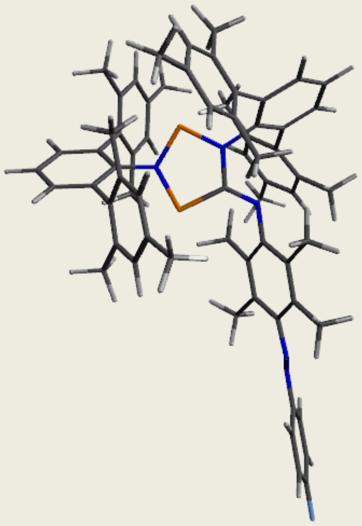

139

***E*-5B-I[5]** @ PBE-D3/def2-TZVP

```
P           0.20887         -0.44528          1.12255
N           1.93400         -0.32566          1.29547
C           0.26067          0.07856         -0.59225
P           2.89942          0.14203          0.00835
C           2.51615         -0.59573          2.58970
N           1.58612          0.34997         -1.04881
N          -0.72082          0.14258         -1.43813
C           3.17480          0.43348          3.29476
C           2.37565         -1.88664          3.14771
C           1.82192          0.69491         -2.42885
C          -2.05107          0.00737         -1.07905
C           3.68605          0.14292          4.56937
C           3.34614          1.83012          2.79340
C           2.88996         -2.12270          4.42807
C           1.74783         -3.01015          2.39627
C           1.21717          1.84602         -2.98733
C           2.65496         -0.13240         -3.21178
C          -2.67049          0.78979         -0.05894
C          -2.82994         -0.90545         -1.84367
H           4.18987          0.94173          5.11634
C           3.54207         -1.11819          5.13922
C           2.31306          2.77471          2.98106
C           4.59128          2.23670          2.27120
H           2.78405         -3.12261          4.85206
C           2.50774         -3.69423          1.42780
C           0.42469         -3.41008          2.67812
C           1.41282          2.10503         -4.35025
C           0.45601          2.85659         -2.20215
C           2.82966          0.17887         -4.56948
C           3.39705         -1.32828         -2.70915
C          -4.03013          0.60369          0.24266
C          -1.90848          1.87483          0.65626
C          -4.17446         -1.10668         -1.53529
C          -2.18365         -1.65718         -2.97568
H           3.93954         -1.31866          6.13530
C           2.54886          4.10905          2.63935
C           0.98991          2.37064          3.57209
C           4.77996          3.58409          1.93881
C           5.71678          1.25308          2.08552
C           1.90520         -4.73212          0.70878
C           3.94717         -3.33655          1.17101
C          -0.14163         -4.44054          1.92226
C          -0.37445         -2.75121          3.77061
H           0.94858          2.99564         -4.77553
C           2.20126          1.27757         -5.14432
```

C	-0.89995	3.12412	-2.50709
C	1.12903	3.64484	-1.24415
H	3.46800	-0.47142	-5.17000
C	2.76034	-2.58427	-2.63310
C	4.78923	-1.22484	-2.48179
C	-4.79211	-0.33569	-0.50882
C	-4.61541	1.35464	1.41046
H	-2.51682	2.78725	0.72867
H	-1.63180	1.58660	1.68476
H	-0.98515	2.13436	0.12985
C	-4.97713	-2.11460	-2.31552
H	-1.88457	-2.67562	-2.67459
H	-2.86695	-1.76144	-3.82949
H	-1.27493	-1.13752	-3.30142
H	1.74760	4.83715	2.79273
C	3.77588	4.53756	2.12096
H	1.12055	1.87868	4.54796
H	0.33927	3.24393	3.70774
H	0.46403	1.65369	2.92177
H	5.74478	3.89480	1.52900
H	6.07521	0.86169	3.04969
H	5.40121	0.38044	1.49306
H	6.56487	1.72405	1.57192
H	2.49799	-5.26401	-0.03918
C	0.57461	-5.10214	0.91905
H	4.47330	-3.07990	2.10092
H	4.47378	-4.16994	0.69018
H	4.03356	-2.47066	0.49469
H	-1.17692	-4.73016	2.12107
H	-0.34460	-1.65480	3.69045
H	-1.42491	-3.06629	3.72841
H	0.01854	-3.00992	4.76620
H	2.34012	1.49971	-6.20343
C	-1.56049	4.14047	-1.81253
C	-1.65261	2.34806	-3.55601
C	0.42058	4.64699	-0.56778
C	2.59748	3.48139	-0.96576
C	3.53490	-3.71761	-2.36320
C	1.28170	-2.71520	-2.86414
C	5.51758	-2.38193	-2.19143
C	5.49146	0.10547	-2.55390
N	-6.14128	-0.62437	-0.30389
H	-5.04977	2.31881	1.10306
H	-5.43614	0.79732	1.87461
H	-3.84532	1.55586	2.16648
H	-5.22732	-1.73418	-3.31998
H	-4.40881	-3.04469	-2.45919
H	-5.91986	-2.33638	-1.80494
C	3.99123	5.98028	1.75201
C	-0.07586	-6.16650	0.07709
H	-2.61466	4.32625	-2.03642
C	-0.92508	4.90625	-0.82904
H	-1.57545	2.83132	-4.54332
H	-1.26910	1.32582	-3.64764
H	-2.71894	2.29342	-3.29857
H	0.94932	5.25037	0.17538
H	3.13656	3.06701	-1.82763
H	3.05277	4.44623	-0.70891
H	2.77886	2.81270	-0.10736
H	3.03964	-4.69108	-2.32594

```
C          4.91283       -3.64265       -2.14755
H          0.70403       -2.13045       -2.13476
H          0.99616       -2.33749       -3.85764
H          0.96437       -3.76320       -2.78722
H          6.59159       -2.29515       -2.00469
H          5.00461        0.85573       -1.91121
H          6.53780        0.01096       -2.23570
H          5.47873        0.51640       -3.57450
N         -6.88034        0.29816        0.16870
H          3.70886        6.65083        2.57703
H          5.04038        6.17674        1.49461
H          3.37522        6.26503        0.88352
H         -0.81197       -6.74439        0.65310
H         -0.61156       -5.71586       -0.77442
H          0.66685       -6.86437       -0.33368
C         -1.68127        5.95229       -0.05581
C          5.72720       -4.87983       -1.88105
C         -8.21181       -0.10430        0.42046
H         -2.40567        6.47974       -0.69241
H         -2.24974        5.49349        0.77000
H         -1.00283        6.69556        0.38516
H          5.08824       -5.72919       -1.60323
H          6.30094       -5.17590       -2.77368
H          6.45327       -4.71613       -1.07130
C         -9.12331        0.92632        0.70096
C         -8.65574       -1.44047        0.43217
H         -8.75626        1.95373        0.69586
C        -10.46123        0.64368        0.96597
C         -9.98696       -1.73532        0.70231
H         -7.93345       -2.23187        0.23234
H        -11.18422        1.43126        1.17815
C        -10.86927       -0.68642        0.96115
H        -10.35388       -2.76218        0.72428
F        -12.16862       -0.97641        1.22959
```

6.5.8 *E*-5B-I^{6}

```
139
E-5B-I^6 @ PBE-D3/def2-TZVP
P          0.20927       -0.44544       -1.12282
N          1.93439       -0.32539       -1.29544
C          0.26063        0.07826        0.59203
P          2.89948        0.14240       -0.00809
C          2.51686       -0.59522       -2.58957
N          1.58593        0.34993        1.04885
N         -0.72101        0.14192        1.43775
C          3.17546        0.43418       -3.29440
C          2.37675       -1.88611       -3.14772
C          1.82143        0.69481        2.42895
C         -2.05121        0.00664        1.07860
C          3.68707        0.14383       -4.56891
C          3.34635        1.83083       -2.79290
C          2.89140       -2.12196       -4.42799
C          1.74899       -3.00982       -2.39652
C          2.65459       -0.13233        3.21193
C          1.21632        1.84573        2.98744
C         -2.83004       -0.90630        1.84313
C         -2.67067        0.78907        0.05852
```

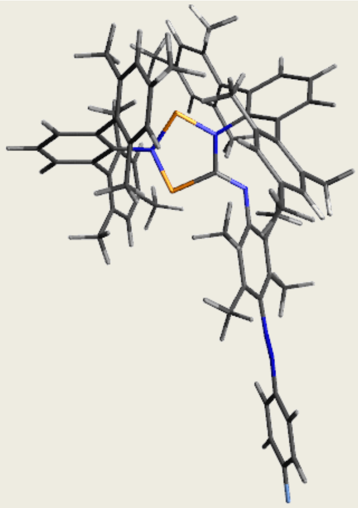

H	4.19085	0.94278	-5.11570
C	3.54347	-1.11726	-5.13890
C	4.59128	2.23772	-2.27046
C	2.31303	2.77515	-2.98065
H	2.78580	-3.12186	-4.85208
C	0.42599	-3.41000	-2.67870
C	2.50883	-3.69383	-1.42795
C	2.82905	0.17890	4.56966
C	3.39701	-1.32801	2.70931
C	1.41173	2.10470	4.35039
C	0.45501	2.85617	2.20223
C	-4.17456	-1.10755	1.53475
C	-2.18371	-1.65811	2.97506
C	-4.03030	0.60294	-0.24308
C	-1.90872	1.87414	-0.65669
H	3.94123	-1.31757	-6.13489
C	4.77952	3.58514	-1.93790
C	5.71700	1.25439	-2.08460
C	2.54838	4.10952	-2.63876
C	0.99009	2.37079	-3.57196
C	-0.14028	-4.44062	-1.92304
C	-0.37305	-2.75119	-3.77130
C	1.90634	-4.73189	-0.70914
C	3.94814	-3.33590	-1.17085
H	3.46749	-0.47127	5.17022
C	2.20030	1.27739	5.14451
C	2.76060	-2.58413	2.63305
C	4.78921	-1.22424	2.48217
H	0.94719	2.99516	4.77568
C	-0.90108	3.12330	2.50695
C	1.12799	3.64470	1.24444
C	-4.79225	-0.33647	0.50839
C	-4.97720	-2.11556	2.31490
H	-1.88448	-2.67646	2.67384
H	-1.27506	-1.13838	3.30091
H	-2.86703	-1.76258	3.82883
C	-4.61556	1.35388	-1.41090
H	-2.51717	2.78646	-0.72932
H	-0.98550	2.13388	-0.13018
H	-1.63183	1.58583	-1.68511
H	5.74420	3.89608	-1.52793
C	3.77520	4.53833	-2.12012
H	5.40177	0.38209	-1.49145
H	6.07513	0.86242	-3.04864
H	6.56521	1.72580	-1.57159
H	1.74695	4.83741	-2.79222
H	1.12108	1.87860	-4.54767
H	0.46410	1.65393	-2.92162
H	0.33939	3.24397	-3.70801
H	-1.17547	-4.73044	-2.12210
C	0.57587	-5.10216	-0.91972
H	-1.42336	-3.06683	-3.72963
H	-0.34381	-1.65479	-3.69075
H	0.02049	-3.00935	-4.76682
H	2.49907	-5.26372	0.03891
H	4.47445	-3.07923	-2.10067
H	4.03423	-2.46994	-0.49458
H	4.47477	-4.16916	-0.68983
H	2.33898	1.49951	6.20365
C	3.53546	-3.71728	2.36316

C	1.28196	-2.71543	2.86387
C	5.51787	-2.38114	2.19184
C	5.49113	0.10623	2.55453
C	-1.56177	4.13953	1.81236
C	-1.65371	2.34697	3.55570
C	0.41940	4.64672	0.56803
C	2.59655	3.48167	0.96635
N	-6.14145	-0.62508	0.30350
H	-5.22727	-1.73531	3.31945
H	-5.91998	-2.33722	1.80437
H	-4.40890	-3.04570	2.45835
H	-5.43661	0.79681	-1.87476
H	-5.04942	2.31832	-1.10361
H	-3.84554	1.55464	-2.16711
C	3.99007	5.98109	-1.75102
C	-0.07458	-6.16673	-0.07801
H	3.04042	-4.69086	2.32574
C	4.91341	-3.64199	2.14774
H	0.99619	-2.33785	3.85735
H	0.70424	-2.13079	2.13445
H	0.96490	-3.76350	2.78685
H	6.59189	-2.29410	2.00528
H	6.53747	0.01203	2.23623
H	5.00405	0.85651	1.91205
H	5.47839	0.51692	3.57523
H	-2.61604	4.32500	2.03606
C	-0.92639	4.90557	0.82905
H	-1.57693	2.83027	4.54302
H	-2.71995	2.29196	3.29802
H	-1.26986	1.32487	3.64742
H	0.94811	5.25031	-0.17497
H	3.13561	3.06771	1.82844
H	2.77833	2.81280	0.10818
H	3.05157	4.44659	0.70931
N	-6.88050	0.29761	-0.16878
H	3.70792	6.65160	-2.57614
H	3.37360	6.26567	-0.88280
H	5.03905	6.17777	-1.49313
H	-0.61153	-5.71620	0.77278
H	-0.80964	-6.74545	-0.65455
H	0.66828	-6.86383	0.33377
C	5.72811	-4.87896	1.88122
C	-1.68273	5.95145	0.05576
C	-8.21202	-0.10469	-0.42053
H	6.45374	-4.71522	1.07109
H	6.30239	-5.17454	2.77366
H	5.08933	-5.72862	1.60395
H	-1.00443	6.69505	-0.38488
H	-2.25075	5.49257	-0.77032
H	-2.40754	6.47851	0.69220
C	-8.65609	-1.44082	-0.43239
C	-9.12345	0.92606	-0.70083
H	-7.93386	-2.23231	-0.23272
C	-9.98735	-1.73550	-0.70248
C	-10.46140	0.64359	-0.96579
H	-8.75628	1.95343	-0.69560
H	-10.35438	-2.76232	-0.72457
C	-10.86958	-0.68647	-0.96113
H	-11.18433	1.43127	-1.17782
F	-12.16898	-0.97629	-1.22951

6.5.9 *E*-5H-I[1]

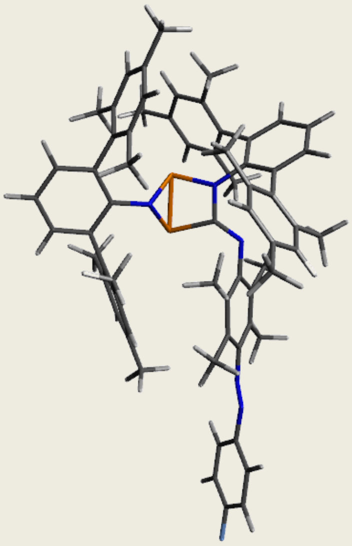

```
139
E-5H-I¹ @ PBE-D3/def2-TZVP
P          -0.34950          1.03804          0.79566
N          -1.53541          1.34731         -0.54004
C          -0.46316         -0.83332          0.53689
P          -2.49495          0.57084          0.76401
C          -1.83971          2.69694         -0.88870
N          -1.83004         -1.07570          0.44075
N           0.44305         -1.71574          0.69660
C          -0.84672          3.71405         -0.89782
C          -3.15557          3.02604         -1.30840
C          -2.41156         -2.36301          0.65484
C           1.80548         -1.40553          0.69944
C          -1.19579          5.01557         -1.28296
C           0.59359          3.47932         -0.58425
C          -3.45398          4.34182         -1.68503
C          -4.25704          2.03211         -1.44650
C          -2.01524         -3.47901         -0.12142
C          -3.34876         -2.52834          1.70221
C           2.53085         -1.61242          1.89575
C           2.45236         -0.99361         -0.49357
H          -0.40912          5.77296         -1.28640
C          -2.49086          5.34553         -1.66816
C           1.43824          2.96183         -1.58838
C           1.13775          3.88472          0.65487
H          -4.47167          4.55977         -2.01539
C          -5.36571          2.07386         -0.57126
C          -4.27153          1.17067         -2.56428
C          -2.52738         -4.74132          0.21421
C          -1.15120         -3.39957         -1.33228
C          -3.84119         -3.80627          1.99027
C          -3.85846         -1.37120          2.48965
C           3.89313         -1.30932          1.92995
C           1.81633         -2.12810          3.11479
C           3.84069         -0.77393         -0.48711
C           1.66463         -0.82602         -1.76595
H          -2.74082          6.36327         -1.96979
C           2.80263          2.81305         -1.31818
C           0.88404          2.60871         -2.94364
C           2.50390          3.70687          0.88600
C           0.25682          4.44451          1.73790
C          -6.46317          1.24828         -0.82965
C          -5.37400          2.98325          0.62769
C          -5.38918          0.35667         -2.77848
C          -3.12322          1.16014         -3.53703
H          -2.22033         -5.59463         -0.39243
C          -3.42342         -4.91725          1.26361
C          -1.65590         -2.77648         -2.49395
C           0.09667         -4.05959         -1.37391
H          -4.55480         -3.91499          2.80922
C          -5.14943         -0.86948          2.20698
C          -3.08520         -0.79741          3.52081
C           4.55693         -0.91561          0.73519
C           4.66167         -1.44398          3.21732
H           0.92000         -2.68922          2.82085
H           2.46284         -2.78355          3.71276
H           1.48448         -1.30881          3.77459
```

C	4.51356	-0.33418	-1.75956
H	0.66540	-1.26270	-1.68996
H	1.54720	0.23666	-2.02105
H	2.16962	-1.31172	-2.61105
H	3.45547	2.42050	-2.10102
C	3.35494	3.16070	-0.08146
H	0.43607	3.48932	-3.42910
H	1.67126	2.21649	-3.60077
H	0.08721	1.85305	-2.87097
H	2.91707	4.00240	1.85440
H	-0.44926	3.67858	2.10163
H	0.85499	4.78383	2.59346
H	-0.35295	5.28554	1.37900
H	-7.32238	1.28989	-0.15460
C	-6.49547	0.37833	-1.92440
H	-6.31286	2.88148	1.18720
H	-4.54469	2.73698	1.30986
H	-5.24976	4.03827	0.34595
H	-5.40198	-0.30191	-3.65107
H	-2.20422	0.78481	-3.06050
H	-3.34608	0.52268	-4.40209
H	-2.90213	2.17488	-3.89980
H	-3.80486	-5.91075	1.50321
C	-0.88718	-2.78650	-3.66261
C	-3.02613	-2.15822	-2.50699
C	0.82859	-4.04689	-2.56631
C	0.65763	-4.78013	-0.17635
C	-5.61452	0.23723	2.92025
C	-6.00135	-1.49676	1.13744
C	-3.58813	0.31697	4.20259
C	-1.75156	-1.38027	3.89763
N	5.90698	-0.59144	0.90419
H	4.08694	-1.04715	4.06589
H	4.88132	-2.50092	3.44253
H	5.62100	-0.91954	3.14898
H	5.34069	0.35808	-1.56342
H	4.96376	-1.18771	-2.28891
H	3.79800	0.14716	-2.43486
C	4.81480	2.94293	0.20988
C	-7.67407	-0.52712	-2.15911
H	-1.28757	-2.30434	-4.55894
C	0.36280	-3.40987	-3.71920
H	-3.77985	-2.84420	-2.09261
H	-3.06697	-1.24030	-1.90469
H	-3.32345	-1.89712	-3.53007
H	1.80309	-4.54297	-2.58697
H	1.75459	-4.80927	-0.22595
H	0.36616	-4.28355	0.75649
H	0.30123	-5.82159	-0.13030
H	-6.60715	0.63253	2.68913
C	-4.84080	0.86137	3.90581
H	-5.49937	-1.46175	0.15849
H	-6.20561	-2.55569	1.35118
H	-6.95841	-0.96983	1.04421
H	-2.98028	0.77079	4.98987
H	-1.07261	-1.44515	3.03748
H	-1.26389	-0.77668	4.67377
H	-1.86414	-2.40710	4.27893
N	6.68973	-0.76608	-0.08036
H	5.40758	2.89423	-0.71353

```
H             5.22233          3.74667          0.83944
H             4.96844          1.99432          0.74899
H            -7.55639         -1.47598         -1.61006
H            -8.61047         -0.06636         -1.81457
H            -7.78311         -0.77748         -3.22325
C             1.19702         -3.36389         -4.97075
C            -5.34315          2.09126          4.61205
C             8.00455         -0.29272          0.13767
H             0.56983         -3.30695         -5.87132
H             1.85211         -2.47679         -4.97336
H             1.84523         -4.24710         -5.05534
H            -5.24659          2.97943          3.96682
H            -6.40670          1.99771          4.87470
H            -4.77620          2.28661          5.53195
C             8.97143         -0.71122         -0.78984
C             8.37377          0.57882          1.17937
H             8.65942         -1.37664         -1.59607
C            10.29564         -0.29602         -0.67364
C             9.69018          1.00785          1.29955
H             7.60740          0.91261          1.87891
H            11.06300         -0.61682         -1.37796
C            10.63081          0.55838          0.37237
H            10.00140          1.69031          2.09117
F            11.91423          0.98162          0.48811
```

6.5.10 *E*-5H-I²

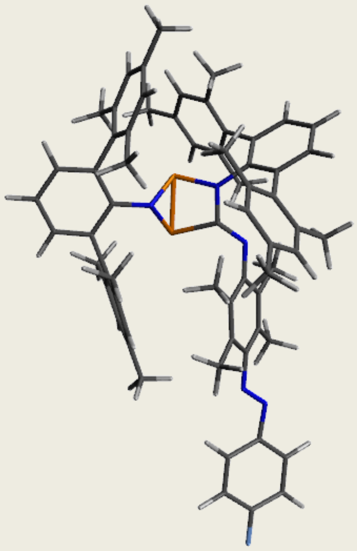

```
139
E-5H-I² @ PBE-D3/def2-TZVP
P             0.35937          1.00347         -0.81397
N             1.65271          1.39243          0.39683
C             0.40300         -0.83475         -0.36280
P             2.47896          0.42449         -0.87057
C             2.03978          2.75863          0.54596
N             1.76145         -1.13245         -0.31885
N            -0.54404         -1.68763         -0.38014
C             1.08776          3.81367          0.52261
C             3.40164          3.07443          0.79507
C             2.26917         -2.46441         -0.40282
C            -1.89312         -1.33128         -0.34390
C             1.52043          5.13608          0.69195
C            -0.38782          3.61280          0.41530
C             3.78270          4.41207          0.96210
C             4.47290          2.05388          0.97206
C             1.87658         -3.44852          0.53681
C             3.12503         -2.81051         -1.47418
C            -2.44796         -0.72929          0.81112
C            -2.70515         -1.68740         -1.44983
H             0.76481          5.92425          0.67589
C             2.85922          5.45053          0.89955
C            -1.11359          3.26753          1.57517
C            -1.07486          3.90575         -0.78367
H             4.83488          4.62157          1.16531
C             5.50284          1.92805          0.01286
C             4.55119          1.34363          2.18965
C             2.30568         -4.76905          0.33920
C             1.10161         -3.16249          1.77629
C             3.53888         -4.13993         -1.62063
```

C	3.63492	-1.79317	-2.43597
C	-3.82322	-0.46459	0.83637
C	-1.60568	-0.41441	2.01601
C	-4.07034	-1.37481	-1.45129
C	-2.06484	-2.38583	-2.62104
H	3.17424	6.48571	1.03520
C	-2.50786	3.18823	1.50485
C	-0.40085	3.01474	2.87734
C	-2.46709	3.78740	-0.81466
C	-0.32439	4.29313	-2.02809
C	6.58551	1.08841	0.28896
C	5.44316	2.67318	-1.29303
C	5.64911	0.50739	2.41784
C	3.49568	1.51976	3.24780
H	2.00167	-5.52027	1.06972
C	3.11982	-5.12500	-0.73150
C	1.71205	-2.41715	2.80836
C	-0.17048	-3.73956	1.98318
H	4.18964	-4.39099	-2.46034
C	4.96924	-1.34295	-2.30203
C	2.82540	-1.30403	-3.48229
C	-4.63468	-0.77600	-0.28750
C	-4.44978	0.08497	2.08892
H	-2.08399	-0.78033	2.93488
H	-0.61186	-0.86715	1.95992
H	-1.47808	0.67156	2.12810
C	-4.86731	-1.60558	-2.70815
H	-2.73034	-3.14705	-3.04859
H	-1.80652	-1.68544	-3.43301
H	-1.13308	-2.87061	-2.30441
H	-3.06627	2.94545	2.41248
C	-3.20614	3.42149	0.31548
H	0.29251	2.16366	2.79541
H	0.20336	3.88443	3.17668
H	-1.11647	2.79622	3.68073
H	-2.99209	3.99103	-1.75193
H	0.32394	3.46774	-2.36755
H	-1.01731	4.53576	-2.84407
H	0.33385	5.15700	-1.85825
H	7.38431	1.00044	-0.45252
C	6.67790	0.36467	1.48202
H	6.32758	2.45610	-1.90577
H	4.55143	2.37895	-1.86938
H	5.38048	3.76039	-1.14542
H	5.71107	-0.03384	3.36584
H	2.52217	1.13699	2.90461
H	3.76865	0.98743	4.16784
H	3.35180	2.58309	3.49031
H	3.43801	-6.16013	-0.86271
C	1.02293	-2.22481	4.01022
C	3.10859	-1.88168	2.65478
C	-0.81980	-3.52425	3.20394
C	-0.84373	-4.58387	0.93334
C	5.44529	-0.36879	-3.18099
C	5.85912	-1.88305	-1.21573
C	3.34457	-0.32393	-4.33744
C	1.42263	-1.80750	-3.68214
N	-5.96779	-0.37117	-0.16694
H	-3.82795	0.87166	2.53348
H	-5.45213	0.47655	1.88557

```
H          -4.55332        -0.70907         2.84781
H          -5.36305        -2.58827        -2.70591
H          -5.67212        -0.86928        -2.81365
H          -4.21978        -1.55188        -3.59282
C          -4.70083         3.26588         0.24781
C           7.83634        -0.56017         1.74008
H           1.50469        -1.64908         4.80564
C          -0.24897        -2.76333         4.22698
H           3.48818        -1.50876         3.61405
H           3.79747        -2.65713         2.28827
H           3.15210        -1.05224         1.93561
H          -1.81288        -3.95818         3.35147
H          -0.54278        -5.64063         1.01394
H          -1.93518        -4.54256         1.05018
H          -0.59064        -4.23826        -0.07569
H           6.47291        -0.01405        -3.06623
C           4.64292         0.17096        -4.19358
H           5.42967        -1.69398        -0.22005
H           5.99144        -2.97095        -1.30259
H           6.84722        -1.40908        -1.25198
H           2.71216         0.05966        -5.14268
H           0.71677        -1.30509        -3.00431
H           1.08184        -1.61823        -4.70858
H           1.34443        -2.88367        -3.47676
N          -6.84481        -1.01685        -0.82067
H          -5.17104         3.48805         1.21601
H          -5.13854         3.92912        -0.51123
H          -4.97519         2.23158        -0.01745
H           7.61528        -1.57803         1.37873
H           8.74476        -0.22099         1.22309
H           8.05794        -0.63714         2.81350
C          -0.99453        -2.50036         5.50737
C           5.16676         1.25941        -5.09047
C          -8.14864        -0.47735        -0.72257
H          -0.30754        -2.38249         6.35704
H          -1.58394        -1.57119         5.43449
H          -1.69571        -3.31386         5.73939
H           4.54721         1.37211        -5.99005
H           5.17188         2.22891        -4.56653
H           6.20062         1.05781        -5.40580
C          -9.18593        -1.28608        -1.21284
C          -8.44883         0.79635        -0.20323
H          -8.92827        -2.26490        -1.61983
C         -10.50874        -0.85266        -1.16836
C          -9.76406         1.24277        -0.16115
H          -7.63152         1.42189         0.15575
H         -11.32870        -1.46894        -1.53670
C         -10.77343         0.40759        -0.64135
H         -10.02346         2.22785         0.22826
F         -12.05611         0.84690        -0.60342
```

6.5.11 *E*-5H-I^3

```
139
E-5H-I³ @ PBE-D3/def2-TZVP
P          -0.25060         1.03742         0.63366
N          -1.54415         1.33963        -0.60212
C          -0.42547        -0.83862         0.44013
```

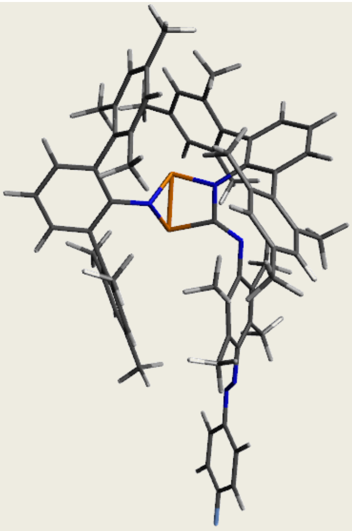

P	-2.40211	0.61739	0.79805
C	-1.85491	2.68849	-0.95109
N	-1.80145	-1.04897	0.46397
N	0.46536	-1.74208	0.55130
C	-3.19626	3.03399	-1.26290
C	-0.84940	3.68654	-1.06416
C	-2.38997	-2.31575	0.75992
C	1.83558	-1.49041	0.44956
C	-3.50299	4.34818	-1.63859
C	-4.32254	2.05857	-1.28454
C	-1.20742	4.98738	-1.44281
C	0.60656	3.42483	-0.87091
C	-3.23908	-2.43487	1.88527
C	-2.08328	-3.45835	-0.01899
C	2.64755	-1.72498	1.58909
C	2.40837	-1.14473	-0.79793
H	-4.54126	4.57930	-1.88534
C	-2.52492	5.33394	-1.72321
C	-5.35008	2.13888	-0.31765
C	-4.44885	1.17499	-2.37770
H	-0.41109	5.72965	-1.52851
C	1.34482	2.85001	-1.92491
C	1.26686	3.84536	0.30620
C	-3.73515	-3.69437	2.24221
C	-3.65642	-1.25081	2.68824
C	-2.59329	-4.70012	0.38761
C	-1.31916	-3.42599	-1.29739
C	4.02401	-1.46732	1.53353
C	1.99793	-2.22224	2.85399
C	3.79910	-0.98943	-0.88232
C	1.56804	-0.95621	-2.02914
H	-2.78252	6.35042	-2.02266
C	-6.47906	1.32794	-0.46116
C	-5.24059	3.07417	0.85611
C	-5.59408	0.37726	-2.47592
C	-3.39073	1.12416	-3.44667
C	2.72249	2.66387	-1.76461
C	0.66480	2.45949	-3.21043
C	2.63900	3.62077	0.43052
C	0.49786	4.46329	1.44183
H	-4.37864	-3.76810	3.12088
C	-3.40359	-4.83084	1.51099
C	-2.78960	-0.65641	3.62796
C	-4.96501	-0.74084	2.50972
H	-2.35576	-5.57404	-0.22092
C	-0.09363	-4.11587	-1.42416
C	-1.90352	-2.81732	-2.42907
C	4.59309	-1.06374	0.29148
C	4.86380	-1.69519	2.76214
H	0.95194	-2.48650	2.66577
H	2.50945	-3.11326	3.24661
H	2.01965	-1.46481	3.65395
C	4.42817	-0.70014	-2.21811
H	0.52798	-1.25755	-1.87864
H	1.57720	0.09696	-2.34438
H	1.96233	-1.54438	-2.86933
H	-7.27505	1.39884	0.28519
C	-6.62089	0.43623	-1.52931
H	-6.12466	2.99299	1.50160
H	-4.35355	2.83585	1.46429

H	-5.13592	4.12165	0.54015
H	-5.69378	-0.29862	-3.32947
H	-2.43944	0.74261	-3.04438
H	-3.69898	0.47329	-4.27472
H	-3.18616	2.12716	-3.84956
H	3.29382	2.23397	-2.59087
C	3.38822	3.01923	-0.58848
H	-0.10057	1.68652	-3.04088
H	0.14785	3.31885	-3.66352
H	1.39002	2.06968	-3.93671
H	3.13982	3.91789	1.35618
H	-0.22120	3.74235	1.86630
H	1.17426	4.77860	2.24676
H	-0.08936	5.33343	1.11578
H	-3.78532	-5.80932	1.80562
C	-3.22519	0.47593	4.32951
C	-1.40757	-1.19636	3.87451
C	-5.35558	0.38666	3.23232
C	-5.91634	-1.38351	1.53723
C	0.53794	-4.14776	-2.67244
C	0.54753	-4.82337	-0.25944
C	-1.23334	-2.87108	-3.65558
C	-3.25773	-2.16996	-2.34631
N	5.94653	-0.76300	0.09964
H	4.44452	-2.50156	3.37763
H	5.89928	-1.94587	2.50872
H	4.91520	-0.79141	3.38834
H	5.51729	-0.64136	-2.13558
H	4.16333	-1.48531	-2.94379
H	4.05808	0.24661	-2.63843
C	-7.83035	-0.45186	-1.64111
C	4.86140	2.77024	-0.41353
H	-2.54732	0.93998	5.05118
C	-4.49385	1.02439	4.13416
H	-0.66501	-0.68306	3.24404
H	-1.10699	-1.03886	4.91930
H	-1.34139	-2.26768	3.64684
H	-6.36193	0.78588	3.08070
H	-5.49965	-1.38880	0.51855
H	-6.12119	-2.43089	1.80125
H	-6.86843	-0.84020	1.50975
H	1.49582	-4.66839	-2.76047
C	-0.00632	-3.52570	-3.79865
H	0.34505	-4.29985	0.68216
H	0.17315	-5.85476	-0.15999
H	1.63583	-4.87998	-0.39763
H	-1.69548	-2.40001	-4.52781
H	-3.62934	-1.92002	-3.34764
H	-3.99001	-2.83284	-1.86173
H	-3.23326	-1.24144	-1.75966
N	6.55410	-0.21868	1.07353
H	-8.03677	-0.72025	-2.68626
H	-7.68068	-1.39217	-1.08527
H	-8.72502	0.03244	-1.22537
H	5.30053	2.30407	-1.30499
H	5.40374	3.70756	-0.21581
H	5.05150	2.09907	0.43765
C	-4.92870	2.26786	4.86097
C	0.72222	-3.52836	-5.11539
C	7.92834	0.02030	0.83976

```
H          -4.23687          2.51993          5.67544
H          -4.96574          3.12932          4.17501
H          -5.93588          2.15239          5.28739
H           1.36843         -4.41126         -5.21614
H           0.02294         -3.51267         -5.96309
H           1.36659         -2.63835         -5.20966
C           8.57686          0.83394          1.78186
C           8.65968         -0.50973         -0.23977
H           7.99161          1.22527          2.61524
C           9.92882          1.14053          1.64739
C          10.01076         -0.21709         -0.38005
H           8.14640         -1.15499         -0.95260
H          10.44879          1.77604          2.36402
C          10.62204          0.60772          0.56510
H          10.60282         -0.62020         -1.20240
F          11.94166          0.88946          0.42807
```

6.5.12 *E*-5H-I^4

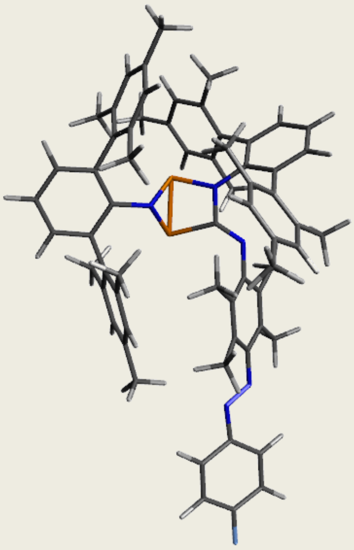

139

***E*-5H-I^4** @ PBE-D3/def2-TZVP

```
P           0.31435         -0.94438          0.86459
N           1.55814         -1.40162         -0.37742
C           0.43414          0.88957          0.39159
P           2.46047         -0.46627          0.85887
C           1.87687         -2.78527         -0.52711
N           1.80543          1.11366          0.30542
N          -0.45560          1.79995          0.41254
C           0.87528         -3.79107         -0.48160
C           3.21720         -3.16880         -0.79824
C           2.38283          2.41826          0.34514
C          -1.82943          1.57292          0.44453
C           1.23957         -5.13417         -0.65244
C          -0.58733         -3.52520         -0.34461
C           3.52945         -4.52416         -0.96423
C           4.33451         -2.20457         -1.00507
C           2.01649          3.39900         -0.60836
C           3.28052          2.74373          1.38826
C          -2.55217          2.10366          1.54112
C          -2.50074          0.96277         -0.64721
H           0.44616         -5.88368         -0.61972
C           2.55707         -5.51554         -0.88109
C          -1.32062         -3.13202         -1.48478
C          -1.26336         -3.82489          0.85887
H           4.56659         -4.78582         -1.18382
C           5.38245         -2.11154         -0.06190
C           4.43185         -1.52472         -2.23848
C           2.51728          4.69973         -0.45380
C           1.19364          3.12017         -1.81816
C           3.76524          4.05317          1.49189
C           3.75860          1.72259          2.36283
C          -3.93521          1.91899          1.60831
C          -1.81573          2.82195          2.63793
C          -3.89686          0.80480         -0.59866
C          -1.74962          0.52346         -1.87427
H           2.81811         -6.56561         -1.01701
C          -2.71290         -3.03652         -1.39640
C          -0.61883         -2.84968         -2.78646
```

C	-2.65378	-3.69403	0.90727
C	-0.50385	-4.24153	2.08857
C	6.50012	-1.33000	-0.36769
C	5.30621	-2.83220	1.25661
C	5.56504	-0.74631	-2.49657
C	3.35762	-1.67678	-3.28147
H	2.23317	5.44908	-1.19422
C	3.37521	5.03733	0.58857
C	1.73578	2.31435	-2.84288
C	-0.05327	3.75408	-2.00767
H	4.44695	4.29076	2.31065
C	5.07027	1.21082	2.21653
C	2.94469	1.28350	3.42727
C	-4.60336	1.22229	0.56376
C	-4.69417	2.40731	2.81336
H	-0.82470	3.13707	2.29120
H	-2.36873	3.70688	2.98145
H	-1.66806	2.17753	3.52078
C	-4.60419	0.29071	-1.82478
H	-0.67186	0.68645	-1.78938
H	-1.92294	-0.54182	-2.07364
H	-2.08403	1.08027	-2.76147
H	-3.27723	-2.75944	-2.29121
C	-3.40235	-3.30455	-0.20846
H	-0.01084	-3.70891	-3.10721
H	-1.34123	-2.61983	-3.58057
H	0.06800	-1.99461	-2.69116
H	-3.17139	-3.91393	1.84495
H	0.12381	-5.12524	1.90519
H	0.17607	-3.43856	2.41947
H	-1.19017	-4.46590	2.91530
H	7.31254	-1.26754	0.36145
C	6.61094	-0.63400	-1.57561
H	6.21584	-2.66175	1.84682
H	4.44769	-2.47450	1.84746
H	5.17260	-3.91526	1.12687
H	5.64091	-0.22804	-3.45634
H	2.40231	-1.25475	-2.93364
H	3.63887	-1.16850	-4.21255
H	3.17267	-2.73767	-3.50642
H	3.74880	6.05747	0.68723
C	1.00160	2.11728	-4.01666
C	3.11072	1.72026	-2.71362
C	-0.74856	3.53422	-3.20221
C	-0.65181	4.66604	-0.96910
C	5.51633	0.23025	3.10319
C	5.96767	1.69441	1.10987
C	3.43457	0.29506	4.29120
C	1.56395	1.83989	3.64179
N	-5.97853	1.05505	0.76006
H	-4.50246	3.47615	2.99295
H	-5.77004	2.25522	2.68653
H	-4.37612	1.87427	3.72389
H	-4.58502	-0.80756	-1.87887
H	-5.65873	0.58200	-1.83958
H	-4.11551	0.66929	-2.73311
C	-4.89733	-3.16842	-0.13000
C	7.80881	0.22908	-1.86577
H	1.43067	1.49404	-4.80646
C	-0.24827	2.71199	-4.21460

H	3.84316	2.47238	-2.38511
H	3.13796	0.90666	-1.97581
H	3.44639	1.30836	-3.67319
H	-1.72200	4.01465	-3.33653
H	-1.74814	4.65896	-1.03840
H	-0.36778	4.35655	0.04321
H	-0.31905	5.70692	-1.10984
H	6.52531	-0.17167	2.97855
C	4.70716	-0.25778	4.13712
H	5.51076	1.52246	0.12349
H	6.16069	2.77405	1.18585
H	6.92933	1.16813	1.13282
H	2.79795	-0.04860	5.11113
H	0.80541	1.22753	3.13044
H	1.30742	1.84659	4.70991
H	1.47037	2.86063	3.25062
N	-6.51249	0.03301	0.22623
H	-5.31169	-3.78531	0.67958
H	-5.19772	-2.12415	0.06102
H	-5.37420	-3.47252	-1.07367
H	8.70747	-0.14581	-1.35641
H	8.01750	0.27801	-2.94341
H	7.64317	1.26212	-1.51812
C	-1.04403	2.44610	-5.46399
C	5.19844	-1.35259	5.04462
C	-7.91258	-0.05345	0.40022
H	-1.65621	1.53543	-5.35353
H	-1.72925	3.27496	-5.68893
H	-0.38977	2.29208	-6.33356
H	6.23492	-1.17393	5.36555
H	4.57073	-1.44358	5.94093
H	5.18370	-2.32543	4.52722
C	-8.72247	0.98751	0.89237
C	-8.50292	-1.27070	0.02421
H	-8.25221	1.93298	1.16219
C	-10.09403	0.80593	1.02016
C	-9.87576	-1.46608	0.15378
H	-7.85858	-2.06029	-0.36479
H	-10.74533	1.59757	1.39217
C	-10.64749	-0.42099	0.65184
H	-10.35194	-2.40564	-0.12603
F	-11.98697	-0.59467	0.77378

6.5.13 *Z*-5H-I^{1}

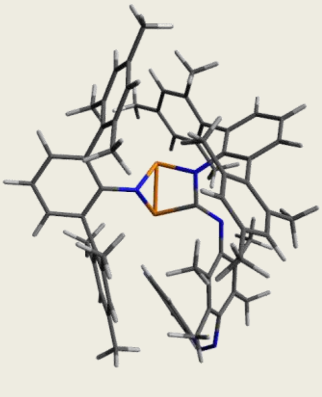

139

***Z*-5H-I^{1}** @ PBE-D3/def2-TZVP

P	-0.37632	-0.86079	0.09713
N	1.06118	-1.05109	-0.99856
C	0.00592	0.97589	0.38737
P	1.75731	-0.76075	0.62865
C	1.29050	-2.32935	-1.59119
N	1.38300	0.99408	0.59901
N	-0.78106	1.94463	0.64411
C	0.22830	-3.12446	-2.09623
C	2.62347	-2.79665	-1.74708
C	2.04471	2.09694	1.21965
C	-2.15567	1.89632	0.40005

C	0.51505	-4.36746	-2.68028
C	-1.20662	-2.71437	-2.15503
C	2.85693	-4.04364	-2.34024
C	3.83499	-2.00344	-1.39494
C	2.72590	1.89968	2.44245
C	1.96405	3.39135	0.65122
C	-2.66703	1.77602	-0.91478
C	-3.02364	2.10279	1.50208
H	-0.32249	-4.95142	-3.06738
C	1.81530	-4.84436	-2.79699
C	-1.62669	-1.84969	-3.18687
C	-2.16647	-3.34592	-1.33077
H	3.89326	-4.36713	-2.45663
C	4.66916	-2.41012	-0.32940
C	4.24702	-0.96522	-2.25835
C	3.27680	3.00353	3.10486
C	2.91390	0.54243	3.02774
C	2.52183	4.46645	1.35734
C	1.38423	3.66923	-0.69212
C	-4.06002	1.73319	-1.09666
C	-1.76950	1.71529	-2.11900
C	-4.40736	1.97465	1.32987
C	-2.44751	2.40080	2.85888
H	2.01558	-5.81107	-3.26017
C	-2.99720	-1.63339	-3.37656
C	-0.62195	-1.19909	-4.09982
C	-3.52232	-3.09238	-1.54913
C	-1.74338	-4.26115	-0.21403
C	5.89267	-1.76095	-0.14293
C	4.25840	-3.52196	0.59733
C	5.47500	-0.33699	-2.02590
C	3.40544	-0.57746	-3.44417
H	3.78949	2.83629	4.05403
C	3.16423	4.28713	2.57897
C	1.87017	-0.10580	3.72164
C	4.17701	-0.08023	2.90428
H	2.45868	5.46045	0.91190
C	0.25195	4.49995	-0.83629
C	2.05180	3.18565	-1.83833
C	-4.90018	1.70736	0.03301
C	-4.64420	1.63317	-2.47947
H	-0.71439	1.85134	-1.86540
H	-1.88006	0.75312	-2.63813
H	-2.03701	2.50073	-2.84125
C	-5.32821	2.13769	2.50887
H	-2.98432	3.22603	3.34845
H	-2.52103	1.53148	3.53320
H	-1.38868	2.67130	2.77582
H	-3.31629	-0.98669	-4.19858
C	-3.96360	-2.24292	-2.57118
H	0.05708	-0.53841	-3.53938
H	0.00868	-1.95082	-4.59803
H	-1.12245	-0.59941	-4.87119
H	-4.26054	-3.58179	-0.90876
H	-1.12342	-3.72093	0.52105
H	-2.61637	-4.67212	0.30972
H	-1.13138	-5.09892	-0.57732
H	6.53931	-2.08474	0.67716
C	6.31393	-0.71844	-0.97457
H	3.31740	-3.27309	1.11338

H	4.08496	-4.46413	0.05859
H	5.02660	-3.69543	1.36172
H	5.79470	0.46118	-2.70133
H	3.17915	-1.45295	-4.07083
H	2.43965	-0.15552	-3.12685
H	3.91946	0.16867	-4.06330
H	3.58731	5.14086	3.11016
C	2.08800	-1.39633	4.21841
C	0.54668	0.57411	3.93705
C	4.35099	-1.36474	3.42393
C	5.31481	0.61701	2.20928
C	-0.21521	4.78789	-2.12390
C	-0.46222	5.09153	0.35047
C	1.54470	3.49376	-3.10469
C	3.32733	2.39940	-1.71560
N	-6.28090	1.45503	-0.21078
H	-4.23726	2.42401	-3.12774
H	-4.39066	0.67524	-2.95652
H	-5.73592	1.72440	-2.44997
H	-5.22808	1.30107	3.21918
H	-5.08664	3.05663	3.06284
H	-6.37964	2.19497	2.20797
C	-5.43324	-2.00209	-2.78592
C	7.61961	-0.01179	-0.73058
H	1.27412	-1.90107	4.74580
C	3.31313	-2.05119	4.06448
H	-0.09434	-0.01832	4.60251
H	0.67856	1.57363	4.37695
H	0.00486	0.72268	2.99291
H	5.32587	-1.84755	3.31670
H	5.57091	1.56533	2.70300
H	6.20821	-0.01843	2.19410
H	5.05546	0.86153	1.16796
H	-1.10417	5.41643	-2.22894
C	0.40416	4.28556	-3.27066
H	-0.39795	4.43196	1.22321
H	-0.03231	6.06662	0.63058
H	-1.52325	5.25483	0.11725
H	2.06928	3.11633	-3.98713
H	3.81682	2.30055	-2.69223
H	4.03142	2.88373	-1.02311
H	3.14856	1.38623	-1.33017
N	-6.89250	0.51431	0.36212
H	-5.85468	-1.36059	-1.99567
H	-5.62422	-1.50945	-3.74847
H	-5.99758	-2.94556	-2.76403
H	8.05261	0.36745	-1.66649
H	7.47965	0.85331	-0.06164
H	8.35378	-0.67645	-0.25441
C	3.51201	-3.45779	4.55988
C	-0.15687	4.55944	-4.63990
C	-6.24225	-0.46788	1.17710
H	3.43855	-4.17854	3.72932
H	4.50501	-3.58784	5.01337
H	2.75318	-3.73321	5.30435
H	-0.85436	3.76154	-4.94479
H	-0.71315	5.50660	-4.66513
H	0.63635	4.60356	-5.39934
C	-7.02569	-0.97941	2.22396
C	-4.97783	-1.03515	0.92518

```
H          -8.02140        -0.56020         2.37541
C          -6.54259        -1.98611         3.05634
C          -4.50014        -2.06501         1.72968
H          -4.36253        -0.68892         0.09660
H          -7.13239        -2.37700         3.88535
C          -5.28081        -2.51029         2.79383
H          -3.52599        -2.51670         1.54129
F          -4.80140        -3.50082         3.58785
```

6.5.14 *Z*-5H-I^2

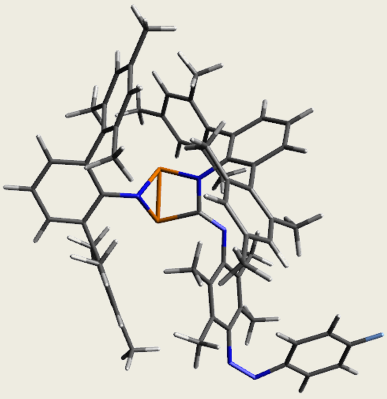

```
139
Z-5H-I² @ PBE-D3/def2-TZVP
P           0.65112         1.28663        -0.99228
N           1.93844         1.28570         0.28524
C          -0.02174        -0.38250        -0.40892
P           2.39585        -0.03976        -0.83743
C           2.80952         2.41268         0.37472
N           1.12039        -1.15796        -0.22831
N          -1.21304        -0.83297        -0.45537
C           2.34228         3.74219         0.18726
C           4.16935         2.21252         0.73156
C           1.08343        -2.58477        -0.17359
C          -2.34551        -0.01648        -0.51217
C           3.23976         4.81186         0.30652
C           0.91231         4.09832        -0.04976
C           5.02593         3.31545         0.84176
C           4.74913         0.88477         1.07963
C           1.79893        -3.33036        -1.13956
C           0.28727        -3.25322         0.78860
C          -3.23498        -0.19354        -1.59901
C          -2.65352         0.86921         0.55033
H           2.84949         5.82161         0.16297
C           4.58058         4.61455         0.61974
C           0.03919         4.17798         1.05577
C           0.45928         4.47635        -1.33295
H           6.06260         3.13010         1.13032
C           5.71434         0.28745         0.23796
C           4.46307         0.31714         2.34000
C           1.68325        -4.72521        -1.15654
C           2.69941        -2.67220        -2.12687
C           0.19318        -4.65104         0.72267
C          -0.40520        -2.57783         1.92201
C          -4.43921         0.52085        -1.64500
C          -2.86434        -1.15213        -2.69766
C          -3.86740         1.57251         0.51176
C          -1.74334         1.02142         1.73697
H           5.26164         5.46106         0.71366
C          -1.28324         4.58207         0.84820
C           0.52847         3.85342         2.44263
C          -0.87666         4.85265        -1.49725
C           1.37871         4.42333        -2.52199
C           6.36976        -0.86752         0.67282
C           6.03505         0.87169        -1.11124
C           5.13690        -0.84586         2.72861
C           3.48430         0.97745         3.27302
H           2.23428        -5.28256        -1.91644
C           0.87276        -5.38967        -0.24100
```

C	2.17629	-2.02283	-3.26516
C	4.09661	-2.72972	-1.92166
H	-0.41929	-5.15784	1.47010
C	-1.81322	-2.60281	2.02838
C	0.36856	-2.02551	2.96578
C	-4.76059	1.36344	-0.55703
C	-5.33033	0.41233	-2.85446
H	-2.19582	-1.93022	-2.30708
H	-3.74918	-1.63305	-3.13360
H	-2.32618	-0.64801	-3.51823
C	-4.28152	2.47121	1.64665
H	-1.02035	0.20371	1.81095
H	-1.18171	1.96419	1.68117
H	-2.31741	1.04004	2.67294
H	-1.95117	4.65730	1.71005
C	-1.77266	4.89712	-0.42357
H	1.37101	4.50096	2.72901
H	-0.27351	3.98081	3.18147
H	0.89348	2.81705	2.50786
H	-1.23019	5.11714	-2.49758
H	0.88514	4.82322	-3.41717
H	2.30571	4.98826	-2.34955
H	1.68073	3.38434	-2.73759
H	7.12208	-1.32133	0.02189
C	6.09430	-1.45470	1.91203
H	5.13256	0.91649	-1.74119
H	6.41949	1.89862	-1.03544
H	6.78208	0.25972	-1.63293
H	4.91981	-1.27770	3.70932
H	3.47272	0.47670	4.24945
H	3.73759	2.03673	3.42770
H	2.46414	0.95068	2.85968
H	0.78048	-6.47621	-0.27019
C	3.05855	-1.37835	-4.14033
C	0.70018	-2.04011	-3.55188
C	4.93931	-2.08138	-2.82696
C	4.67029	-3.45447	-0.73473
C	-2.41583	-2.03207	3.15522
C	-2.68438	-3.23508	0.97485
C	-0.27795	-1.46711	4.07335
C	1.87054	-2.08472	2.92778
N	-5.95768	2.13916	-0.54479
H	-4.73320	0.42866	-3.77677
H	-5.90647	-0.52748	-2.85568
H	-6.05194	1.23569	-2.90582
H	-3.42581	3.01978	2.05712
H	-5.04227	3.18841	1.31113
H	-4.72390	1.89340	2.47674
C	-3.21919	5.25151	-0.63335
C	6.78772	-2.71933	2.34080
H	2.65032	-0.86167	-5.01305
C	4.44042	-1.37904	-3.93015
H	0.46289	-1.42315	-4.42804
H	0.35087	-3.06583	-3.74852
H	0.10938	-1.67228	-2.70307
H	6.01876	-2.11511	-2.65762
H	5.76419	-3.37992	-0.72535
H	4.29415	-3.03078	0.20890
H	4.39384	-4.51856	-0.73714
H	-3.50774	-2.03705	3.22352

C	-1.67166	-1.45046	4.18436
H	-2.85613	-4.30276	1.18605
H	-3.66657	-2.74303	0.94238
H	-2.22902	-3.15653	-0.01866
H	0.32960	-1.04493	4.87883
H	2.22526	-3.10318	2.71014
H	2.28688	-1.43087	2.14933
H	2.29533	-1.76973	3.88887
N	-7.10345	1.62664	-0.61571
H	-3.81704	4.35051	-0.84634
H	-3.65191	5.72244	0.26004
H	-3.34557	5.93743	-1.48238
H	7.80750	-2.77641	1.93510
H	6.84649	-2.79433	3.43528
H	6.24341	-3.60791	1.98062
C	5.36893	-0.63533	-4.85130
C	-2.34882	-0.79542	5.35774
C	-7.33522	0.21962	-0.51241
H	4.88460	-0.40309	-5.80910
H	5.68626	0.31855	-4.39964
H	6.28079	-1.21434	-5.05630
H	-2.52073	0.27658	5.16412
H	-3.32752	-1.25058	5.56362
H	-1.73642	-0.86584	6.26752
C	-6.66750	-0.63553	0.38380
C	-8.41327	-0.27576	-1.26159
H	-5.84989	-0.25860	0.99594
C	-7.05580	-1.96637	0.50229
C	-8.78438	-1.61566	-1.17997
H	-8.94852	0.41254	-1.91716
H	-6.56079	-2.64254	1.19993
C	-8.09527	-2.43996	-0.29613
H	-9.60557	-2.02012	-1.77150
F	-8.45797	-3.74204	-0.19274

6.5.15 *Z*-5H-I^3

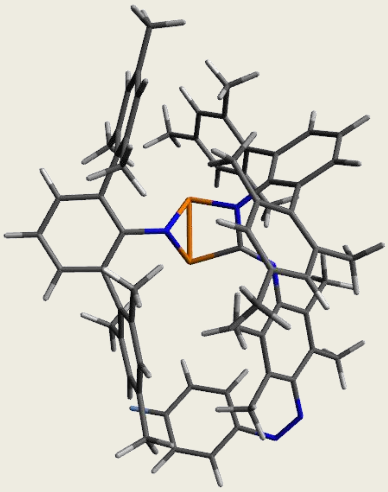

139

Z-5H-I^3 @ PBE-D3/def2-TZVP

P	-0.22632	0.57378	-0.80046
N	1.03183	1.34866	0.27300
C	0.09165	-1.15406	-0.07060
P	1.95265	0.29747	-0.84872
C	1.17827	2.76353	0.14136
N	1.48416	-1.18757	0.01426
N	-0.64327	-2.18710	0.05931
C	0.04961	3.62140	0.22608
C	2.46717	3.34620	-0.00483
C	2.20657	-2.38582	0.28623
C	-2.03131	-2.24079	-0.03408
C	0.22158	5.00173	0.04380
C	-1.33221	3.21687	0.62941
C	2.58402	4.73197	-0.17811
C	3.75489	2.60702	0.14987
C	3.09322	-2.90626	-0.68047
C	1.98132	-3.07219	1.50277
C	-2.88033	-1.37461	0.69900
C	-2.57422	-3.30243	-0.80599

H	-0.66280	5.63747	0.11912
C	1.47133	5.56593	-0.18486
C	-1.59973	2.98015	1.99842
C	-2.40445	3.31809	-0.28523
H	3.58746	5.14946	-0.28340
C	4.62000	2.40392	-0.94691
C	4.19726	2.31023	1.46025
C	3.69853	-4.14715	-0.44110
C	3.43759	-2.14655	-1.91653
C	2.59648	-4.31555	1.69212
C	1.17829	-2.46771	2.60031
C	-4.27434	-1.46787	0.53127
C	-2.32337	-0.37673	1.67251
C	-3.96125	-3.40677	-0.95766
C	-1.65978	-4.29091	-1.47570
H	1.58064	6.64210	-0.32306
C	-2.92769	2.90903	2.43043
C	-0.48268	2.81513	2.99097
C	-3.71715	3.20787	0.18866
C	-2.15947	3.55381	-1.75121
C	5.90278	1.89603	-0.71190
C	4.18266	2.70898	-2.35275
C	5.47837	1.78425	1.64421
C	3.33245	2.62939	2.64937
H	4.37421	-4.55074	-1.19765
C	3.43888	-4.86181	0.72561
C	2.55898	-2.10666	-3.02052
C	4.68243	-1.48202	-1.98026
H	2.41936	-4.84453	2.62991
C	-0.04113	-3.03687	3.02239
C	1.68639	-1.32309	3.25386
C	-4.79297	-2.44061	-0.35026
C	-5.17667	-0.55516	1.31460
H	-2.84969	-0.43092	2.63667
H	-1.26083	-0.54183	1.87528
H	-2.44951	0.65190	1.31003
C	-4.57202	-4.47697	-1.82254
H	-2.02541	-5.31981	-1.34973
H	-1.58663	-4.11161	-2.56111
H	-0.64887	-4.22115	-1.05917
H	-3.12447	2.74706	3.49410
C	-4.00339	3.03541	1.54586
H	0.06962	1.88117	2.80111
H	0.24420	3.63755	2.92408
H	-0.87230	2.77630	4.01668
H	-4.54100	3.28617	-0.52401
H	-3.10438	3.57307	-2.30807
H	-1.63632	4.50442	-1.92941
H	-1.52069	2.76251	-2.17498
H	6.57397	1.75339	-1.56292
C	6.35207	1.57549	0.57130
H	3.72732	3.70493	-2.43707
H	5.03012	2.64845	-3.04788
H	3.42472	1.98086	-2.68721
H	5.81494	1.56196	2.66079
H	3.84514	2.37407	3.58558
H	3.08124	3.70079	2.67348
H	2.37997	2.08253	2.61700
H	3.90671	-5.83352	0.89017
C	2.91575	-1.35271	-4.14359

C	1.27048	-2.88011	-3.00820
C	5.00195	-0.75034	-3.12784
C	5.64878	-1.53868	-0.82869
C	-0.76630	-2.40049	4.03733
C	-0.58054	-4.30695	2.41915
C	0.92679	-0.72015	4.26049
C	3.04619	-0.77655	2.91277
N	-6.19353	-2.59926	-0.55122
H	-4.87732	-0.52618	2.37220
H	-5.11334	0.47829	0.94174
H	-6.22539	-0.86696	1.27140
H	-4.11096	-5.45653	-1.63415
H	-5.65083	-4.55228	-1.63919
H	-4.42922	-4.26235	-2.89492
C	-5.42424	3.01832	2.04280
C	7.73091	1.01837	0.80049
H	2.22892	-1.31937	-4.99372
C	4.12549	-0.65484	-4.21302
H	0.59404	-2.54015	-2.21289
H	0.74585	-2.78300	-3.96758
H	1.45179	-3.94896	-2.81924
H	5.96371	-0.23279	-3.16985
H	5.91075	-2.57358	-0.56739
H	6.57061	-0.99689	-1.07028
H	5.21724	-1.08237	0.07576
H	-1.72612	-2.82792	4.34099
C	-0.31677	-1.22878	4.65035
H	-0.26852	-4.41891	1.37484
H	-0.22473	-5.18984	2.97458
H	-1.67839	-4.31620	2.45398
H	1.32390	0.16795	4.76043
H	3.40374	-0.10692	3.70473
H	3.78134	-1.58303	2.78130
H	3.03290	-0.19949	1.97662
N	-6.93995	-1.67763	-0.96756
H	-6.12570	2.74759	1.24160
H	-5.55475	2.30615	2.86980
H	-5.71827	4.01169	2.41846
H	8.24526	1.54560	1.61731
H	7.68657	-0.04597	1.08155
H	8.35125	1.09923	-0.10230
C	4.46711	0.18778	-5.41185
C	-1.15811	-0.51388	5.67259
C	-6.44979	-0.43929	-1.48672
H	4.00198	-0.20537	-6.32626
H	4.10664	1.22096	-5.27936
H	5.55319	0.23942	-5.57057
H	-1.77885	0.26019	5.19079
H	-1.83839	-1.20400	6.19031
H	-0.53720	-0.00879	6.42585
C	-7.28726	0.66995	-1.29084
C	-5.31016	-0.30737	-2.30158
H	-8.18945	0.53782	-0.69200
C	-6.97571	1.90826	-1.84627
C	-5.01009	0.91602	-2.89318
H	-4.66443	-1.16475	-2.48430
H	-7.60701	2.78300	-1.69003
C	-5.84048	2.00555	-2.64389
H	-4.13846	1.03689	-3.53656
F	-5.53491	3.20440	-3.20617

6.5.16 *Z*-5B-I^1

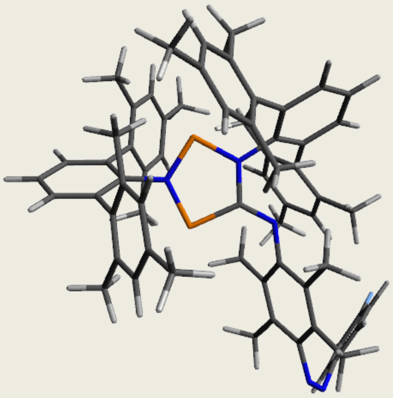

139

***Z*-5B-I^1** @ PBE-D3/def2-TZVP

P	0.59540	0.55449	-1.43901
N	2.05010	-0.33584	-1.10844
C	0.03730	0.44082	0.26403
P	2.39596	-0.87937	0.43860
C	2.93903	-0.63785	-2.20659
N	0.95883	-0.26110	1.10058
N	-1.01295	0.97492	0.80176
C	3.14649	-1.97781	-2.59680
C	3.55687	0.42674	-2.90087
C	0.72041	-0.37217	2.51848
C	-2.06326	1.52689	0.08303
C	3.98424	-2.22719	-3.69513
C	2.51182	-3.16070	-1.94158
C	4.37234	0.12716	-3.99866
C	3.41093	1.84640	-2.47109
C	1.67401	0.15530	3.41576
C	-0.45287	-1.00025	3.00083
C	-2.76495	0.82256	-0.93669
C	-2.50574	2.81950	0.48355
H	4.13920	-3.26378	-3.99940
C	4.58897	-1.18955	-4.39755
C	1.21505	-3.56411	-2.32957
C	3.26421	-3.95536	-1.05252
H	4.85212	0.95254	-4.52722
C	4.19518	2.32228	-1.40248
C	2.53495	2.71514	-3.15518
C	1.41828	0.08136	4.79415
C	2.96352	0.79933	3.02085
C	-0.66700	-1.03495	4.38525
C	-1.44769	-1.70605	2.14596
C	-3.89234	1.40607	-1.54135
C	-2.38167	-0.57293	-1.34482
C	-3.60752	3.41032	-0.13929
C	-1.77492	3.53137	1.58896
H	5.22958	-1.40674	-5.25358
C	0.69565	-4.75421	-1.81324
C	0.41426	-2.75656	-3.31444
C	2.70175	-5.13967	-0.55981
C	4.65948	-3.56101	-0.64446
C	4.04341	3.65219	-0.99576
C	5.19280	1.43256	-0.71060
C	2.40183	4.03055	-2.70119
C	1.75505	2.25071	-4.35617
H	2.15751	0.49966	5.47936
C	0.25061	-0.49422	5.28097
C	2.99431	2.16010	2.64988
C	4.17168	0.08634	3.19784
H	-1.56968	-1.52524	4.75203
C	-1.08342	-2.90224	1.49088
C	-2.79060	-1.26336	2.10620
C	-4.33521	2.67136	-1.09932
C	-4.55772	0.69485	-2.69052
H	-3.27682	-1.19012	-1.49919
H	-1.80954	-0.58652	-2.28873
H	-1.76890	-1.06553	-0.58375

C	-4.07639	4.79049	0.24303
H	-1.29705	2.79937	2.25200
H	-0.97155	4.17790	1.19672
H	-2.44829	4.16758	2.17805
H	-0.30766	-5.06305	-2.11939
C	1.42326	-5.56109	-0.93144
H	0.95687	-2.63373	-4.26435
H	-0.54830	-3.23976	-3.52509
H	0.20970	-1.74452	-2.93109
H	3.28602	-5.75098	0.13321
H	5.05127	-4.24777	0.11694
H	5.34974	-3.57033	-1.50153
H	4.68963	-2.54105	-0.23092
H	4.65738	4.02086	-0.17064
C	3.13344	4.51487	-1.61161
H	4.70830	0.80320	0.05338
H	5.69658	0.75989	-1.41846
H	5.95409	2.03044	-0.19476
H	1.70192	4.69534	-3.21446
H	2.41735	2.05775	-5.21436
H	1.21605	1.31396	-4.15195
H	1.01959	3.00705	-4.65884
H	0.06269	-0.53580	6.35489
C	4.23386	2.78877	2.49367
C	1.72281	2.93596	2.45305
C	5.38674	0.75151	3.00835
C	4.16620	-1.36719	3.59217
C	-2.05557	-3.60565	0.76703
C	0.30507	-3.47001	1.59133
C	-3.72412	-1.99862	1.37137
C	-3.24705	-0.02645	2.83445
N	-5.46892	3.31519	-1.66570
H	-5.21345	1.36202	-3.26122
H	-3.80153	0.29109	-3.37863
H	-5.17156	-0.15518	-2.35016
H	-3.22786	5.46986	0.40106
H	-4.72454	5.20284	-0.54079
H	-4.66125	4.78177	1.17864
C	0.83097	-6.83379	-0.38972
C	2.93145	5.91742	-1.10450
H	4.25090	3.84812	2.22555
C	5.44067	2.10893	2.67583
H	1.09470	2.91166	3.35646
H	1.93783	3.98415	2.20808
H	1.11195	2.51568	1.64198
H	6.31827	0.19267	3.13399
H	5.18033	-1.78539	3.55046
H	3.77875	-1.50933	4.61221
H	3.52134	-1.96481	2.92918
H	-1.76411	-4.53154	0.26299
C	-3.37856	-3.16766	0.68610
H	0.94638	-3.13070	0.76029
H	0.80019	-3.18157	2.52793
H	0.28159	-4.56561	1.53936
H	-4.75404	-1.63615	1.31911
H	-2.43884	0.70859	2.91818
H	-4.08249	0.44192	2.29728
H	-3.59755	-0.26668	3.85119
N	-6.60355	2.76915	-1.71215
H	-0.03287	-6.62538	0.26198

```
H          0.46922      -7.48330      -1.20065
H          1.56488      -7.39917       0.19955
H          2.67649       6.60934      -1.91918
H          2.10420       5.95338      -0.37678
H          3.83050       6.29552      -0.59827
C          6.76052       2.81707       2.52939
C         -4.39741      -3.90390      -0.14117
C         -6.91329       1.56590      -1.00536
H          6.66615       3.72016       1.91053
H          7.15095       3.13163       3.51057
H          7.51876       2.16438       2.07360
H         -4.18110      -4.98058      -0.18453
H         -5.41262      -3.76838       0.25705
H         -4.40278      -3.52952      -1.17853
C         -6.53074       1.32526       0.32680
C         -7.78843       0.67098      -1.63928
H         -5.87676       2.02817       0.84132
C         -6.99076       0.19521       0.99633
C         -8.21970      -0.48612      -0.99306
H         -8.11264       0.89291      -2.65699
H         -6.71994       0.00119       2.03461
C         -7.80746      -0.70581       0.31662
H         -8.87822      -1.20342      -1.48288
F         -8.22361      -1.82763       0.96094
```

6.5.17 *Z*-5B-I^2

```
139
Z-5B-I² @ PBE-D3/def2-TZVP
P         -0.59535       0.55498      -1.43892
N         -2.05018      -0.33523      -1.10864
C         -0.03727       0.44068       0.26406
P         -2.39609      -0.87926       0.43823
C         -2.93921      -0.63669      -2.20686
N         -0.95886      -0.26141       1.10040
N          1.01301       0.97458       0.80194
C         -3.14710      -1.97648      -2.59738
C         -3.55669       0.42827      -2.90091
C         -0.72043      -0.37295       2.51826
C          2.06336       1.52661       0.08334
C         -3.98490      -2.22534      -3.69580
C         -2.51289      -3.15972      -1.94236
C         -4.37226       0.12921      -3.99876
C         -3.41022       1.84777      -2.47077
C          0.45282      -1.00123       3.00041
C         -1.67398       0.15430       3.41572
C          2.50593       2.81911       0.48417
C          2.76502       0.82246      -0.93653
H         -4.14018      -3.26181      -4.00032
C         -4.58930      -1.18734      -4.39797
C         -3.26559      -3.95426      -1.05346
C         -1.21616      -3.56342      -2.33021
H         -4.85178       0.95486      -4.52713
C         -2.53391       2.71636      -3.15466
C         -4.19415       2.32364      -1.40195
C          0.66699      -1.03633       4.38482
C          1.44761      -1.70683       2.14535
C         -1.41823       0.07995       4.79408
```

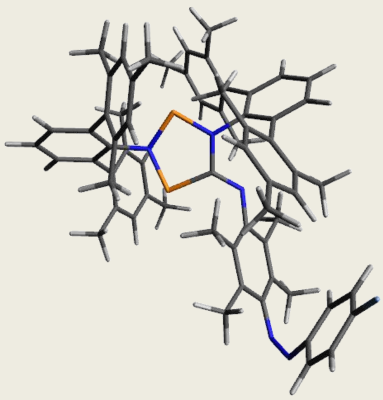

```
C          -2.96347       0.79851       3.02101
C           3.60780       3.40998      -0.13847
C           1.77501       3.53075       1.58966
C           3.89251       1.40600      -1.54098
C           2.38158      -0.57287      -1.34506
H          -5.22998      -1.40412      -5.25406
C          -2.70347      -5.13874      -0.56076
C          -4.66072      -3.55947      -0.64535
C          -0.69712      -4.75368      -1.81391
C          -0.41502      -2.75595      -3.31486
C          -2.40010       4.03152      -2.70020
C          -1.75433       2.25195      -4.35586
C          -4.04168       3.65333      -0.99473
C          -5.19216       1.43417      -0.71032
H           1.56966      -1.52674       4.75143
C          -0.25059      -0.49582       5.28071
C           2.79053      -1.26418       2.10568
C           1.08329      -2.90289       1.49005
H          -2.15744       0.49808       5.47943
C          -4.17167       0.08550       3.19779
C          -2.99421       2.15939       2.65052
C           4.33549       2.67114      -1.09861
C           4.07676       4.79004       0.24413
H           2.44815       4.16745       2.17847
H           0.97114       4.17670       1.19752
H           1.29772       2.79858       2.25295
C           4.55790       0.69499      -2.69027
H           3.27666      -1.19011      -1.49965
H           1.76879      -1.06563      -0.58410
H           1.80939      -0.58612      -2.28893
H          -3.28792      -5.74988       0.13225
C          -1.42506      -5.56045      -0.93227
H          -5.05320      -4.24691       0.11509
H          -4.69027      -2.54001      -0.23052
H          -5.35073      -3.56721      -1.50262
H           0.30622      -5.06265      -2.11982
H          -0.95719      -2.63343      -4.26507
H          -0.21076      -1.74381      -2.93166
H           0.54771      -3.23908      -3.52496
H          -1.69993       4.69616      -3.21331
C          -3.13134       4.51579      -1.61033
H          -1.21516       1.31527      -4.15174
H          -2.41686       2.05885      -5.21384
H          -1.01904       3.00835      -4.65880
H          -4.65539       4.02199      -0.16941
H          -4.70795       0.80433       0.05345
H          -5.95315       2.03226      -0.19428
H          -5.69628       0.76197      -1.41838
H          -0.06265      -0.53770       6.35463
C           3.72402      -1.99932       1.37070
C           3.24708      -0.02747       2.83420
C           2.05542      -3.60617       0.76604
C          -0.30520      -3.47065       1.59046
C          -5.38670       0.75076       3.00854
C          -4.16621      -1.36815       3.59167
C          -4.23376       2.78817       2.49455
C          -1.72268       2.93526       2.45389
N           5.46930       3.31498      -1.66477
H           4.66146       4.78111       1.17984
H           4.72508       5.20242      -0.53953
```

```
H        3.22829       5.46948       0.40210
H        3.80172       0.29145      -3.37851
H        5.21370       1.36225      -3.26078
H        5.17166      -0.15515      -2.35007
C       -0.83334      -6.83362      -0.39101
C       -2.92855       5.91804      -1.10273
H        4.75396      -1.63688       1.31851
C        3.37843      -3.16820       0.68518
H        4.08187       0.44154       2.29656
H        2.43868       0.70724       2.91903
H        3.59858      -0.26815       3.85050
H        1.76393      -4.53196       0.26183
H       -0.80001      -3.18287       2.52742
H       -0.94675      -3.13067       0.75988
H       -0.28180      -4.56621       1.53764
H       -6.31824       0.19191       3.13403
C       -5.44057       2.10832       2.67648
H       -5.18030      -1.78640       3.54953
H       -3.52109      -1.96553       2.92873
H       -3.77908      -1.51055       4.61180
H       -4.25078       3.84762       2.22683
H       -1.11194       2.51530       1.64257
H       -1.93767       3.98358       2.20940
H       -1.09446       2.91053       3.35721
N        6.60386       2.76880      -1.71131
H       -0.47979      -7.48679      -1.20267
H        0.03593      -6.62644       0.25377
H       -1.56505      -7.39477       0.20500
H       -3.82647       6.29549      -0.59403
H       -2.09950       5.95375      -0.37706
H       -2.67581       6.61063      -1.91754
C        4.39726      -3.90428      -0.14225
C       -6.76044       2.81643       2.53016
C        6.91344       1.56536      -1.00480
H        5.41234      -3.76980       0.25666
H        4.18028      -4.98077      -0.18682
H        4.40347      -3.52883      -1.17923
H       -6.66490       3.72284       1.91636
H       -7.51701       2.16571       2.06875
H       -7.15389       3.12540       3.51190
C        6.53077       1.32435       0.32726
C        7.78857       0.67054      -1.63891
H        5.87680       2.02716       0.84193
C        6.99067       0.19408       0.99650
C        8.21970      -0.48677      -0.99300
H        8.11287       0.89277      -2.65653
H        6.71975      -0.00021       2.03470
C        7.80735      -0.70681       0.31659
H        8.87820      -1.20398      -1.48297
F        8.22337      -1.82884       0.96062
```

6.5.18 *Z*-5B-I[3]

```
139
Z-5B-I3 @ PBE-D3/def2-TZVP
P        0.30280       0.03150      -1.09271
N       -1.15297       0.98909      -1.07779
C       -0.05228      -0.68026       0.52271
```

P	-2.18767	0.95905	0.23735
C	-1.49894	1.73627	-2.26621
N	-1.25735	-0.16850	1.09639
N	0.66619	-1.50177	1.21995
C	-0.66822	2.80084	-2.67989
C	-2.63457	1.36871	-3.02089
C	-1.62267	-0.52472	2.44554
C	1.79038	-2.13127	0.70005
C	-0.97756	3.46720	-3.87334
C	0.48116	3.31283	-1.87957
C	-2.90803	2.07599	-4.20190
C	-3.57812	0.26371	-2.67313
C	-1.86352	-1.87691	2.78612
C	-1.72209	0.49294	3.41799
C	2.98460	-2.07385	1.47207
C	1.75753	-2.91206	-0.49005
H	-0.33686	4.29512	-4.18133
C	-2.08581	3.11057	-4.63568
C	1.80840	3.06472	-2.29337
C	0.22687	4.15664	-0.78112
H	-3.78335	1.78425	-4.78483
C	-4.82531	0.57403	-2.09002
C	-3.29989	-1.05530	-3.09114
C	-2.14908	-2.18421	4.12288
C	-1.91852	-2.99272	1.80137
C	-2.02424	0.13313	4.74098
C	-1.53634	1.95254	3.15647
C	4.18018	-2.59358	0.96373
C	2.96472	-1.40443	2.81902
C	2.95337	-3.46017	-0.98474
C	0.47503	-3.18884	-1.22422
H	-2.31273	3.64267	-5.56071
C	2.85787	3.63778	-1.57013
C	2.10451	2.20207	-3.49168
C	1.31100	4.70092	-0.08216
C	-1.17791	4.50531	-0.36850
C	-5.77046	-0.44555	-1.92797
C	-5.16009	1.98122	-1.67027
C	-4.27401	-2.04127	-2.90726
C	-2.00463	-1.39365	-3.77725
H	-2.34030	-3.22655	4.38096
C	-2.22147	-1.19527	5.09963
C	-1.01659	-4.07894	1.90269
C	-2.95948	-3.03180	0.85001
H	-2.09049	0.92490	5.48907
C	-2.67303	2.79233	3.11768
C	-0.24195	2.51397	3.12796
C	4.16432	-3.20437	-0.31149
C	5.44092	-2.50303	1.78094
H	3.44784	-2.02964	3.58455
H	3.50900	-0.44555	2.80355
H	1.93314	-1.20047	3.12527
C	2.96654	-4.22937	-2.27957
H	0.38026	-4.26357	-1.43857
H	-0.40431	-2.89088	-0.64590
H	0.43624	-2.66487	-2.19371
H	3.88559	3.45624	-1.89481
C	2.63305	4.44944	-0.45157
H	1.63088	1.21296	-3.39764
H	1.72218	2.65008	-4.42118

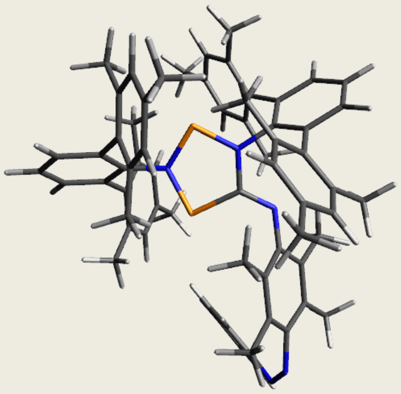

H	3.18601	2.05366	-3.60851
H	1.10899	5.35539	0.76902
H	-1.19336	5.44051	0.20479
H	-1.84012	4.61868	-1.23803
H	-1.61282	3.72702	0.27878
H	-6.73515	-0.20151	-1.47487
C	-5.51776	-1.75870	-2.33340
H	-4.39633	2.39715	-0.99540
H	-5.21345	2.65916	-2.53563
H	-6.12653	2.01342	-1.15089
H	-4.05542	-3.05994	-3.23884
H	-1.95616	-2.46385	-4.01544
H	-1.89132	-0.82506	-4.71343
H	-1.13766	-1.14491	-3.14613
H	-2.44652	-1.45868	6.13421
C	-1.14776	-5.15057	1.01632
C	0.07700	-4.11602	2.93751
C	-3.04637	-4.12633	-0.02085
C	-4.01134	-1.96036	0.77521
C	-2.49208	4.17795	3.06531
C	-4.06559	2.21882	3.13999
C	-0.11078	3.90574	3.08822
C	0.98099	1.64227	3.17606
N	5.35438	-3.64403	-0.95559
H	5.79545	-1.46293	1.86588
H	5.26163	-2.86235	2.80502
H	6.25521	-3.09814	1.35389
H	2.18281	-5.00102	-2.28971
H	2.77089	-3.57286	-3.14375
H	3.94006	-4.70883	-2.43516
C	3.78545	5.05623	0.30276
C	-6.53748	-2.84596	-2.13061
H	-0.43299	-5.97546	1.08474
C	-2.14661	-5.19049	0.03724
H	0.42564	-3.10686	3.18317
H	-0.26893	-4.59378	3.86826
H	0.93313	-4.69637	2.56801
H	-3.85412	-4.14584	-0.75724
H	-4.13521	-1.43659	1.73185
H	-3.76783	-1.20471	0.00978
H	-4.98149	-2.38800	0.49139
H	-3.37407	4.82315	3.02594
C	-1.21915	4.75658	3.06966
H	-4.19996	1.45105	2.36212
H	-4.28858	1.72981	4.10012
H	-4.81293	3.00591	2.97568
H	0.89479	4.33358	3.07824
H	1.02980	0.95983	2.31647
H	1.89498	2.24980	3.17912
H	0.98295	1.00720	4.07495
N	6.31988	-2.86227	-1.17332
H	4.23372	5.88918	-0.26196
H	3.46186	5.45101	1.27554
H	4.58342	4.31968	0.47293
H	-7.55046	-2.43382	-2.02905
H	-6.53605	-3.55890	-2.96716
H	-6.32461	-3.42277	-1.21500
C	-2.22482	-6.33013	-0.94220
C	-1.04898	6.25181	3.06974
C	6.23979	-1.44912	-0.96430

H	-3.23246	-6.42429	-1.37007
H	-1.52461	-6.17524	-1.77981
H	-1.95826	-7.28679	-0.47115
H	-1.86781	6.75071	2.53251
H	-1.04516	6.64766	4.09810
H	-0.09892	6.55022	2.60455
C	5.10874	-0.65441	-1.23465
C	7.43990	-0.82473	-0.59069
H	4.17376	-1.11078	-1.55486
C	5.17058	0.72719	-1.08998
C	7.50696	0.55664	-0.41803
H	8.31838	-1.45032	-0.42620
H	4.29565	1.34908	-1.27727
C	6.36419	1.30768	-0.66812
H	8.42739	1.05170	-0.10857
F	6.41608	2.65907	-0.50912

6.5.19 *Z*-5B-I^{4}

139

***Z*-5B-I^{4}** @ PBE-D3/def2-TZVP

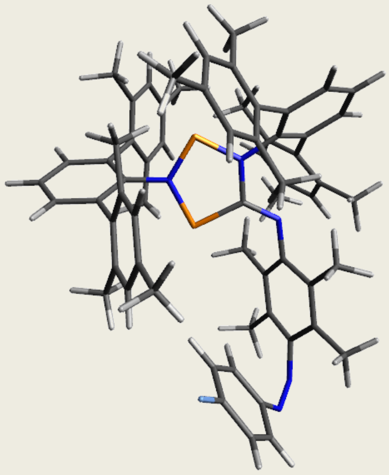

P	0.30277	-0.03161	-1.09274
N	-1.15313	-0.98903	-1.07773
C	-0.05218	0.68032	0.52262
P	-2.18781	-0.95874	0.23740
C	-1.49915	-1.73619	-2.26615
N	-1.25735	0.16875	1.09634
N	0.66639	1.50176	1.21983
C	-0.66851	-2.80083	-2.67981
C	-2.63473	-1.36853	-3.02086
C	-1.62257	0.52510	2.44548
C	1.79066	2.13111	0.69995
C	-0.97784	-3.46710	-3.87331
C	0.48070	-3.31303	-1.87938
C	-2.90819	-2.07574	-4.20191
C	-3.57825	-0.26352	-2.67305
C	-1.72213	-0.49249	3.41800
C	-1.86319	1.87735	2.78598
C	1.75796	2.91184	-0.49020
C	2.98483	2.07361	1.47203
H	-0.33720	-4.29507	-4.18130
C	-2.08601	-3.11034	-4.63570
C	0.22608	-4.15690	-0.78101
C	1.80802	-3.06508	-2.29290
H	-3.78347	-1.78392	-4.78486
C	-3.30004	1.05550	-3.09106
C	-4.82540	-0.57384	-2.08987
C	-2.02421	-0.13254	4.74097
C	-1.53655	-1.95212	3.15654
C	-2.14862	2.18479	4.12273
C	-1.91801	2.99310	1.80115
C	2.95388	3.45982	-0.98484
C	0.47552	3.18868	-1.22446
C	4.18050	2.59316	0.96372
C	2.96480	1.40430	2.81905
H	-2.31292	-3.64238	-5.56078
C	1.30997	-4.70138	-0.08189
C	-1.17884	-4.50534	-0.36863

C	2.85729	-3.63827	-1.56943
C	2.10449	-2.20247	-3.49116
C	-4.27415	2.04147	-2.90714
C	-2.00480	1.39386	-3.77720
C	-5.77054	0.44576	-1.92774
C	-5.16017	-1.98103	-1.67012
H	-2.09058	-0.92425	5.48911
C	-2.22117	1.19592	5.09954
C	-0.24226	-2.51367	3.12795
C	-2.67337	-2.79181	3.11785
H	-2.33959	3.22719	4.38075
C	-2.95898	3.03229	0.84979
C	-1.01589	4.07916	1.90238
C	4.16476	3.20392	-0.31151
C	2.96716	4.22902	-2.27967
H	0.43666	2.66452	-2.19384
H	-0.40388	2.89098	-0.64610
H	0.38093	4.26338	-1.43905
C	5.44121	2.50246	1.78096
H	3.50904	0.44540	2.80371
H	3.44789	2.02957	3.58456
H	1.93318	1.20044	3.12522
H	1.10769	-5.35592	0.76916
C	2.63215	-4.44998	-0.45101
H	-1.19450	-5.44056	0.20461
H	-1.61369	-3.72700	0.27862
H	-1.84094	-4.61856	-1.23826
H	3.88510	-3.45677	-1.89383
H	3.18602	-2.05406	-3.60767
H	1.72246	-2.65056	-4.42075
H	1.63081	-1.21337	-3.39734
H	-4.05556	3.06014	-3.23870
C	-5.51787	1.75890	-2.33320
H	-1.13779	1.14475	-3.14628
H	-1.89171	0.82558	-4.71360
H	-1.95617	2.46414	-4.01503
H	-6.73519	0.20172	-1.47456
H	-6.12640	-2.01317	-1.15035
H	-5.21397	-2.65886	-2.53553
H	-4.39618	-2.39713	-0.99561
H	-2.44613	1.45943	6.13412
C	-0.11127	-3.90549	3.08818
C	0.98080	-1.64215	3.17599
C	-2.49259	-4.17741	3.06549
C	-4.06585	-2.21809	3.14028
C	-3.04570	4.12678	-0.02111
C	-4.01100	1.96100	0.77504
C	-1.14694	5.15080	1.01599
C	0.07780	4.11618	2.93711
N	5.35490	3.64345	-0.95556
H	2.77121	3.57259	-3.14384
H	2.18366	5.00092	-2.28971
H	3.94081	4.70819	-2.43535
H	5.26192	2.86181	2.80504
H	5.79561	1.46233	1.86590
H	6.25557	3.09749	1.35393
C	3.78426	-5.05719	0.30343
C	-6.53757	2.84617	-2.13040
H	0.89425	-4.33346	3.07813
C	-1.21969	-4.75619	3.06970

```
H            0.98268       -1.00675        4.07465
H            1.89470       -2.24983        3.17948
H            1.02992       -0.96005        2.31616
H           -3.37468       -4.82250        3.02622
H           -4.20004       -1.45002        2.36268
H           -4.81329       -3.00503        2.97563
H           -4.28883       -1.72942        4.10058
H           -3.85345        4.14640       -0.75749
C           -2.14579        5.19083        0.03693
H           -4.13476        1.43710        1.73162
H           -4.98115        2.38883        0.49150
H           -3.76775        1.20546        0.00942
H           -0.43203        5.97558        1.08435
H            0.42521        3.10694        3.18414
H            0.93467        4.69491        2.56678
H           -0.26749        4.59570        3.86719
N            6.32032        2.86159       -1.17325
H            4.22899       -5.89363       -0.25896
H            4.58462       -4.32230        0.46936
H            3.46139       -5.44745        1.27828
H           -6.53757        3.55795       -2.96794
H           -7.55028        2.43390       -2.02677
H           -6.32346        3.42433       -1.21593
C           -1.04985       -6.25146        3.06966
C           -2.22385        6.33042       -0.94257
C            6.24006        1.44843       -0.96428
H           -1.86393       -6.74960        2.52450
H           -0.09572       -6.54951        2.61271
H           -1.05562       -6.64850        4.09755
H           -1.52418        6.17504       -1.78053
H           -3.23167        6.42513       -1.36993
H           -1.95649        7.28698       -0.47175
C            7.44011        0.82389       -0.59073
C            5.10894        0.65385       -1.23472
H            8.31865        1.44937       -0.42616
C            7.50705       -0.55751       -0.41823
C            5.17066       -0.72777       -1.09023
H            4.17399        1.11034       -1.55487
H            8.42744       -1.05268       -0.10884
C            6.36422       -1.30842       -0.66845
H            4.29568       -1.34956       -1.27760
F            6.41602       -2.65984       -0.50968
```

6.5.20 *Z*-5B-I[5]

```
139
Z-5B-I5 @ PBE-D3/def2-TZVP
P            0.68448       -0.38424       -1.53525
N            2.27949       -0.00077       -0.96277
C           -0.05028       -0.11875        0.08142
P            2.54959        0.39131        0.64394
C            3.36651        0.01603       -1.91423
N            0.91134        0.25176        1.07136
N           -1.28053       -0.30347        0.44435
C            4.03442        1.22568       -2.19937
C            3.71485       -1.18199       -2.57797
C            0.51391        0.41198        2.44799
C           -2.31960       -0.52026       -0.45018
```

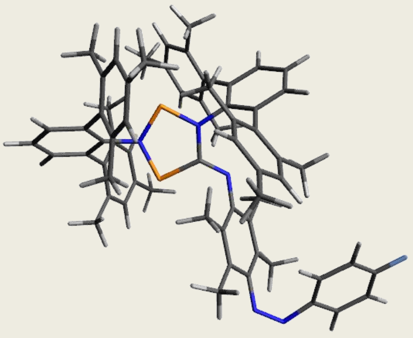

C	5.05643	1.20910	-3.16139
C	3.71187	2.54138	-1.56997
C	4.73092	-1.14388	-3.54025
C	3.07422	-2.48718	-2.24990
C	-0.46958	1.36660	2.80115
C	1.11573	-0.39846	3.43453
C	-3.19308	-1.60887	-0.16977
C	-2.59379	0.34170	-1.55037
H	5.56846	2.14667	-3.38484
C	5.40166	0.04094	-3.83377
C	4.53664	3.04718	-0.54415
C	2.67655	3.33872	-2.10561
H	5.00004	-2.07288	-4.04554
C	2.07813	-3.02694	-3.09038
C	3.50962	-3.19740	-1.11433
C	-0.86856	1.44615	4.14187
C	-1.05617	2.35999	1.85928
C	0.69058	-0.26906	4.76626
C	2.19223	-1.40411	3.18311
C	-4.31170	-1.85422	-0.97313
C	-2.89452	-2.49827	1.00673
C	-3.69271	0.06913	-2.38038
C	-1.79462	1.58965	-1.80650
H	6.19339	0.05348	-4.58433
C	4.30552	4.34270	-0.06500
C	5.66592	2.22751	0.02285
C	2.48492	4.62652	-1.59765
C	1.81654	2.83740	-3.23364
C	1.47791	-4.23714	-2.73076
C	1.66290	-2.32844	-4.35765
C	2.89407	-4.41493	-0.80468
C	4.62441	-2.67578	-0.24793
H	-1.62297	2.18698	4.40970
C	-0.30528	0.63327	5.12106
C	-2.44930	2.36639	1.60851
C	-0.24220	3.38293	1.32822
H	1.15534	-0.90623	5.52058
C	3.52055	-1.10036	3.56104
C	1.86202	-2.70373	2.74525
C	-4.57322	-0.98181	-2.05651
C	-5.15825	-3.07435	-0.71923
H	-2.27833	-1.95728	1.73464
H	-3.81420	-2.84294	1.49778
H	-2.32635	-3.39594	0.70817
C	-4.00151	0.94889	-3.56331
H	-1.09810	1.46975	-2.65397
H	-2.46115	2.42812	-2.05334
H	-1.20315	1.88277	-0.93357
H	4.94425	4.73005	0.73335
C	3.29023	5.15128	-0.58087
H	6.12799	2.73906	0.87707
H	5.32057	1.23927	0.36349
H	6.44810	2.04204	-0.72887
H	1.68537	5.24143	-2.01997
H	2.42619	2.55764	-4.10646
H	1.25159	1.93916	-2.93857
H	1.09549	3.60360	-3.54576
H	0.68623	-4.64076	-3.36761
C	1.85837	-4.93900	-1.58206
H	0.76183	-2.79127	-4.78005

H	1.44920	-1.26398	-4.18328
H	2.45735	-2.37464	-5.11886
H	3.23757	-4.96720	0.07327
H	4.25343	-1.94135	0.48518
H	5.08597	-3.49070	0.32315
H	5.40390	-2.18063	-0.84329
H	-0.63099	0.71461	6.15913
C	-2.98404	3.36884	0.79590
C	-3.37168	1.32934	2.19372
C	-0.82515	4.36402	0.51446
C	1.22285	3.48257	1.65064
C	4.49011	-2.10577	3.50149
C	3.89923	0.28011	4.02897
C	2.86116	-3.68230	2.72308
C	0.45687	-3.04720	2.33915
N	-5.67549	-1.17653	-2.93314
H	-4.52269	-3.94418	-0.50141
H	-5.82810	-2.93665	0.14531
H	-5.78738	-3.32332	-1.58143
H	-3.08534	1.23947	-4.09510
H	-4.67305	0.42838	-4.25830
H	-4.50625	1.88276	-3.26046
C	3.04815	6.53730	-0.04773
C	1.15861	-6.21051	-1.18365
H	-4.05810	3.35660	0.58855
C	-2.19060	4.37232	0.22878
H	-4.21834	1.14393	1.51902
H	-2.85167	0.37840	2.35349
H	-3.78457	1.65999	3.16039
H	-0.18612	5.15169	0.10557
H	1.54300	4.53171	1.68053
H	1.46338	3.02314	2.61818
H	1.84364	2.98766	0.88493
H	5.51857	-1.86297	3.78286
C	4.17719	-3.40991	3.10436
H	3.41673	0.53122	4.98543
H	4.98583	0.36140	4.16216
H	3.58375	1.05223	3.30981
H	2.59506	-4.69206	2.40072
H	0.10983	-2.42183	1.50507
H	0.38417	-4.09804	2.03026
H	-0.25043	-2.87905	3.16576
N	-6.86581	-1.24394	-2.52667
H	2.11851	6.57921	0.54318
H	3.86931	6.86671	0.60253
H	2.94131	7.26723	-0.86345
H	1.81473	-6.86174	-0.58956
H	0.26993	-5.99040	-0.56963
H	0.81422	-6.77469	-2.06144
C	-2.78894	5.40509	-0.68739
C	5.22691	-4.48826	3.09771
C	-7.25609	-0.90795	-1.19360
H	-2.15966	6.30377	-0.74604
H	-2.89236	5.00820	-1.71094
H	-3.79233	5.70692	-0.35505
H	5.37668	-4.90005	4.10866
H	4.94152	-5.32330	2.44306
H	6.19880	-4.10136	2.75915
C	-6.74909	0.18991	-0.47324
C	-8.33613	-1.63280	-0.66668

```
H          -5.92901        0.77925       -0.88092
C          -7.29804        0.53510        0.75806
C          -8.86705       -1.32027        0.58275
H          -8.74663       -2.45343       -1.25678
H          -6.92834        1.38988        1.32532
C          -8.33578       -0.23748        1.27547
H          -9.69207       -1.88807        1.01286
F          -8.85535        0.08966        2.48607
```

6.5.21 *Z*-5B-I^6

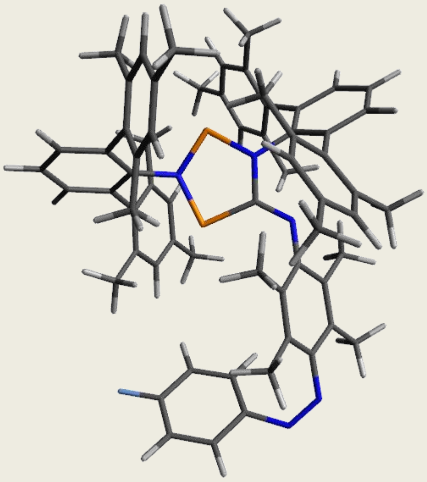

```
139
Z-5B-I^6 @ PBE-D3/def2-TZVP
P           0.49035       -0.46457        0.68328
N          -1.16451       -0.82890        1.08534
C           0.01305        0.53009       -0.73744
P          -2.42484       -0.25860        0.14029
C          -1.45659       -1.58491        2.28155
N          -1.40377        0.56933       -0.92912
N           0.76573        1.11726       -1.61432
C          -2.19497       -0.98965        3.32784
C          -0.97729       -2.90874        2.39549
C          -1.95926        1.22683       -2.08666
C           2.13891        1.26037       -1.48525
C          -2.44827       -1.75024        4.48010
C          -2.70710        0.41399        3.32752
C          -1.24975       -3.62320        3.56909
C          -0.23522       -3.59390        1.29989
C          -2.70548        0.46806       -3.01316
C          -1.74785        2.61132       -2.28898
C           2.92670        0.96750       -2.63506
C           2.76422        1.79921       -0.32308
H          -3.01312       -1.28454        5.28948
C          -1.98121       -3.05422        4.60776
C          -4.08236        0.65520        3.12512
C          -1.84985        1.47196        3.70243
H          -0.88532       -4.64908        3.64446
C           1.16158       -3.77110        1.38647
C          -0.95699       -4.13186        0.21568
C          -3.20027        1.10551       -4.16217
C          -3.02883       -0.98438       -2.87281
C          -2.25196        3.19666       -3.45760
C          -1.09151        3.50976       -1.29910
C           4.31896        1.07348       -2.57604
C           2.25392        0.51172       -3.90081
C           4.16810        1.85206       -0.24508
C           1.95337        2.36806        0.80912
H          -2.18598       -3.62515        5.51462
C          -4.57613        1.95397        3.29687
C          -5.02293       -0.46000        2.75049
C          -2.38887        2.75134        3.86518
C          -0.38953        1.23084        3.97181
C           1.81879       -4.42912        0.34142
C           1.94953       -3.24641        2.55616
C          -0.25631       -4.78109       -0.80549
C          -2.45795       -4.04110        0.16056
H          -3.76931        0.51031       -4.87836
C          -2.96508        2.45550       -4.39556
```

C	-4.33812	-1.36331	-2.49678
C	-2.10003	-1.96478	-3.28097
H	-2.09178	4.26505	-3.60772
C	0.09514	4.20133	-1.64023
C	-1.72745	3.77280	-0.06739
C	4.93351	1.42846	-1.35389
C	5.17607	0.71224	-3.76019
H	2.38420	-0.57117	-4.06518
H	1.17823	0.71132	-3.84958
H	2.67146	1.01806	-4.78288
C	4.82208	2.41995	0.98646
H	2.32201	3.36770	1.08187
H	0.89702	2.47415	0.54590
H	2.01625	1.74519	1.71735
H	-5.64181	2.13731	3.13513
C	-3.74915	3.01509	3.67303
H	-4.65267	-1.03080	1.88536
H	-5.14287	-1.18231	3.57224
H	-6.01523	-0.06395	2.49861
H	-1.72222	3.56454	4.16493
H	0.13468	0.88544	3.06681
H	0.10083	2.15020	4.31640
H	-0.24755	0.45277	4.73737
H	2.90095	-4.57258	0.40753
C	1.13395	-4.92151	-0.77394
H	1.45205	-3.45045	3.51367
H	2.95244	-3.68864	2.58906
H	2.06801	-2.15249	2.48654
H	-0.81912	-5.20035	-1.64332
H	-2.79030	-3.06448	-0.22647
H	-2.86906	-4.80614	-0.50885
H	-2.90726	-4.16887	1.15523
H	-3.34931	2.93326	-5.29806
C	-4.69528	-2.71428	-2.54704
C	-5.34902	-0.33682	-2.05761
C	-2.50831	-3.30171	-3.33158
C	-0.70597	-1.58493	-3.69279
C	0.64541	5.09425	-0.71741
C	0.77894	4.00978	-2.96822
C	-1.13389	4.67292	0.82762
C	-3.05201	3.15910	0.29125
N	6.35432	1.46732	-1.34916
H	5.20391	-0.37832	-3.92362
H	4.78699	1.15827	-4.68639
H	6.20756	1.05256	-3.60761
H	5.89072	2.60748	0.83975
H	4.35156	3.37196	1.27239
H	4.71765	1.74193	1.84918
C	-4.29284	4.40829	3.83628
C	1.87180	-5.57638	-1.91052
H	-5.70577	-3.00334	-2.24508
C	-3.80123	-3.69814	-2.98103
H	-5.63854	0.32707	-2.88607
H	-6.25642	-0.82283	-1.67621
H	-4.94899	0.31339	-1.26420
H	-1.78847	-4.05467	-3.66275
H	-0.16702	-1.06908	-2.88632
H	-0.12647	-2.47282	-3.97701
H	-0.71847	-0.89168	-4.54779
H	1.57543	5.60728	-0.97794

```
C          0.05694         5.33542         0.52876
H          1.86150         4.16496        -2.86762
H          0.61169         3.00100        -3.36103
H          0.40937         4.73150        -3.71451
H         -1.63244         4.86797         1.78099
H         -3.65336         3.85609         0.88862
H         -3.63213         2.88205        -0.59851
H         -2.92802         2.25048         0.90414
N          7.04872         0.86982        -0.48462
H         -5.38604         4.40388         3.94056
H         -3.86364         4.90577         4.71776
H         -4.04703         5.03451         2.96252
H          2.80262        -6.04924        -1.56819
H          1.25581        -6.34202        -2.40258
H          2.14556        -4.83377        -2.67769
C         -4.22387        -5.13947        -3.07518
C          0.70638         6.25652         1.52575
C          6.50383        -0.08629         0.42671
H         -4.76226        -5.33224        -4.01700
H         -3.35769        -5.81513        -3.04972
H         -4.90053        -5.41499        -2.25364
H         -0.02551         6.65468         2.24209
H          1.47801         5.72393         2.10616
H          1.20160         7.10343         1.03024
C          5.53617        -1.04653         0.07732
C          7.12200        -0.16245         1.68433
H          5.06175        -1.01668        -0.90227
C          5.18714        -2.04987         0.97511
C          6.75117        -1.13895         2.60614
H          7.89971         0.56381         1.92494
H          4.44680        -2.80473         0.71221
C          5.78933        -2.07048         2.23091
H          7.21037        -1.19971         3.59281
F          5.43882        -3.04508         3.11220
```

6.5.22 *Z*-5B-I[7]

```
139
Z-5B-I7 @ PBE-D3/def2-TZVP
P         -0.49032        -0.46452         0.68332
N          1.16450        -0.82891         1.08533
C         -0.01299         0.53009        -0.73743
P          2.42487        -0.25870         0.14025
C          1.45664        -1.58489         2.28155
N          1.40379         0.56927        -0.92918
N         -0.76571         1.11729        -1.61426
C          2.19512        -0.98965         3.32776
C          0.97727        -2.90870         2.39558
C          1.95918         1.22666        -2.08680
C         -2.13887         1.26047        -1.48514
C          2.44844        -1.75020         4.48005
C          2.70736         0.41396         3.32740
C          1.24976        -3.62312         3.56918
C          0.23512        -3.59386         1.30003
C          2.70514         0.46781        -3.01343
C          1.74784         2.61117        -2.28915
C         -2.76412         1.79937        -0.32298
C         -2.92671         0.96763        -2.63495
```

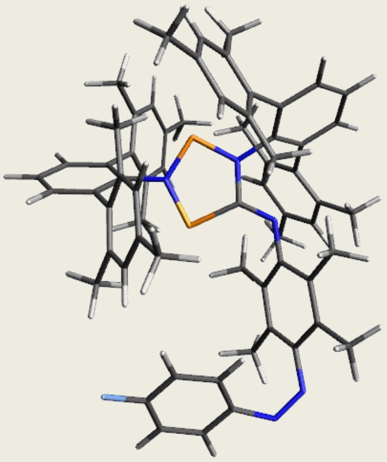

H	3.01337	-1.28450	5.28936
C	1.98132	-3.05415	4.60779
C	1.85020	1.47199	3.70229
C	4.08265	0.65506	3.12498
H	0.88531	-4.64899	3.64460
C	0.95674	-4.13183	0.21578
C	-1.16170	-3.77107	1.38679
C	3.19972	1.10514	-4.16259
C	3.02848	-0.98464	-2.87302
C	2.25174	3.19641	-3.45791
C	1.09175	3.50968	-1.29916
C	-4.16799	1.85232	-0.24495
C	-1.95321	2.36816	0.80921
C	-4.31895	1.07374	-2.57592
C	-2.25392	0.51176	-3.90066
H	2.18610	-3.62506	5.51465
C	2.38932	2.75135	3.86500
C	0.38987	1.23099	3.97172
C	4.57651	1.95379	3.29671
C	5.02311	-0.46022	2.75033
C	0.25590	-4.78111	-0.80529
C	2.45768	-4.04105	0.16041
C	-1.81905	-4.42910	0.34189
C	-1.94938	-3.24640	2.55668
H	3.76854	0.50986	-4.87887
C	2.96457	2.45514	-4.39600
C	2.09963	-1.96504	-3.28107
C	4.33778	-1.36357	-2.49707
H	2.09162	4.26481	-3.60805
C	1.72782	3.77257	-0.06752
C	-0.09491	4.20138	-1.64012
C	-4.93345	1.42879	-1.35375
C	-4.82191	2.42023	0.98661
H	-2.32176	3.36785	1.08195
H	-2.01616	1.74531	1.71744
H	-0.89686	2.47415	0.54599
C	-5.17615	0.71258	-3.76004
H	-1.17831	0.71189	-3.84967
H	-2.38366	-0.57126	-4.06462
H	-2.67186	1.01757	-4.78284
H	1.72273	3.56460	4.16476
C	3.74961	3.01499	3.67282
H	0.24784	0.45294	4.73728
H	-0.10041	2.15040	4.31630
H	-0.13440	0.88564	3.06673
H	5.64220	2.13706	3.13496
H	5.14242	-1.18295	3.57179
H	4.65313	-1.03050	1.88473
H	6.01565	-0.06433	2.49913
H	0.81859	-5.20038	-1.64321
C	-1.13432	-4.92155	-0.77356
H	2.86871	-4.80633	-0.50880
H	2.78995	-3.06457	-0.22703
H	2.90715	-4.16847	1.15505
H	-2.90120	-4.57255	0.40816
H	-1.45263	-3.45229	3.51418
H	-2.06613	-2.15223	2.48824
H	-2.95296	-3.68715	2.58870
H	3.34862	2.93283	-5.29861
C	2.50788	-3.30197	-3.33166

C	0.70554	-1.58517	-3.69279
C	4.69492	-2.71455	-2.54730
C	5.34875	-0.33706	-2.05808
C	1.13442	4.67269	0.82763
C	3.05234	3.15873	0.29103
C	-0.64498	5.09428	-0.71721
C	-0.77889	4.00994	-2.96804
N	-6.35425	1.46777	-1.34900
H	-4.35119	3.37209	1.27268
H	-5.89051	2.60800	0.83986
H	-4.71768	1.74208	1.84926
H	-4.78705	1.15854	-4.68626
H	-5.20415	-0.37798	-3.92343
H	-6.20757	1.05305	-3.60742
C	4.29341	4.40817	3.83594
C	-1.87242	-5.57642	-1.90997
H	1.78801	-4.05494	-3.66273
C	3.80083	-3.69841	-2.98120
H	0.16688	-1.06871	-2.88651
H	0.71798	-0.89246	-4.54823
H	0.12581	-2.47312	-3.97633
H	5.70543	-3.00361	-2.24540
H	4.94885	0.31320	-1.26465
H	6.25620	-0.82306	-1.67677
H	5.63816	0.32677	-2.88662
H	1.63307	4.86759	1.78097
C	-0.05635	5.33533	0.52893
H	3.65408	3.85592	0.88778
H	2.92829	2.25052	0.90450
H	3.63211	2.88102	-0.59876
H	-1.57498	5.60741	-0.97761
H	-0.40945	4.73175	-3.71430
H	-0.61168	3.00120	-3.36097
H	-1.86144	4.16509	-2.86726
N	-7.04868	0.87034	-0.48443
H	3.86358	4.90608	4.71687
H	5.38652	4.40362	3.94108
H	4.04838	5.03406	2.96172
H	-1.25435	-6.33778	-2.40603
H	-2.80012	-6.05454	-1.56643
H	-2.15184	-4.83279	-2.67411
C	4.22343	-5.13975	-3.07532
C	-0.70560	6.25654	1.52596
C	-6.50386	-0.08589	0.42681
H	4.76157	-5.33260	-4.01728
H	4.90029	-5.41521	-2.25394
H	3.35724	-5.81539	-3.04959
H	-1.47965	5.72497	2.10405
H	0.02583	6.65196	2.24428
H	-1.19785	7.10532	1.03069
C	-5.53630	-1.04621	0.07736
C	-7.12199	-0.16204	1.68445
H	-5.06188	-1.01635	-0.90224
C	-5.18735	-2.04963	0.97508
C	-6.75123	-1.13862	2.60621
H	-7.89961	0.56429	1.92512
H	-4.44710	-2.80455	0.71213
C	-5.78951	-2.07024	2.23090
H	-7.21039	-1.19938	3.59289
F	-5.43906	-3.04491	3.11213

6.5.23 TS-B

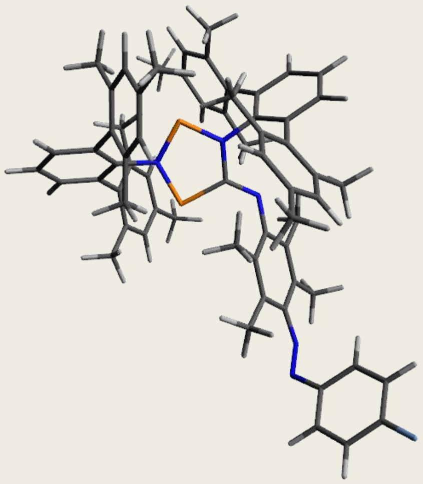

```
139
TS-B @ UPBE-D3/def2-TZVP
P        0.43009    -0.52430    -1.29322
N        2.04178     0.12077    -1.22415
C        0.16147    -0.25887     0.45824
P        2.69184     0.66698     0.22131
C        2.79766     0.21916    -2.45141
N        1.28583     0.32735     1.11214
N       -0.85772    -0.62987     1.17441
C        3.17077     1.48542    -2.94913
C        3.11515    -0.96373    -3.15658
C        1.27225     0.53073     2.53942
C       -2.05626    -1.06583     0.65046
C        3.86710     1.54075    -4.16665
C        2.85132     2.78551    -2.28640
C        3.79810    -0.85497    -4.37371
C        2.79308    -2.31632    -2.62012
C        2.24529    -0.11179     3.33419
C        0.29158     1.36266     3.12984
C       -2.62303    -2.23939     1.24345
C       -2.78345    -0.36550    -0.36469
H        4.15005     2.52110    -4.55385
C        4.17480     0.38659    -4.88020
C        3.85345     3.46715    -1.56565
C        1.59798     3.39710    -2.50990
H        4.04658    -1.77144    -4.91139
C        1.69176    -3.04198    -3.12106
C        3.62744    -2.87820    -1.63453
C        2.19260     0.05248     4.72756
C        3.35599    -0.96379     2.81131
C        0.27449     1.48307     4.52544
C       -0.67487     2.19357     2.35990
C       -3.82114    -2.75441     0.77787
C       -1.88783    -2.91798     2.36781
C       -3.99053    -0.87771    -0.85006
C       -2.31917     0.97698    -0.86794
H        4.70913     0.45445    -5.82898
C        3.58195     4.75049    -1.07482
C        5.20704     2.84716    -1.33717
C        1.37426     4.68089    -2.00518
C        0.52798     2.70458    -3.30908
C        1.39848    -4.28938    -2.56273
C        0.84001    -2.49692    -4.23625
C        3.30604    -4.13828    -1.11855
C        4.85375    -2.15495    -1.14600
H        2.94296    -0.45537     5.33585
C        1.20693     0.82891     5.32564
C        3.13422    -2.32740     2.52674
C        4.66801    -0.43688     2.77575
H       -0.47956     2.12961     4.97604
C       -2.06764     1.99481     2.51341
C       -0.20091     3.27206     1.58238
C       -4.54605    -2.06927    -0.26230
C       -4.39502    -4.01822     1.35734
H       -1.28038    -3.76428     2.00453
H       -1.20051    -2.21334     2.85007
H       -2.58338    -3.31740     3.11802
```

C	-4.68804	-0.18277	-1.98817
H	-3.16513	1.67641	-0.92110
H	-1.56666	1.41984	-0.20879
H	-1.88118	0.91895	-1.87880
H	4.35964	5.27409	-0.51236
C	2.35314	5.37873	-1.28911
H	5.12568	1.83970	-0.90103
H	5.76418	2.73434	-2.27968
H	5.80869	3.46466	-0.65775
H	0.40456	5.15231	-2.18717
H	0.89436	2.42035	-4.30729
H	0.19725	1.77693	-2.81540
H	-0.34892	3.35274	-3.43271
H	0.52786	-4.83843	-2.93118
C	2.18157	-4.84800	-1.54679
H	-0.05048	-3.12052	-4.38654
H	0.50555	-1.47036	-4.02594
H	1.39588	-2.46332	-5.18609
H	3.95731	-4.57419	-0.35722
H	5.35674	-1.61366	-1.95918
H	4.60317	-1.41817	-0.36562
H	5.56772	-2.85885	-0.70112
H	1.17504	0.93863	6.41065
C	4.23366	-3.14413	2.24198
C	1.74847	-2.90698	2.55847
C	5.73280	-1.28721	2.46398
C	4.93058	1.01577	3.07415
C	-2.94937	2.84976	1.84758
C	-2.62883	0.89293	3.37360
C	-1.12501	4.09735	0.92774
C	1.26332	3.59770	1.47990
N	-5.73918	-2.60448	-0.58630
H	-4.84022	-3.84184	2.35131
H	-5.18581	-4.41307	0.70925
H	-3.61852	-4.78566	1.48653
H	-5.25144	-0.89651	-2.60141
H	-5.41543	0.56915	-1.63573
H	-3.96686	0.33396	-2.63311
C	2.07395	6.75495	-0.74880
C	1.81163	-6.16515	-0.91983
H	4.05801	-4.20405	2.04175
C	5.53981	-2.64982	2.21332
H	1.28437	-2.77901	3.54834
H	1.08196	-2.40942	1.84031
H	1.76663	-3.97848	2.32072
H	6.74406	-0.87275	2.42621
H	5.98261	1.26743	2.88730
H	4.30527	1.67672	2.45351
H	4.69921	1.26338	4.12094
H	-4.02416	2.67625	1.95150
C	-2.50175	3.90009	1.03894
H	-2.80768	1.24205	4.40337
H	-3.59045	0.54670	2.97113
H	-1.94836	0.03561	3.41994
H	-0.74855	4.92858	0.32504
H	1.82541	3.24706	2.35521
H	1.72199	3.14220	0.58608
H	1.41377	4.68076	1.38790
N	-6.62229	-2.19063	-1.44519
H	1.33939	6.71852	0.07222

```
H          2.98572         7.22573        -0.35817
H          1.65334         7.41172        -1.52439
H          1.08582        -6.01744        -0.10346
H          1.34555        -6.84320        -1.64821
H          2.69004        -6.66786        -0.49200
C          6.70786        -3.55513         1.92902
C         -3.47628         4.76578         0.28757
C         -7.62837        -1.35439        -1.03194
H          7.44179        -3.06932         1.26975
H          7.23580        -3.82294         2.85818
H          6.38449        -4.49114         1.45332
H         -3.02842         5.73060         0.01255
H         -3.79842         4.27371        -0.64501
H         -4.38228         4.96063         0.87860
C         -7.72442        -0.82593         0.28363
C         -8.61080        -0.98851        -1.98673
H         -6.97975        -1.11660         1.02474
C         -8.75616         0.04048         0.61998
C         -9.63740        -0.11836        -1.65217
H         -8.53256        -1.40486        -2.99171
H         -8.84836         0.45310         1.62548
C         -9.69559         0.38569        -0.35128
H        -10.39606         0.17551        -2.37788
F        -10.70274         1.23677        -0.02041
```

6.5.24 TS-H

```
139
TS-H @ UPBE-D3/def2-TZVP
P         -0.20583        -0.85032         0.17425
N          1.22473        -1.13765        -0.90575
C          0.25280         0.96714         0.42015
P          1.92972        -0.83056         0.71637
C          1.39044        -2.44977        -1.44467
N          1.62362         0.94062         0.64386
N         -0.51763         1.95911         0.65448
C          2.69876        -2.98621        -1.58304
C          0.28708        -3.21289        -1.91109
C          2.33230         2.02751         1.23867
C         -1.87235         1.95251         0.34911
C          2.86836        -4.26818        -2.12084
C          3.94427        -2.23382        -1.26162
C          0.51010        -4.49379        -2.43711
C         -1.12267        -2.72361        -1.99198
C          3.00206         1.82690         2.46753
C          2.31561         3.30995         0.63903
C         -2.79258         2.20683         1.40832
C         -2.32516         1.82448        -0.99463
H          3.88689        -4.64701        -2.22686
C          1.78603        -5.03732        -2.53578
C          4.76226        -2.63714        -0.18204
C          4.39328        -1.23539        -2.15304
H         -0.35649        -5.05398        -2.79427
C         -1.49302        -1.90835        -3.08359
C         -2.11537        -3.22303        -1.11958
C          3.61206         2.91671         3.10063
C          3.10993         0.47976         3.09538
C          2.93037         4.37192         1.31741
```

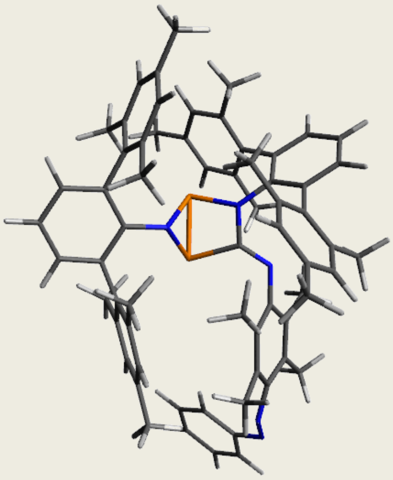

C	1.74671	3.59173	-0.70842
C	-4.16355	2.12868	1.17907
C	-2.25489	2.51153	2.78219
C	-3.69358	1.81550	-1.24805
C	-1.36258	1.73071	-2.14619
H	1.93689	-6.03295	-2.95433
C	6.00651	-2.02451	-0.01044
C	4.31115	-3.70355	0.77901
C	5.64145	-0.64251	-1.93483
C	3.56692	-0.84939	-3.35001
C	-2.84474	-1.60983	-3.28104
C	-0.45490	-1.39992	-4.04821
C	-3.45190	-2.87677	-1.34108
C	-1.74920	-4.09519	0.04959
H	4.11354	2.74784	4.05538
C	3.56665	4.19036	2.54181
C	2.02358	-0.09874	3.78391
C	4.34452	-0.20750	3.01437
H	2.91769	5.35628	0.84695
C	0.65683	4.47619	-0.86157
C	2.39130	3.06537	-1.84894
C	-4.62562	1.82135	-0.15267
C	-5.13422	2.36164	2.30300
H	-1.16873	2.64722	2.74542
H	-2.70440	3.42756	3.19290
H	-2.47329	1.70181	3.49676
C	-4.20503	1.76905	-2.65832
H	-0.32738	1.91541	-1.84621
H	-1.41324	0.73768	-2.61510
H	-1.61734	2.46477	-2.92397
H	6.63935	-2.34522	0.82154
C	6.46441	-1.02068	-0.86993
H	5.06759	-3.87117	1.55637
H	3.37297	-3.41057	1.27665
H	4.11599	-4.65930	0.27264
H	5.98916	0.12495	-2.63168
H	2.62150	-0.37571	-3.04437
H	4.11144	-0.14601	-3.99272
H	3.30079	-1.73321	-3.94857
H	-3.12660	-1.01305	-4.15289
C	-3.84413	-2.07724	-2.42021
H	0.28079	-0.75764	-3.54030
H	0.11031	-2.22878	-4.50056
H	-0.91998	-0.81628	-4.85332
H	-4.21591	-3.26728	-0.66293
H	-1.11368	-3.54487	0.76351
H	-2.64680	-4.43151	0.58470
H	-1.17559	-4.97987	-0.26132
H	4.03525	5.03399	3.05040
C	2.17232	-1.38203	4.32613
C	0.71411	0.62596	3.93471
C	4.44700	-1.48176	3.57355
C	5.52639	0.41077	2.31800
C	0.21063	4.78155	-2.15241
C	-0.03279	5.10459	0.32092
C	1.90726	3.39519	-3.11901
C	3.61869	2.20731	-1.71680
N	-5.89134	1.47887	-0.46394
H	-4.73745	3.09713	3.01437
H	-6.10171	2.72124	1.93439

H	-5.33364	1.43715	2.87120
H	-5.29856	1.71256	-2.67653
H	-3.89391	2.67387	-3.20639
H	-3.79445	0.91176	-3.20972
C	7.79181	-0.35053	-0.64034
C	-5.28998	-1.73016	-2.64655
H	1.32598	-1.83114	4.85301
C	3.36617	-2.09783	4.21687
H	0.85181	1.71456	3.95650
H	0.03711	0.40497	3.09544
H	0.20097	0.31670	4.85525
H	5.39890	-2.01403	3.49818
H	5.28985	0.64955	1.27002
H	5.83298	1.35206	2.79620
H	6.38198	-0.27496	2.32337
H	-0.64561	5.45275	-2.26460
C	0.81134	4.24569	-3.29405
H	-0.03529	4.42913	1.18426
H	0.46438	6.03942	0.62538
H	-1.07357	5.35169	0.07103
H	2.41453	2.98574	-3.99725
H	4.09966	2.06601	-2.69251
H	4.35161	2.65843	-1.03172
H	3.38051	1.21204	-1.31697
N	-6.91024	1.34657	0.33150
H	8.22660	0.01346	-1.58137
H	7.68269	0.51979	0.02760
H	8.51138	-1.03423	-0.16868
H	-5.51246	-1.62653	-3.71783
H	-5.95650	-2.49479	-2.22512
H	-5.55013	-0.77271	-2.16665
C	3.49277	-3.49382	4.76335
C	0.27379	4.54466	-4.66737
C	-7.16700	0.12814	0.90599
H	4.44099	-3.62966	5.30338
H	2.66855	-3.73381	5.44798
H	3.47602	-4.23563	3.94863
H	-0.22527	5.52295	-4.70034
H	1.07172	4.53736	-5.42305
H	-0.46850	3.78789	-4.97126
C	-8.37574	-0.01392	1.63397
C	-6.27344	-0.97575	0.83904
H	-9.05458	0.83839	1.67771
C	-8.68316	-1.20532	2.27281
C	-6.58234	-2.16757	1.47979
H	-5.33780	-0.87441	0.28959
H	-9.60819	-1.32908	2.83614
C	-7.78109	-2.26804	2.18613
H	-5.90666	-3.02337	1.44522
F	-8.07988	-3.43563	2.81125

6.5.25 *E*-11

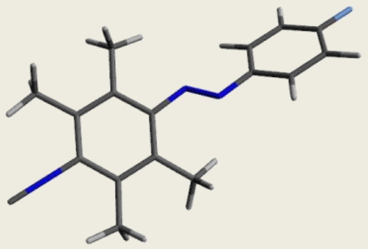

```
37
E-11 @ PBE-D3/def2-TZVP
C          -6.16791       -0.31391        0.17933
N          -4.99236       -0.20518        0.11447
C          -3.61744       -0.07606        0.02995
C          -3.05912        1.21631        0.10387
C          -2.84034       -1.24198       -0.14040
C          -1.66681        1.33851        0.00930
C          -3.92352        2.43141        0.28583
C          -1.44330       -1.11811       -0.22147
C          -3.50015       -2.59072       -0.22985
C          -0.86704        0.17519       -0.11464
C          -1.02957        2.69795        0.09535
H          -3.89104        3.07641       -0.60669
H          -4.96963        2.16476        0.46767
H          -3.57058        3.04194        1.12991
C          -0.62286       -2.35200       -0.48900
H          -2.98882       -3.32394        0.40896
H          -4.55197       -2.54624        0.07086
H          -3.46680       -2.98282       -1.25929
N           0.51664        0.41619       -0.20665
H          -1.03727        3.07341        1.13192
H           0.01446        2.65916       -0.23338
H          -1.57651        3.43156       -0.51328
H           0.33019       -2.11240       -0.97148
H          -0.37138       -2.88105        0.44280
H          -1.17518       -3.05067       -1.13110
N           1.26895       -0.42531        0.36563
C           2.65358       -0.19369        0.18754
C           3.19810        0.74466       -0.70901
C           3.50618       -0.99177        0.96546
H           2.52144        1.34463       -1.31738
C           4.57494        0.88829       -0.81438
C           4.88832       -0.85149        0.87372
H           3.05707       -1.71573        1.64664
H           5.02729        1.60123       -1.50428
C           5.39707        0.08884       -0.01713
H           5.56988       -1.45551        1.47210
F           6.73839        0.22964       -0.12390
```

6.5.26 *Z*-11

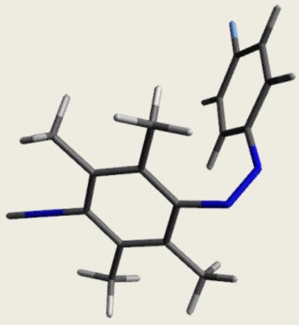

```
37
Z-11 @ PBE-D3/def2-TZVP
C           4.38658       -2.39727       -0.84563
N           3.55985       -1.62850       -0.49452
C           2.59685       -0.72400       -0.08317
C           2.62407        0.58057       -0.62131
C           1.64082       -1.14037        0.86708
C           1.64899        1.49154       -0.19463
C           3.66464        0.98836       -1.62571
C           0.65449       -0.22813        1.27645
C           1.66688       -2.53404        1.42907
C           0.65273        1.06422        0.70768
C           1.61605        2.89838       -0.72609
H           4.36887        1.71544       -1.19050
```

```
H          4.24755        0.13118       -1.97835
H          3.20316        1.47539       -2.49685
C         -0.34730       -0.63654        2.32191
H          0.67089       -2.99717        1.38231
H          2.37139       -3.17570        0.88999
H          1.96619       -2.52706        2.48960
N         -0.25921        2.06295        1.16503
H          1.12629        2.94831       -1.71343
H          1.05016        3.55048       -0.04809
H          2.62806        3.30556       -0.84982
H          0.13442       -1.22726        3.11266
H         -0.82610        0.22959        2.79200
H         -1.14692       -1.26184        1.89185
N         -1.50581        1.97540        1.06260
C         -2.15830        0.95185        0.31197
C         -1.70481        0.44411       -0.91863
C         -3.40702        0.54785        0.80996
H         -0.75970        0.78303       -1.33909
C         -2.47593       -0.47767       -1.61960
C         -4.16613       -0.40544        0.13818
H         -3.76175        0.99125        1.74125
H         -2.15446       -0.87193       -2.58365
C         -3.68409       -0.90267       -1.06956
H         -5.12710       -0.74802        0.52131
F         -4.42289       -1.81273       -1.74452
```

6.5.27 TS-11

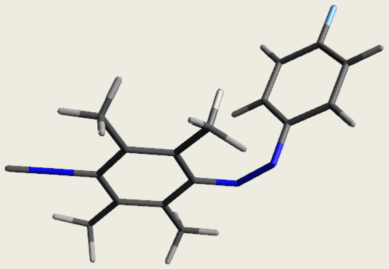

```
37
TS-11 @ UPBE-D3/def2-TZVP
C         -5.31415       -1.53239        1.19252
N         -4.31316       -1.05829        0.77368
C         -3.15301       -0.50014        0.28310
C         -3.02564        0.91077        0.29039
C         -2.14565       -1.36150       -0.22135
C         -1.85045        1.47286       -0.19916
C         -4.12665        1.78618        0.81896
C         -0.96952       -0.80678       -0.72952
C         -2.35476       -2.85236       -0.20695
C         -0.79364        0.62232       -0.67689
C         -1.67165        2.96330       -0.20134
H         -4.56069        2.40011        0.01401
H         -4.93544        1.20124        1.26837
H         -3.74324        2.48529        1.57672
C          0.07058       -1.69852       -1.34699
H         -1.46453       -3.37321        0.17087
H         -3.20753       -3.13237        0.41994
H         -2.55067       -3.23713       -1.22069
N          0.33244        1.26876       -1.05905
H         -1.57557        3.35442        0.82511
H         -0.77204        3.24799       -0.75697
H         -2.54313        3.46415       -0.64817
H         -0.37966       -2.62406       -1.72365
H          0.57994       -1.19791       -2.17995
H          0.85502       -1.97979       -0.62452
N          1.43879        0.77040       -1.49365
C          2.47449        0.45759       -0.65749
C          2.39630        0.57118        0.75709
```

C	3.67591	-0.01508	-1.24503
H	1.47967	0.94916	1.21001
C	3.47882	0.21427	1.54781
C	4.75366	-0.37684	-0.45291
H	3.72677	-0.09066	-2.33151
H	3.44215	0.29768	2.63443
C	4.64030	-0.25773	0.93437
H	5.68243	-0.74671	-0.88719
F	5.69449	-0.60746	1.70963

7 References

[1] C. B. Fischer, S. Xu, H. Zipse, *Chemistry* **2006**, *12*, 5779–5784.

[2] F. Reiß, A. Schulz, A. Villinger, N. Weding, *Dalton Trans.* **2010**, *39*, 9962.

[3] E. Zander, J. Bresien, V. V. Zhivonitko, J. Fessler, A. Villinger, D. Michalik, A. Schulz, *J. Am. Chem. Soc.* **2023**, *145*, 14484–14497.

[4] J. Bresien, T. Kröger-Badge, S. Lochbrunner, D. Michalik, H. Müller, A. Schulz, E. Zander, *Chem. Sci.* **2019**, *10*, 3486–3493.

[5] G. M. Sheldrick, *Acta Crystallogr. Sect. A Found. Adv.* **2015**, *71*, 3–8.

[6] G. M. Sheldrick, *Acta Crystallogr. Sect. C Struct. Chem.* **2015**, *71*, 3–8.

[7] G. M. Sheldrick, *SADABS Version 2*, University of Göttingen, Germany, **2004**.

[8] B. Priewisch, K. Rück-Braun, *J. Org. Chem.* **2005**, *70*, 2350–2352.

[9] S. Wu, J. Huang, S. Gazzarrini, S. He, L. Chen, J. Li, L. Xing, C. Li, L. Chen, C. G. Neochoritis, G. P. Liao, H. Zhou, A. Dömling, A. Moroni, W. Wang, *ChemMedChem* **2015**, *10*, 1837–1845.

[10] C. Feldmeier, H. Bartling, E. Riedle, R. M. Gschwind, *J. Magn. Reson.* **2013**, *232*, 39–44.

[11] *Gaussian 09, Revision E.01*, M. J. Frisch, G. W. Trucks, H. B. Schlegel, G. E. Scuseria, M. A. Robb, J. R. Cheeseman, G. Scalmani, V. Barone, B. Mennucci, G. A. Petersson, H. Nakatsuji, M. Caricato, X. Li, H. P. Hratchian, A. F. Izmaylov, J. Bloino, G. Zheng, J. L. Sonnenberg, M. Hada, M. Ehara, K. Toyota, R. Fukuda, J. Hasegawa, M. Ishida, T. Nakajima, Y. Honda, O. Kitao, H. Nakai, T. Vreven, J. A. Montgomery Jr., J. E. Peralta, F. Ogliaro, M. Bearpark, J. J. Heyd, E. Brothers, K. N. Kudin, V. N. Staroverov, T. Keith, R. Kobayashi, J. Normand, K. Raghavachari, A. Rendell, J. C. Burant, S. S. Iyengar, J. Tomasi, M. Cossi, N. Rega, J. M. Millam, M. Klene, J. E. Knox, J. B. Cross, V. Bakken, C. Adamo, J. Jaramillo, R. Gomperts, R. E. Stratmann, O. Yazyev, A. J. Austin, R. Cammi, C. Pomelli, J. W. Ochterski, R. L. Martin, K. Morokuma, V. G. Zakrzewski, G. A. Voth, P. Salvador, J. J. Dannenberg, S. Dapprich, A. D. Daniels, O. Farkas, J. B. Foresman, J. V. Ortiz, J. Cioslowski, D. J. Fox, Gaussian, Inc., Wallingford CT, **2013**.

[12] F. Neese, *WIREs Comput. Mol. Sci.* **2022**, *12*, 1–15.

[13] J. P. Perdew, K. Burke, M. Ernzerhof, *Phys. Rev. Lett.* **1996**, *77*, 3865–3868.

[14] J. P. Perdew, K. Burke, M. Ernzerhof, *Phys. Rev. Lett.* **1997**, *78*, 1396–1396.

[15] C. Adamo, V. Barone, *J. Chem. Phys.* **1999**, *110*, 6158–6170.

[16] S. Grimme, J. Antony, S. Ehrlich, H. Krieg, *J. Chem. Phys.* **2010**, *132*, 154104.

[17] S. Grimme, S. Ehrlich, L. Goerigk, *J. Comput. Chem.* **2011**, *32*, 1456–1465.

[18] F. Weigend, R. Ahlrichs, *Phys. Chem. Chem. Phys.* **2005**, *7*, 3297.

[19] F. Weigend, *Phys. Chem. Chem. Phys.* **2006**, *8*, 1057–1065.

[20] F. London, *J. Phys. le Radium* **1937**, *8*, 397–409.

[21] R. McWeeny, *Phys. Rev.* **1962**, *126*, 1028–1034.

[22] R. Ditchfield, *Mol. Phys.* **1974**, *27*, 789–807.

[23] K. Wolinski, J. F. Hinton, P. Pulay, *J. Am. Chem. Soc.* **1990**, *112*, 8251–8260.

[24] J. R. Cheeseman, G. W. Trucks, T. A. Keith, M. J. Frisch, *J. Chem. Phys.* **1996**, *104*, 5497–5509.

[25] A. Hellweg, C. Hättig, S. Höfener, W. Klopper, *Theor. Chem. Acc.* **2007**, *117*, 587–597.

[26] C. J. Jameson, A. De Dios, A. Keith Jameson, *Chem. Phys. Lett.* **1990**, *167*, 575–582.

[27] C. van Wüllen, *Phys. Chem. Chem. Phys.* **2000**, *2*, 2137–2144.

[28] W. Deng, J. R. Cheeseman, M. J. Frisch, *J. Chem. Theory Comput.* **2006**, *2*, 1028–1037.

[29] C. Riplinger, F. Neese, *J. Chem. Phys.* **2013**, *138*, 034106.

[30] D. G. Liakos, Y. Guo, F. Neese, *J. Phys. Chem. A* **2020**, *124*, 90–100.

[31] D. G. Liakos, M. Sparta, M. K. Kesharwani, J. M. L. Martin, F. Neese, *J. Chem. Theory Comput.* **2015**, *11*, 1525–1539.

[32] C. Riplinger, P. Pinski, U. Becker, E. F. Valeev, F. Neese, *J. Chem. Phys.* **2016**, *144*, 024109.

[33] Christopher J. Cramer, *Essentials of Computational Chemistry: Theories and Models*, John Wiley & Sons, Ltd, Chichester, UK, **2004**.

[34] C. Bannwarth, E. Caldeweyher, S. Ehlert, A. Hansen, P. Pracht, J. Seibert, S. Spicher, S. Grimme, *WIREs Comput. Mol. Sci.* **2021**, *11*, 1–49.

[35] C. Bannwarth, S. Ehlert, S. Grimme, *J. Chem. Theory Comput.* **2019**, *15*, 1652–1671.

[36] S. Grimme, *J. Chem. Theory Comput.* **2019**, *15*, 2847–2862.

[37] P. Pracht, F. Bohle, S. Grimme, *Phys. Chem. Chem. Phys.* **2020**, *22*, 7169–7192.

[38] S. Grimme, F. Bohle, A. Hansen, P. Pracht, S. Spicher, M. Stahn, *J. Phys. Chem. A* **2021**, *125*, 4039–4054.

[39] S. Grimme, *J. Comput. Chem.* **2006**, *27*, 1787–1799.

[40] D. P. Chong, *Recent Advances in Density Functional Methods*, WORLD SCIENTIFIC, Singapore, **1995**.

[41] E. Runge, E. K. U. Gross, *Phys. Rev. Lett.* **1984**, *52*, 997–1000.

[42] R. E. Stratmann, G. E. Scuseria, M. J. Frisch, *J. Chem. Phys.* **1998**, *109*, 8218–8224.

[43] T. Lu, F. Chen, *J. Comput. Chem.* **2012**, *33*, 580–592.
